# Synthesis and *h*LDH Inhibitory Activity of Analogues to Natural Products with 2,8-Dioxabicyclo[3.3.1]nonane Scaffold

**DOI:** 10.3390/ijms24129925

**Published:** 2023-06-08

**Authors:** Sofía Salido, Alfonso Alejo-Armijo, Joaquín Altarejos

**Affiliations:** Departamento de Química Inorgánica y Orgánica, Facultad de Ciencias Experimentales, Universidad de Jaén, Campus de Excelencia Internacional Agroalimentario ceiA3, 23071 Jaén, Spain

**Keywords:** selective *h*LDHA inhibitors, 2,8-dioxabicyclo[3.3.1]nonane core, flavylium salts, *h*LDHB, structure-activity analysis, A-type proanthocyanidins analogues

## Abstract

Human lactate dehydrogenase (*h*LDH) is a tetrameric enzyme present in almost all tissues. Among its five different isoforms, *h*LDHA and *h*LDHB are the predominant ones. In the last few years, *h*LDHA has emerged as a therapeutic target for the treatment of several kinds of disorders, including cancer and primary hyperoxaluria. *h*LDHA inhibition has been clinically validated as a safe therapeutic method and clinical trials using biotechnological approaches are currently being evaluated. Despite the well-known advantages of pharmacological treatments based on small-molecule drugs, few compounds are currently in preclinical stage. We have recently reported the detection of some 2,8-dioxabicyclo[3.3.1]nonane core derivatives as new *h*LDHA inhibitors. Here, we extended our work synthesizing a large number of derivatives (**42**–**70**) by reaction between flavylium salts (**27**–**35**) and several nucleophiles (**36**–**41**). Nine 2,8-dioxabicyclo[3.3.1]nonane derivatives showed IC_50_ values lower than 10 µM against *h*LDHA and better activity than our previously reported compound **2**. In order to know the selectivity of the synthesized compounds against *h*LDHA, their *h*LDHB inhibitory activities were also measured. In particular, compounds **58**, **62a**, **65b**, and **68a** have shown the lowest IC_50_ values against *h*LDHA (3.6–12.0 µM) and the highest selectivity rate (>25). Structure–activity relationships have been deduced. Kinetic studies using a Lineweaver–Burk double-reciprocal plot have indicated that both enantiomers of **68a** and **68b** behave as noncompetitive inhibitors on *h*LDHA enzyme.

## 1. Introduction

Lactate dehydrogenase (LDH; EC 1.1.1.27) is an enzyme widely distributed in nature that belongs to the 2-hydroxyacid oxidoreductases family. It is a tetrameric molecule (140 kDa) mainly formed by two different kinds of subunits of 35 kDa each: the M-type (muscle) subunit encoded by *Ldha* gene and the H-type (heart) encoded by *Ldhb* gene. The combination of both subunits provides up to five different LDH isoforms: H_4_ (also named LDH-1 or LDHB), H_3_M (LDH-2), H_2_M_2_ (LDH-3), HM_3_ (LDH-4), and M_4_ (LDH-5 or LDHA). All of them show different kinetic behaviors and tissue distributions. The homotetramers LDHA and LDHB are the major isozymes that are present in human cells and are mainly located in (i) skeletal muscle and liver, and (ii) in heart and brain tissues, respectively [1,2].

This enzyme plays an important role in several metabolic pathways where it regulates the homeostasis of pyruvate/lactate, hydroxypyruvate/glycerate, and oxalate/glyoxylate, using NADH as cofactor. One of the main and most studied roles of LDH enzymes is the regulation of the energy supply mechanism in cancer and normal cells (Warburg effect) [3].

Non-cancer cells obtain their energy demand by the complete metabolism of glucose, which is performed in two subsequent steps: (i) conversion of glucose into pyruvate (glycolysis), and (ii) degradation of the generated pyruvate to CO_2_ (tricarboxylic acid cycle (TCA)). The complete catabolism of glucose allows a sustainable energy production system due to the generation of ATP molecules and the regeneration of NAD^+^ cofactor during the oxidative phosphorylation (OXPHOS).

LDH and pyruvate dehydrogenase kinase (PDHK) enzymes are in charge of regulating the flux of pyruvate into TCA cycle [4]. The LDH function becomes very important when oxygen supply is reduced and/or cells increase their glucose demand and, therefore, the fermentation pathway is activated. In this case, pyruvate is reduced into lactate, regenerating NAD^+^ and producing ATP molecules but in a less efficient way. This last reaction is catalyzed by LDH enzymes and allows the maintenance of the equilibrium when glucose uptake is increased.

On the opposite hand, cancer cells need precursors to build up macromolecules in a quicker way. In that sense, and trying to fulfill this requirement, they increase their glucose uptake and adjust its metabolism in favor of a quicker but less efficient process of obtaining energy [5], in which lactic acid fermentation and LDH enzymes play the main role even in the presence of oxygen (aerobic glycolysis). As a result, high levels of glycolytic metabolites are formed, which can be used for the quick development of cancer cells [6]. This change in the metabolic glucose pathway causes the need for overexpressed LDH enzymes in cancer tissues, turning these enzymes, and particularly LDHA isozyme, into a possible therapeutic target for the development of new anticancer therapies [7].

Moreover, apart from its relevance in cancer, human LDHA isozyme (*h*LDHA) has recently attracted great interest among scientists, due to its proven important role in molecular mechanism of different kind of disorders [8], such as vascular diseases [9,10], epilepsy [11,12], tuberculosis [13], pulmonary fibrosis [14], arthritis [15] and other inflammatory diseases [16,17], and in primary hyperoxalurias (PHs) [18]. Recent advances in the understanding of the molecular mechanisms of several diseases have led to the development of novel therapeutic approaches, such as enzyme deletion using in vivo CRISPR/Cas9 [19,20,21] or *m*RNA silencing using *si*RNA [22,23,24,25,26]. However, pharmacological treatments based on small-molecule drugs could present some advantages compared to the use of biotechnological approaches. The cost of production is generally lower, and they can be administered orally and present better ADME (absorption, distribution, metabolism, and excretion) properties. In any case, a combined treatment with both types of pharmacological agents could have synergistic effect and benefits, such as lowering the required doses or the frequency of administration. Actually, approaches based on attenuated expression of LDHA or inhibition of LDHA activity by small-molecule drugs appear as emerging strategies for the treatment of these diseases [27,28,29,30,31,32,33,34,35,36,37,38,39,40,41,42,43]. Pharmaceutical companies and researchers in academia are investing significant efforts to identify natural or synthetic *h*LDHA inhibitors with a huge structural variability. Although some of them have shown EC_50_ values in cancer cell line at nanomolar range [33], limitations related to ADME and pharmacokinetic properties have reduced the number of compounds evaluated in vivo and, to the best of our knowledge, none of them have progressed towards clinical trial [27,28].

**Figure 1:**
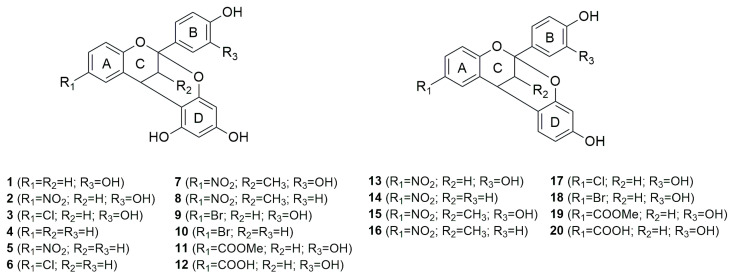
Structure of (±)-2,8-dioxabicyclo[3.3.1]nonane derivatives previously synthesized and evaluated as *h*LDHA inhibitors by us [35].

Our research group is also interested in the development of *h*LDHA inhibitors as a pharmacological option for the treatment of PHs, a rare life-threatening genetic disease [34,35]. We recently reported the detection of (±)-2,8-dioxabicyclo[3.3.1]nonane derivatives, analogues to A-type proanthocyanidin natural products, as a new family of *h*LDHA inhibitors [35]. That research was inspired by the results obtained by Li et al., who performed LDHA inhibition assays on procyanidin-enriched fractions from *Spatholobus suberectus* extract [44], and by our experience in the synthesis of compounds with this bicyclic scaffold [45,46,47]. Thus, we have reported the synthesis of twenty (±)-2,8-dioxabicyclo[3.3.1]nonane derivatives (**1**–**20**) (Figure 1), their *h*LDHA and *h*LDHB inhibitory activities, and the ability of three of them (**2**, **3** and **9**) to reduce the quantity of oxalate generated by hyperoxaluric mouse hepatocytes (PH1, PH2, and PH3) in vitro [35].

These results encouraged us to continue exploring this family of bicyclic compounds and to synthesize and evaluate a more complete collection of 2,8-dioxabicyclo[3.3.1]nonane derivatives in order to establish structure−activity relationships that allow to understand the main features that bicyclic core should fulfil to ensure high potency and selectivity towards *h*LDHA.

## 2. Results and Discussion

### 2.1. Chemical Synthesis

**Scheme 1:**
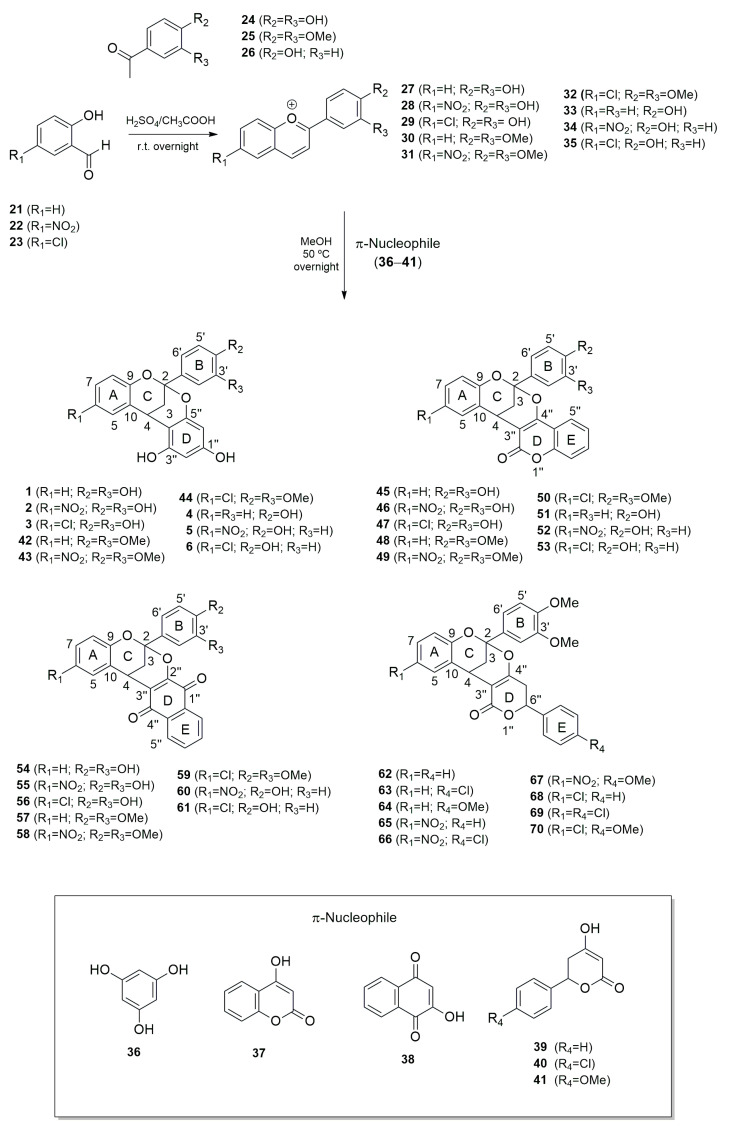
Synthesis of flavylium salts (**27**–**35**) and (±)-2,8-dioxabicyclo[3.3.1]nonane derivatives (**1**–**6**; **42**–**70**).

Our research group previously synthesized (±)-2,8-dioxabicyclo[3.3.1]nonane derivatives by reaction of flavylium salts with phenolic nucleophiles, such as phloroglucinol or resorcinol [45,47]. The fact that these compounds have emerged as a new family of *h*LDHA selective inhibitors [35], encouraged us to systematically design a series of 2,8-dioxabicyclo[3.3.1]nonane compounds in order to gain insight into the structural requirements that are responsible for *h*LDHA activity and *h*LDHA/*h*LDHB selectivity. In that sense, 44 bicyclic derivatives (**1**–**6**; **42**–**70**) (35 are new compounds) have been prepared by reaction between 9 different flavylium salts (**27**–**35**) (3 are new compounds), with modifications at A- and B-rings, and phloroglucinol (**36**) and other non-phenolic π-nucleophiles (**37**–**41**) (Scheme 1). Nucleophiles **36**–**38** are commercially available, but pyrone-type compounds **39**–**41** have been prepared as racemic mixtures according to a previously reported methodology by reaction of the proper benzaldehyde derivative and ethyl acetoacetate [48,49]. The yields obtained and the spectroscopic data of these pyrone derivatives are consistent with the reported ones (**39** (88%) [48], **40** (80%) [48], and **41** (74%) [48,50]).

Flavylium salts were synthesized by an aldol condensation under acidic conditions between salicylic aldehyde (**21**) or derivatives (**22** and **23**) and acetophenone derivatives (**24**–**26**) according to procedures previously used by us [35,45,51]. Three different substituents have been selected at position 6 of the A-ring (R_1_ at Scheme 1) with different electronic effects on the bicyclic core. Strong and weak electron withdrawing groups (EWGs: NO_2_ and Cl, respectively) along with the absence of a substituent in A-ring have been employed. For the B-ring, OH and OMe groups have been selected at position 3′ and/or 4′ (R_2_ and R_3_ at Scheme 1), taking into account that hydroxyl groups always appear in polyphenolic scaffold-based LDHA inhibitors (LDHAi’s) [31]. All flavylium salts were obtained with moderate to quantitative yields (63–99%; Table 1). It is worth noting that the three new flavylium salts with two OMe groups in B-ring (**30**–**32**) were obtained with the highest yields (96–99%).

The synthesis of (±)-2,8-dioxabicyclo[3.3.1]nonane derivatives was carried out by a sequential double addition of the nucleophilic moiety (**36**–**41**, Scheme 1) to the electrophilic moiety of the flavylium salt (**27**–**35**, Scheme 1), according to procedures previously used and optimized by us [35,45]. Although the addition of commercially available nucleophiles, phloroglucinol (**36**) [35,45,52], 4-hydroxycoumarin (**37**) [53], or 2-hydroxy-1,4-naphthoquinone (**38**) [54] as nucleophilic moiety to achieve the synthesis of (±)-2,8-dioxabicyclo[3.3.1]nonane have been previously reported, to the best of our knowledge, it is the first time that pyrone derivatives, such as compounds **39**–**41**, have been employed to obtain the corresponding bicyclic structures (compounds **62**–**70**, Scheme 1).

**Figure 2:**
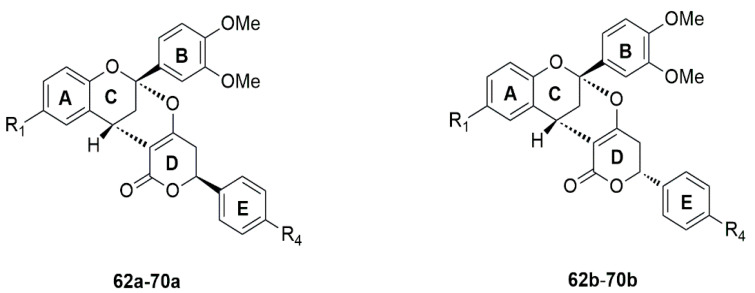
Structures of (±)-2,8-dioxabicyclo[3.3.1]nonane derivatives with pyrone-type moieties (**62**–**70**).

As gathered in Table 1, the highest yields obtained in these nucleophilic addition reactions (54–81%) corresponded to the synthesis of (±)-2,8-dioxabicyclo[3.3.1]nonane derivatives with a 4-hydroxycoumarin moiety (**45**–**53**), the presence or absence of electron-withdrawing groups (EWGs) at A-ring (NO_2_, Cl) being not clearly reflected in the yields. However, the nature of the group at A-ring does influence the yields of derivatives with a phloroglucinol moiety. Thus, compounds **2**, **3**, **5**, **6**, **43**, and **44**, containing EWGs (NO_2_ or Cl) at A-ring have been obtained with moderate yields (42–64%) versus compounds **1**, **4**, and **42**, with no substituents at A-ring, which have been obtained with lower yields (22–38%). This behavior had already been observed previously by us [35] and the same trend was noticed when 2-hydroxy-1,4-naphthoquinone (**38**) was used as a nucleophile (derivatives **54**–**61**). On the other hand, (±)-2,8-dioxabicyclo[3.3.1]nonane derivatives with pyrone-type moieties (**62**–**70**) have been synthesized as mixtures of two diastereomers since racemic 4-hydroxy-6-phenyl-5,6-dihydro-2*H*-pyran-2-one (**39**), 6-(4-chlorophenyl)-4-hydroxy-5,6-dihydro-2*H*-pyran-2-one (**40**), or 4-hydroxy-6-(4-methoxyphenyl)-5,6-dihydro-2*H*-pyran-2-one (**41**) were used as nucleophiles. These mixtures of diastereomers have been obtained with moderate yields (32–45%), except for derivative **62** (14%). Chromatographic separations of these mixtures led to pure (±)-2,8-dioxabicyclo[3.3.1]nonane derivatives **62a**–**70a** and **62b**–**70b** (Figure 2).

**Figure 3:**
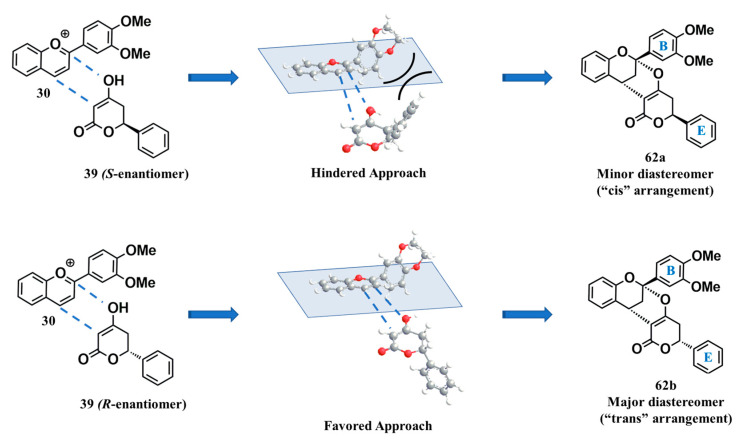
Proposal of the unfavored approach of the *S* enantiomer of the pyrone-type nucleophile **39** to the flavylium salt **30** to give the “*cis*” minor diastereomer, and the more favored approach of the *R* enantiomer to **30** to give the “*trans*” major diastereomer (the approach of the same nucleophiles through the top face of the flavylium salt has not been illustrated).

A diastereomeric excess has been observed for compounds **62**–**70** in all cases, in favor of diastereomers **62b**–**70b** (1:1.2–1:5.5, entries **37**–**45**, Table 1) that have a “*trans*” relative arrangement of rings B and E (Figure 2). A rationale of these results can be found in the less favored approach of one of the enantiomers of the nucleophile to the flavylium salt to give the “*cis*” diastereomer versus the more favored approach of the other enantiomer to the same face of the salt to give the “*trans*” diastereomer (Figure 3).

All synthesized compounds were totally characterized using spectroscopic and spectrometric techniques such as ^1^H NMR, ^13^C NMR, 2D NMR, and HRMS (^1^H NMR and ^13^C NMR spectra are included in the Supplementary Material Figures S1–S82). Moreover, their purities were previously checked by HPLC (chromatograms of all byciclic compounds are included in the Supplementary Material Figures S83–S132). Flavylium salts **27** [55], **28** [46], **29** [35], **33** [56], **34** [57], and **35** [35], and (±)-dioxabicyclic derivatives **1**–**2** [52], **3**–**6** [35], **48** [53], **51** [53], and **57** [54] were described previously and their spectroscopic data agreed with those reported in the literature. On the other hand, flavylium salts **30**–**32** and bicycles **42**–**47**, **49**–**50**, **52**–**56**, and **58**–**70** are new compounds and their structural characterization is reported for the first time

### 2.2. Inhibitory Activity of (±)-2,8-Dioxabicyclo[3.3.1]Nonanes 1–6, 42–70 against hLDHA and hLDHB

The inhibitory activity of all synthetized byciclic derivatives (**1**–**6**, **42**–**70**) against both enzymes *h*LDHA and *h*LDHB have been measured by a kinetic spectrofluorimetric assay [58]. Briefly, the decrease in the coenzyme β-NADH fluorescence (λ_emission_ = 460 nm) was measured for 10 min, at different concentration of inhibitors, and its slope was compared with the one obtained when there were not inhibitors added (100% enzymatic activity). The inhibitory activity of the synthetized compounds is expressed as the concentration needed to reduce the enzymatic activity of *h*LDHA or *h*LDHB up to 50% (IC_50_). The dose response curves against *h*LDHA and *h*LDHB of all new byciclic compounds are included in the Supplementary Material (Figures S133–S198). 

All synthesized bicyclic compounds, except seven of them (**1**, **4**, **42**, **63b**, **64a**, **64b**, and **70a**), showed IC_50_ values against *h*LDHA in the low micromolar range (3.6–50 μM), and it is also worth highlighting that the inhibitory activity of ten bicyclic derivatives (**2**, **43**, **44**, **47**, **58**, **60**, **62a**, **65b**, **66a**, and **66b**) showed IC_50_ values lower than 10 µM (Table 2). Among the 2,8-dioxabicyclo[3.3.1]nonane derivatives with phloroglucinol moiety, compounds **43** and **44** have shown the highest activity, being similar to compound **2**, one of the most active compounds previously reported by us [35]. In the case of derivatives with 4-hydroxycoumarin and 2-hydroxy-1,4-naphthoquinone moieties, only one compound (**47**) and two compounds (**58** and **60**), respectively, showed better activity than **2**. However, four derivatives with pyrone-type moiety (**62a**, **65b**, **66a**, and **66b**) showed better activity than **2**. In fact, the most active *h*LDHA inhibitor synthesized is **62a** (IC_50_ = 3.6 µM), a 2,8-dioxabicyclo[3.3.1]nonane derivative with a 4-hydroxy-6-phenyl-5,6-dihydro-2*H*-pyran-2-one moiety (**39**), and two methoxy groups at B-ring as substituents (Table 2).

**Figure 4:**
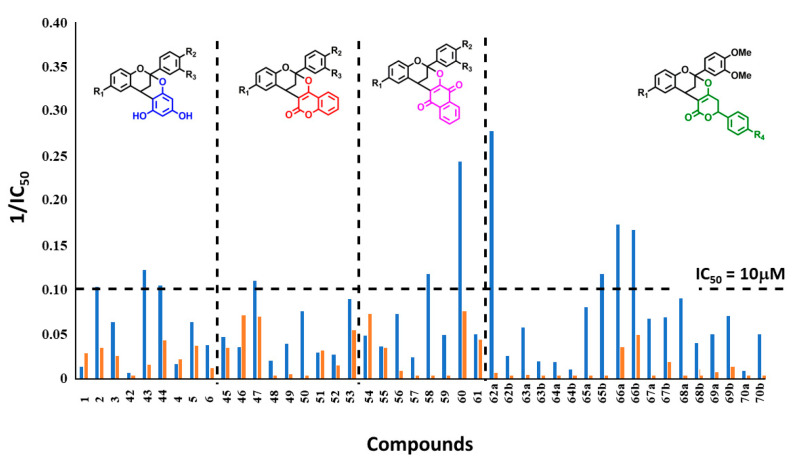
Inhibitory activity of (±)-2,8-dioxabicyclo[3.3.1]nonane derivatives (**1**–**6**, **42**–**70**) against *h*LDHA (blue) and *h*LDHB (orange) expressed as the inverse of IC_50_ (1/IC_50_).

Although inhibition potency (IC_50_ against *h*LDHA) is an important parameter that characterize an inhibitor, its selectivity through the target enzyme governs its applicability and it is a feature that should be also taken into account. In order to know the selectivity of the synthesized compounds against *h*LDHA, their *h*LDHB inhibitory activities were also measured. In Figure 4, the inverse of IC_50_ for each compound against both enzymes is represented for the sake of clarity. All of them showed higher inhibitory activity (higher 1/IC_50_ values) against *h*LDHA than against *h*LDHB, except for compounds **1**, **4**, **46**, **51**, and **54**.

**Figure 5:**
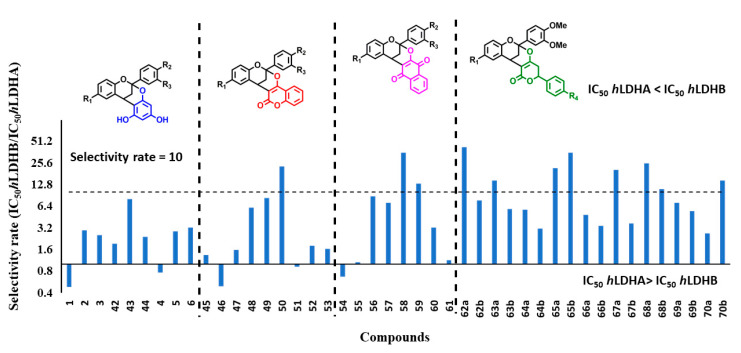
Selectivity rate of 2,8-dioxabicyclo[3.3.1]nonane inhibitors (**1**–**6**, **42**–**70**) calculated as: IC_50_ values against *h*LDHB/IC_50_values against *h*LDHA.

Figure 5 illustrates the selectivity rate, defined as the ratio between IC_50_ values against *h*LDHB and IC_50_ values against *h*LDHA (Table 2), for every 2,8-dioxabicyclo[3.3.1]nonane derivative synthetized (**1**–**6**, **42**–**70**). High selectivity rate values (>10) have been observed for eleven compounds, one with 4-hydroxycoumarin (**50**), two with 2-hydroxy-1,4-naphthoquinone (**58** and **59**), and eight with a pyrone-type moieties (**62a**, **63a**, **65a**, **65b**, **67a**, **68a**, **68b**, and **70b**). In particular, compounds **58**, **62a**, **65b**, and **68a** have shown the lowest IC_50_ values against *h*LDHA (3.6–12.0 µM) and the highest selectivity rate (>25), which allow us to select them as hits for future structural optimization. These data seem to demonstrate that the use of pyrone derivatives as nucleophilic moiety is an important structural feature for ensuring high potency and selectivity against *h*LDHA.

Regarding the behavior of both diastereomers of compounds **62**–**70**, all of them showed better inhibition activity against *h*LDHA than against *h*LDHB (Figure 4), and in terms of selectivity (Figure 5), a slightly higher selectivity is observed for the “*cis*” than for the “*trans*” isomers, with the exception of compounds **65** and **70** (Figure 5). This means that a clear influence of the relative arrangement of rings B and E of compounds **62**–**70** cannot be established yet with the current data.

**Figure 6:**
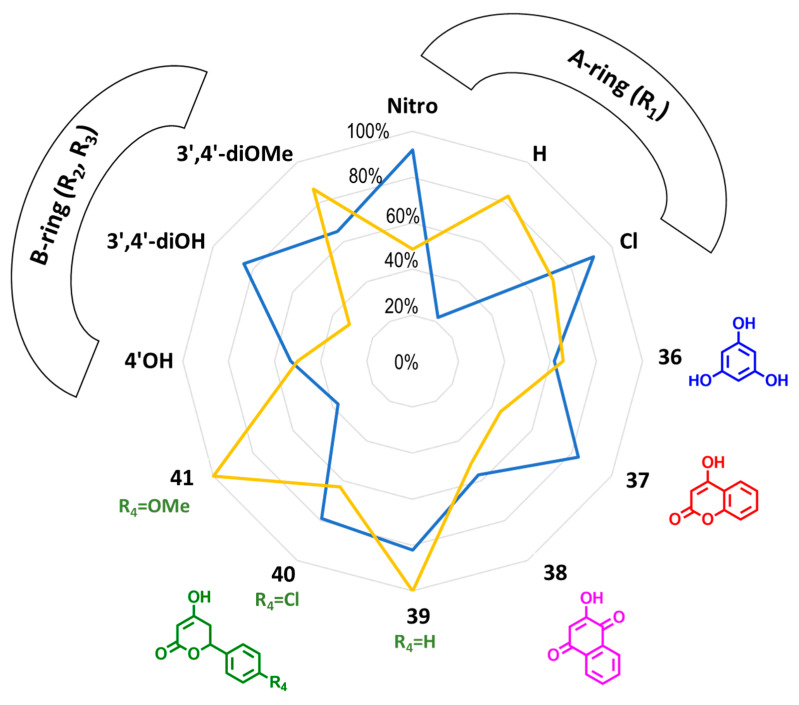
Structure–activity relationship of bicyclic compounds against *h*LDHA and *h*LDHB. **Blue line**: number of compounds (expressed in percentage) with a *h*LDHA inhibition activity lower than 30 μM. **Yellow line**: number of compounds (expressed in percentage) with a *h*LDHB inhibition activity higher than 30 μM.

According to these inhibitory activity and selectivity results, a preliminary structure–activity analysis can be made. Figure 6 shows the number of compounds (in percentage) with a specific structural moiety (substituents at A- and B-rings and nucleophilic moiety) that meet the requirement of a selected inhibition activity (blue line, *h*LDHA inhibition activity lower than 30 μM; yellow line, *h*LDHB inhibition activity higher than 30 μM). It is deduced from the graphic that the presence of EWGs (NO_2_ and Cl) at A-ring is an important feature to ensure higher inhibition activity against *h*LDHA. Percentagewise, the number of compounds with a nitro or chloro substituent at A-ring and IC_50_ values against *h*LDHA lower than 30 µM is much higher than those without a substituent at A-ring. In addition, the number of compounds with chloro at A-ring and *h*LDHB inhibition activity higher than 30 μM is also higher than those with a nitro substituent. This means that derivatives with Cl at A-ring show better selectivity rate.

Regarding B-ring substituents, the percentage of compounds with two oxygenated groups (R_2_=R_3_=OH or OMe) and IC_50_ values against *h*LDHA lower than 30 µM is higher than those with just one group (R_2_=OH; R_3_=H). Within this subgroup of deoxygenated compounds, the percentage of cathecol derivatives (R_2_=R_3_=OH) with *h*LDHB inhibition activity higher than 30 μM is lower, pointing out a decrease in the selectivity when free hydroxyl groups are present.

Finally, the influence of the nucleophilic moiety on the inhibitory activity against *h*LDHA and on the selectivity rate is remarkable (Figure 6). Percentagewise, the highest number of compounds with low IC_50_ values against *h*LDHA corresponds to those with 4-hydroxycoumarin (**37**), 4-hydroxy-6-phenyl-5,6-dihydro-2*H*-pyran-2-one (**39**), or 6-(4-chlorophenyl)-4-hydroxy-5,6-dihydro-2*H*-pyran-2-one (**40**) moieties. In particular, the substitution pattern presented in nucleophile **39** favors a greater number of compounds with IC_50_ values against *h*LDHB higher than 30 μM, thereby increasing its selectivity as happen for compounds **62**, **65**, or **68**.

### 2.3. Resolution of Racemates and Inhibitory Activity of 2,8-Dioxabicyclo[3.3.1]nonanes (+)-66a, (−)-66a, (+)-68a, (−)-68a, (+)-68b, and (−)-68b against hLDHA and hLDHB

Some of the most active and selective racemic compounds synthesized (**66a**, **68a**, and **68b**) were purified by chiral HPLC, using a Chiralpak IC column and hexane and dicholomethane (30:70, *v*/*v*) as mobile phase. The proper purity of each enantiomer has been also checked by HPLC (chromatograms of each pure enantiomer are included in the Supplementary Material Figures S127–S132), using a Chiralpak IC analytical column and by the measurement of their optical rotation. The inhibition activity of pure enantiomers has been determined against both enzymes, *h*LDHA and *h*LDHB (Table 3). The dose response curves against *h*LDHA and *h*LDHB of each pure enantiomer are included in the Supplementary Material (Figures S199–S206).

According to the results obtained, it seems that the spatial influence of the substituents in the selected 2,8-dioxabicyclo[3.3.1]nonane derivatives for the enzymatic inhibition assays is not very high due to the similar values of IC_50_ obtained for each pair of enantiomers. Moreover, the inhibition activity of each single enantiomer was also very similar to the one obtained by its corresponding racemic mixture. For the *h*LDHA inhibition test, it seems that a better behavior is observed for dextrorotarory enantiomers, especially for compounds **66a** and **68a** (1.5 times more potent). For the *h*LDHB inhibition test, only compound **66a** was active and both enantiomers showed also similar potency.

It seems that the relative “*cis–trans*” arrangement of B and E ring in pyrone-type bicycle derivatives (**62a**–**70a** vs. **62b**–**70b**) plays a more important role in the enzymatic inhibition assays performed than the different absolute configurations observed for pure enantiomers.

### 2.4. Mechanism of hLDHA and hLDHB Inhibition

In order to explore the mechanism of inhibition of some of the most active compounds on *h*LDHA, both enantiomers of the racemate **68a** and **68b** have been selected. Assays measuring the enzymatic activity of both compounds in the presence of four different inhibitor concentrations and without an inhibitor and eight different substrate (pyruvate) concentrations have been measured by a similar kinetic spectrofluorimetric procedure to that described to calculate IC_50_ values against *h*LDHA (Section 3.4). The initial velocity (*V*_0_) was determined as the maximum slope per minute calculated in the linear interval of the graph representing the progression of the enzymatic reaction at each inhibitor and substrate concentration. The progression of the enzymatic reaction was monitored by the decrease in fluorescence, due to NADH conversion into NAD^+^, registered at λ_ex_ 340 nm and λ_em_ 460 nm every 60 s during 10 min. Nonlinear fits of *V*_0_ versus substrate concentration (Michaelis–Menten type) were performed for each inhibitor concentration and without inhibitor to determine *V_max_* and *K_M_* at each curve (values of *V_max_* and *K_M_* are included in the Supplementary Material Tables S1–S4). A decrease in apparent *V_max_* and no effect on the apparent *K_M_* were observed (with increasing concentrations of both enantiomers of racemate **68a** and **68b**), which is indicative of a noncompetitive inhibition. In addition, their corresponding Lineweaver–Burk double-reciprocal graphs showed intersection on the x axis, which is also favorable to this type of inhibition (graphs included in the Supplementary Material Figures S207–S210). Finally, a noncompetitive inhibition fit of *V*_0_ versus substrate concentration allowed us to obtain the *K_i_* values, 4.1 and 16.1 μM for dextrorotatory **68a** and levorotatory **68a**, respectively, and 35.9 and 76.9 μM for dextrorotatory **68b** and levorotatory **68b**, respectively. Noncompetitive inhibition is not affected by the substrate concentration and this mechanism could suggest an allosteric binding to the enzyme, which has already been suggested by others [59].

## 3. Materials and Methods

### 3.1. General Experimental Methods

All solvents and reagents used in the synthesis or biological evaluations were purchased and were used as received, except MeOH, which was previously dried, using standard methodologies [60].

All reactions were conducted under inert conditions at room temperature or at 50 °C. Reactions were monitored by analytical thin-layer chromatography (TLC) on silica gel 60 F_254_ precoated aluminum sheets (0.25 mm, Merck Chemicals, Darmsdadt, Germany) and spots were detected using UV light (λ = 254 nm). Purification steps of synthesized racemic compounds were carried out by column chromatography (CC) using Sephadex LH-20 or Silica gel 60 (particle size 0.040–0.063 mm) (Merck Chemicals, Darmsdadt, Germany). Purity grade of synthesized racemic compounds were performed on a Waters 600E analytical HPLC (Waters Chromatography Division, Milford, MA, USA) that had a diode array detector (DAD), operating in the range of 190–800 nm (Waters CapLC 2996 Photodiode Array Detector, Waters Chromatography Division, Milford, MA, USA), at 30 °C and using a C18 reversed-phase Spherisorb ODS-2 column, 250 mm × 3 mm i.d., 5 μm (Waters Chromatography Division, Milford, MA, USA). The best separation conditions were accomplished with H_2_O:CH_3_COOH, 99.8:0.2, *v*/*v* (solvent A) and CH_3_CN:CH_3_COOH, 99.8:0.2, *v*/*v* (solvent B) at a flow rate of 0.7 mL/min: linear gradient from 20% to 80% B for 20 min; 80% B for 20 min; and 5 min to come back to the initial conditions. Purity data of the compounds were measured at 280 nm and are included in the Supplementary Material section. Chiral HPLC purifications were conducted on the same HPLC equipment previously described using a Chiralpak IC column, (250 mm × 10 mm i.d., 5 μm (Daicel Corporation, Illkrich, France). The best separation was achieved with 30% of hexane (solvent A) and 70% of dichloromethane (solvent B) at a flow rate of 8 mL/min for 30 min. The purity of the separated enantiomers has been checked by analytical HPLC using a Chiralpak IC column, (250 mm × 3 mm i.d., 5 μm (Daicel Corporation, Illkrich, France). Optical rotations ([α]_D_) were recorded on a Jasco P-2000 automatic polarimeter (Jasco Analytical Instruments, Easton, MD, USA), using quartz cells of 1 dm path length, in methanol solutions.

^1^H NMR and ^13^C NMR spectra were measured on a Bruker Avance 400 spectrometer (Bruker Daltonik GmbH, Bremen, Germany) operating at 400 and 100 MHz for ^1^H and ^13^C, respectively. Deuterated methanol (CD_3_OD), deuterated chloroform (CDCl_3_), or deuterated dimethylsulfoxide ((CD_3_)_2_SO) was used as solvents. For flavylium salts a drop of TFA-*d* (trifluoroacetic acid deuterated) was added to obtain acidic conditions. Chemical shifts (in ppm) were referenced to solvent peaks as internal reference. The coupling constants (*J*) are expressed in hertz (Hz). The coupling system is described by using the following abbreviations: *d*, doublet; *t*, triplet; *m*, multiplet; *br s*, broad singlet; *br d*, broad doublet; *dd*, doublet of doublets; *ddd*, doublet of doublet of doublets; *td*, triplet of doublets; *dq*, doublet of quartets. The total assignment of ^1^H and ^13^C signals was made using 2D NMR spectra such as COSY, HSQC, and HMBC. High-resolution mass spectra (HRMS) were conducted on an Agilent 6520B Quadrupole time-of-flight (QTOF) mass spectrometer (Agilent Technologies, Santa Clara, CA, USA) with an electrospray ionization (ESI) interface operating in positive or negative mode.

### 3.2. General Methodology A Applied to Synthesize Flavylium Salts (***27***–***35***)

In a round flask was added the salicylic aldehyde derivative (2 mmol), the acetophenone derivative (2 mmol), 98% H_2_SO_4_ (0.6 mL; 10.8 mmol), and HOAc (2.6 mL). The mixture was stirred during one night at room temperature according to procedures previously used and described by us [35,45,46,51]. Then, 30 mL of Et_2_O was slowly added and a reddish solid precipitated. This solid was filtered off and carefully washed with more Et_2_O and dried. The flavylium salts **27** (0.605 g, 90% yield), **28** (0.586 g, 77% yield), **29** (0.608 g, 79%), **33** (0.506 g, 79% yield), **34** (0.555 g, 76% yield), and **35** (0.447 g, 63% yield) were previously described with comparable yields and their structures could be confirmed by analogy to their spectral data with those reported in the literature: **27 [55]**, **28 [46]**, **29** [35], **33** [56], **34** [57], and **35 [35]**.

#### 3.2.1. 3′,4′-Dimethoxyflavylium Hydrogen Sulfate (**30**)

General methodology A was applied using 2-hydroxybenzaldehyde (**21**, 0.217 mL, 2 mmol) and 3′,4′-dimethoxyacetophenone (**25**, 0.360 g, 2 mmol). Pure compound **30** was synthesized as a red solid (0.700 g, 96% yield). Melting point: 193–195 °C; ^1^H NMR (400 MHz, (CD_3_)_2_SO/TFA-*d*, pD ≈ 1.0) δ 9.45 (*d*, *J* = 9.1 Hz, 1H, H-4), 8.99 (*d*, *J* = 9.1 Hz, 1H, H-3), 8.51–8.38 (*m*, 2H, H-8, H-6′), 8.33 (*dd*, *J* = 8.2, 1.6 Hz, 1H, H-5), 8.26 (*ddd*, *J* = 8.7, 7.2, 1.6 Hz, 1H, H-7), 8.07 (*d*, *J* = 2.3 Hz, 1H, H-2′), 7.92 (*t*, *J* = 7.5 Hz, 1H, H-6), 7.38 (*d*, *J* = 8.9 Hz, 1H, H-5′), 4.01 (*s*, 3H, 4′-OMe) 3.98 (*s*, 3H, 3′-OMe); ^13^C NMR (100 MHz, DMSO/TFA-*d*, pD ≈ 1.0) δ 174.7 (C-2), 158.6 (C-4′), 155.6 (C-9), 154.9 (C-4), 150.3 (C-3′), 138.8 (C-7), 130.6 (C-5), 130.3 (C-6), 128.9 (C-6′), 124.1 (C-10), 121.5 (C-1′), 119.8 (C-8), 118.7 (C-3), 113.6 (C-5′), 112.5 (C-2′), 57.0 (C-4′-OMe), 56.5 (C-3′-OMe); HRMS (ESI-TOF) *m*/*z* [M]^+^ Calcd. For C_17_H_15_O_3_ 267.1021, found 267.1023.

#### 3.2.2. 3′,4′-Dimethoxy-6-nitroflavylium Hydrogen Sulfate (**31**)

General methodology A was applied using 2-hydroxy-5-nitrobenzaldehyde (**22**, 0.334 g, 2 mmol) and 3′,4′-dimethoxyacetophenone (**25**, 0.360 g, 2 mmol). Pure compound **31** was synthesized as a red solid (0.810 g, 99% yield). Melting point: 105–107 °C; ^1^H NMR (400 MHz, (CD_3_)_2_SO/TFA-*d*, pD ≈ 1.0; mixture of *trans*-chalcone:flavylium cation 1:0.3) Major compound: δ 8.25 (*d*, *J* = 2.8 Hz, 1H, H-5), 8.02 (*dd*, *J* = 9.0, 2.9 Hz, 1H, H-7), 7.60 (*dd*, *J* = 8.4, 2.0 Hz, 1H, H-6′), 7.40 (*d*, *J* = 2.0 Hz, 1H, H-2′), 7.08 (ov, 1H, H-4), 7.00 (ov*,* 1H, H-5′), 6.99 (ov, 1H, H-3), 6.96 (ov, 1H, H-8), 3.80 (*s*, 3H, 4′-OMe) 3.77 (*s*, 3H, 3′-OMe); ^13^C NMR (100 MHz, DMSO/TFA-*d*, pD ≈ 1.0) δ 191.7 (C-2), 161.8 (C-9), 153.5 (C-4′), 148.9 (C-3′), 139.1 (C-6), 132.7 (C-4), 130.1 (C-1′), 128.4 (C-3), 126.1 (C-5), 125.8 (C-7), 123.7 (C-6′), 123.3 (C-10), 115.6 (C-8), 110.8 (C-5′), 110.4 (C-2′), 55.7 (C-4′-OMe), 55.3 (C-3′-OMe); HRMS (ESI-TOF) *m*/*z* [M]^+^ Calcd. For C_17_H_14_ClO_3_ 301.0631, found 301.0635.

#### 3.2.3. 3′,4′-Dimethoxy-6-chloroflavylium Hydrogen Sulfate (**32**)

General methodology A was applied using 2-hydroxy-5-chlorobenzaldehyde (**23**, 0.313 g, 2 mmol) and 3′,4′-dimethoxyacetophenone (**25**, 0.360 g, 2 mmol). Pure compound **32** was synthesized as a red solid (0.790 g, 99% yield). Melting point: 190 °C (decomposes); ^1^H NMR (400 MHz, (CD_3_)_2_SO/TFA-*d*, pD ≈ 1.0; mixture of *trans*-chalcone:flavylium cation 1:0.7) Major compound: δ 7.58 (*dd*, *J* = 8.4, 2.0 Hz, 1H, H-6′), 7.40 (*d*, *J* = 2.0 Hz, 1H, H-2′), 7.26 (*d*, *J* = 2.7 Hz, 1H, H-5), 7.10 (*dd*, *J* = 8.7, 2.5 Hz, 1H, H-7), 7.04 (*d*, *J* = 8.4 Hz, 1H, H-5′), 7.07 (*d*, *J* = 6.4 Hz, 1H, H-4), 6.82 (*d*, *J* = 8.7, 1H, H-8), 6.81 (*d*, *J* = 6.4 Hz, 1H, H-3), 3.81 (*s*, 3H, 4′-Ome) 3.78 (*s*, 3H, 3′-Ome); ^13^C NMR (100 MHz, DMSO/TFA-*d*, pD ≈ 1.0) δ 191.8 (C-2), 154.5 (C-9), 153.4 (C-4′), 148.5 (C-3′), 133.3 (C-4), 130.1 (C-1′), 129.3 (C-5, C-7), 127.5 (C-3), 124.4 * (C-6), 123.6 (C-6′), 122.0 * (C-10), 117.0 (C-8), 111.1 (C-5′), 110.6 (C-2′), 55.9 (C-4′-Ome), 55.5 (C-3′-Ome); *these signals may be interchangeable. HRMS (ESI-TOF) *m/z* [M]^+^ Calcd. For C_17_H_14_ClO_3_ 301.0631, found 301.0635.

### 3.3. General Methodology B Applied to Synthesize 2,8-Dioxabicyclo[3.3.1]nonanes (***1***–***6***, ***42***–***70***)

In a round flask was added the flavylium salt species (**27**–**35**) (0.5 mmol), phloroglucinol (**36**), 4-hydroxycoumarin (**37**), 2-hydroxy-1,4-naphthoquinone (**38**), 4-hydroxy-6-phenyl-5,6-dihydro-2H-pyran-2-one (**39**), 6-(4-chlorophenyl)-4-hydroxy-5,6-dihydro-2H-pyran-2-one (**40**) or 4-hydroxy-6-(4-methoxyphenyl)-5,6-dihydro-2*H*-pyran-2-one (**41**) (0.5 mmol), and absolute MeOH (8 mL). The mixture was stirred overnight at 50 °C in an oil bath using the method previously described by Kraus et al. [52] and also used by us [35,45,46]. Finally, the solvent was removed and the crude was purified by CC using Silica gel 60 as stationary phase.

The dioxabicyclic derivatives **1** (0.069 g, 34% yield from **21**), **2** (0.170 g, 64% from **22**), **3** (0.084 g, 43% from **23**), **4** (0.032 g, 22% from **21**), **5** (0.088 g, 45% from **22**), **6** (0.077 g, 42% from **23**), **48** (0.123 g, 55% from **21**), **51** (0.129 g, 56% from **21**), and **57** (0.053 g, 23% from **21**) were previously described by us [35] and others [52,53,54] with comparable yields and their structures could be confirmed by analogy to their spectral data with those reported in the literature: **1**-**2** [35,52], **3-6** [35], **48** [53], **51** [53], and **57** [54]. Analytical HPLC (*λ* = 280 nm): compound **1** (purity: 98%; *t*_R_ = 15.9 min); compound **2** (purity: 97%; *t*_R_ = 21.2 min); compound **3** (purity: 98%; *t*_R_ = 18.8 min); compound **4** (purity: 97%; *t*_R_ = 18.0 min); compound **5** (purity: >99%; *t*_R_ = 14.9 min); compound **6** (purity: 98%; *t*_R_ = 20.7 min); compound **48** (purity 98%; *t*_R_ = 21.2 min); compound **51** (purity >99%; *t*_R_ = 19.0 min); and compound **57** (purity 97%; *t*_R_ = 21.6 min).

#### 3.3.1. 2-(3′,4′-Dimethoxyphenyl)-chromane-(4→4″, 2→O-5″)-phloroglucinol (**42**)

General methodology B was applied using the flavylium salt **30** (0.182 g) and phloroglucinol (**36**, 0.063 g, 0.5 mmol). The purification step was performed by CC using mixtures of hexane-EtOAc (75:25) as mobile phase. Pure compound **42** was obtained as a pale orange amorphous solid (0.07 g, 38% from **21**). Melting point: 77–80 °C (decomposes); Compound **42**: ^1^H NMR (400 MHz, CDCl_3_) δ 7.28 (*dd*, *J* = 7.5, 1.7 Hz, 1H, H-5), 7.15 (ov, 1H, H-5′), 7.13 (*br s*, 1H, H-2′), 6.97 (*ddd*, *J* = 8.1, 7.5, 1.7 Hz, 1H, H-7), 6.87 (*d*, *J* = 8.9 Hz, 1H, H-6′), 6.81 (*dd*, *J* = 8.1, 1.2 Hz, 1H, H-8), 6.75 (*td*, *J* = 7.5, 1.2 Hz, 1H, H-6), 5.84 (*br s*, 2H, H-2″, H-6″), 4.26 (*t*, *J* = 3.0 Hz, 1H, H-4), 3.74 (*s*, 6H, 3′-OMe, 4′-OMe), 2.29 (*d*, *J* = 3.0 Hz, 2H, H-3); ^13^C NMR (100 MHz, CDCl_3_) δ 158.2 * (C-1″ (D)), 156.3 * (C-3″ (D)), 154.6 (C-9 (A)), 153.7 (C-5″ (D)), 150.8 (C-4′ (B)), 150.2 (C-3′ (B)), 136.4 (C-1′ (B)), 129.3 (C-10 (A)), 129.0 (C-5 (A)), 128.5 (C-7(A)), 122.1 (C-6(A)), 119.7 (C-6′ (B)), 117.0 (C-8 (A)), 112.5 (C-5′ (B)), 111.0 (C-2′ (B)), 107.3 (C-4″ (D)), 100.0 (C-2 (C)), 96.9 ^#^ (C-2″ (D)), 95.8 ^#^ (C-6″ (D)), 56.6 (3′-OMe, 4′-OMe), 34.8 (C-3 (C)), 28.2 (C-4 (C)); ^*, #^ these signals may be interchangeable. HRMS (ESI-TOF) *m/z* [M+H]^+^ Calcd. For C_23_H_20_O_6_ 392.126, found 392.1265. Analytical HPLC (*λ* = 280 nm): purity: >99%; *t*_R_ = 15.9 min.

#### 3.3.2. 2-(3′,4′-Dimethoxyphenyl)-6-nitrochromane-(4→4″, 2→O-5″)-phloroglucinol (**43**)

General methodology B was applied using the flavylium salt **31** (0.205 g) and phloroglucinol (**36**, 0.063 g, 0.5 mmol). The purification step was performed by CC using mixtures of DCM-acetone (95:5) as mobile phase. Pure compound **43** was obtained as a pale yellow amorphous solid (0.116 g, 52% from **22**). Melting point: 193–195 °C; Compound **43**: ^1^H NMR (400 MHz, DMSO-*d*_6_) δ 8.20 (*d*, *J* = 2.8 Hz, 1H, H-5), 8.04 (*dd*, *J* = 9.0, 2.8 Hz, 1H, H-7), 7.03 (*d*, *J* = 8.3 Hz, 1H, H-5′), 7.27–7.22 (ov, 2H, H-2′, H-6′), 7.17 (*d*, *J* = 9.0 Hz, 1H, H-8), 5.97 (*d*, J = 2.2 Hz, 1H, H-2″), 5.89 (*d*, *J* = 2.2 Hz, H-6″), 4.44 (*br s*, 1H, H-4), 3.80 (*s*, 3H, 3′-OMe), 3.78 (*s*, 3H, 4′-OMe), 2.45–2.36 (*m*, 2H, H-3); ^13^C NMR (100 MHz, DMSO-*d*_6_) δ 158.5 (C-9 (A)), 158.0 (C-1″ (D)), 155.5 (C-3″ (D)), 152.8 (C-5″ (D)), 149.9 (C-4′ (B)), 149.1 (C-3′ (B)), 141.4 (C-6(A)), 133.4 (C-1′ (B)), 129.6 (C-10 (A)), 124.2 (C-7(A)), 123.4 (C-5 (A)), 118.6 (C-6′ (B)), 117.5 (C-8 (A)), 111.9 (C-5′ (B)), 111.1 (C-2′ (B)), 104.3 (C-4″ (D)), 100.1 (C-2 (C)), 96.8 (C-2″ (D)), 95.0 (C-6″ (D)), 56.2 (3′-OMe, 4′-OMe), 32.0 (C-3 (C)), 26.6 (C-4 (C)). HRMS (ESI-TOF) *m*/*z* [M+H]^+^ Calcd. For C_23_H_19_NO_8_ 437.1111, found 437.1108. Analytical HPLC (*λ* = 280 nm): purity: 98%; *t*_R_ = 16.7 min.

#### 3.3.3. 2-(3′,4′-Dimethoxyphenyl)-6-chlorochromane-(4→4″, 2→O-5″)-phloroglucinol (**44**)

General methodology B was applied using the flavylium salt **32** (0.199 g) and phloroglucinol (**36**, 0.063 g, 0.5 mmol). The purification step was performed by CC using mixtures of DCM-MeOH (97:3) as mobile phase. Pure compound **44** was obtained as a pale pink amorphous solid (0.135 g, 62% from **23**). Melting point: 257–259 °C; Compound **44**: ^1^H NMR (400 MHz, MeOD) δ 7.37 (*d*, *J* = 2.6 Hz, 1H, H-5), 7.26–7.23 (ov, 2H, H-2′, H-6′), 7.07 (*dd*, *J* = 8.6, 2.6 Hz, 1H, H-7), 7.00 (*d*, *J* = 8.9 Hz, 1H, H-5′), 6.90 (*d*, *J* = 8.6 Hz, 1H, H-8), 5.98-5.96 (ov, 2H, H-2″, H-6″), 4.365 (*t*, *J* = 3.0 Hz, 1H, H-4), 3.87 (*s*, 3H, 4′-OMe), 3.86 (*s*, 3H, 3′-OMe), 2.30–2.19 (*m*, 2H, H-3); ^13^C NMR (100 MHz, MeOD) δ 158.5 (C-3″ (D)), 156.3 (C-1″ (D)), 154.5 (C-5″ (D)), 152.5 (C-9 (A)), 150.9 (C-4′ (B)), 150.2 (C-3′ (B)), 136.0 (C-1′ (B)), 131.2 (C-10 (A)), 128.4 (C-5 (A)), 128.2 (C-7(A)), 126.7 (C-6(A)), 119.7 (C-6′ (B)), 118.5 (C-8 (A)), 112.4 (C-5′ (B)), 111.0 (C-2′ (B)), 106.5 (C-4″ (D)), 100.2 (C-2 (C)), 97.0 (C-2″ (D)), 95.9 (C-6″ (D)), 55.1 (3′-OMe), 55.0 (4′-OMe), 32.8 (C-3 (C)), 26.6 (C-4 (C)). HRMS (ESI-TOF) *m/z* [M+H]^+^ Calcd. For C_23_H_19_ClO_6_ 426.087, found 426.0866. Analytical HPLC (*λ* = 280 nm): purity: >99%; *t*_R_ = 17.6 min.

#### 3.3.4. 2-(3′,4′-Dihydroxyphenyl)-chromane-(4→3″, 2→O-4″)-4″-hydroxycoumarin (**45**)

General methodology B was applied using the flavylium salt **27** (0.168 g) and 4-hydroxycoumarin (**37**, 0.081g, 0.5 mmol). The purification step was performed by CC using mixtures of DCM-acetone (95:5) as mobile phase. Pure compound **45** was obtained as a pink amorphous solid (0.159 g, 72% from **21**). Melting point: 128–130 °C (decomposes); Compound **45**: ^1^H NMR (400 MHz, DMSO-*d*_6_) δ 7.79 (*dd*, *J* = 7.9, 1.6 Hz, 1H, H-5″), 7.62 (*ddd*, *J* = 8.5, 7.3, 1.6, 1H, H-7″), 7.43–7.33 (ov, 3H, H-5, H-6″, H-8″), 7.18 (ov, 2H, H-7, H-2′), 7.05 (*dd*, *J* = 8.3, 2.3 Hz, 1H, H-6′), 7.00 (*dd*, *J* = 8.2, 1.2 Hz, 1H, H-8), 6.96 (*td*, *J* = 7.4, 1.2 Hz, 1H, H-6), 6.85 (*d*, *J* = 8.3 Hz, 1H, H-5′), 4.21 (*d*, *J* = 3.0 Hz, 1H, H-4), 2.54–2.38 (*m*, 2H, H-3); ^13^C NMR (100 MHz, DMSO-*d*_6_) δ 160.5 (C-2″ (D)), 157.5 (C-4″ (D)), 151.8 (C-9″ (D)), 151.2 (C-9 (A)), 146.3 (C-4′ (B)), 145.1 (C-3′ (B)), 132.5 (C-7″ (D)), 130.3 (C-1′ (B)), 128.3 (C-7(A)), 127.8 * (C-6″ (D)), 125.6 (C-10 (A)), 124.6 (C-5 (A)), 122.4 (C-5″ (D)), 121.7 (C-6(A)), 116.6 (C-8″ (D), C-6′ (B)), 116.1 (C-8 (A)), 115.4 (C-5′ (B)), 114.4 (C-10″ (D)), 113.3 (C-2′ (B)), 105.9 (C-3″ (D)), 100.5 (C-2 (C)), 31.6 (C-3 (C)), 26.8 (C-4 (C)). HRMS (ESI-TOF) *m/z* [M+H]^+^ Calcd. For C_26_H_20_O_6_ 428.126, found 428.1261. Analytical HPLC (*λ* = 280 nm): purity: >99%; *t*_R_ = 17.2 min.

#### 3.3.5. 2-(3′,4′-Dihydroxyphenyl)-6-nitrochromane-(4→3″, 2→O-4″)-4″-hydroxycoumarin (**46**)

General methodology B was applied using the flavylium salt **28** (0.191 g) and 4-hydroxycoumarin (**37**, 0.081g, 0.5 mmol). The purification step was performed by CC using mixtures of DCM-acetone (95:5). Pure compound **46** was obtained as a pale grey amorphous solid (0.228 g, 79% from **22**). Melting point: 128–130 °C (decomposes); Compound **46**: ^1^H NMR (400 MHz, MeOD) δ 8.23 (*d*, *J* = 2.8 Hz, 1H, H-5), 7.99 (*dd*, *J* = 9.0, 2.8 Hz 1H, H-7), 7.74 (*dd*, *J* = 8.5, 1.6 Hz, 1H, H-5″), 7.50 (*ddd*, J = 8.2, 7.5, 1.6, 1H, H-7″), 7.26–7.20 (ov, 2H, H-6″, H-8″), 7.11 (*d*, *J* = 2.3 Hz, 1H, H-2′), 7.05 (*d*, *J* = 9.0 Hz, 1H, H-8), 7.00 (*dd*, *J* = 8.3, 2.3 Hz, 1H, H-6′), 6.78 (*d*, *J* = 8.3 Hz, 1H, H-5′), 4.30 (*t*, *J* = 3.0 Hz, 1H, H-4), 2.50 (*dd*, *J* = 14.0, 3.0 Hz, 1H, H-3), 2.39 (*dd*, *J* = 14.0, 3.0 Hz, 1H, H-3); ^13^C NMR (100 MHz, MeOD) δ 163.3 (C-2″ (D)), 159.9 (C-4″ (D)), 158.5 (C-9 (A)), 153.9 (C-9″ (D)), 148.0 (C-4′ (B)), 146.7 (C-3′ (B)), 143.6 (C-6(A)), 134.0 (C-7″ (D)), 131.6 (C-1′ (B)), 128.3 (C-10 (A)), 125.9 (C-6″ (D)), 125.5 (C-7(A)), 124.8 (C-5 (A)), 123.9 (C-5″ (D)), 118.5 (C-6′ (B)), 118.3 (C-8 (A)), 117.9 (C-8″ (D)), 116.4 (C-5′ (B)), 116.1 (C-10″ (D)), 114.2 (C-2′ (B)), 106.4 (C-3″ (D)), 102.6 (C-2 (C)), 33.0 (C-3 (C)), 28.8 (C-4 (C)). HRMS (ESI-TOF) *m/z* [M+H]^+^ Calcd. For C_24_H_15_NO_8_ 445.0798, found 445.0795. Analytical HPLC (*λ* = 280 nm): purity: 99%; *t*_R_ = 17.8 min.

#### 3.3.6. 2-(3′,4′-Dihydroxyphenyl)-6-chlorochromane-(4→3″, 2→O-4″)-4″-hydroxycoumarin (**47**)

General methodology B was applied using the flavylium salt **29** (0.185 g) and 4-hydroxycoumarin (**37**, 0.081g, 0.5 mmol). The purification step was performed by CC using mixtures of DCM-acetone (95:5). Pure compound **47** was obtained as a pale pink amorphous solid (0.225 g, 81% from **23**). Melting point: 98–100 °C (decomposes); Compound **47**: ^1^H NMR (400 MHz, DMSO-*d*_6_) δ 7.81 (*dd*, *J* = 7.9, 1.6 Hz, 1H, H-5″), 7.65 (*ddd*, J = 8.7, 7.3, 1.6, 1H, H-7″), 7.44–7.37 (ov, 2H, H-6″, H-8″), 7.36 (*d*, *J* = 2.7 Hz, 1H, H-5), 7.24 (*dd*, *J* = 8.7, 2.6 Hz 1H, H-7), 7.17 (*d*, *J* = 2.3 Hz, 1H, H-2′), 7.05 (ov, 2H, H-8, H-6′), 6.85 (*d*, *J* = 8.3 Hz, 1H, H-5′), 4.23 (br *t*, *J* = 3.0 Hz, 1H, H-4), 2.55 (*dd*, *J* = 13.9, 3.2 Hz, 1H, H-3), 2.44 (*dd*, *J* = 13.9, 2.9 Hz, 1H, H-3); ^13^C NMR (100 MHz, DMSO-*d*_6_) δ 160.5 (C-2″ (D)), 157.5 (C-4″ (D)), 151.8 (C-9″ (D)), 150.2 (C-9 (A)), 146.4 (C-4′ (B)), 145.1 (C-3′ (B)), 132.7 (C-7″ (D)), 129.9 (C-1′ (B)), 128.0 (C-7(A)), 127.6 (C-10 (A)), 127.1 (C-5 (A)), 125.1 (C-6(A)), 124.6 (C-8″ (D)), 122.4 (C-5″ (D)), 117.9 (C-8 (A)), 116.8 (C-6′ (B)), 116.6 (C-6″ (D)), 115.4 (C-5′ (B)), 114.3 (C-10″ (D)), 113.2 (C-2′ (B)), 105.3 (C-3″ (D)), 100.6 (C-2 (C)), 30.7 (C-3 (C)), 26.7 (C-4 (C)). HRMS (ESI-TOF) *m/z* [M+H]^+^ Calcd. For C_24_H_15_ClO_6_ 434.0557, found 434.0557. Analytical HPLC (*λ* = 280 nm): purity: 98%; *t*_R_ = 19.0 min.

#### 3.3.7. 2-(3′,4′-Dimethoxyphenyl)-6-nitrochromane-(4→3″, 2→O-4″)-4″-hydroxycoumarin (**49**)

General methodology B was applied using the flavylium salt **31** (0.205 g) and 4-hydroxycoumarin (**37**, 0.081g, 0.5 mmol). The purification step was performed by CC using mixtures of DCM-acetone (97:3). Pure compound **49** was obtained as a white amorphous solid (0.126 g, 54% from **22**). Melting point: 142–144 °C; Compound **49**: ^1^H NMR (400 MHz, MeOD) δ 8.43 (*d*, *J* = 2.7 Hz, 1H, H-5), 8.08 (*dd*, *J* = 9.0, 2.7 Hz 1H, H-7), 7.84 (*dd*, *J* = 7.9, 1.6 Hz, 1H, H-5″), 7.54 (*ddd*, J = 8.7, 7.3, 1.6, 1H, H-7″), 7.33-7.26 (ov, 3H, H-6′, H-6″, H-8″), 7.21 (*d*, *J* = 2.2 Hz, 1H, H-2′), 7.11 (*d*, *J* = 9.0 Hz, 1H, H-8), 6.98 (*d*, *J* = 8.5 Hz, 1H, H-5′), 4.48 (*t*, *J* = 3.0 Hz, 1H, H-4), 3.95 (*s*, 3H, 3′-OMe), 3.94 (*s*, 3H, 4′-OMe), 2.51 (*qd*, *J* = 13.8, 3.0 Hz, 2H, H-3); ^13^C NMR (100 MHz, MeOD) δ 161.3 (C-2″ (D)), 158.3 (C-4″ (D)), 156.9 (C-9 (A)), 152.8 (C-9″ (D)), 152.8 (C-4′ (B)), 150.5 (C-3′ (B)), 142.6 (C-6(A)), 132.8 (C-7″ (D)), 131.2 (C-1′ (B)), 126.4 (C-10 (A)), 124.7 * (C-8″ (D)), 124.6 (C-7(A)), 124.3 (C-5 (A)), 122.9 (C-5″ (D)), 118.4 * (C-6′ (B)), 117.3 (C-8 (A)), 117.3 * (C-6″ (D)), 114.9 (C-10″ (D)), 111.1 (C-5′ (B)), 109.0 (C-2′ (B)), 105.3 (C-3″ (D)), 100.8 (C-2 (C)), 56.4 (3′-OMe), 56.3 (4′-OMe), 32.4 (C-3 (C)), 27.4 (C-4 (C)). HRMS (ESI-TOF) *m/z* [M+H]^+^ Calcd. For C_26_H_19_NO_8_ 473.1110, found 473.1111. Analytical HPLC (*λ* = 280 nm): purity: 97%; *t*_R_ = 21.3 min.

#### 3.3.8. 2-(3′,4′-Dimethoxyphenyl)-6-chlorochromane-(4→3″, 2→O-4″)-4″-hydroxycoumarin (**50**)

General methodology B was applied using the flavylium salt **32** (0.199 g) and 4-hydroxycoumarin (**37**, 0.081g, 0.5 mmol). The purification step was performed by CC using mixtures of DCM-MeOH (98:2). Pure compound **50** was obtained as a white amorphous solid (0.168 g, 81% from **23**). Melting point: 130–133 °C; Compound **50**: ^1^H NMR (400 MHz, CDCl_3_) δ 7.84 (*dd*, *J* = 7.9, 1.3 Hz, 1H, H-5″), 7.53–7.49 (ov, 2H, H-5, H-8″), 7.28–7.25 (ov, 3H, H-6′, H-6″, 7″), 7.21 (*d*, *J* = 2.2 Hz, 1H, H-2′), 7.13 (*dd*, *J* = 8.7, 2.5 Hz 1H, H-7), 6.96 (*d*, *J* = 8.5 Hz, 2H, H-8, H-5′), 4.33 (*t*, *J* = 3.0 Hz, 1H, H-4), 3.93 (s, 6H, 3′-OMe, 4′-OMe), 2.42 (*d*, *J* = 3.0 Hz, 2H, H-3); ^13^C NMR (100 MHz, CDCl_3_) δ 161.6 (C-2″ (D)), 158.6 (C-9″ (D)), 152.7 (C-4″ (D)), 150.2 (C-9 (A), C-4′ (B)), 149.2 (C-3′ (B)), 124.3 (C-7″ (D)), 132.6 (C-8″ (D)), 132.0 * (C-1′ (B)), 128.6 (C-7(A)), 128.1 (C-5 (A)), 127.2 * (C-6(A)), 126.9 * (C-10 (A)), 122.9 (C-5″ (D)), 118.4 (C-6′ (B)), 117.8 (C-8 (A)), 117.2 (C-6″ (D)), 115.7 (C-3″ (D)), 115.1 (C-10″ (D)), 111.1 (C-5′ (B)), 109.1 (C-2′ (B)), 100.5 (C-2 (C)), 56.3 (3′-OMe), 56.2 (4′-OMe), 32.8 (C-3 (C)), 27.4 (C-4 (C)). HRMS (ESI-TOF) *m/z* [M+H]^+^ Calcd. For C_26_H_19_ClO_6_ 462.087, found 462.0868. Analytical HPLC (*λ* = 280 nm): purity: >99%; *t*_R_ = 22.9 min.

#### 3.3.9. 2-(4′-Hydroxyphenyl)-6-nitrochromane-(4→3″, 2→O-4″)-4″-hydroxycoumarin (**52**)

General methodology B was applied using the flavylium salt **34** (0.183 g) and 4-hydroxycoumarin (**37**, 0.081g, 0.5 mmol). The purification step was performed by CC using mixtures of DCM-acetone (95:5). Pure compound **52** was obtained as a white amorphous solid (0.158 g, 58% from **22**). Melting point: 275–278 °C; Compound **52**: ^1^H NMR (400 MHz, DMSO-*d*_6_) δ 8.23 (*d*, *J* = 2.9 Hz, 1H, H-5), 8.10 (*dd*, *J* = 9.0, 2.9 Hz 1H, H-7), 7.84 (*dd*, *J* = 8.0, 1.7 Hz, 1H, H-5″), 7.68–7.63 (ov, 3H, H-2′, H-6′, H-7″), 7.43 (*dd*, *J* = 8.5, 1.1 Hz, 1H, H-8″), 7.39 (*td*, J = 7.6, 1.1 Hz, 1H, H-6″), 7.26 (*d*, *J* = 9.0 Hz, 1H, H-8), 6.95-6.89 (*m*, 2H, H-3′, H-5′), 4.42 (*t*, *J* = 3.0 Hz, 1H, H-4), 2.67 (*dd*, *J* = 13.9, 3.0 Hz, 1H, H-3), 2.58 (*dd*, *J* = 13.9, 3.0 Hz, 1H, H-3); ^13^C NMR (100 MHz, DMSO-*d*_6_) δ 160.4 (C-2″ (D)), 158.6 (C-4′ (B)), 157.3 (C-4″ (D)), 156.9 (C-9 (A)), 151.9 (C-9″ (D)), 141.4 (C-6(A)), 132.8 (C-7″ (D)), 128.1 (C-1′ (B)), 127.2 (C-2′ (B), C-6′ (B)), 126.7 (C-10 (A)), 124.7 (C-6″ (D)), 124.4 (C-7(A)), 123.3 (C-5 (A)), 122.6 (C-5″ (D)), 117.3 (C-8 (A)), 116.7 (C-8″ (D)), 115.2 (C-3′ (B), C-5′ (B)), 114.2 (C-10″ (D)), 105.3 (C-3″ (D)), 101.1 (C-2 (C)), 30.3 (C-3 (C)), 26.6 (C-4 (C)). HRMS (ESI-TOF) *m/z* [M+H]^+^ Calcd. For C_24_H_15_ClO_6_ 429.0849, found 429.0847. Analytical HPLC (*λ* = 280 nm): purity: 98%; *t*_R_ = 19.3 min.

#### 3.3.10. 2-(4′-Hydroxyphenyl)-6-chlorochromane-(4→3″, 2→O-4″)-4″-hydroxycoumarin (**53**)

General methodology B was applied using the flavylium salt **35** (0.177 g) and 4-hydroxycoumarin (**37**, 0.081g, 0.5 mmol). The purification step was performed by CC using mixtures of DCM-acetone (95:5). Pure compound **53** was obtained as a white amorphous solid (0.190 g, 57% from **23**). Melting point: 250–252 °C (decomposes); Compound **53**: ^1^H NMR (400 MHz, DMSO-*d*_6_) δ 7.81 (*dd*, *J* = 7.9, 1.6 Hz, 1H, H-5″), 7.67–7.59 (ov, 3H, H-2′, H-6′, H-7″), 7.42 (*dd*, *J* = 8.4, 1.0 Hz, 1H, H-8″), 7.40–7.35 (ov, 2H, H-5, H-6″), 7.24 (*dd*, *J* = 8.7, 2.6 Hz 1H, H-7), 7.06 (*d*, *J* = 8.7 Hz, 1H, H-8), 6.92–6.87 (*m*, 2H, H-3′, H-5′), 4.25 (*t*, *J* = 3.0 Hz, 1H, H-4), 2.59 (*dd*, *J* = 13.8, 3.2 Hz, 1H, H-3), 2.49–2.42 (ov, 1H, H-3); ^13^C NMR (100 MHz, DMSO-*d*_6_) δ 160.5 (C-2″ (D)), 158.4 (C-4′ (B)), 157.6 (C-4″ (D)), 151.8 (C-9″ (D)), 150.2 (C-9 (A)), 132.7 (C-7″ (D)), 129.4 (C-1′ (B)), 128.0 (C-7(A)), 127.6 (C-10 (A)), 127.1 (C-2′ (B), C-6′ (B)), 127.0 (C-5 (A)), 125.1 (C-6(A)), 124.6 (C-6″ (D)), 122.5 (C-5″ (D)), 118.0 (C-8 (A)), 116.6 (C-8″ (D)), 115.1 (C-3′ (B), C-5′ (B)), 114.3 (C-10″ (D)), 105.2 (C-3″ (D)), 100.7 (C-2 (C)), 30.8 (C-3 (C)), 26.7 (C-4 (C)). HRMS (ESI-TOF) *m/z* [M+H]^+^ Calcd. For C_24_H_15_ClO_6_ 418.0608, found 418.0606. Analytical HPLC (*λ* = 280 nm): purity: 98%; *t*_R_ = 20.7 min.

#### 3.3.11. 2-(3′,4′-Dihydroxyphenyl)-chromane-(4→3″, 2→O-2″)-2″-hydroxy-1″,4″-naphthoquinone (**54**)

General methodology B was applied using the flavylium salt **27** (0.168 g) and 2-hydroxy-1,4-naphthoquinone (**38**, 0.087g, 0.5 mmol). The purification step was performed by CC using mixtures of DCM-acetone (90:10). Pure compound **54** was obtained as a yellow amorphous solid (0.023 g, 10% from **21**). Melting point: 190–192 °C (decomposes); Compound **54**: ^1^H NMR (400 MHz, DMSO-*d*_6_) δ 8.01–7.94 (*m*, 2H, H-5″, H-8″), 7.86–7.76 (*m*, 2H, H-6″, H-7″), 7.35 (*dd*, *J* = 7.6, 1.7 Hz, 1H, H-5), 7.02–6.99 (*m*, 2H, H-8, H-6′), 7.14 (*d*, *J* = 2.3 Hz, 1H, H-2′), 7.18 (*ddd*, *J* = 8.2, 7.4, 1.7 Hz, 1H, H-7), 6.95 *(td*, *J* = 7.4, 1.2 Hz, 1H, H-6), 6.83 (*d*, *J* = 8.3 Hz, 1H, H-5′), 4.41 (*t*, *J* = 3.0 Hz, 1H, H-4), 2.43–2.34 (*m*, 2H, H-3); ^13^C NMR (100 MHz, DMSO-*d*_6_) δ 182.2 (C-1″ (D)), 178.5 (C-4″ (D)), 152.8 (C-3″ (D)), 151.6 (C-9 (A)), 146.3 (C-4′ (B)), 145.0 (C-3′ (B)), 134.5 ^#^ (C-7″ (D)), 133.9 ^#^ (C-6″ (D)), 131.1^$^ (C-9″ (D)), 130.6^$^ (C-10″ (D)), 130.3 (C-1′ (B)), 128.3 (C-5 (A), C-7 (A)), 126.0 * (C-8″ (D)), 125.8 * (C-5″ (D)), 124.5 (C-2″ (D)), 124.8 (C-10 (A)), 121.6 (C-6(A)), 116.8 (C-6′ (B)), 116.2 (C-8 (A)), 115.2 (C-5′ (B)), 113.4 (C-2′ (B)), 100.1 (C-2 (C)), 30.6 (C-3 (C)), 25.7 (C-4 (C)). HRMS (ESI-TOF) *m/z* [M+H]^+^ Calcd. For C_25_H_16_O_6_ 412.0947, found 412.0942. Analytical HPLC (*λ* = 280 nm): purity: 97%; *t*_R_ = 18.6 min.

#### 3.3.12. 2-(3′.4′-Dihydroxyphenyl)-6-nitrochromane-(4→3″, 2→O-2″)-2″-hydroxy-1″,4″-naphthoquinone (**55**)

General methodology B was applied using the flavylium salt **28** (0.191 g) and 2-hydroxy-1,4-naphthoquinone (**38**, 0.087g, 0.5 mmol). The purification step was performed by CC using mixtures of DCM-acetone (95:5). Pure compound **55** was obtained as a yellow amorphous solid (0.140 g, 47% from **22**). Melting point: 247–250 °C; Compound **55**: ^1^H NMR (400 MHz, DMSO-*d*_6_) δ 8.21 (*d*, *J* = 2.8 Hz, 1H, H-5), 8.08 (*dd*, *J* = 9.0, 2.8 Hz 1H, H-7), 8.02–7.96 (*m*, 2H, H-5″, H-8″), 7.86–7.78 (*m*, 2H, H-6″, H-7″), 7.24 *(d*, *J* = 9.0 Hz, 1H, H-8), 7.17 (*d*, *J* = 2.3 Hz, 1H, H-2′), 7.03 (*dd*, *J* = 8.3, 2.2 Hz 1H, H-6′), 6.85 (*d*, *J* = 8.2 Hz, 1H, H-5′), 4.58 (*t*, *J* = 2.9 Hz, 1H, H-4), 2.54-2.46 (*m*, 2H, H-3); ^13^C NMR (100 MHz, CDCl_3_) δ 182.2 (C-1″ (D)), 178.2 (C-4″ (D)), 157.3 (C-9 (A)), 152.5 (C-3″ (D)), 146.6 (C-4′ (B)), 145.1 (C-3′ (B)), 141.3 (C-6(A)), 134.5 ^#^ (C-6″ (D)), 134.1 ^#^ (C-7″ (D)), 131.1 (C-10″ (D)), 130.7 (C-9″ (D)), 129.2 (C-1′ (B)), 126.4 (C-2″ (D)), 126.0 * (C-10 (A), C-8″ (D)), 125.8 * (C-5″ (D)) 124.3 (C-7(A)), 123.9 (C-5 (A)), 117.3 (C-8 (A)), 116.8 (C-6′ (B)), 115.3 (C-5′ (B)), 113.4 (C-2′ (B)), 100.6 (C-2 (C)), 29.7 (C-3 (C)), 25.6 (C-4 (C)). HRMS (ESI-TOF) *m/z* [M+H]^+^ Calcd. For C_25_H_15_NO_8_ 457.0798, found 457.0803. Analytical HPLC (*λ* = 280 nm): purity: 98%; *t*_R_ = 18.2 min.

#### 3.3.13. 2-(3′,4′-Dihydroxyphenyl)-6-chlorochromane-(4→3″, 2→O-2″)-2″-hydroxy-1″,4″-naphthoquinone (**56**)

General methodology B was applied using the flavylium salt **29** (0.185 g) and 2-hydroxy-1,4-naphthoquinone (**38**, 0.087g, 0.5 mmol). The purification step was performed by CC using mixtures of DCM-acetone (90:10). Pure compound **56** was obtained as a yellow amorphous solid (0.052 g, 19% from **23**). Melting point: 300–302 °C (decomposes); Compound **56**: ^1^H NMR (400 MHz, DMSO-*d*_6_) δ 8.03-7.95 (*m*, 2H, H-5″, H-8″), 7.87–7.77 (*m*, 2H, H-6″, H-7″), 7.35–7.33 (*m*, 1H, H-5), 7.22 (*dd*, *J* = 8.7, 2.6 Hz 1H, H-7), 7.13 (*d*, *J* = 2.3 Hz, 1H, H-2′), 7.04 *(d*, *J* = 8.7 Hz, 1H, H-8), 7.00 (*dd*, *J* = 8.3, 2.3 Hz 1H, H-6′), 6.85–6.81 (*m*, 1H, H-5′), 4.41 (*t*, *J* = 3.1 Hz, 1H, H-4), 2.40 (ov, 1H, H-3); ^13^C NMR (100 MHz, DMSO-*d*_6_) δ 182.2 (C-1″ (D)), 178.3 (C-4″ (D)), 152.9 (C-3″ (D)), 150.6 (C-9 (A)), 146.4 (C-4′ (B)), 145.1 (C-3′ (B)), 134.5 ^#^ (C-7″ (D)), 133.9 ^#^ (C-6″ (D)), 131.1^$^ (C-9″ (D)), 130.7^$^ (C-10″ (D)), 129.9 (C-1′ (B)), 128.0 (C-7(A)), 127.6 (C-5 (A)), 126.8 (C-10 (A)), 126.0 * (C-8″ (D)), 125.8 * (C-5″ (D)), 125.0 (C-6(A)), 123.8 (C-2″ (D)), 118.0 (C-8 (A)), 116.8 (C-6′ (B)), 115.2 (C-5′ (B)), 113.4 (C-2′ (B)), 100.2 (C-2 (C)), 30.1 (C-3 (C)), 25.7 (C-4 (C)). HRMS (ESI-TOF) *m/z* [M+H]^+^ Calcd. For C_25_H_15_ClO_6_ 446.0557, found 446.0557. Analytical HPLC (*λ* = 280 nm): purity: 95%; *t*_R_ = 19.7 min.

#### 3.3.14. 2-(3′.4′-Dimethoxyphenyl)-6-nitrochromane-(4→3″, 2→O-2″)-2″-hydroxy-1″,4″-naphthoquinone (**58**)

General methodology B was applied using the flavylium salt **31** (0.205 g) and 2-hydroxy-1,4-naphthoquinone (**38**, 0.087g, 0.5 mmol). The purification step was performed by CC using mixtures of DCM-acetone (98:2). Pure compound **58** was obtained as a yellow amorphous solid (0.100 g, 42% from **22**). Melting point: 148–150 °C; Compound **58**: ^1^H NMR (400 MHz, CDCl_3_) δ 8.38 (*d*, *J* = 2.7 Hz, 1H, H-5), 8.13-8.04 (ov, 3H, H-7, H-5″, H-8″), 7.77–7.64 (*m*, 2H, H-6″, H-7″), 7.27 (*dd*, *J* = 8.3, 2.1 Hz 1H, H-6′), 7.24 (*d*, *J* = 2.1 Hz, 1H, H-2′), 7.10 *(d*, *J* = 9.0 Hz, 1H, H-8), 6.94 (*d*, *J* = 8.3 Hz, 1H, H-5′), 4.63 (*t*, *J* = 2.7 Hz, 1H, H-4), 3.94 (*s*, 3H, 3′-OMe), 3.92 (*s*, 3H, 3′-OMe), 2.49 (*dd*, *J* = 13.8, 3.0 Hz, 1H, H-3), 2.40 (*dd*, *J* = 13.8, 3.0 Hz, 1H, H-3); ^13^C NMR (100 MHz, CDCl_3_) δ 182.5 (C-1″ (D)), 178.7 (C-4″ (D)), 157.4 (C-9 (A)), 153.0 (C-3″ (D)), 150.4 (C-4′ (B)), 149.2 (C-3′ (B)), 142.5 (C-6(A)), 134.6 ^#^ (C-6″ (D)), 134.0 ^#^ (C-7″ (D)), 131.7 (C-10″ (D)), 131.4 (C-1′ (B)), 130.8 (C-9″ (D)), 126.8 * (C-5″ (D)), 126.7 * (C-8″ (D)), 125.4 (C-10 (A)), 123.4 (C-2″ (D)), 124.7 (C-5 (A), C-7 (A)), 118.6 (C-6′ (B)), 117.4 (C-8 (A)), 111.1 (C-5′ (B)), 109.1 (C-2′ (B)), 100.5 (C-2 (C)), 56.3 (3′-OMe), 56.2 (4′-OMe), 31.5 (C-3 (C)), 26.3 (C-4 (C)); ^*, #^these signals may be interchangeable. HRMS (ESI-TOF) *m/z* [M+H]^+^ Calcd. For C_27_H_19_NO_8_ 485.1111, found 485.1112. Analytical HPLC (*λ* = 280 nm): purity: >99%; *t*_R_ = 21.3 min.

#### 3.3.15. 2-(3′,4′-Dimethoxyphenyl)-6-chlorochromane-(4→3″, 2→O-2″)-2″-hydroxy-1″,4″-naphthoquinone (**59**)

General methodology B was applied using the flavylium salt **32** (0.199 g) and 2-hydroxy-1,4-naphthoquinone (**38**, 0.087g, 0.5 mmol). The purification step was performed by CC using mixtures of DCM-MeOH (98:2). Pure compound **59** was obtained as a yellow amorphous solid (0.150 g, 67% from **23**). Melting point: 124–127 °C.; Compound **59**: ^1^H NMR (400 MHz, DMSO-*d*_6_) δ 8.06-7.99 (*m*, 2H, H-5″, H-8″), 7.71–7.64 (*m*, 2H, H-6″, H-7″), 7.42 (*d*, *J* = 2.6 Hz, 1H, H-5), 7.29 (*dd*, *J* = 8.4, 2.1 Hz 1H, H-6′), 7.23 (*d*, *J* = 2.1 Hz, 1H, H-2′), 7.10 (*dd*, *J* = 8.7, 2.6 Hz 1H, H-7), 6.94 *(d*, *J* = 8.7 Hz, 1H, H-8), 6.91 (*d*, *J* = 8.4 Hz, 1H, H-5′), 4.48 (*t*, *J* = 3.0 Hz, 1H, H-4), 3.92 (*s*, 3H, 3′-OMe), 3.90 (*s*, 3H, 4′-OMe), 2.43 (*dd*, *J* = 13.7, 3.0 Hz, 1H, H-3), 2.29 (*dd*, *J* = 13.7, 3.0 Hz, 1H, H-3); ^13^C NMR (100 MHz, CDCl_3_) δ 182.4 (C-1″ (D)), 178.6 (C-4″ (D)), 153.2 (C-3″ (D)), 150.5 (C-9 (A)), 149.8 (C-4′ (B)), 148.9 (C-3′ (B)), 134.2 (C-6″ (D)), 133.5 (C-7″ (D)), 131.5 (C-10″ (D)), 131.4 (C-1′ (B)), 130.9 (C-9″ (D)), 128.3 (C-7(A)), 128.0 (C-5 (A)), 126.7 (C-2″ (D)), 126.5 * (C-8″ (D)), 126.3 * (C-5″ (D)), 125.8 (C-6(A)), 124.1 (C-10 (A)), 118.3 (C-6′ (B)), 117.9 (C-8 (A)), 110.8 (C-5′ (B)), 108.9 (C-2′ (B)), 100.0 (C-2 (C)), 56.0 (3′-OMe, 4′-OMe)31.6 (C-3 (C)), 26.1 (C-4 (C)). HRMS (ESI-TOF) *m/z* [M+H]^+^ Calcd. For C_27_H_19_ClO_6_ 474.087, found 474.087. Analytical HPLC (*λ* = 280 nm): purity: 98%; *t*_R_ = 23.2 min.

#### 3.3.16. 2-(4′-Hydroxyphenyl)-6-nitrochromane-(4→3″, 2→O-2″)-2″-hydroxy-1″,4″-naphthoquinone (**60**)

General methodology B was applied using the flavylium salt **34** (0.183 g) and 2-hydroxy-1,4-naphthoquinone (**38**, 0.087 g, 0.5 mmol). Compound **60** was recovered by filtration as a yellow amorphous solid (0.134 g, 46% from **22**). Melting point: 270–273 °C (decomposes); Compound **60**: ^1^H NMR (400 MHz, DMSO-*d*_6_) δ 8.21 (*d*, *J* = 2.8 Hz, 1H, H-5), 8.08 (*dd*, *J* = 9.0, 2.9 Hz 1H, H-7), 8.00–7.95 (*m*, 2H, H-5″, H-8″), 7.86–7.79 (*m*, 2H, H-6″, H-7″), 7.59 (*d*, *J* = 8.3 Hz, 2H, H-2′, H-6′), 7.25 *(d*, *J* = 9.0 Hz, 1H, H-8), 6.89 (*d*, *J* = 8.3 Hz, 2H, H-3′, H-5′), 4.60 (*t*, *J* = 3.0 Hz, 1H, H-4), 2.53-2.49 (*m*, 2H, H-3); ^13^C NMR (100 MHz, CDCl_3_) δ 182.2 (C-1″ (D)), 178.0 (C-4″ (D)), 158.5 (C-4′ (B)), 157.3 (C-9 (A)), 152.6 (C-3″ (D)), 141.3 (C-6(A)), 134.6 ^#^ (C-7″ (D)), 134.0 ^#^ (C-6″ (D)), 131.1 * (C-9″ (D)), 130.7 * (C-10″ (D)), 128.8 (C-1′ (B)), 127.2 (C-2′ (B), C-6′ (B)), 126.1 (C-5″ (D)), 126.0 (C-10 (A)), 125.9 (C-8″ (D)), 124.3 (C-7(A)), 123.9 (C-5 (A)), 123.4 (C-2″ (D)), 117.3 (C-8 (A)), 115.1 (C-3′ (B), C-5′ (B)), 100.6 (C-2 (C)), 29.5 (C-3 (C)), 25.6 (C-4 (C)); *these signals may be interchangeable. HRMS (ESI-TOF) *m/z* [M+H]^+^ Calcd. For C_25_H_15_NO_7_ 441.0849, found 441.0855. Analytical HPLC (*λ* = 280 nm): purity: >99%; *t*_R_ = 19.6 min.

#### 3.3.17. 2-(4′-Hydroxyphenyl)-6-chlorochromane-(4→3″, 2→O-2″)-2″-hydroxy-1″,4″-naphthoquinone (**61**)

General methodology B was applied using the flavylium salt **35** (0.177 g) and 2-hydroxy-1,4-naphthoquinone (**38**, 0.087g, 0.5 mmol). The purification step was performed by CC using mixtures of DCM-acetone (98:2). Pure compound **61** was obtained as a red-orange amorphous solid (0.050 g, 15% from **23**). Melting point: 249–251 °C; Compound **61**: ^1^H NMR (400 MHz, DMSO-*d*_6_) δ 8.03-7.96 (*m*, 2H, H-5″, H-8″), 7.89–7.78 (*m*, 2H, H-6″, H-7″), 7.59–7.53 (*m*, 2H, H-2′, H-6′), 7.34 (*d*, *J* = 2.6 Hz, 1H, H-5), 7.23 (*dd*, *J* = 8.7, 2.6 Hz 1H, H-7), 7.06 *(d*, *J* = 8.7 Hz, 1H, H-8), 6.92-6.85 (*m*, 2H, H-3′, H-5′), 4.45-4.42 (*m*, 1H, H-4), 2.48-2.39 (*m*, 2H, H-3); ^13^C NMR (100 MHz, CDCl_3_) δ 182.2 (C-1″ (D)), 178.3 (C-4″ (D)), 158.3 (C-4′ (B)), 152.9 (C-3″ (D)), 150.0 (C-9 (A)), 134.5 ^#^ (C-7″ (D)), 133.9 ^#^ (C-6″ (D)), 131.1 * (C-9″ (D)), 130.7 * (C-10″ (D)), 129.4 (C-1′ (B)), 128.0 (C-7(A)), 127.6 (C-5 (A)), 127.1 (C-2′ (B), C-6′ (B)), 126.7 (C-10 (A)), 126.0 (C-5″ (D)), 125.8 (C-8″ (D)), 125.0 (C-6(A)), 123.7 (C-2″ (D)), 118.1 (C-8 (A)), 115.2 (C-3′ (B), C-5′ (B)), 100.3 (C-2 (C)), 30.0 (C-3 (C)), 25.6 (C-4 (C)); *these signals may be interchangeable. HRMS (ESI-TOF) *m/z* [M+H]^+^ Calcd. For C_25_H_15_ClO_5_ 430.0608, found 430.0615. Analytical HPLC (*λ* = 280 nm): purity: 99%; *t*_R_ = 21.3 min.

#### 3.3.18. 2-(3′,4′-Dimethoxyphenyl)-chromane-(4→3″, 2→O-4″)-4″-hydroxy-6″-phenyl-5″,6″-dihydro-2H-pyran-2″-one (**62a** and **62b**)

General methodology B was applied using the flavylium salt **30** (0.182 g) and 4-hydroxy-6-phenyl-5,6-dihydro-2*H*-pyran-2-one (**39**, 0.095 g, 0.5 mmol), previously prepared according to the methodology described by Andersh et al. [48,49]. The purification step was performed by CC using mixtures of hexane-EtOAc (75:25). Pure compound **62a** was obtained as a pale yellow amorphous solid (0.011 g, 5.0% from **21**) and pure compound **62b** as a white amorphous solid (0.019 g, 9.0% from **21**); Compound **62a**: ^1^H NMR (400 MHz, CDCl_3_) δ 7.38–7.24 (*m*, 5H, H-5, H-2‴, H-3‴), 7.23–7.16 (ov, 3H, H-7, H-6′, H-4‴), 7.13 (*d*, *J* = 2.2 Hz, 1H, H-2′), 7.06 (*dd*, *J* = 8.1, 1.2 Hz, 1H, H-8), 6.97 (*td*, *J* = 7.4, 1.2 Hz, 1H, H-6), 6.90 (*d*, *J* = 8.5 Hz, 1H, H-5′), 5.31 (*dd*, *J* = 12.3, 4.0 Hz, 1H, H-6″), 4.30 (*t*, *J* = 3.0 Hz, 1H, H-4), 3.90 (*s*, 6H, 3′-OMe, 4′-OMe), 3.03-2.96 (*m*, 1H, H-5″), 2.70 (*dd*, *J* = 17.2, 4.0 Hz, 1H, H-5″), 2.35-2.22 (*m*, 2H, H-3); ^13^C NMR (100 MHz, CDCl_3_) δ 165.8 (C-2″ (D)), 163.3 (C-4″ (D)), 151.6 (C-9 (A)), 150.0 (C-4′ (B)), 148.0 (C-3′ (B)), 138.4 (C-1‴ (E)), 132.2 (C-1′ (B)), 128.9 (C-5 (A), C-3‴ (E)), 128.3 * (C-7(A)), 128.1 * (C-4‴ (E)), 126.6 (C-2‴ (E)), 126.4 (C-10 (A)), 122.2 (C-6(A)), 118.3 (C-6′ (B)), 116.5 (C-8 (A)), 110.9 (C-5′ (B)), 109.1 (C-2′ (B)), 106.6 (C-3″ (D)), 100.6 (C-2 (C)), 76.6 (C-6″ (D)), 56.0 (3′-OMe, 4′-OMe), 34.3 (C-5″ (D)), 33.2 (C-3 (C)), 26.5 (C-4 (C)); *these signals could be interchangeable. Melting point: 94–96 °C. HRMS (ESI-TOF) *m/z* [M+H]^+^ Calcd. For C_28_H_24_O_6_ 456.1573, found 456.1571. Analytical HPLC (*λ* = 280 nm): purity: 96%; *t*_R_ = 20.7 min. Compound **62b**: ^1^H NMR (400 MHz, CDCl_3_) δ 7.56 (*dd*, *J* = 7.5, 1.6 Hz, 1H, H-5), 7.35–7.30 (*m*, 5H, H-2‴, H-3‴, H-4‴), 7.21 (*dd*, *J* = 8.4, 2.1 Hz, 1H, H-6′), 7.19-7.16 (ov, 2H, H-2′, H-7), 7.01 (*dd*, *J* = 8.2, 1.1 Hz, 1H, H-8), 7.01 (*td*, *J* = 7.4, 1.1 Hz, 1H, H-6), 6.91 (*d*, *J* = 8.4, 1H, H-5′), 5.49 (*dd*, *J* = 12.2, 4.1 Hz, 1H, H-6″), 4.04 (*t*, *J* = 3.1 Hz, 1H, H-4), 3.92 (*s*, 3H, 4′-OMe), 3.90 (*s*, 3H, 3′-OMe), 2.84 (*dd*, *J* = 17.5, 12.3 Hz, 1H, H-5″), 2.68 (*ddd*, *J* = 17.5, 4.1, 1.1 Hz, 1H, H-5″), 2.38 (*qd*, *J* = 13.5, 3.1, 2H, H-3); ^13^C NMR (100 MHz, CDCl_3_) δ 165.7 (C-2″ (D)), 163.6 (C-4″ (D)), 151.5 (C-9 (A)), 150.0 (C-4′ (B)), 149.0 (C-3′ (B)), 138.3 (C-1‴ (E)), 132.4 (C-1′ (B)), 129.1 (C-5 (A)), 128.9 (C-3‴ (E), C-4‴ (E)), 128.2 (C-7(A)), 126.2 (C-2‴ (E)), 126.0 (C-10 (A)), 122.0 (C-6(A)), 118.3 (C-6′ (B)), 116.5 (C-8 (A)), 110.9 (C-5′ (B)), 109.1 (C-2′ (B)), 106.7 (C-3″ (D)), 100.4 (C-2 (C)), 77.5 (C-6″ (D)), 56.3 (4′-OMe), 56.2 (3′-OMe), 34.3 (C-5″ (D)), 33.3 (C-3 (C)), 27.6 (C-4 (C)). Melting point: 172–174 °C. HRMS (ESI-TOF) *m/z* [M+H]^+^ Calcd. For C_28_H_24_O_6_ 456.1573, found 456.1573. Analytical HPLC (*λ* = 280 nm): purity: >99%; *t*_R_ = 20.7 min.

#### 3.3.19. 2-(3′,4′-Dimethoxyphenyl)-chromane-(4→3″, 2→O-4″)-6″-(4‴-chlorophenyl)-4″-hydroxy-5″,6″-dihydro-2H-pyran-2″-one (**63a** and **63b**)

General methodology B was applied using the flavylium salt **30** (0.182 g) and 6-(4-chlorophenyl)-4-hydroxy-5,6-dihydro-2*H*-pyran-2-one (**40**, 0.122 g, 0.5 mmol), previously prepared according to the methodology described by Andersh et al. [48,49]. The purification step was performed by CC using mixtures of hexane-EtOAc (75:25). Pure compound **63a** was obtained as a pale yellow amorphous solid (0.031 g, 12% from **21**) and pure compound **63b** as a pale yellow amorphous solid (0.050 g, 20% from **21**); Compound **63a**: ^1^H NMR (400 MHz, CDCl_3_) δ 7.37–7.28 (ov, 5H, H-5, H-2‴, H-3‴), 7.28–7.16 (ov, 2H, H-7, H-6′), 7.12 (*d*, *J* = 2.1 Hz, 1H, H-2′), 7.05 (*dd*, *J* = 7.7, 1.1 Hz, 1H, H-8), 6.96 (*td*, *J* = 7.4, 1.2 Hz, 1H, H-6), 6.90 (*d*, *J* = 8.5 Hz, 1H, H-5′), 5.29 (*dd*, *J* = 12.3, 4.1 Hz, 1H, H-6″), 4.28 (*t*, *J* = 2.8 Hz, 1H, H-4), 3.90 (*s*, 6H, 3′-OMe, 4′-OMe), 2.84 (*ddd*, *J* = 17.2, 12.3, 1.0 Hz, 1H, H-5″), 2.68 (*dd*, *J* = 17.2, 4.1 Hz, 1H, H-5″), 2.30 (*qd*, *J* = 13.5, 2.8 Hz, 2H, H-3); ^13^C NMR (100 MHz, CDCl_3_) δ 165.5 (C-2″ (D)), 163.1 (C-4″ (D)), 151.6 (C-9 (A)), 150.0 (C-4′ (B)), 149.0 (C-3′ (B)), 136.1 (C-1‴ ©), 134.7 (C-4‴ (E)), 132.1 (C-1′ (B)), 129.1 (C-3‴ (E)), 128.4 (C-7(A)), 128.1 (C-5 (A)), 127.6 (C-2‴ (E)), 126.3 (C-10 (A)), 122.2 (C-6(A)), 118.3 (C-6′ (B)), 116.5 (C-8 (A)), 110.9 (C-5′ (B)), 109.0 (C-2′ (B)), 106.6 (C-3″ (D)), 100.6 (C-2 (C)), 76.0 (C-6″ (D)), 56.2 (3′-OMe, 4′-OMe), 34.3 (C-5″ (D)), 33.1 (C-3 (C)), 26.8 (C-4 (C)). Melting point: 110–112 °C HRMS (ESI-TOF) *m/z* [M+H]^+^ Calcd. for C_28_H_23_ClO_6_ 490.1183, found 490.1177. Analytical HPLC (*λ* = 280 nm): purity: 97%; *t*_R_ = 21.8 min. Compound **63b**: ^1^H NMR (400 MHz, CDCl_3_) δ 7.53 (*dd*, *J* = 7.5, 1.2 Hz, 1H, H-5), 7.31–7.25 (*m*, 4H, H-2‴, H-3‴), 7.22-7.16 (ov, 3H, H-7, H-2′, H-6′), 7.01 (*dd*, *J* = 8.2, 1.2 Hz, 1H, H-8), 6.97 (*td*, *J* = 7.4, 1.2 Hz, 1H, H-6), 6.91 (*d*, *J* = 8.4 Hz, 1H, H-5′), 5.47 (*dd*, *J* = 12.0, 4.4 Hz, 1H, H-6″), 4.04 (*t*, *J* = 3.1 Hz, 1H, H-4), 3.92 (*s*, 3H, 3′-OMe), 3.90 (*s*, 3H, 4′-OMe), 2.79 (*ddd*, *J* = 17.5, 12.2, 0.8 Hz, 1H, H-5″), 2.67 (*ddd*, *J* = 17.5, 4.4, 1.2 Hz, 1H, H-5″), 2.39 (*qd*, *J* = 13.5, 3.1 Hz, 2H, H-3); ^13^C NMR (100 MHz, CDCl_3_) δ 165.4 (C-2″ (D)), 163.4 (C-4″ (D)), 151.5 (C-9 (A)), 150.1 (C-4′ (B)), 149.1 (C-3′ (B)), 136.8 (C-1‴ (E)), 134.7 (C-4‴ (E)), 132.3 (C-1′ (B)), 129.1 (C-3‴ (E)), 128.9 (C-5 (A)), 128.3 (C-7(A)), 127.6 (C-2‴ (E)), 125.9 (C-10 (A)), 122.1 (C-6(A)), 118.3 (C-6′ (B)), 116.2 (C-8 (A)), 110.9 (C-5′ (B)), 109.1 (C-2′ (B)), 106.8 (C-3″ (D)), 100.5 (C-2 (C)), 76.7 (C-6″ (D)), 56.3 (3′-OMe), 56.0 (4′-OMe), 34.2 (C-5″ (D)), 33.3 (C-3 (C)), 27.5 (C-4 (C)). Melting point: 173–176 °C HRMS (ESI-TOF) *m/z* [M+H]^+^ Calcd. for C_28_H_23_ClO_6_ 490.1183, found 490.1164. Analytical HPLC (*λ* = 280 nm): purity: >99%; *t*_R_ = 21.9 min.

#### 3.3.20. 2-(3′,4′-Dimethoxyphenyl)-chromane-(4→3″, 2→O-4″)-4″-hydroxy-6″-(4‴-methoxyphenyl)-5″,6″-dihydro-2H-pyran-2″-one (**64a** and **64b**)

General methodology B was applied using the flavylium salt **30** (0.182 g) and 4-hydroxy-6-(4-methoxyphenyl)-5,6-dihydro-2*H*-pyran-2-one (**41**, 0.110 g, 0.5 mmol), previously prepared according to the methodology described by Andersh et al. [48,49] (spectroscopic data agree with those reported in the literature [50]). The purification step was performed by CC using mixtures of hexane-EtOAc (75:25). Pure compound **64a** was obtained as a white amorphous solid (0.044 g, 17% from **21**) and pure compound **64b** as a white amorphous solid (0.052 g, 21% from **21**); Compound **64a**: ^1^H NMR (400 MHz, CDCl_3_) δ 7.36 (*dd*, *J* = 7.5, 1.6 Hz, 1H, H-5), 7.30–7.26 (*m*, 2H, H-2‴), 7.23–7.16 (ov, 2H, H-7, H-6′), 7.14 (*d*, *J* = 2.2 Hz, 1H, H-2′), 7.05 (*dd*, *J* = 8.2, 1.2 Hz, 1H, H-8), 6.96 (*td*, *J* = 7.4, 1.1 Hz, 1H, H-6), 6.90 (*d*, *J* = 8.4 Hz, 1H, H-5′), 6.89-6.85 (*m*, 2H, H-3‴), 5.25 (*dd*, *J* = 12.3, 4.0 Hz, 1H, H-6″), 4.28 (*t*, *J* = 3.1 Hz, 1H, H-4), 3.92 (*s*, 3H, 4′-OMe), 3.90 (*s*, 3H, 3′-OMe), 3.81 (*s*, 3H, 4‴-OMe), 3.00 (*ddd*, *J* = 17.2, 12.4, 4.0 Hz, 1H, H-5″), 2.64 (*dd*, *J* = 17.2, 4.0 Hz, 1H, H-5″), 2.29 (*qd*, *J* = 13.5, 3.2 Hz, 2H, H-3); ^13^C NMR (100 MHz, CDCl_3_) δ 165.9 (C-2″ (D)), 163.3 (C-4″ (D)), 160.0 (C-4‴ (E)), 151.6 (C-9 (A)), 150.0 (C-4′ (B)), 149.0 (C-3′ (B)), 132.3 (C-1′ (B)), 130.4 (C-1‴ (E)), 128.3 (C-7(A)), 128.1 (C-5 (A)), 128.0 (C-2‴ (E)), 126.5 (C-10 (A)), 122.2 (C-6(A)), 118.3 (C-6′ (B)), 116.5 (C-8 (A)), 114.2 (C-3‴ (E)), 110.9 (C-5′ (B)), 109.1 (C-2′ (B)), 106.5 (C-3″ (D)), 100.5 (C-2 (C)), 76.7 (C-6″ (D)), 56.2 (3′-OMe, 4′-OMe), 55.4 (4‴-OMe), 34.2 (C-5″ (D)), 33.1 (C-3 (C)), 26.7 (C-4 (C)). Melting point: 172–175 °C. HRMS (ESI-TOF) *m/z* [M+H]^+^ Calcd. For C_29_H_26_O_7_ 486.1679, found 486.1658. Analytical HPLC (*λ* = 280 nm): purity: 98%; *t*_R_ = 20.7 min. Compound **64b**: ^1^H NMR (400 MHz, CDCl_3_) δ 7.59 (*dd*, *J* = 7.6, 1.7 Hz, 1H, H-5), 7.32–7.28 (*m*, 2H, H-2‴), 7.28-7.20 (ov, 3H, H-7, H-2′, H-6′), 7.06 (*dd*, *J* = 8.2, 1.2 Hz, 1H, H-8), 7.01 (*td*, *J* = 7.5, 1.2 Hz, 1H, H-6), 6.95 (*d*, *J* = 8.4 Hz, 1H, H-5′), 6.92-6.86 (*m*, 2H, H-3‴), 5.48 (*dd*, *J* = 12.4, 4.0 Hz, 1H, H-6″), 4.08 (*t*, *J* = 3.2 Hz, 1H, H-4), 3.97 (*s*, 3H, 3′-Ome), 3.95 (*s*, 3H, 4′-OMe), 3.81 (*s*, 3H, 4‴-OMe), 2.89 (*dd*, *J* = 17.2, 12.4 Hz, 1H, H-5″), 2.68 (*ddd*, *J* = 17.2, 4.0, 1.2 Hz, 1H, H-5″), 2.42 (*qd*, *J* = 13.5, 3.2 Hz, 2H, H-3); ^13^C NMR (100 MHz, CDCl_3_) δ 165.8 (C-2″ (D)), 163.6 (C-4″ (D)), 160.0 (C-4‴ (E)), 151.5 (C-9 (A)), 149.9 (C-4′ (B)), 149.0 (C-3′ (B)), 132.4 (C-1′ (B)), 130.3 (C-1‴ (E)), 128.9 (C-7(A)), 128.1 (C-5 (A)), 127.8 (C-2‴ (E)), 126.0 (C-10 (A)), 121.9 (C-6(A)), 118.3 (C-6′ (B)), 116.1 (C-8 (A)), 114.1 (C-3‴ (E)), 110.9 (C-5′ (B)), 109.1 (C-2′ (B)), 106.6 (C-3″ (D)), 100.3 (C-2 (C)), 77.4 (C-6″ (D)), 56.2 (3′-OMe, 4′-OMe), 55.4 (4‴-OMe), 34.2 (C-5″ (D)), 33.3 (C-3 (C)), 27.5 (C-4 (C)). Melting point: 170–173 °C (decomposes). HRMS (ESI-TOF) *m/z* [M+H]^+^ Calcd. For C_29_H_26_O_7_ 486.1679, found 486.1663. Analytical HPLC (*λ* = 280 nm): purity: 98%; *t*_R_ = 20.6 min.

#### 3.3.21. 2-(3′,4′-Dimethoxyphenyl)-6-nitrochromane-(4→3″, 2→O-4″)-4″-hydroxy-6″-phenyl-5″,6″-dihydro-2H-pyran-2″-one (**65a** and **65b**)

General methodology B was applied using the flavylium salt **31** (0.205 g) and 4-hydroxy-6-phenyl-5,6-dihydro-2*H*-pyran-2-one (**39**, 0.095 g, 0.5 mmol), previously prepared according to the methodology described by Andersh et al. [48,49]. The purification step was performed by CC using mixtures of hexane-EtOAc (75:25). Pure compound **65a** was obtained as a pale yellow amorphous solid (0.015 g, 6% from **22**) and pure compound **65b** as a pale orange amorphous solid (0.081 g, 33% from **22**); Compound **65a**: ^1^H NMR (400 MHz, CDCl_3_) δ 8.28 (*d*, *J* = 2.7 Hz, 1H, H-5), 8.11 (*dd*, *J* = 9.0, 2.8 Hz, 1H, H-7), 7.37–7.33 (*m*, 5H, H-2‴, H-3‴, H-4‴), 7.16 (*dd*, *J* = 8.5, 2.2 Hz, 1H, H-6′), 7.12 (*d*, *J* = 9.0 Hz, 1H, H-8), 7.10 (*d*, *J* = 2.2 Hz, 1H, H-2′), 6.92 (*d*, *J* = 8.5 Hz, 1H, H-5′), 5.32 (*dd*, *J* = 12.0, 4.0 Hz, 1H, H-6″), 4.40 (*t*, *J* = 3.2 Hz, 1H, H-4), 3.91 (*s*, 6H, 3′-OMe, 4′-OMe), 3.03 (*ddd*, *J* = 17.2, 12.0, 1.1 Hz, 1H, H-5″), 2.74 (*dd*, *J* = 17.2, 4.1 Hz, 1H, H-5″), 2.36 (*d*, *J* = 3.2 Hz, 2H, H-3); ^13^C NMR (100 MHz, CDCl_3_) δ 165.5 (C-2″ (D)), 163.2 (C-4″ (D)), 157.0 (C-9 (A)), 150.4 (C-4′ (B)), 149.3 (C-3′ (B)), 142.5 (C-6(A)), 138.0 (C-1‴ (E)), 130.9 (C-1′ (B)), 129.0 (C-3‴ (E)), 129.1 (C-4‴ (E)), 127.3 (C-10 (A)), 126.2 (C-2‴ (E)), 124.5 (C-7(A)), 123.4 (C-5 (A)), 118.2 (C-6′ (B)), 117.1 (C-8 (A)), 111.0 (C-5′ (B)), 108.9 (C-2′ (B)), 106.1 (C-3″ (D)), 100.8 (C-2 (C)), 76.9 (C-6″ (D)), 56.3 (3′-OMe), 56.2 (4′-OMe), 34.0 (C-5″ (D)), 32.3 (C-3 (C)), 26.9 (C-4 (C)). Melting point: 198–200 °C. HRMS (ESI-TOF) *m/z* [M+H]^+^ Calcd. for C_28_H_23_NO_8_ 501.1424, found 501.1425. Analytical HPLC (*λ* = 280 nm): purity: 99%; *t*_R_ = 20.4 min. Compound **65b**: ^1^H NMR (400 MHz, CDCl_3_) δ 8.44 (*d*, *J* = 2.7 Hz, 1H, H-5), 8.07 (*dd*, *J* = 9.0, 2.8 Hz, 1H, H-7), 7.34–7.31 (*m*, 5H, H-2‴, H-3‴, H-4‴), 7.19 (*dd*, *J* = 8.4, 2.2 Hz, 1H, H-6′), 7.14 (*d*, *J* = 2.2 Hz, 1H, H-2′), 7.07 (*d*, *J* = 9.0 Hz, 1H, H-8), 6.92 (*d*, *J* = 8.5 Hz, 1H, H-5′), 5.51 (*dd*, *J* = 12.3, 4.1 Hz, 1H, H-6″), 4.14 (*br s*, 1H, H-4), 3.93 (*s*, 3H, 3′-OMe), 3.91 (*s*, 3H, 4′-OMe), 2.85 (*dd*, *J* = 17.7, 12.3 Hz, 1H, H-5″), 2.71 (*ddd*, *J* = 17.6, 4.1, 1.1 Hz, 1H, H-5″), 2.53 (*dd*, *J* = 13.7, 3.3 Hz, 1H, H-3), 2.38 (*dd*, *J* = 13.7, 2.8 Hz, 1H, H-3); ^13^C NMR (100 MHz, CDCl_3_) δ 165.3 (C-2″ (D)), 163.5 (C-4″ (D)), 156.8 (C-9 (A)), 150.3 (C-4′ (B)), 149.2 (C-3′ (B)), 142.3 (C-6(A)), 137.9 (C-1‴ (E)), 131.0 (C-1′ (B)), 129.0 (C-4‴ (E)), 128.9 (C-3‴ (E)), 126.9 (C-10 (A)), 126.1 (C-2‴ (E)), 124.8 (C-5 (A)), 124.4 (C-7(A)), 118.2 (C-6′ (B)), 116.7 (C-8 (A)), 111.0 (C-5′ (B)), 108.9 (C-2′ (B)), 106.1 (C-3″ (D)), 100.7 (C-2 (C)), 77.5 (C-6″ (D)), 56.3 (3′-OMe), 56.2 (4′-OMe), 34.2 (C-5″ (D)), 32.4 (C-3 (C)), 27.5 (C-4 (C)). Melting point: 143–145 °C. HRMS (ESI-TOF) *m/z* [M+H]^+^ Calcd. for C_28_H_23_NO_8_ 501.1424, found 501.1421. Analytical HPLC (*λ* = 280 nm): purity: >99%; *t*_R_ = 20.6 min.

#### 3.3.22. 2-(3′,4′-Dimethoxyphenyl)-6-nitrochromane-(4→3″, 2→O-4″)-6″-(4‴-chlorophenyl)-4″-hydroxy-5″,6″-dihydro-2H-pyran-2″-one (**66a** and **66b**)

General methodology B was applied using the flavylium salt **31** (0.205 g) and 6-(4-chlorophenyl)-4-hydroxy-5,6-dihydro-2*H*-pyran-2-one (**40**, 0.122 g, 0.5 mmol), previously prepared according to the methodology described by Andersh et al. [48,49]. The purification step was performed by CC using mixtures of hexane-EtOAc (75:25). Pure compound **66a** was obtained as a white amorphous solid (0.051 g, 19 % from **22**) and pure compound **66b** as a pale yellow amorphous solid (0.071 g, 26% from **22**); Compound **66a**: ^1^H NMR (400 MHz, CDCl_3_) δ 8.27 (*d*, *J* = 2.7 Hz, 1H, H-5), 8.10 (*dd*, *J* = 9.0, 2.7 Hz, 1H, H-7), 7.37–7.28 (*m*, 4H, H-2‴, H-3‴), 7.16 (*dd*, *J* = 8.5, 2.2 Hz, 1H, H-6′), 7.12 (*d*, *J* = 9.0 Hz, 1H, H-8), 7.09 (*d*, *J* = 2.22 Hz, 1H, H-2′), 6.91 (*d*, *J* = 8.5 Hz, 1H, H-5′), 5.30 (*dd*, *J* = 12.1, 4.1 Hz, 1H, H-6″), 4.38 (*t*, *J* = 3.1 Hz, 1H, H-4), 3.91 (*s*, 3H, 3′-OMe), 3.90 (*s*, 3H, 4′-OMe), 2.98 (*ddd*, *J* = 17.2, 12.1, 1.0 Hz, 1H, H-5″), 2.72 (*dd*, *J* = 17.2, 4.1 Hz, 1H, H-5″), 2.36 (*d*, *J* = 3.1 Hz, 2H, H-3); ^13^C NMR (100 MHz, CDCl_3_) δ 164.9 (C-2″ (D)), 163.0 (C-4″ (D)), 156.9 (C-9 (A)), 150.4 (C-4′ (B)), 149.2 (C-3′ (B)), 142.5 (C-6(A)), 136.5 (C-1‴ (E)), 134.9 (C-4‴ (E)), 130.7 (C-1′ (B)), 129.2 (C-3‴ (E)), 127.1 (C-2‴ (E), C-10 (A)), 124.5 (C-7(A)), 123.9 (C-5 (A)), 118.2 (C-6′ (B)), 117.1 (C-8 (A)), 111.0 (C-5′ (B)), 108.9 (C-2′ (B)), 106.1 (C-3″ (D)), 100.9 (C-2 (C)), 76.1 (C-6″ (D)), 56.3 (3′-OMe), 56.2 (4′-OMe), 34.0 (C-5″ (D)), 32.5 (C-3 (C)), 26.6 (C-4 (C)). Melting point: 194–196 °C. HRMS (ESI-TOF) *m/z* [M+H]^+^ Calcd. for C_28_H_22_NO_8_ 535.1034, found 535.1032. Analytical HPLC (*λ* = 280 nm): purity: 97%; *t*_R_ = 21.7 min. Compound (+)-**66a**: Chiral HPLC (*λ* = 280 nm): purity: >99%; *t*_R_ = 19.7 min. [α]_D_: +34 (c. 0.4, CHCl_3_). Compound (−)-**66a**: Chiral HPLC (*λ* = 280 nm): purity: >99%; *t*_R_ = 25.5 min. [α]_D_: −26 (c. 0.2, CHCl_3_). Compound **66b**: ^1^H NMR (400 MHz, CDCl_3_) δ 8.42 (*d*, *J* = 2.7 Hz, 1H, H-5), 8.08 (*dd*, *J* = 8.9, 2.7 Hz, 1H, H-7), 7.31–7.26 (*m*, 4H, H-2‴, H-3‴), 7.18 (*dd*, *J* = 8.4, 2.1 Hz, 1H, H-6′), 7.13 (*d*, *J* = 2.1 Hz, 1H, H-2′), 7.06 (*d*, *J* = 9.0 Hz, 1H, H-8), 6.91 (*d*, *J* = 8.5 Hz, 1H, H-5′), 5.49 (*dd*, *J* = 12.1, 4.2 Hz, 1H, H-6″), 4.12 (*br s*, 1H, H-4), 3.92 (*s*, 3H, 3′-OMe), 3.91 (*s*, 3H, 4′-OMe), 2.80 (*dd*, *J* = 17.6, 12.1 Hz, 1H, H-5″), 2.70 (*dd*, *J* = 17.6, 4.2 Hz, 1H, H-5″), 2.52 (*dd*, *J* = 13.8, 3.3 Hz, 1H, H-3), 2.38 (*dd*, *J* = 13.8, 2.8 Hz, 1H, H-3); ^13^C NMR (100 MHz, CDCl_3_) δ 165.1 (C-2″ (D)), 163.4 (C-4″ (D)), 156.8 (C-9 (A)), 150.4 (C-4′ (B)), 149.2 (C-3′ (B)), 142.4 (C-6(A)), 136.4 (C-1‴ (E)), 134.9 (C-4‴ (E)), 130.9 (C-1′ (B)), 129.1 (C-3‴ (E)), 127.6 (C-2‴ (E)), 126.8 (C-10 (A)), 124.8 (C-5 (A)), 124.4 (C-7(A)), 118.2 (C-6′ (B)), 116.8 (C-8 (A)), 111.0 (C-5′ (B)), 108.9 (C-2′ (B)), 106.1 (C-3″ (D)), 100.7 (C-2 (C)), 76.7 (C-6″ (D)), 56.3 (3′-OMe), 56.2 (4′-OMe), 34.1 (C-5″ (D)), 32.4 (C-3 (C)), 27.5 (C-4 (C)). Melting point: 154–157 °C. HRMS (ESI-TOF) *m/z* [M+H]^+^ Calcd. for C_28_H_22_NO_8_ 535.1034, found 535.1030. Analytical HPLC (*λ* = 280 nm): purity: >99%; *t*_R_ = 21.8 min.

#### 3.3.23. 2-(3′,4′-Dimethoxyphenyl)-6-nitrochromane-(4→3″, 2→O-4″)-4″-hydroxy-6″-(4‴-methoxyphenyl)-5″,6″-dihydro-2H-pyran-2″-one (**67a** and **67b**)

General methodology B was applied using the flavylium salt **31** (0.205 g) and 4-hydroxy-6-(4-methoxyphenyl)-5,6-dihydro-2*H*-pyran-2-one (**41**, 0.110 g, 0.5 mmol), previously prepared according to the methodology described by Andersh et al. [48,49] (spectroscopic data agree with those reported in the literature [50]). The purification step was performed by CC using mixtures of hexane-EtOAc (75:25). Pure compound **67a** was obtained as a yellow foam (0.040 g, 14% from **22**) and pure compound **67b** as a pale yellow amorphous solid (0.064 g, 24% from **22**); Compound **67a**: ^1^H NMR (400 MHz, CDCl_3_) δ 8.28 (*d*, *J* = 2.7 Hz, 1H, H-5), 8.10 (*dd*, *J* = 9.0, 2.7 Hz, 1H, H-7), 7.30–7.25 (*m*, 2H, H-2‴), 7.16 (*dd*, *J* = 8.5, 2.2 Hz, 1H, H-6′), 7.13ߝ7.09 (ov, 2H, H-8, H-2′), 6.92 (*d*, *J* = 8.5 Hz, 1H, H-5′), 6.90–6.84 (*m*, 2H, H-3‴), 5.26 (*dd*, *J* = 12.1, 4.1 Hz, 1H, H-6″), 4.39 (*t*, *J* = 3.0 Hz, 1H, H-4), 3.91 (*s*, 6H, 3′-OMe, 4′-OMe), 3.78 (*s*, 3H, 4‴-OMe), 3.03 (*ddd*, *J* = 17.3, 12.2, 1.0 Hz, 1H, H-5″), 2.69 (*dd*, *J* = 17.3, 4.1 Hz, 1H, H-5″), 2.40-2.32 (*m*, 2H, H-3); ^13^C NMR (100 MHz, CDCl_3_) δ 165.3 (C-2″ (D)), 163.3 (C-4″ (D)), 160.1 (C-4‴ (E)), 156.9 (C-9 (A)), 150.4 (C-4′ (B)), 149.2 (C-3′ (B)), 142.5 (C-6(A)), 138.0 (C-1‴ (E)), 130.9 (C-1′ (B)), 127.7 (C-2‴ (E)), 127.0 (C-10 (A)), 124.4 (C-7(A)), 123.7 (C-5 (A)), 118.2 (C-6′ (B)), 117.1 (C-8 (A)), 114.3 (C-3‴ (E)), 111.1 (C-5′ (B)), 108.9 (C-2′ (B)), 105.9 (C-3″ (D)), 100.8 (C-2 (C)), 76.7 (C-6″ (D)), 56.3 (3′-OMe), 56.2 (4′-OMe), 55.5 (4‴-OMe), 33.9 (C-5″ (D)), 32.3 (C-3 (C)), 26.6 (C-4 (C)). HRMS (ESI-TOF) *m/z* [M+H]^+^ Calcd. for C_29_H_25_NO_9_ 531.1529, found 531.1532. Analytical HPLC (*λ* = 280 nm): purity: >99%; *t*_R_ = 20.6 min. Compound **67b**: ^1^H NMR (400 MHz, CDCl_3_) δ 8.44 (*d*, *J* = 2.7 Hz, 1H, H-5), 8.08 (*dd*, *J* = 8.9, 2.8 Hz, 1H, H-7), 7.27–7.24 (*m*, 2H, H-2‴), 7.19 (*dd*, *J* = 8.4, 2.2 Hz, 1H, H-6′), 7.13 (*d*, *J* = 2.2 Hz, 1H, H-2′), 7.07 (*d*, *J* = 8.9 Hz, 1H, H-8), 6.92 (*d*, *J* = 8.5 Hz, 1H, H-5′), 6.87-6.82 (*m*, 2H, H-3‴), 5.45 (*dd*, *J* = 12.5, 4.0 Hz, 1H, H-6″), 4.13 (*br s*, 1H, H-4), 3.93 (*s*, 3H, 3′-OMe), 3.91 (*s*, 3H, 4′-OMe), 3.77 (*s*, 3H, 4‴-OMe), 2.86 (*dd*, *J* = 17.4, 12.5 Hz, 1H, H-5″), 2.67 (*ddd*, *J* = 17.4, 4.0, 1.2 Hz, 1H, H-5″), 2.51 (*dd*, *J* = 13.7, 3.3 Hz, 2H, H-3); ^13^C NMR (100 MHz, CDCl_3_) δ 165.5 (C-2″ (D)), 163.6 (C-4″ (D)), 160.1 (C-4‴ (E)), 156.9 (C-9 (A)), 150.4 (C-4′ (B)), 149.2 (C-3′ (B)), 142.4 (C-6(A)), 131.0 (C-1′ (B)), 129.9 (C-1‴ (E)), 127.8 (C-2‴ (E)), 126.9 (C-10 (A)), 125.0 (C-5 (A)), 124.4 (C-7(A)), 118.2 (C-6′ (B)), 116.7 (C-8 (A)), 114.2 (C-3‴ (E)), 111.0 (C-5′ (B)), 108.9 (C-2′ (B)), 106.1 (C-3″ (D)), 100.6 (C-2 (C)), 77.6 (C-6″ (D)), 56.3 (3′-OMe), 56.2 (4′-OMe), 55.6 (4‴-OMe), 34.1 (C-5″ (D)), 32.5 (C-3 (C)), 27.5 (C-4 (C)). Melting point: 162–164 °C. HRMS (ESI-TOF) *m/z* [M+H]^+^ Calcd. for C_29_H_25_NO_9_ 531.1529, found 531.1529. Analytical HPLC (*λ* = 280 nm): purity: >99%; *t*_R_ = 20.6 min.

#### 3.3.24. 2-(3′,4′-Dimethoxyphenyl)-6-chlorochromane-(4→3″, 2→O-4″)-4″-hydroxy-6″-phenyl-5″,6″-dihydro-2H-pyran-2″-one (**68a** and **68b**)

General methodology B was applied using the flavylium salt **32** (0.199 g) and 4-hydroxy-6-phenyl-5,6-dihydro-2*H*-pyran-2-one (**39**, 0.095 g, 0.5 mmol), previously prepared according to the methodology described by Andersh et al. [48,49]. The purification step was performed by CC using mixtures of hexane-EtOAc (75:25). Pure compound **68a** was obtained as a white amorphous solid (0.026 g, 12% from **23**) and pure compound **68b** as a white amorphous solid (0.066 g, 29% from **23**); Compound **68a**: ^1^H NMR (400 MHz, CDCl_3_) δ 7.38–7.30 (*m*, 6H, H-5, H-2‴, H-3‴, H-4‴), 7.17–7.13 (ov, 2H, H-7, H-6′), 7.10 (*d*, *J* = 2.2 Hz, 1H, H-2′), 6.97 (*d*, *J* = 8.7 Hz, 1H, H-8), 6.90 (*d*, *J* = 8.5 Hz, 1H, H-5′), 5.32 (*dd*, *J* = 12.2, 4.0 Hz, 1H, H-6″), 4.25 (*t*, *J* = 2.8 Hz, 1H, H-4), 3.90 (*s*, 6H, 3′-OMe, 4′-OMe), 3.00 (*ddd*, *J* = 17.2, 12.3, 1.1 Hz, 1H, H-5″), 2.71 (*dd*, *J* = 17.2, 4.1 Hz, 1H, H-5″), 2.30 (*qd*, *J* = 13.5, 2.8 Hz, 2H, H-3); ^13^C NMR (100 MHz, CDCl_3_) δ 165.5 (C-2″ (D)), 163.4 (C-4″ (D)), 150.3 (C-9 (A)), 150.1 (C-4′ (B)), 149.1 (C-3′ (B)), 138.8 (C-1‴ (E)), 131.8 (C-1′ (B)), 128.9 (C-5 (A), C-3‴ (E)), 128.3 (C-7(A)), 127.9 (C-10 (A)), 127.7 (C-4‴ (E)), 127.0 (C-6(A)), 126.2 (C-2‴ (E)), 118.3 (C-6′ (B)), 117.8 (C-8 (A)), 110.9 (C-5′ (B)), 109.0 (C-2′ (B)), 106.1 (C-3″ (D)), 100.6 (C-2 (C)), 76.9 (C-6″ (D)), 56.2 (3′-OMe, 4′-OMe), 34.2 (C-5″ (D)), 32.7 (C-3 (C)), 26.7 (C-4 (C)). Melting point: 106–108 °C HRMS (ESI-TOF) *m/z* [M+H]^+^ Calcd. for C_28_H_23_ClO_6_ 490.1182, found 490.1183. Analytical HPLC (*λ* = 280 nm): purity: >99%; *t*_R_ = 21.9 min. Compound (+)-**68a**: Chiral HPLC (*λ* = 280 nm): purity: >99%; *t*_R_ = 10.9 min. [α]_D_: + 17 (c. 0.5, CHCl_3_). Compound (−)-**68a**: Chiral HPLC (*λ* = 280 nm): purity: > 99%; *t*_R_ = 14.1 min. [α]_D_: −16 (c. 0.2, CHCl_3_). Compound **68b**: ^1^H NMR (400 MHz, CDCl_3_) δ 7.63 (*d*, *J* = 2.5 Hz, 1H, H-5), 7.47–7.40 (*m*, 5H, H-2‴, H-3‴, H-4‴), 7.28 (*dd*, *J* = 8.4, 2.2 Hz, 1H, H-6′), 7.25-7.19 (ov, 1H, H-7), 7.24 (*d*, *J* = 2.2 Hz, 1H, H-2′), 7.04-6.98 (ov, 1H, H-8, H-5′), 5.59 (*dd*, *J* = 12.3, 4.1 Hz, 1H, H-6″), 4.25 (*br s*, 1H, H-4), 3.90 (*s*, 6H, 3′-OMe, 4′-OMe), 2.94 (*dd*, *J* = 17.5, 12.3 Hz, 1H, H-5″), 2.78 (*ddd*, *J* = 17.5, 4.1, 1.1 Hz, 1H, H-5″), 2.46 (*qd*, *J* = 13.5, 2.8 Hz, 2H, H-3); ^13^C NMR (100 MHz, CDCl_3_) δ 165.5 (C-2″ (D)), 163.7 (C-4″ (D)), 150.2 (C-9 (A)), 150.1 (C-4′ (B)), 149.1 (C-3′ (B)), 138.1 (C-1‴ (E)), 131.9 (C-1′ (B)), 129.0 (C-4‴ (E)), 128.9 (C-3‴ (E)), 128.6 (C-5 (A)), 128.2 (C-7(A)), 127.5 (C-10 (A)), 126.8 (C-6(A)), 126.2 (C-2‴ (E)), 118.2 (C-6′ (B)), 117.4 (C-8 (A)), 110.9 (C-5′ (B)), 109.0 (C-2′ (B)), 106.2 (C-3″ (D)), 100.4 (C-2 (C)), 77.5 (C-6″ (D)), 56.3 (3′-Ome, 4′-Ome), 34.3 (C-5″ (D)), 32.9 (C-3 (C)), 27.5 (C-4 (C)). Melting point: 178–180 °C. HRMS (ESI-TOF) *m/z* [M+H]^+^ Calcd. For C_28_H_23_ClO_6_ 490.1182, found 490.1183. Analytical HPLC (*λ* = 280 nm): purity: 97%; *t*_R_ = 21.8 min. Compound (+)-**68b**: Chiral HPLC (*λ* = 280 nm): purity: >99%; *t*_R_ = 8.2 min. [α]_D_: + 236 (c. 0.5, CHCl_3_). Compound (−)-**68b**: Chiral HPLC (*λ* = 280 nm): purity: > 99%; *t*_R_ = 20.9 min. [α]_D_: −237 (c. 0.9, CHCl_3_).

#### 3.3.25. 2-(3′,4′-Dimethoxyphenyl)-6-chlorochromane-(4→3″, 2→O-4″)-6″-(4‴-chlorophenyl)-4″-hydroxy-5″,6″-dihydro-2H-pyran-2″-one (**69a** and **69b**)

General methodology B was applied using the flavylium salt **32** (0.199 g) and 6-(4-chlorophenyl)-4-hydroxy-5,6-dihydro-2*H*-pyran-2-one (**40**, 0.122 g, 0.5 mmol), previously prepared according to the methodology described by Andersh et al. [48,49]. The purification step was performed by CC using mixtures of hexane-EtOAc (75:25). Pure compound **69a** was obtained as a yellow amorphous solid (0.025 g, 10% from **23**) and pure compound **69b** as a pale orange amorphous solid (0.082 g, 33% from **23**); Compound **69a**: ^1^H NMR (400 MHz, CDCl_3_) δ 7.36–7.29 (*m*, 5H, H-5, H-2‴, H-3‴), 7.17–7.13 (ov, 2H, H-7, H-6′), 7.09 (*d*, *J* = 2.2 Hz, 1H, H-2′), 6.97 (*d*, *J* = 8.7 Hz, 1H, H-8), 6.90 (*d*, *J* = 8.5 Hz, 1H, H-5′), 5.30 (*dd*, *J* = 12.3, 4.1 Hz, 1H, H-6″), 4.24 (*br s*, 1H, H-4), 3.90 (*s*, 6H, 3′-OMe, 4′-OMe), 2.95 (*dd*, *J* = 17.2, 12.3 Hz, 1H, H-5″), 2.69 (*dd*, *J* = 17.2, 4.1 Hz, 1H, H-5″), 2.34-2.23 (*m*, 2H, H-3); ^13^C NMR (100 MHz, CDCl_3_) δ 165.3 (C-2″ (D)), 163.3 (C-4″ (D)), 150.2 * (C-4′ (B)), 150.1 * (C-9 (A)), 149.1 (C-3′ (B)), 136.7 (C-1‴ (E)), 134.8 (C-4‴ (E)), 131.7 (C-1′ (B)), 129.2 (C-3‴ (E)), 128.3 (C-7(A)), 127.8 (C-6(A)), 127.6 (C-2‴ (E)), 127.7 (C-5 (A)), 127.1 (C-10 (A)), 118.2 (C-6′ (B)), 117.8 (C-8 (A)), 110.9 (C-5′ (B)), 109.0 (C-2′ (B)), 106.2 (C-3″ (D)), 100.6 (C-2 (C)), 76.1 (C-6″ (D)), 56.2 (3′-OMe, 4′-OMe), 34.2 (C-5″ (D)), 32.7 (C-3 (C)), 26.6 (C-4 (C)); *these signals could be interchangeable. Melting point: 172–175 °C. HRMS (ESI-TOF) *m/z* [M+H]^+^ Calcd. for C_28_H_22_Cl_2_O_6_ 524.0793, found 524.0793. Analytical HPLC (*λ* = 280 nm): purity: 97%; *t*_R_ = 22.9 min. Compound **69b**: ^1^H NMR (400 MHz, CDCl_3_) δ 7.51 (*d*, *J* = 2.5 Hz, 1H, H-5), 7.34–7.24 (*m*, 4H, H-2‴, H-3‴), 7.17 (*dd*, *J* = 8.4, 2.2 Hz, 1H, H-6′), 7.16-7.09 (ov, 2H, H-2′, H-7), 6.93-6.91 (ov,*2*H, H-8, H-5′), 5.47 (*dd*, *J* = 12.2, 4.3 Hz, 1H, H-6″), 4.00 (*d*, *J* = 3.0 Hz, 1H, H-4), 3.92 (*s*, 3H, 3′-OMe), 3.90 (*s*, 3H, 4′-OMe), 2.80 (*dd*, *J* = 17.5, 12.2 Hz, 1H, H-5″), 2.68 (*ddd*, *J* = 17.5, 4.3, 1.0 Hz, 1H, H-5″), 2.37 (*qd*, *J* = 13.6, 3.0, 2H, H-3); ^13^C NMR (100 MHz, CDCl_3_) δ 166.2 (C-2″ (D)), 163.3 (C-4″ (D)), 150.1 (C-4′ (B), C-9 (A)), 149.1 (C-3′ (B)), 136.7 (C-1‴ (E)), 134.8 (C-4‴ (E)), 131.8 (C-1′ (B)), 129.1 (C-3‴ (E)), 128.5 (C-5 (A)), 128.2 (C-7(A)), 127.5 (C-2‴ (E)), 127.4 (C-10 (A)), 126.9 (C-6(A)), 118.2 (C-6′ (B)), 117.4 (C-8 (A)), 110.9 (C-5′ (B)), 109.2 (C-2′ (B)), 106.3 (C-3″ (D)), 100.5 (C-2 (C)), 76.7 (C-6″ (D)), 56.3 (3′-OMe), 56.2 (4′-OMe), 34.2 (C-5″ (D)), 32.8 (C-3 (C)), 27.4 (C-4 (C)). Melting point: 110–113 °C. HRMS (ESI-TOF) *m/z* [M+H]^+^ Calcd. for C_28_H_22_Cl_2_O_6_ 524.0793, found 524.0794. Analytical HPLC (*λ* = 280 nm): purity: 98%; *t*_R_ = 23.2 min.

#### 3.3.26. 2-(3′.4′-Dimethoxyphenyl)-6-chlorochromane-(4→3″, 2→O-4″)-4″-hydroxy-6″-(4‴-methoxyphenyl)-5″,6″-dihydro-2H-pyran-2″-one (**70a** and **70b**)

General methodology B was applied using the flavylium salt **32** (0.199 g) and 4-hydroxy-6-(4-methoxyphenyl)-5,6-dihydro-2*H*-pyran-2-one (**41**, 0.110 g, 0.5 mmol), previously prepared according to the methodology described by Andersh et al. [48,49] (spectroscopic data agree with those reported in the literature [50]). The purification step was performed by CC using mixtures of hexane-EtOAc (75:25). Pure compound **70a** was obtained as a white foam (0.032 g, 12% from **23**) and pure compound **70b** as a yellow amorphous solid (0.075 g, 29% from **23**); Compound **70a**: ^1^H NMR (400 MHz, CDCl_3_) δ 7.35 (*d*, *J* = 2.5 Hz, 1H, H-5), 7.29 (*d*, *J* = 8.7 Hz, 2H, H-2‴), 7.17–7.13 (ov, 2H, H-7, H-6′), 7.10 (*d*, J = 2.1 Hz, 1H, H-2′), 6.97 (*d*, *J* = 8.7 Hz, 1H, H-8), 6.92-6.87 (ov, 3H, H-5′, H-3‴), 5.26 (*dd*, *J* = 12.4, 4.0 Hz, 1H, H-6″), 4.24 (*br s*, 1H, H-4), 3.90 (*s*, 6H, 3′-OMe, 4′-OMe), 3.78 (*s*, 3H, 4‴-OMe), 3.00 (*dd*, *J* = 17.0, 12.6 Hz, 1H, H-5″), 2.65 (*dd*, *J* = 17.2, 4.0 Hz, 1H, H-5″), 2.36-2.20 (*m*, 2H, H-3); ^13^C NMR (100 MHz, CDCl_3_) δ 165.7 (C-2″ (D)), 163.5 (C-4″ (D)), 160.0 (C-4‴ (E)), 150.3 (C-9 (A)), 150.1 (C-4′ (B)), 149.1 (C-3′ (B)), 131.8 (C-1′ (B)), 130.2 (C-1‴ (E)), 127.8 (C-2‴ (E)), 128.2 (C-7(A)), 127.9 (C-10 (A)), 127.7 (C-5 (A)), 127.0 (C-6(A)), 118.2 (C-6′ (B)), 117.8 (C-8 (A)), 114.2 (C-3‴ (E)), 110.9 (C-5′ (B)), 109.0 (C-2′ (B)), 106.1 (C-3″ (D)), 100.5 (C-2 (C)), 76.7 (C-6″ (D)), 56.2 (3′-OMe, 4′-OMe), 55.3 (4‴-OMe), 34.1 (C-5″ (D)), 32.7 (C-3 (C)), 26.6 (C-4 (C)). HRMS (ESI-TOF) *m/z* [M+H]^+^ Calcd. for C_29_H_25_ClO_7_ 520.1289, found 520.1280. Analytical HPLC (*λ* = 280 nm): purity: 98%; *t*_R_ = 21.9 min. Compound **70b**: ^1^H NMR (400 MHz, CDCl_3_) δ 7.53 (*d*, *J* = 2.5 Hz, 1H, H-5), 7.27 (*d*, *J* = 8.7 Hz, 2H, H-2‴), 7.18 (*dd*, *J* = 8.4, 2.1 Hz, 1H, H-6′), 7.14-7.11 (ov, 2H, H-7, H-2′), 6.94-6.90 (ov, 2H, H-8, H-5′), 6.85 (*d*, *J* = 8.7 Hz, 2H, H-3‴), 5.44 (*dd*, *J* = 12.3, 3.8 Hz, 1H, H-6″), 4.00 (*br s*, 1H, H-4), 3.92 (*s*, 3H, 3′-OMe), 3.90 (*s*, 3H, 4′-OMe), 3.78 (*s*, 3H, 4‴-OMe), 2.85 (*dd*, *J* = 17.5, 12.5 Hz, 1H, H-5″), 2.64 (*dd*, *J* = 17.5, 3.8 Hz, 1H, H-5″), 2.36 (*qd*, *J* = 13.6, 3.2 Hz, 2H, H-3); ^13^C NMR (100 MHz, CDCl_3_) δ 165.7 (C-2″ (D)), 163.8 (C-4″ (D)), 160.1 (C-4‴ (E)), 150.2 (C-9 (A)), 150.1 (C-4′ (B)), 149.1 (C-3′ (B)), 132.0 (C-1′ (B)), 130.2 (C-1‴ (E)), 127.8 (C-2‴ (E)), 128.6 (C-5 (A)), 128.2 (C-7(A)), 127.5 (C-10 (A)), 126.8 (C-6(A)), 118.2 (C-6′ (B)), 117.4 (C-8 (A)), 114.2 (C-3‴ (E)), 110.9 (C-5′ (B)), 109.0 (C-2′ (B)), 106.2 (C-3″ (D)), 100.4 (C-2 (C)), 77.4 (C-6″ (D)), 56.3 (3′-OMe), 56.2 (4′-OMe), 55.5 (4‴-OMe), 34.2 (C-5″ (D)), 32.9 (C-3 (C)), 27.5 (C-4 (C)). Melting point: 108-111 °C HRMS (ESI-TOF) *m/z* [M+H]^+^ Calcd. for C_29_H_25_ClO_7_ 520.1289, found 520.1289. Analytical HPLC (*λ* = 280 nm): purity: 98%; *t*_R_ = 21.9 min.

### 3.4. Human Lactate Dehydrogenase A Enzymatic Activity Assay

The ability of the synthesized compounds to inhibit the *h*LDHA enzyme was measured using recombinant human LDHA (95%, specific activity >300 units/mg and concentration of 0.5 mg/mL, Abcam, Cambridge, United Kingdom) with sodium pyruvate (96%, Merck) as substrate and β-NADH (≥97%, Merck) as cofactor in a potassium phosphate buffer (100 mM, pH 7.3). The enzymatic assay was conducted on 96-well microplates and the decrease in the β-NADH fluorescence (λ_excitation_ = 340 nm; λ_emission_ = 460 nm) was detected in a TECAN *Infinite 200 Pro M Plex* fluorescent plate reader (Tecan Instrument, Inc.) at 28 °C. The activity was determined according to our previously described protocol [34,35]. The final volume in each well was set to 200 µL using 100 mM potassium phosphate buffer, 0.041 units/mL LDHA, 155 µM β-NADH, 1 mM pyruvate (saturated conditions), and DMSO solutions (5%, *v*/*v*) of pure compounds at concentrations in the range of 1–600 µM. The addition of pyruvate allowed the beginning of the reaction and fluorescence was registered every 60 s during 10 min. A lineal time interval was selected to calculate the slope at every single concentration. The establishment of the 0% and 100% enzymatic activity was performed by controls and by the use of the inhibitor 3-[[3[(cyclopropylamino)sulfonyl]-7-(2,4-dimethoxy-5-pyrimidinyl)-4-quinolinyl]amino]-5-(3,5-difluorophenoxy)benzoic acid (GSK 2,837,808 A, Tocris, MN, USA) at 1 µM [61]. The slope obtained at each concentration was compared to the one obtained for the 100% enzymatic activity control to determine the corresponding enzymatic activity. A nonlinear regression analysis in GraphPad Prism version 9.00 for Windows (GraphPad Software, La Jolla, CA, USA) was used for dose response curve, fitting of logarithm of inhibitor concentration vs. normalized enzymatic activity, to calculate IC_50_ values (Supplementary Material section). All measurements were made in triplicate and data were expressed as the mean ± SD.

### 3.5. Human Lactate Dehydrogenase B Enzymatic Activity Assay

The ability of the synthesized compounds to inhibit the *h*LDHB enzyme was measured using recombinant human LDHB (95%, specific activity >300 units/mg and concentration of 1.0 mg/mL, Abcam, Cambridge, UK) following the same fluorimetric protocol described in Section 3.4.

### 3.6. Method for Determining the Mechanism of Inhibition on hLDHA

For each compound evaluated, three replicates of four different concentrations of the compounds in the presence of eight substrate concentrations were included in the kinetic fluorometric assay. The preparation of 96-well microplates and the reaction mixture in each well is the same as that described in Section 3.4. After the addition of the substrate, the maximum slope per minute, determined in a linear interval for each well, was used to establish the initial velocity (*V*_0_) corresponding to each inhibitor and substrate concentration. Therefore, values of *V*_0_ were obtained as a mean of three replicates. Nonlinear regression fits of *V*_0_ *versus* substrate concentration have been performed in GraphPad Prism 9.00 for Windows (GraphPad Software, La Jolla, California, USA) to calculate *V_max_, K_M_* and *K_i_*. Linear Lineweaver–Burk double-reciprocal plots were created using Microsoft Excel 2019 (Microsoft Office Professional Plus 2019) to propose a mechanism of inhibition on *h*LDHA.

## 4. Conclusions

The synthesis of 38 compounds (**42**–**70**) with a 2,8-dioxabicyclo[3.3.1]nonane core has been carried out following a two-step methodology previously used by us. The yields of these syntheses are significantly affected by the substituents of the electrophilic flavylium salt (**27**–**35**) and by the type of nucleophile added in the second stage (**36**–**41**). As expected, the highest yields were obtained when the more nucleophilic unit (4-hydroxycoumarin, **37**) and/or when EWGs (NO_2_ and Cl) in the flavylium salt unit were used (42–81%).

Most of the synthesized (±)-2,8-dioxabicyclo[3.3.1]nonane compounds showed higher inhibitory activity against *h*LDHA than against *h*LDHB. Nine of the new compounds (**43**, **44**, **47**, **58**, **60**, **62a**, **65b**, **66a**, and **66b**) and the reference compound **2**, previously synthesized, showed IC_50_ values against *h*LDHA lower than 10 µM. On the other hand, all of the synthesized compounds showed IC_50_ values against *h*LDHB higher than 10 µM. In fact, only three of the new compounds (**46**, **51** and **54**) presented a slightly higher inhibitory activity against *h*LDHB than against *h*LDHA. Quiral HPLC was used to separate enantiomers of a selection of the most active and selective compounds synthesized (**66a**, **68a**, and **68b**). The inhibition assays performed with these pure enantiomers revealed that the influence of the absolute spatial configurations in the inhibition behaviors of the selected 2,8-dioxabicyclo[3.3.1]nonane derivatives is not very high, although it seems that it is always favorable to dextrorotatory enantiomers on *h*LDHA. Moreover, the study of the inhibition kinetics of enantiomers of **68a** and **68b** seems to indicate a noncompetitive inhibition behavior for all cases and a slight preference for the dextrorotatory enantiomer (2–4 times more potent) during the inhibition kinetic process on *h*LDHA enzyme.

All these studies seem to indicate that 2,8-dioxabicyclo[3.3.1]nonane derivatives are promising inhibitors in terms of potency and selectivity against *h*LDHA enzyme. However, some additional tests have to be performed in order to reduce possible unspecific inhibition due to protein aggregation induced by the tested compounds (using a BSA/Triton enriched medium).

A structure–activity analysis has been conducted taking into account potency and selectivity against *h*LDHA and *h*LDHB enzymes, concluding that the presence of electron–withdrawing groups (NO_2_ and Cl) at the A-ring, two methoxy groups at the B-ring, and preferably a pyrone moiety in the final compounds seems to be important features that 2,8-dioxabicyclo[3.3.1]nonane derivatives should fulfil in order to ensure achieving *h*LDHA inhibitors with high activity and selectivity (**62a**, **65b** and **68a**).

## Figures and Tables

**Table 1:** Reaction yields in the synthesis of flavylium salts **27**−**35** and (±)-dioxabicycles **1**-**6** and **42**−**70**.

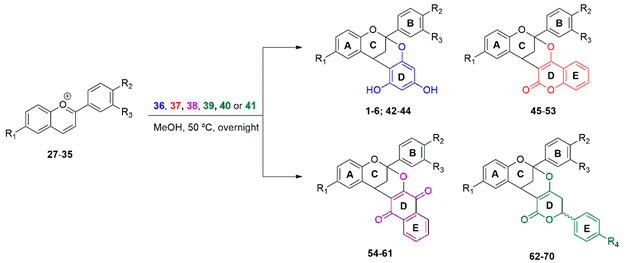						
Entry	Compound	R_1_	R_2_	R_3_	R_4_	Yield ^1^
1	**27**	H	OH	OH	-	90%
2	**28**	NO_2_	OH	OH	-	77%
3	**29**	Cl	OH	OH	-	79%
4	**30**	H	OMe	OMe	-	96%
5	**31**	NO_2_	OMe	OMe	-	99%
6	**32**	Cl	OMe	OMe	-	99%
7	**33**	H	OH	H	-	79%
8	**34**	NO_2_	OH	H	-	76%
9	**35**	Cl	OH	H	-	63%
10	(±)-**1 ^2^**	H	OH	OH	-	34%
11	(±)-**2 ^2^**	NO_2_	OH	OH	-	64%
12	(±)-**3 ^2^**	Cl	OH	OH	-	43%
13	(±)-**42**	H	OMe	OMe	-	38%
14	(±)-**43**	NO_2_	OMe	OMe	-	52%
15	(±)-**44**	Cl	OMe	OMe	-	62%
16	(±)-**4 ^2^**	H	OH	H	-	22%
17	(±)-**5 ^2^**	NO_2_	OH	H	-	45%
18	(±)-**6 ^2^**	Cl	OH	H	-	42%
19	(±)-**45**	H	OH	OH	-	72%
20	(±)-**46**	NO_2_	OH	OH	-	79%
21	(±)-**47**	Cl	OH	OH	-	81%
22	(±)-**48**	H	OMe	OMe	-	55%
23	(±)-**49**	NO_2_	OMe	OMe	-	54%
24	(±)-**50**	Cl	OMe	OMe	-	81%
25	(±)-**51**	H	OH	H	-	56%
26	(±)-**52**	NO_2_	OH	H	-	58%
27	(±)-**53**	Cl	OH	H	-	57%
28	(±)-**54**	H	OH	OH	-	10%
29	(±)-**55**	NO_2_	OH	OH	-	47%
30	(±)-**56**	Cl	OH	OH	-	19%
31	(±)-**57**	H	OMe	OMe	-	23%
32	(±)-**58**	NO_2_	OMe	OMe	-	42%
33	(±)-**59**	Cl	OMe	OMe	-	67%
34	(±)-**60**	NO_2_	OH	H	-	46%
35	(±)-**61**	Cl	OH	H	-	15%
36	(±)-**62a**	H	OMe	OMe	H	5%
	(±)-**62b**	H	OMe	OMe	H	9%
37	(±)-**63a**	H	OMe	OMe	Cl	12%
	(±)-**63b**	H	OMe	OMe	Cl	20%
38	(±)-**64a**	H	OMe	OMe	OMe	17%
	(±)-**64b**	H	OMe	OMe	OMe	21%
39	(±)-**65a**	NO_2_	OMe	OMe	H	6%
	(±)-**65b**	NO_2_	OMe	OMe	H	33%
40	(±)-**66a**	NO_2_	OMe	OMe	Cl	19%
	(±)-**66b**	NO_2_	OMe	OMe	Cl	26%
41	(±)-**67a**	NO_2_	OMe	OMe	OMe	14%
	(±)-**67b**	NO_2_	OMe	OMe	OMe	24%
42	(±)-**68a**	Cl	OMe	OMe	H	12%
	(±)-**68b**	Cl	OMe	OMe	H	29%
43	(±)-**69a**	Cl	OMe	OMe	Cl	10%
	(±)-**69b**	Cl	OMe	OMe	Cl	33%
44	(±)-**70a**	Cl	OMe	OMe	OMe	12%
	(±)-**70b**	Cl	OMe	OMe	OMe	29%

^1 ^Yields calculated from the starting aldehydes **21**–**23** (see Scheme 1). ^2^ These compounds have been previously synthesized by us [35] and their yields are included here for comparative purposes.

**Table 2:** Inhibition activity of (±)-2,8-dioxabicyclo[3.3.1]nonane derivatives against *h*LDHA and *h*LDHB.

Compound	*h*LDHA IC_50_ (μM) ^2^	R^2^	*h*LDHB IC_50_ (μM) ^2^	R^2^	Selectivity Rate *(*IC_50_ *h*LDHB/*h*LDHA)
(±)-**1** ^1^	73.1 ± 2.5	0.87	35.4 ± 0.8	0.93	0.5
(±)-**2** ^1^	9.7 ± 1.1	0.93	28.6 ± 1.9	0.97	3.0
(±)-**3** ^1^	15.7 ± 2.7	0.94	39.7 ± 2.8	0.98	2.5
(±)-**42**	153.6 ± 11.3	0.93	293.1 ± 2.4	0.98	1.9
(±)-**43**	8.2 ± 0.2	0.97	65.3 ± 2.1	0.96	8.0
(±)-**44**	9.6 ± 0.3	0.98	23.1 ± 1.1	0.95	2.4
(±)-**4** ^1^	60.0 ± 4.7	0.92	45.9 ± 1.5	0.96	0.8
(±)-**5** ^1^	24.4 ± 2.7	0.94	69.7 ± 4.7	0.95	2.9
(±)-**6** ^1^	26.7 ± 0.8	0.94	85.5 ± 2.5	0.98	3.2
(±)-**45**	21.4 ± 0.5	0.98	28.6 ± 0.6	0.95	1.3
(±)-**46**	28.5 ± 0.4	0.99	14.1 ± 0.3	0.96	0.5
(±)-**47**	9.1 ± 0.5	0.97	14.3 ± 0.9	0.99	1.6
(±)-**48**	49.6 ± 1.7	0.97	>300	-	>6.0
(±)-**49**	25.7 ± 0.3	0.97	212.3 ± 1.4	0.98	8.3
(±)-**50**	13.2 ± 0.8	0.94	>300	-	>22.7
(±)-**51**	34.4 ± 0.8	0.91	31.5 ± 3.0	0.97	0.9
(±)-**52**	36.9 ± 1.2	0.97	66.2 ± 1.0	0.93	1.8
(±)-**53**	11.2 ± 0.6	0.97	18.3 ± 1.0	0.97	1.6
(±)-**54**	20.7 ± 0.5	0.99	13.8 ± 0.2	0.98	0.7
(±)-**55**	27.4 ± 0.6	0.93	29.0 ± 2.4	0.94	1.1
(±)-**56**	13.7 ± 0.6	0.98	118.9 ± 2.7	0.99	8.7
(±)-**57**	42.2 ± 2.4	0.91	>300	-	>7.1
(±)-**58**	8.5 ± 1.0	0.96	>300	-	>35.3
(±)-**59**	20.4 ± 1.2	0.98	266.1 ± 4.8	0.96	13.0
(±)-**60**	4.1 ± 0.4	0.96	13.2 ± 1.3	0.97	3.2
(±)-**61**	20.1 ± 1.6	0.97	22.8 ± 1.4	0.96	1.1
(±)-**62a**	3.6 ± 0.3	0.94	150.3 ± 3,9	0.97	41.8
(±)-**62b**	39.4 ± 1.2	0.97	>300	-	>7.6
(±)-**63a**	17.4 ± 0.3	0.97	250.6 ± 10.5	0.97	14.4
(±)-**63b**	50.8 ± 0.4	0.98	296.1 ± 2.7	0.99	5.8
(±)-**64a**	52.8 ± 5.3	0.98	>300	-	>5.7
(±)-**64b**	96.7 ± 9.0	0.95	>300	-	>3.1
(±)-**65a**	12.5 ± 1.1	0.97	269.2 ± 11.0	0.97	21.5
(±)-**65b**	8.5 ± 0.4	0.98	>300	-	>35.3
(±)-**66a**	5.8 ± 0.2	0.98	28.1 ± 0.4	0.99	4.8
(±)-**66b**	6.0 ± 0.1	0.97	20.4 ± 0.7	0.98	3.4
(±)-**67a**	14.8 ± 0.6	0.93	>300	-	>20.3
(±)-**67b**	14.6 ± 0.3	0.98	53.7 ± 0.9	0.99	3.7
(±)-**68a**	12.0 ± 0.9	0.99	>300	-	>25.0
(±)-**68b**	28.5 ± 0.5	0.97	>300	-	>10.5
(±)-**69a**	20.0 ± 0.6	0.99	140.9 ± 2.5	0.99	7.1
(±)-**69b**	14.2 ± 0.8	0.98	77.0 ± 9.7	0.98	5.4
(±)-**70a**	112.2 ± 3.0	0.99	>300	-	>2.7
(±)-**70b**	20.2 ± 0.7	0.95	292.3 ± 4.1	0.98	14.5

^1^ These compounds have been synthesized and evaluated previously by us [35] and their inhibition data are included here for comparative purposes. ^2^ IC_50_ data are presented as the mean ± SD of *n* = 3 replicates.

**Table 3:** Inhibition activity (*h*LDHA and *h*LDHB) of pure enantiomers of a selection of the most active and selective 2,8-dioxabicyclo[3.3.1]nonane derivatives.

Compound	*h*LDHA IC_50_ (μM)	R^2^	*h*LDHB IC_50_ (μM)	R^2^	Selectivity Rate *(*IC_50_ *h*LDHB/*h*LDHA)
(+)-**66a**	9.1 ± 0.5	0.99	43.2 ± 1.7	0.99	4.8
(−)-**66a**	14.9 ± 0.8	0.96	40.8 ± 0.2	0.98	2.7
(+)-**68a**	10.2 ± 0.3	0.98	>300	-	>29.4
(−)-**68a**	15.8 ± 0.6	0.99	>300	-	>19.0
(+)-**68b**	27.3 ± 0.2	0.99	>300	-	>11.0
(−)-**68b**	27.6 ± 1.8	0.96	>300	-	>11.0

## Data Availability

Data are contained within the article and Supplementary Materials.

## Supplementary Materials

The following supporting information can be downloaded at: https://www.mdpi.com/article/10.3390/ijms24129925/s1.
