# Supplementary material for: Synthesis and hLDH Inhibitory Activity of Analogues to Natural Products with 2,8-Dioxabicyclo[3.3.1]nonane Scaffold
Source: Int J Mol Sci. 2023 Jun 8;24(12):9925. doi: 10.3390/ijms24129925 (PMC10298284; doi:10.3390/ijms24129925)
Supplement: Supplementary file 1 [file ijms-24-09925-s001.zip › ijms-2384295-supplementary.pdf]

Synthesis and *h*LDH inhibitory activity of analogues to natural products with 2,8-dioxabicyclo[3.3.1]nonane scaffold

Sofia Salido, Alfonso Alejo-Armijo*, Joaquin Altarejos

Departamento de Química Inorgánica y Orgánica, Facultad de Ciencias Experimentales, Universidad de Jaén, Campus de Excelencia Internacional Agroalimentario ceiA3, 23071 Jaén, Spain. aalejo@ujaen.es

Table of Contents

1. NMR spectra S1

2. HPLC chromatograms S42

3. Quiral HPLC chromatograms S57

4. Dose response curves against *h*LDHA and *h*LDHB S59

5. Dose response curves against *h*LDHA and *h*LDHB of pure enantiomers S92

6. Lineweaver-Burk double reciprocal graphs for pure enantiomers on *h*LDHA S95

7. Values of V_{max} and K_M for pure enantiomers on *h*LDHA S97

1. NMR spectra

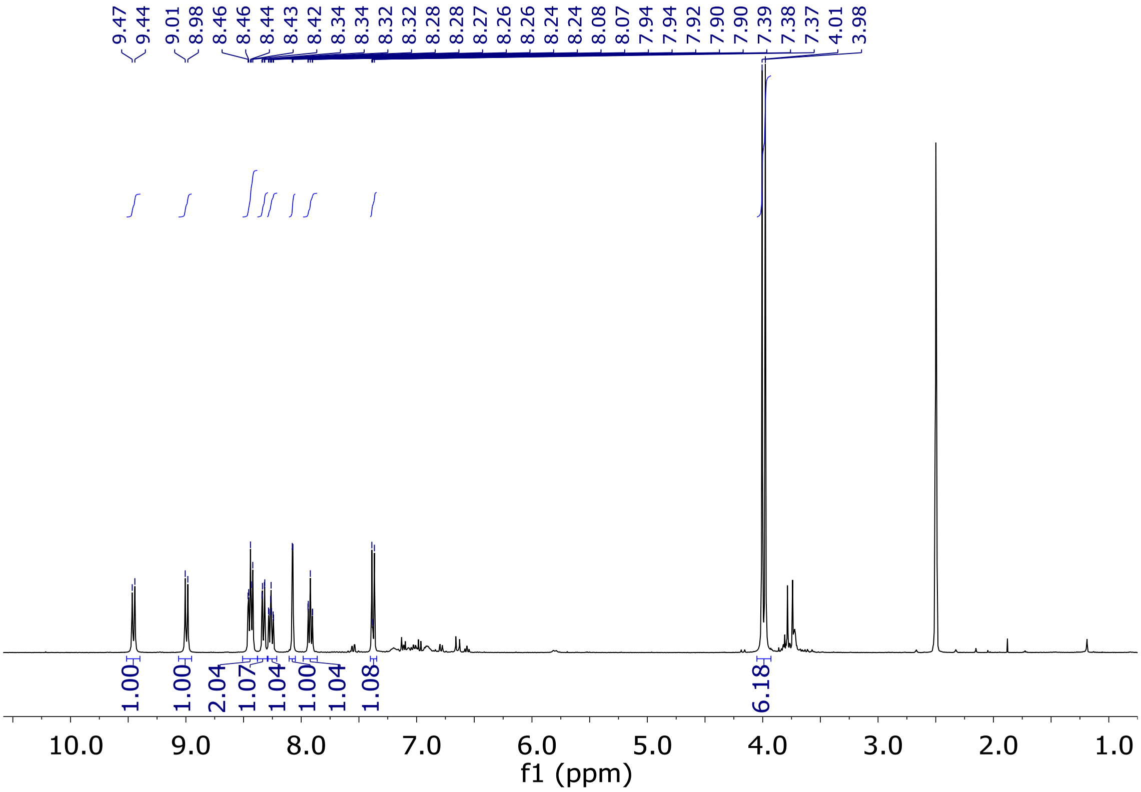

Figure S1.^{1}H-NMR spectrum of compound **30** (flavylium species) in DMSO:TFA 9:1.

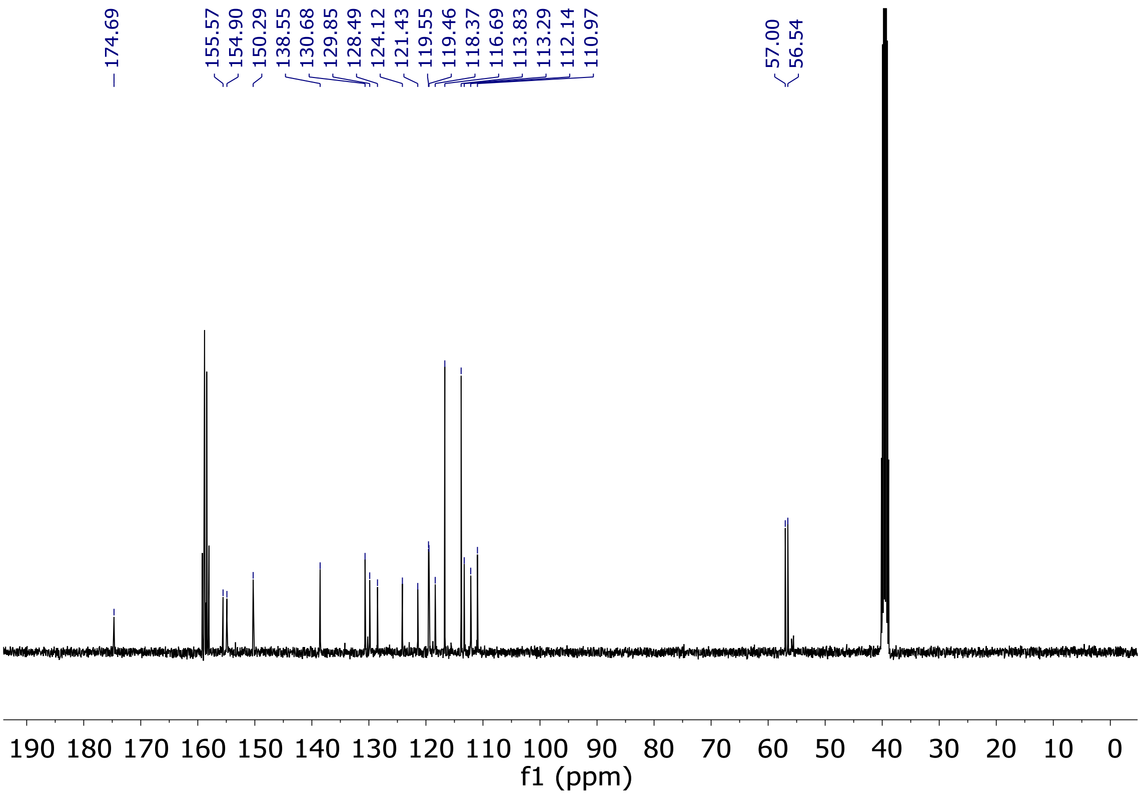

Figure S2.^{13}C-NMR spectrum of **30** (flavylium species) in DMSO:TFA 9:1.

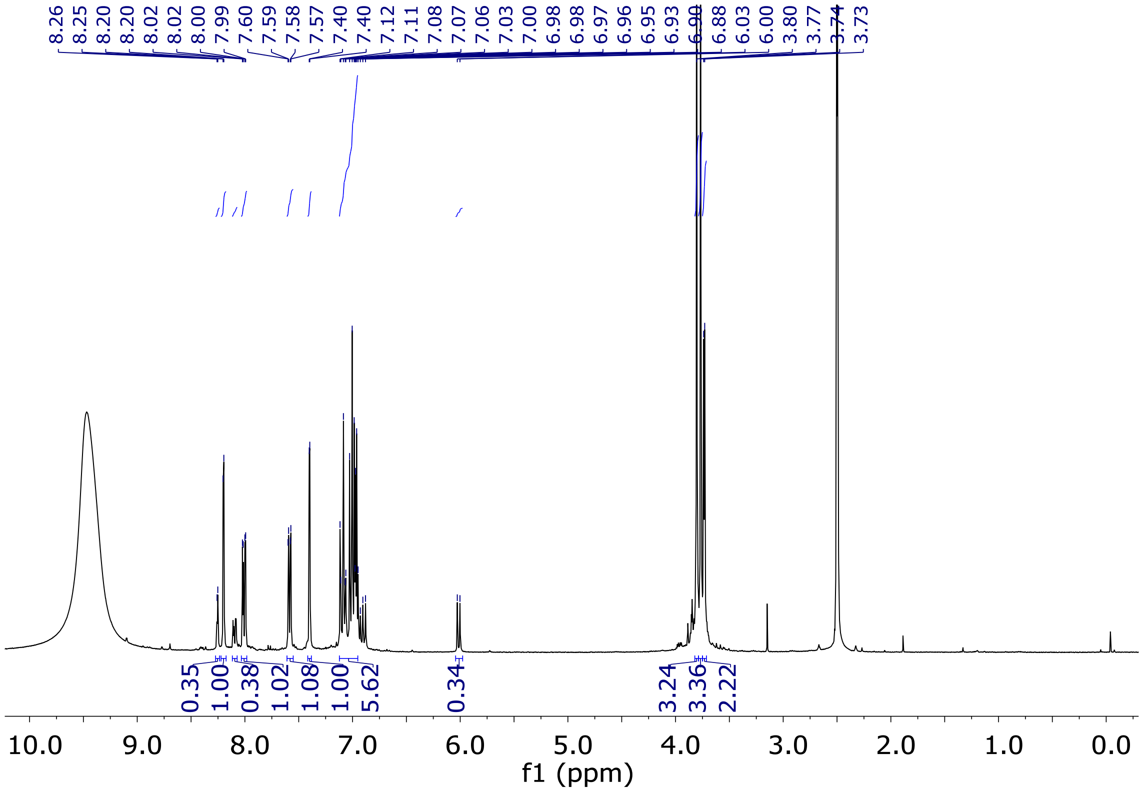

Figure S3.^{1}H-NMR spectrum of compound **31** (*trans*-chalcone species) in DMSO:TFA 9:1.

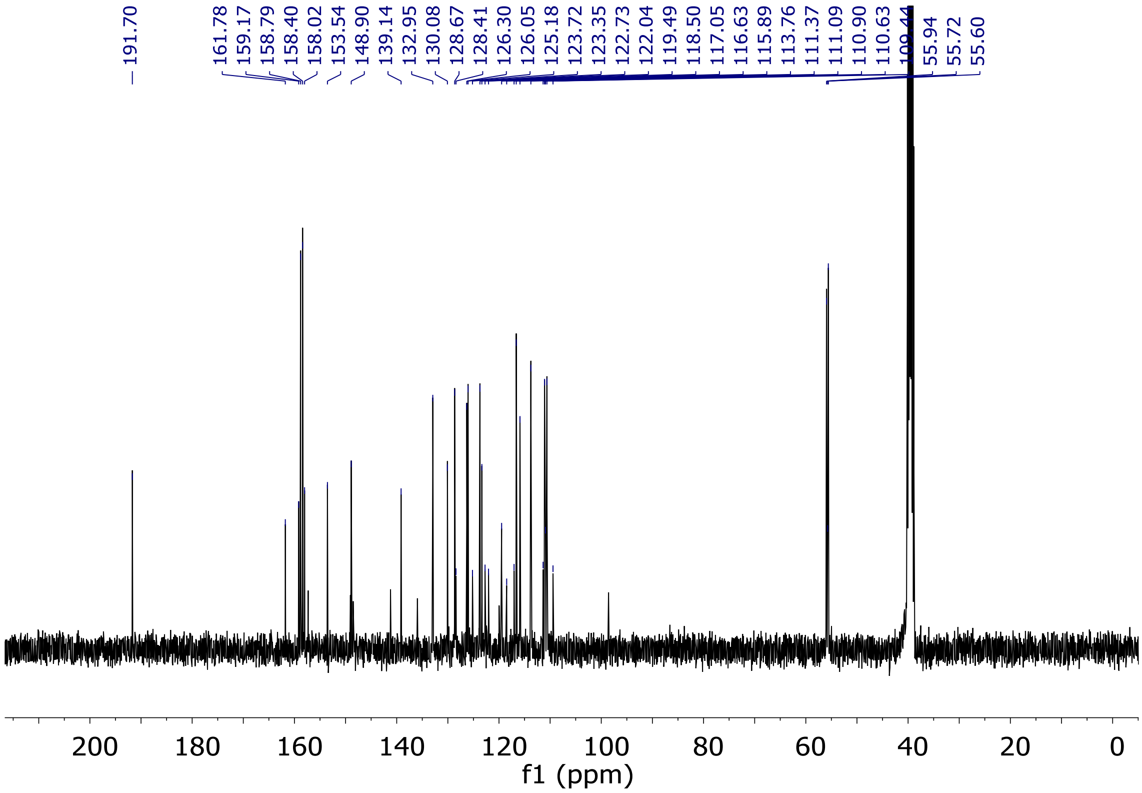

Figure S4^{13}C-NMR spectrum of compound **31** (*trans*-chalcone species) in DMSO:TFA 9:1.

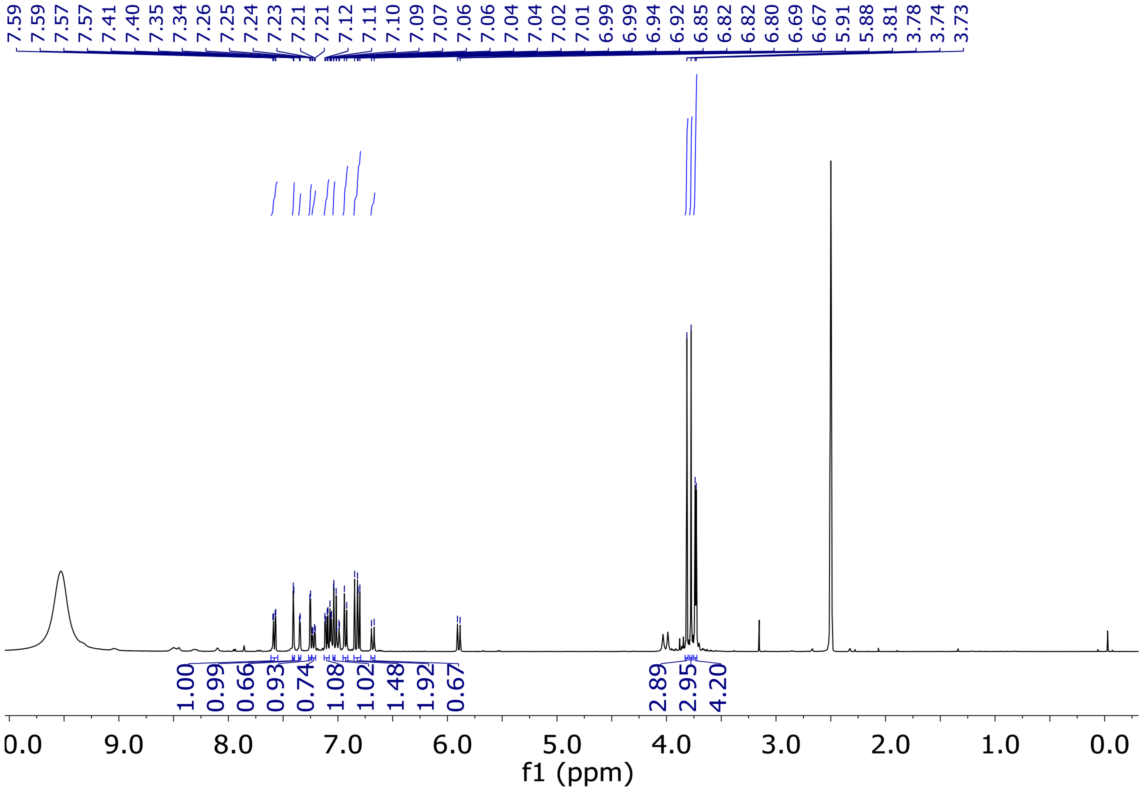

Figure S5.^{1}H-NMR spectrum of compound **32** (*trans*-chalcone species) in DMSO:TFA 9:1.

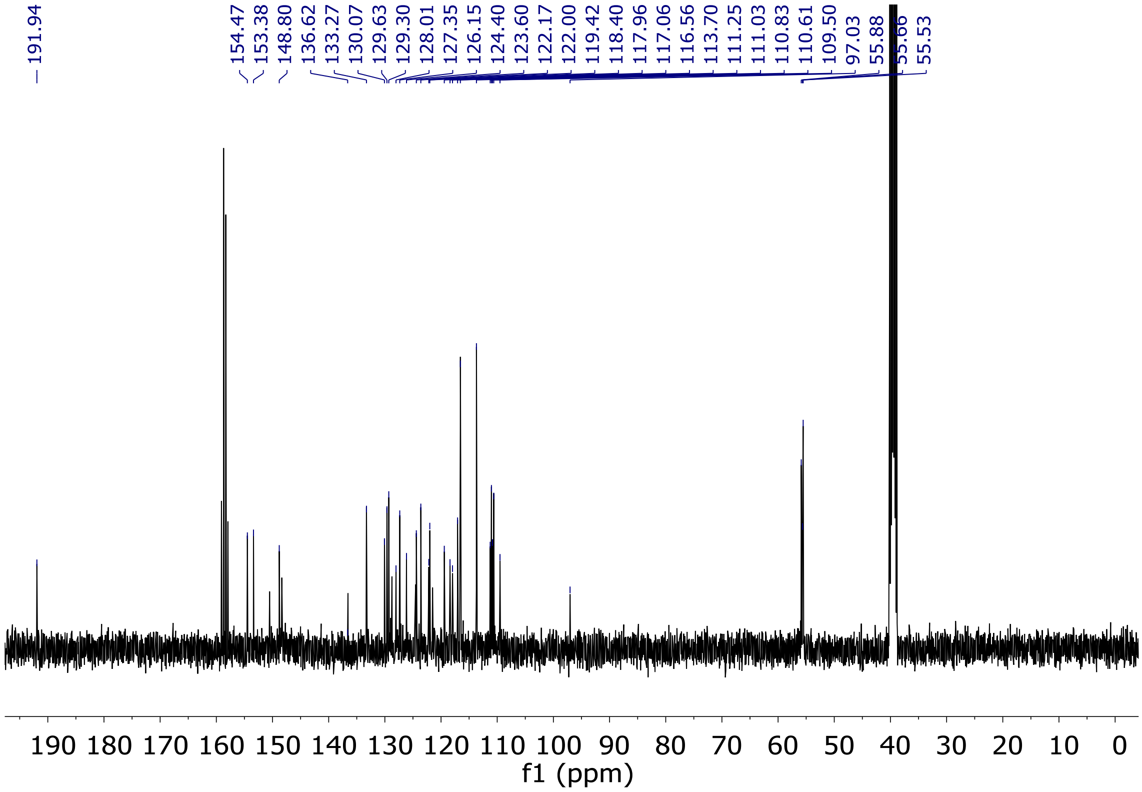

Figure S6.^{13}C-NMR spectrum of compound **32** (*trans*-chalcone species) in DMSO:TFA 9:1.

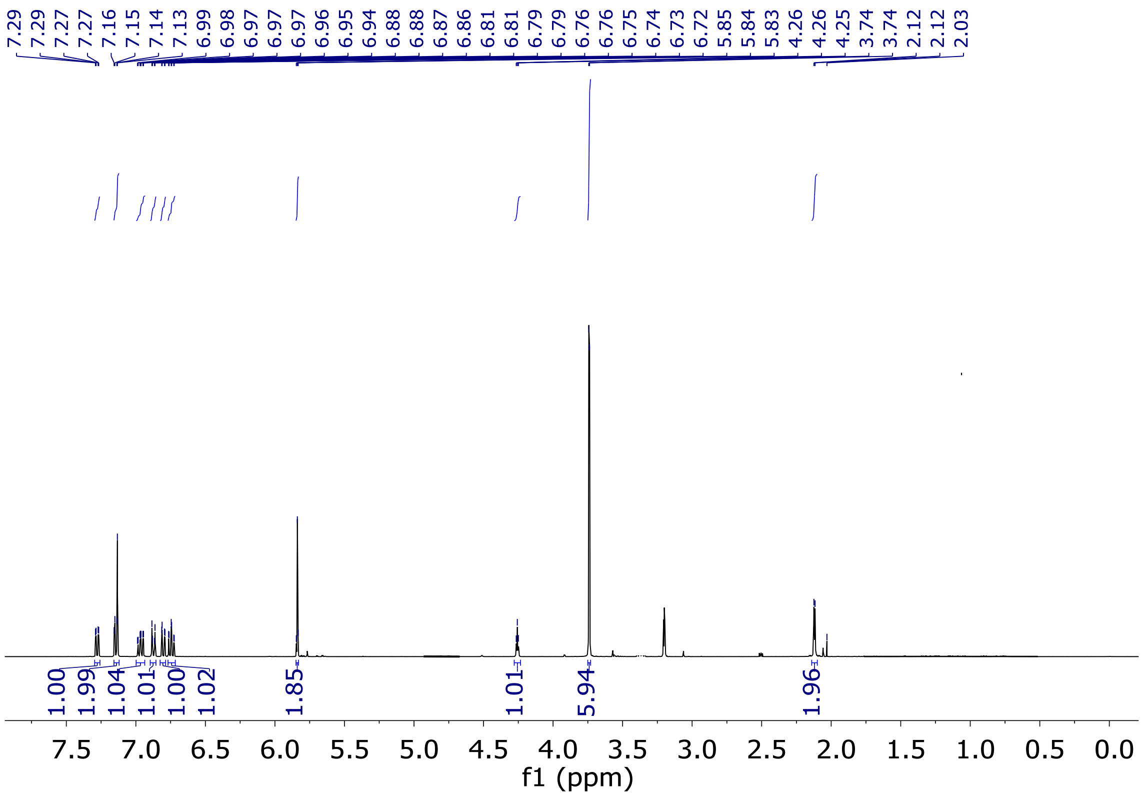

Figure S7.^{1}H-NMR spectrum of compound **42** in MeOD

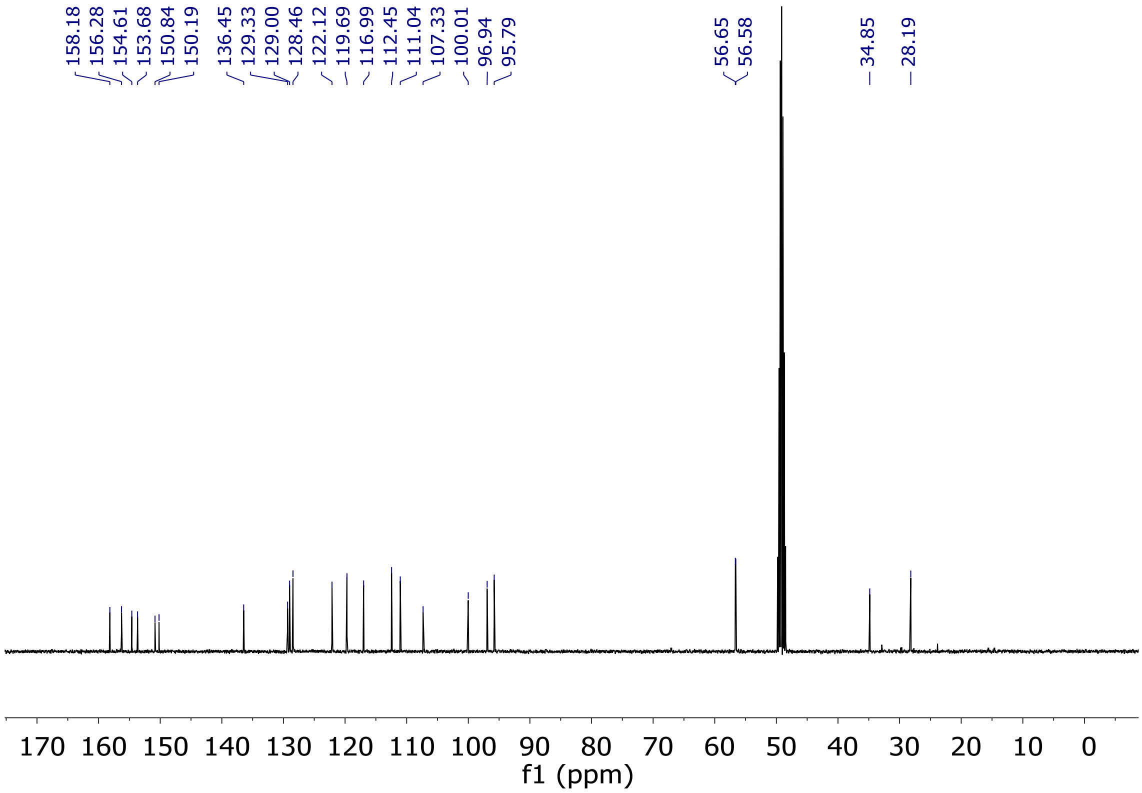

Figure S8.^{13}C-NMR spectrum of compound **42** in MeOD

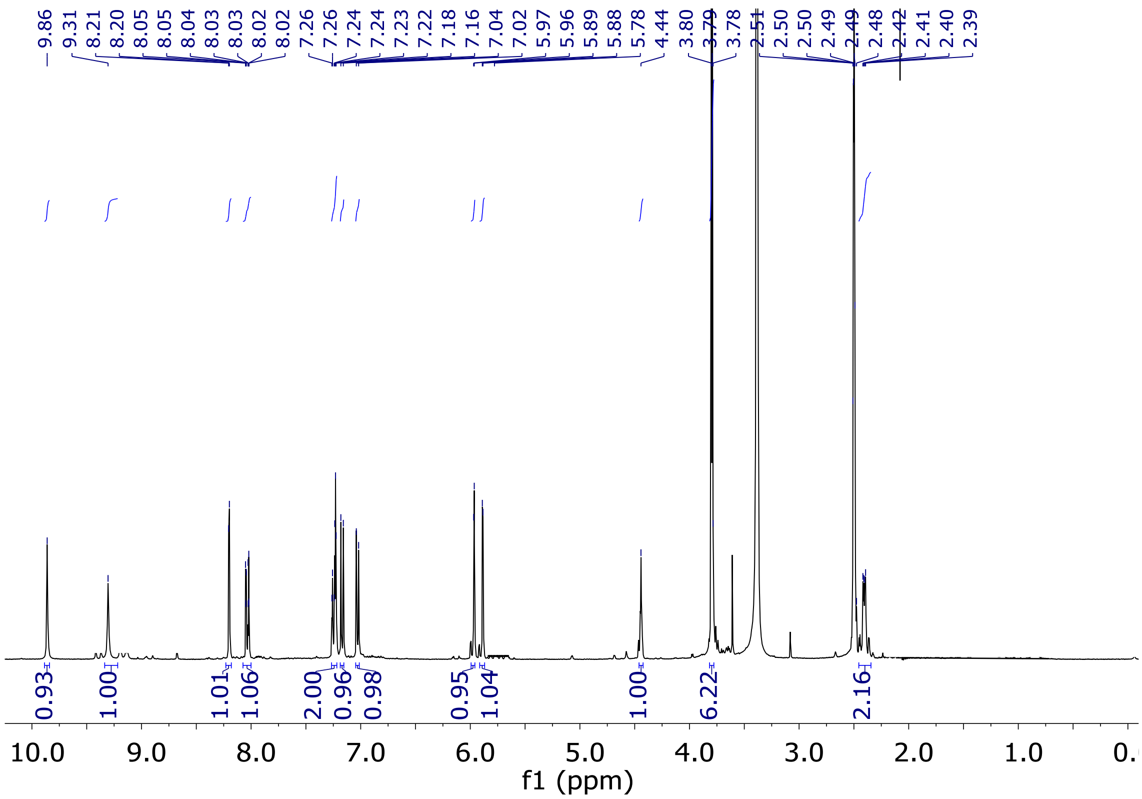

Figure S9.^{1}H-NMR spectrum of compound **43** in DMSO-d_6

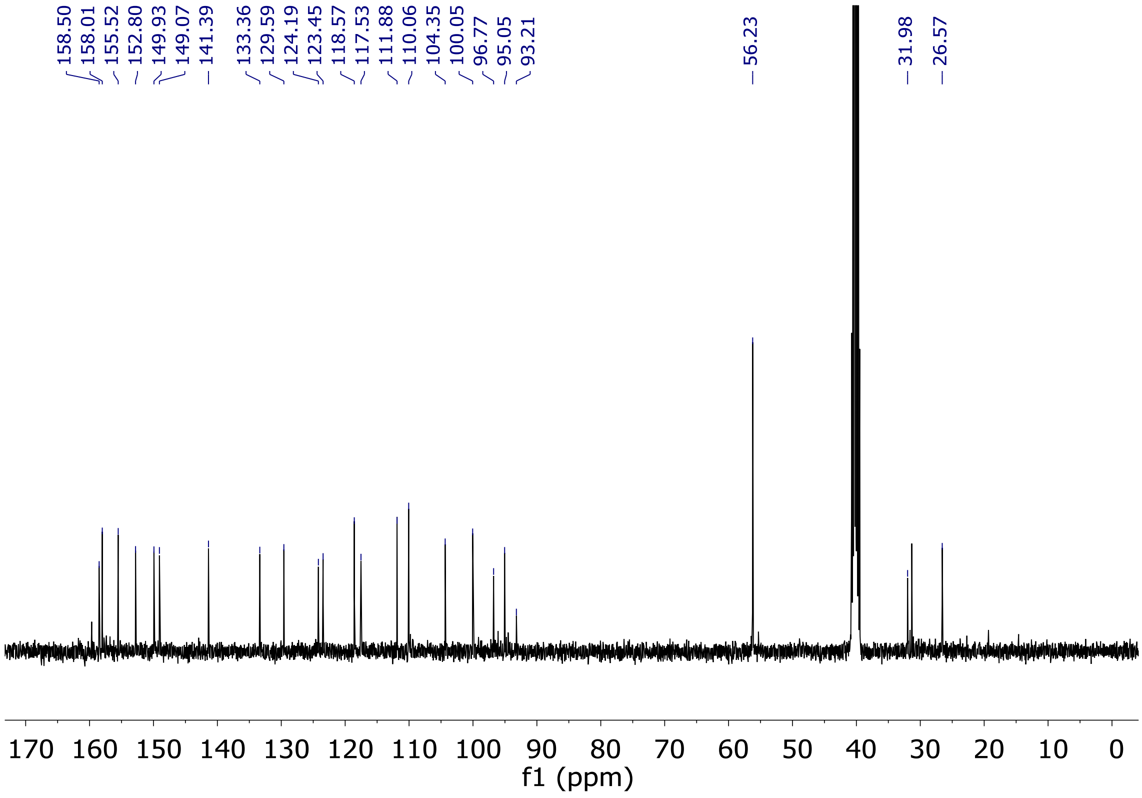

Figure S10.^{13}C-NMR spectrum of compound **43** in DMSO-d_6

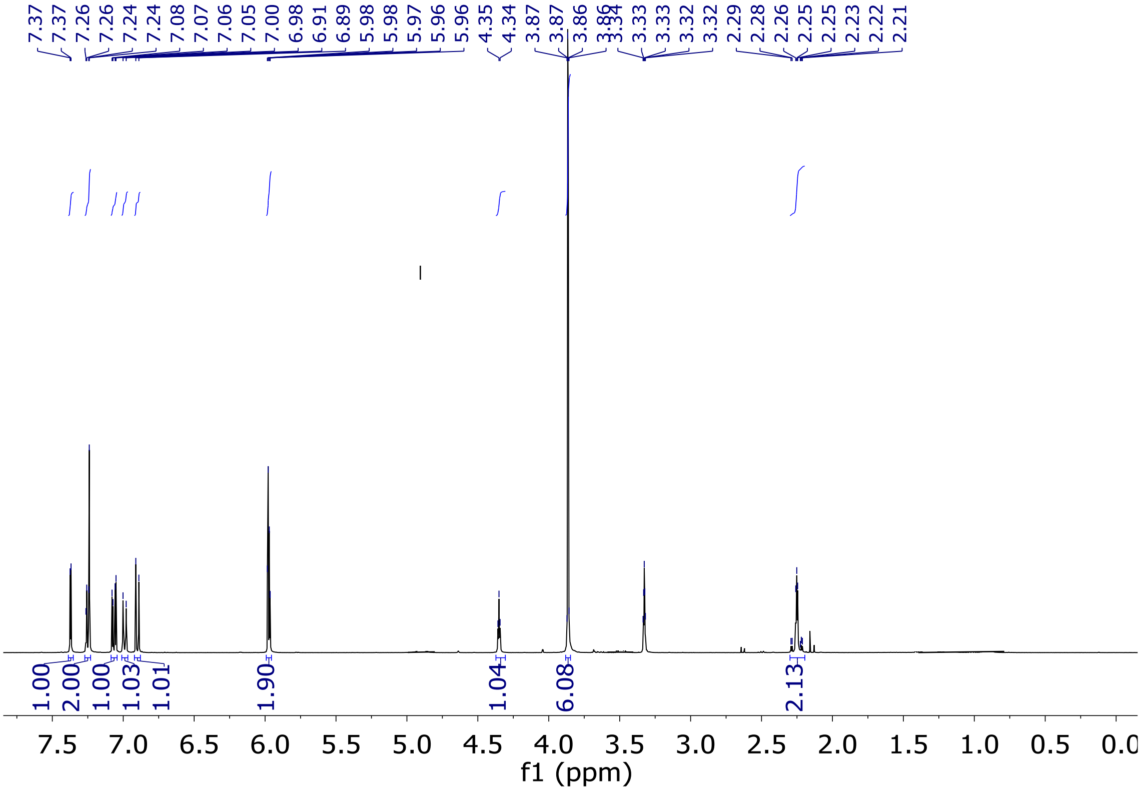

Figure S11.^{1}H-NMR spectrum of compound **44** in MeOD.

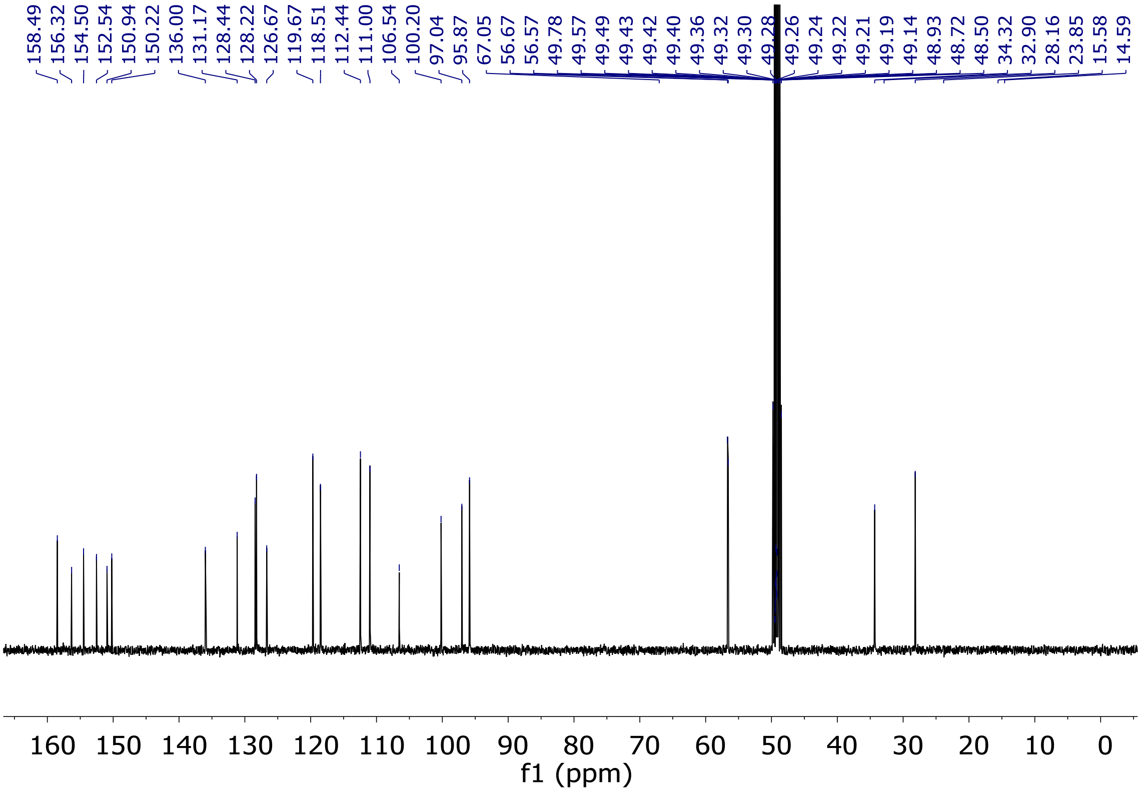

Figure S12.^{13}C-NMR spectrum of compound **44** in MeOD.

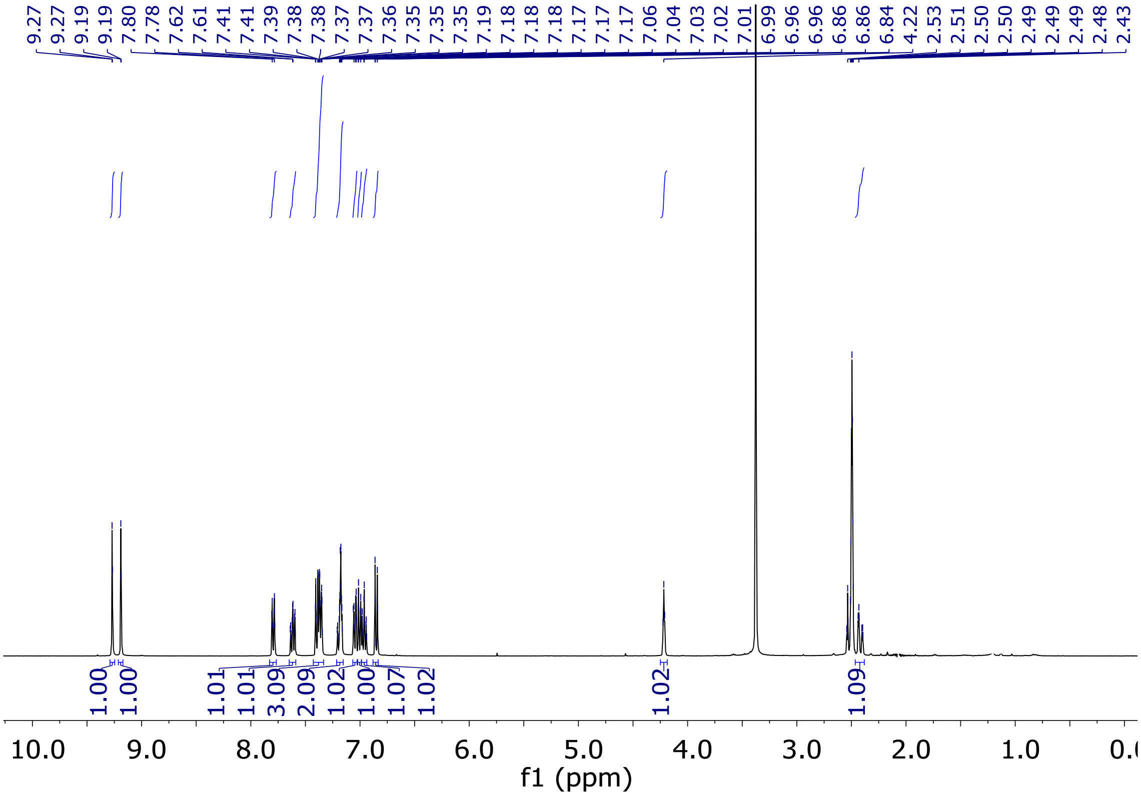

Figure S13.^{1}H-NMR spectrum of compound **45** in DMSO-d_6

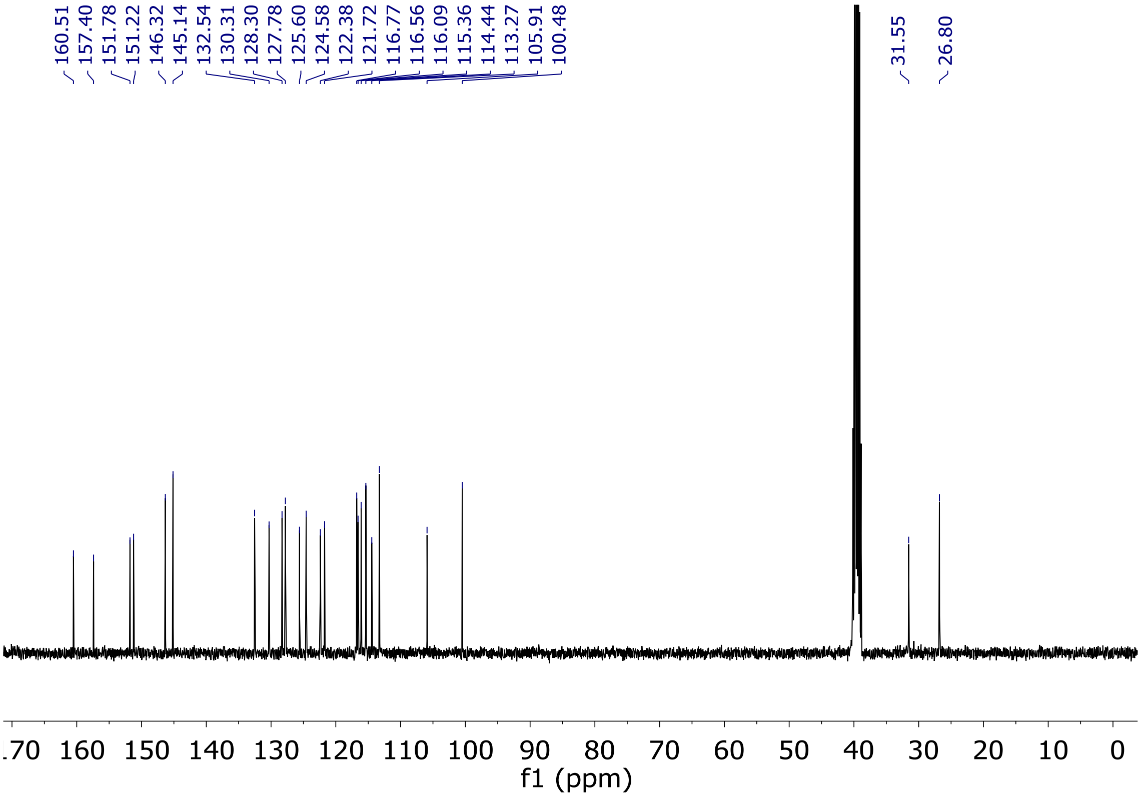

Figure S14.^{13}C-NMR spectrum of compound **45** in DMSO-d_6

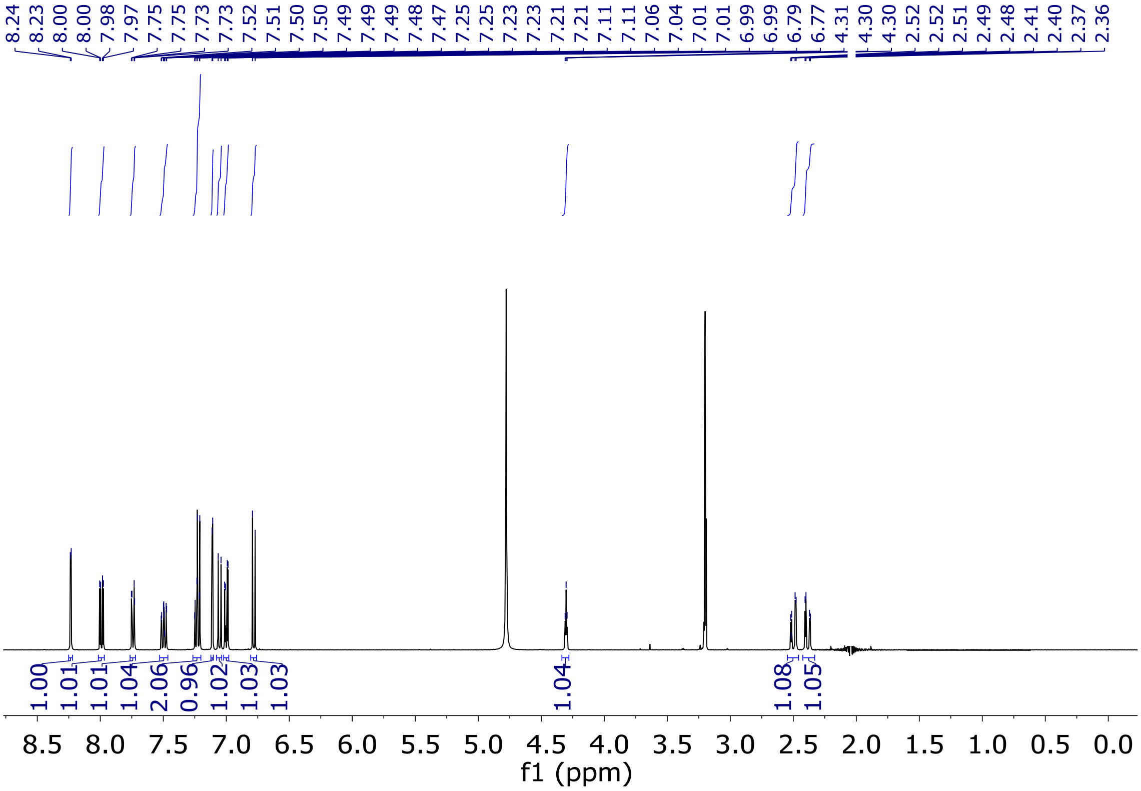

Figure S15.^{1}H-NMR spectrum of compound **46** in MeOD

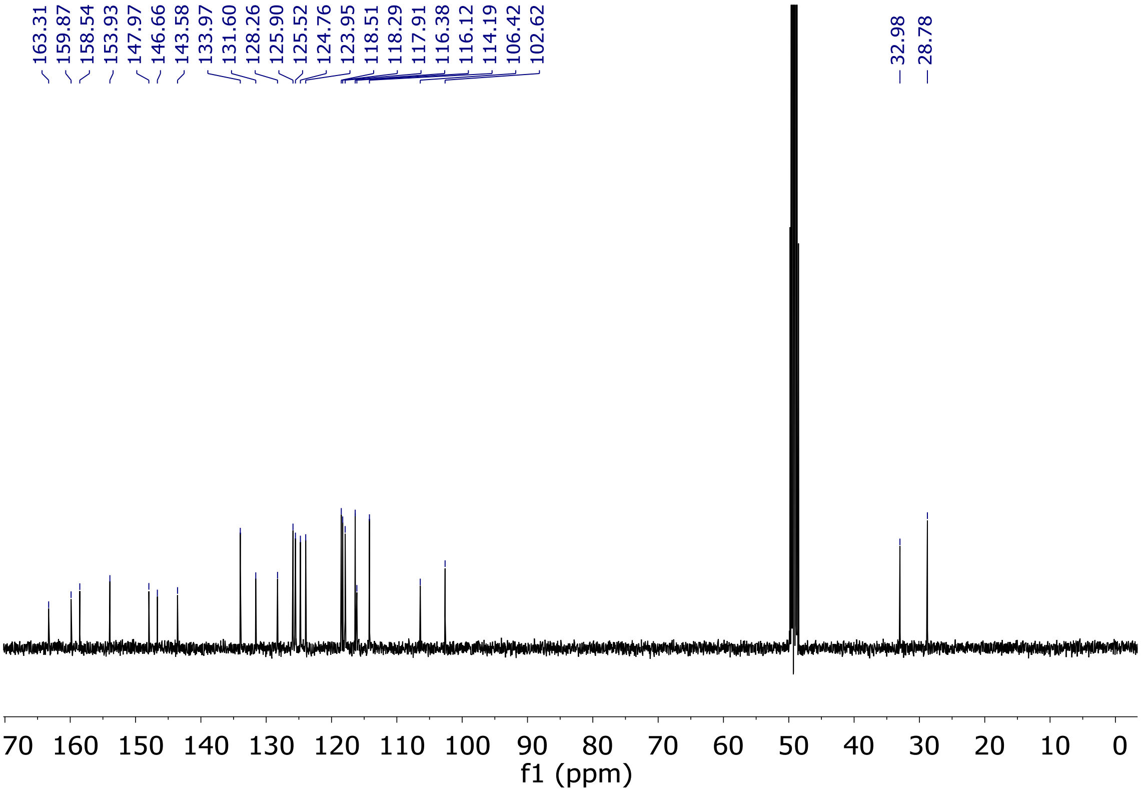

Figure S16.^{13}C-NMR spectrum of compound **46** in MeOD

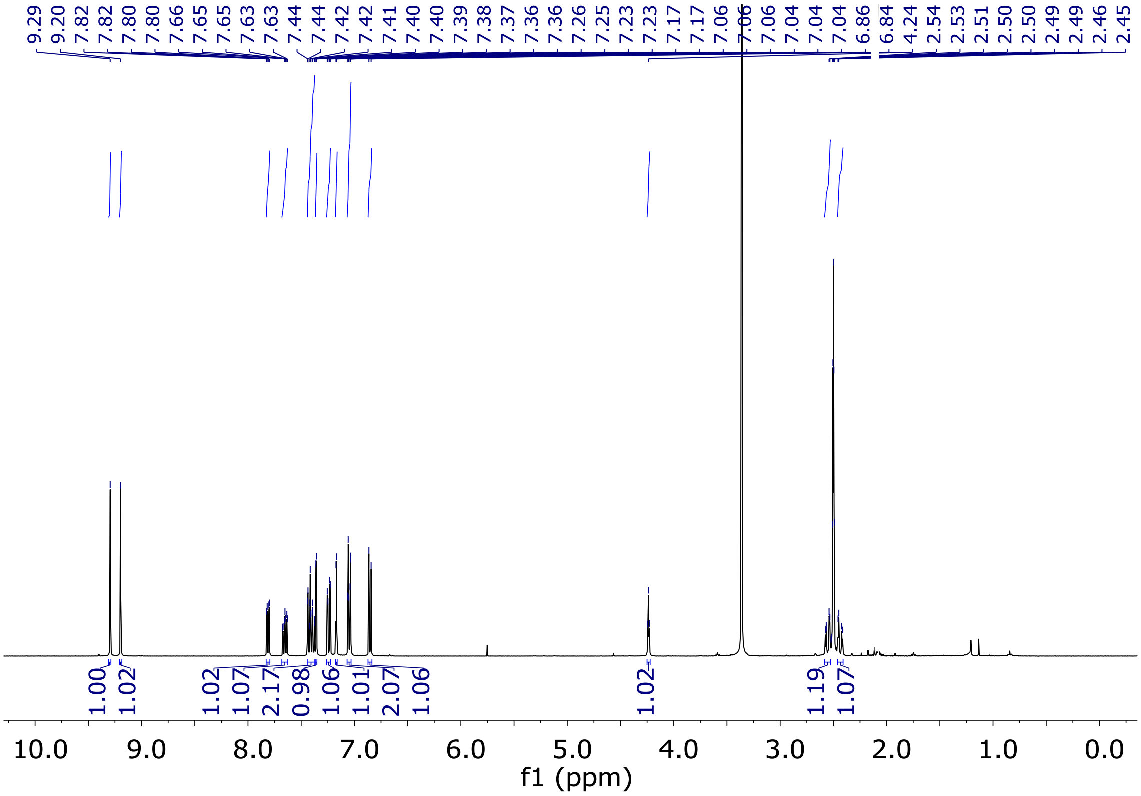

Figure S17.^{1}H-NMR spectrum of compound **47** in DMSO-d_6

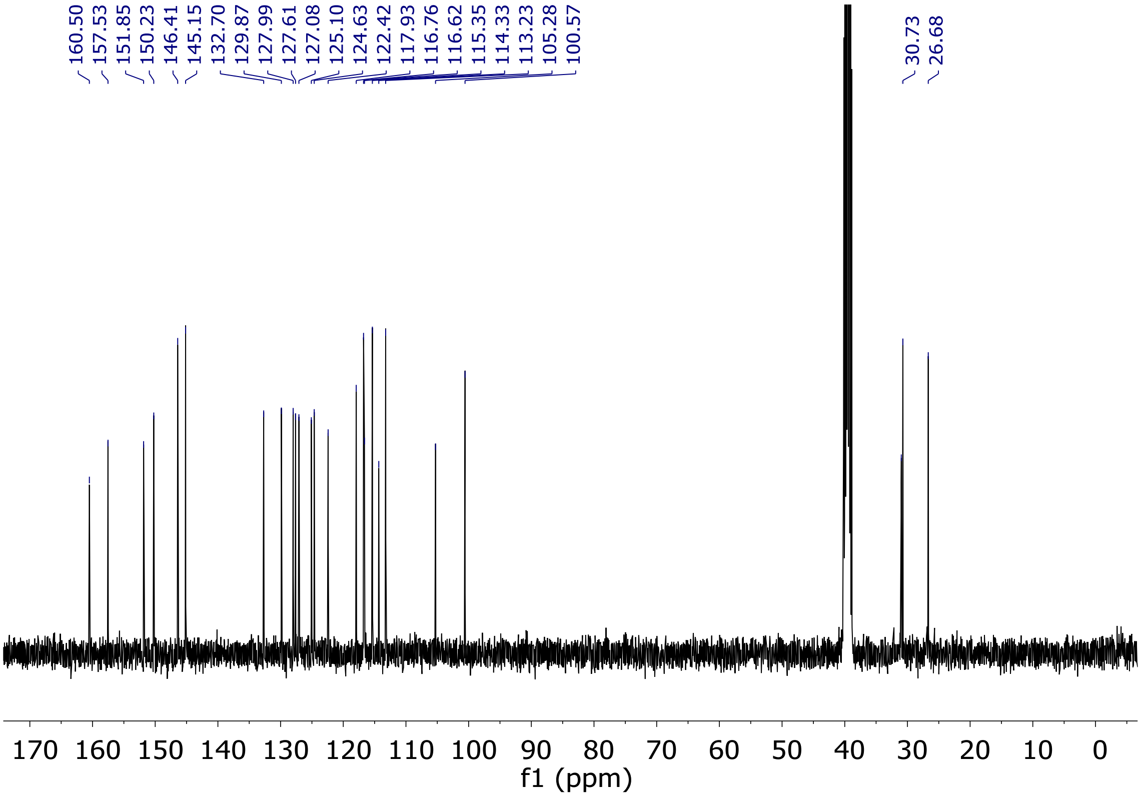

Figure S18.^{13}C-NMR spectrum of compound **47** in DMSO-d_6

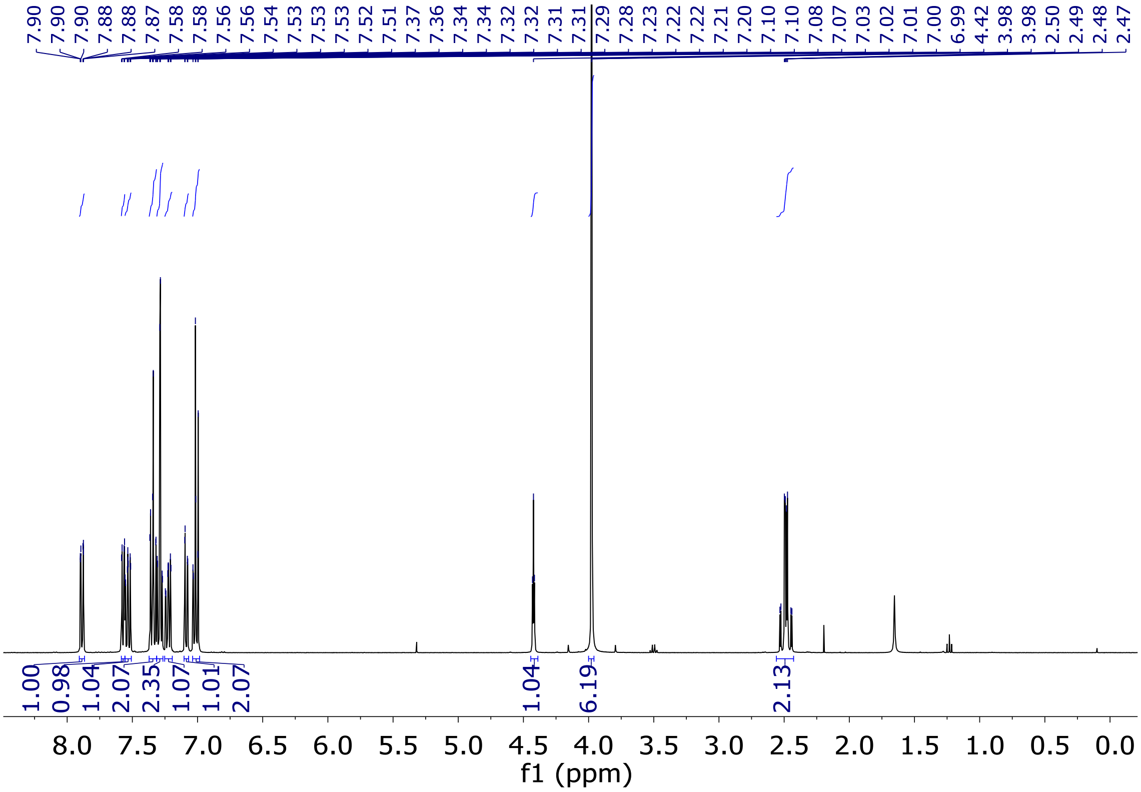

Figure S19.^{1}H-NMR spectrum of compound **48** in $CDCl_3$

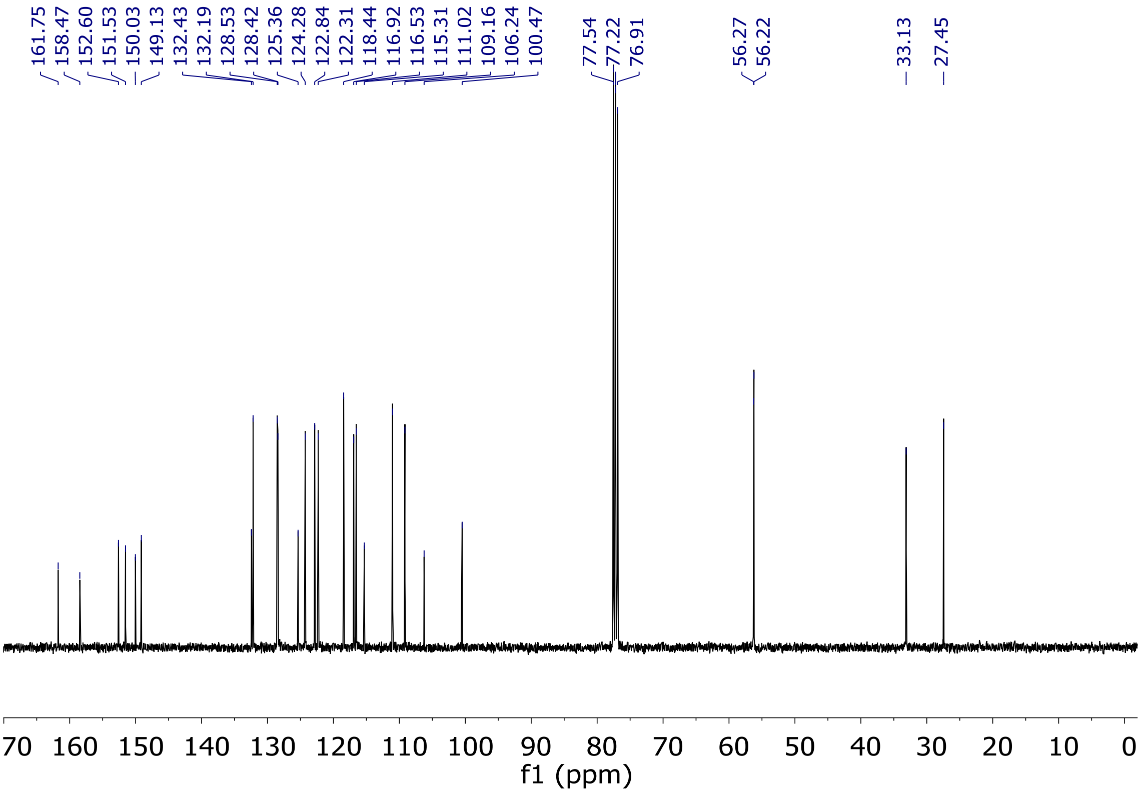

Figure S20.^{13}C-NMR spectrum of compound **48** in $CDCl_3$

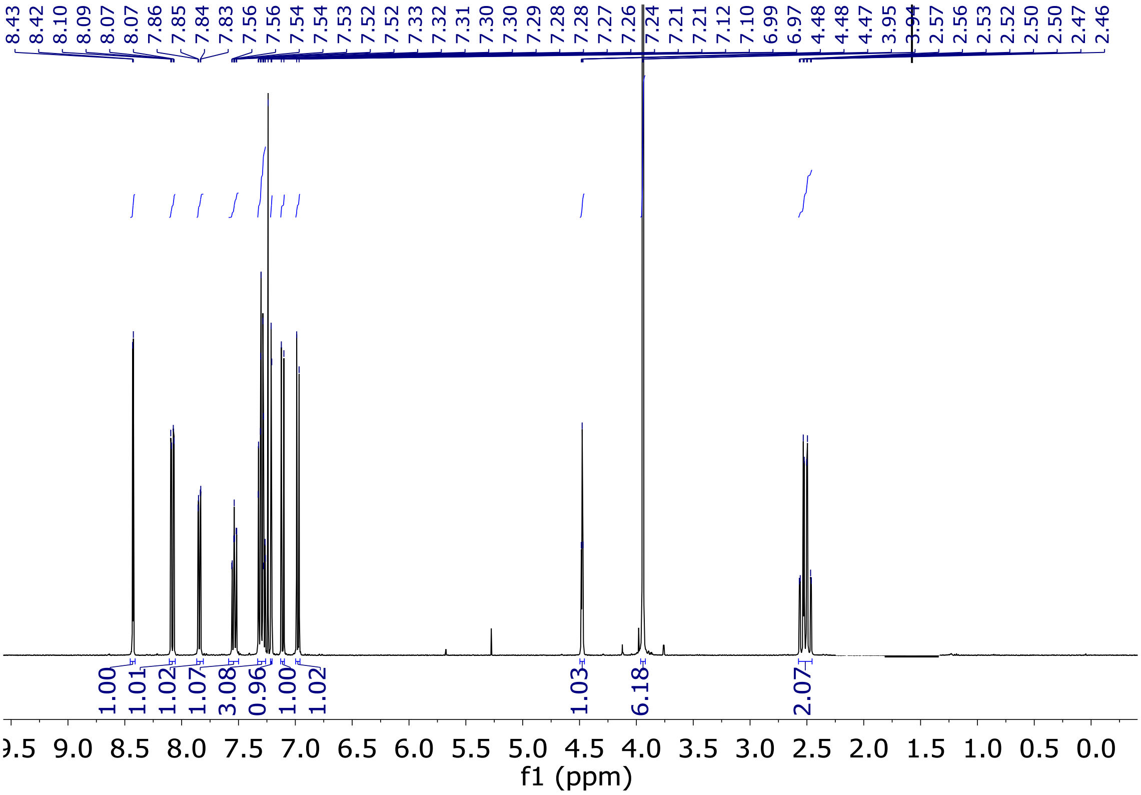

Figure S21.^{1}H-NMR spectrum of compound **49** in $CDCl_3$

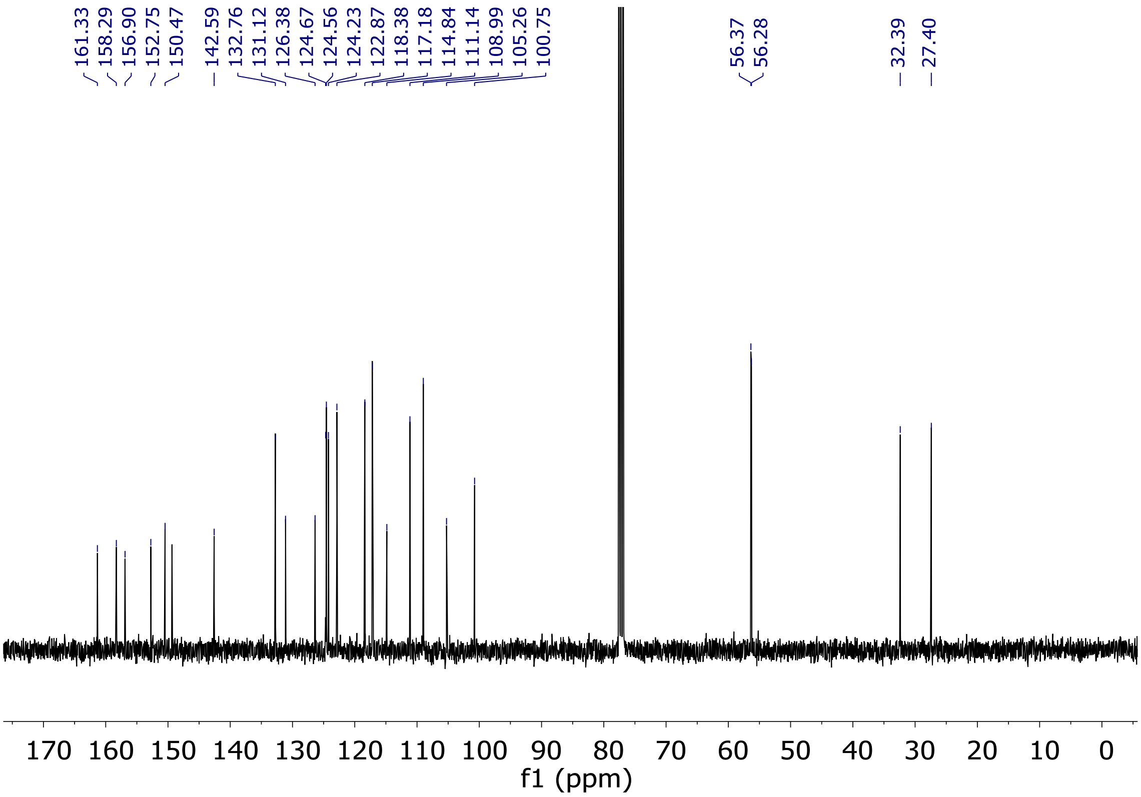

Figure S22.^{13}C-NMR spectrum of compound **49** in $CDCl_3$

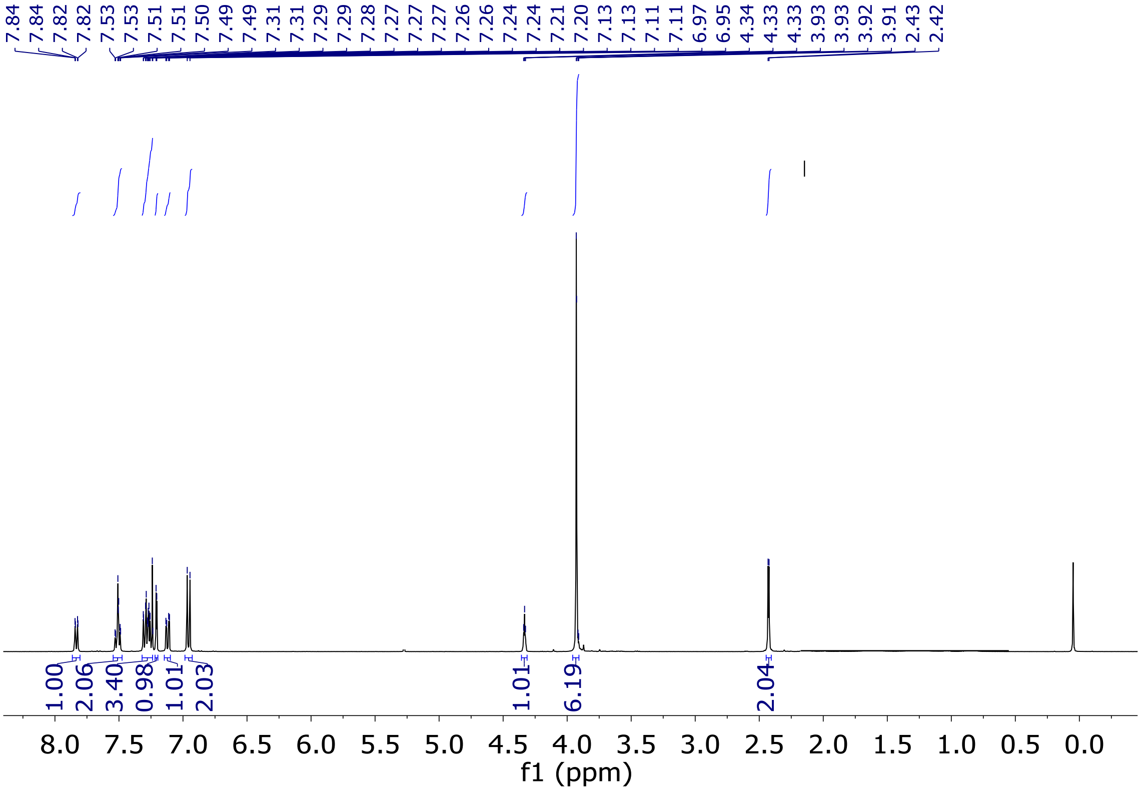

Figure S23.^{1}H-NMR spectrum of compound **50** in $CDCl_3$.

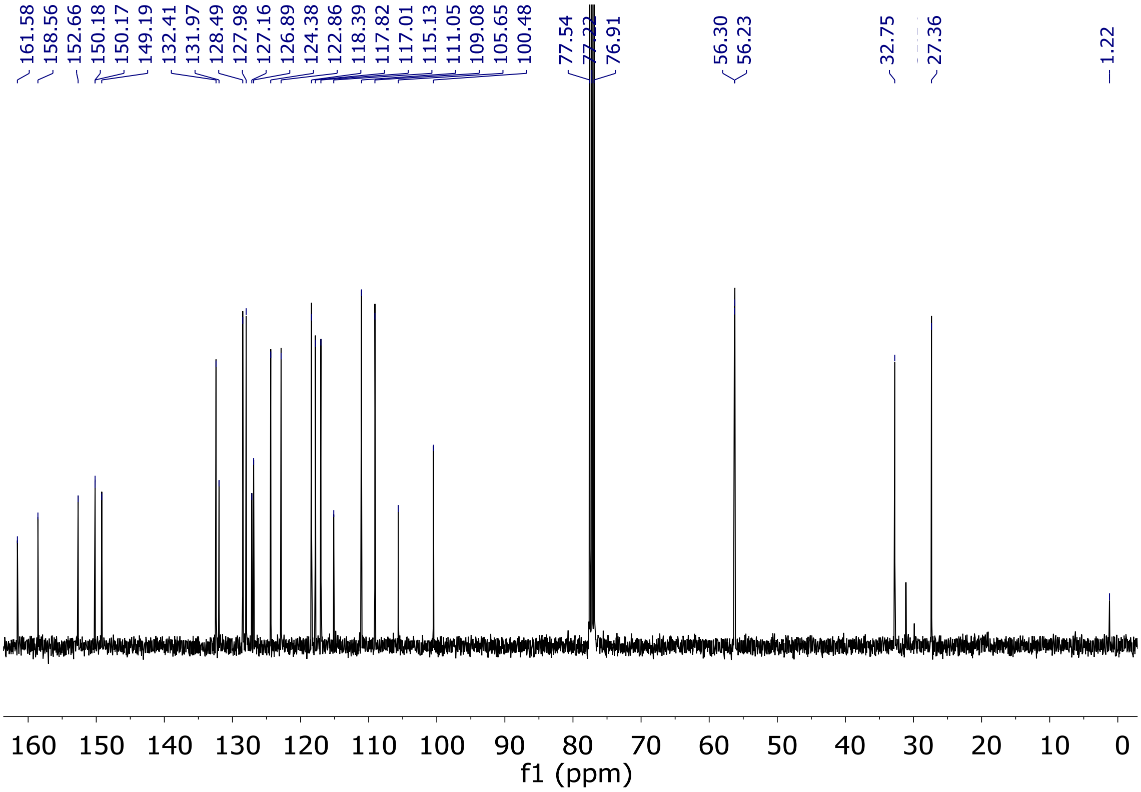

Figure S24.^{13}C-NMR spectrum of compound **50** in $CDCl_3$.

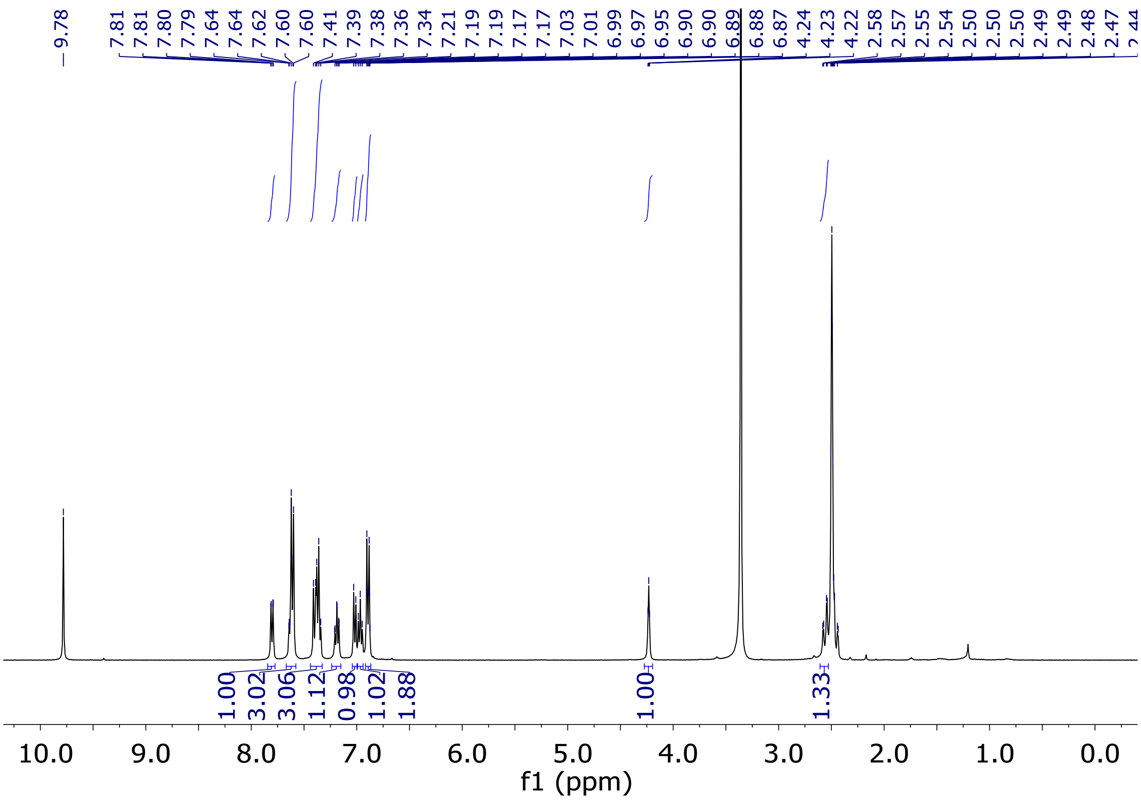

Figure S25.^{1}H-NMR spectrum of compound **51** in DMSO-d_6

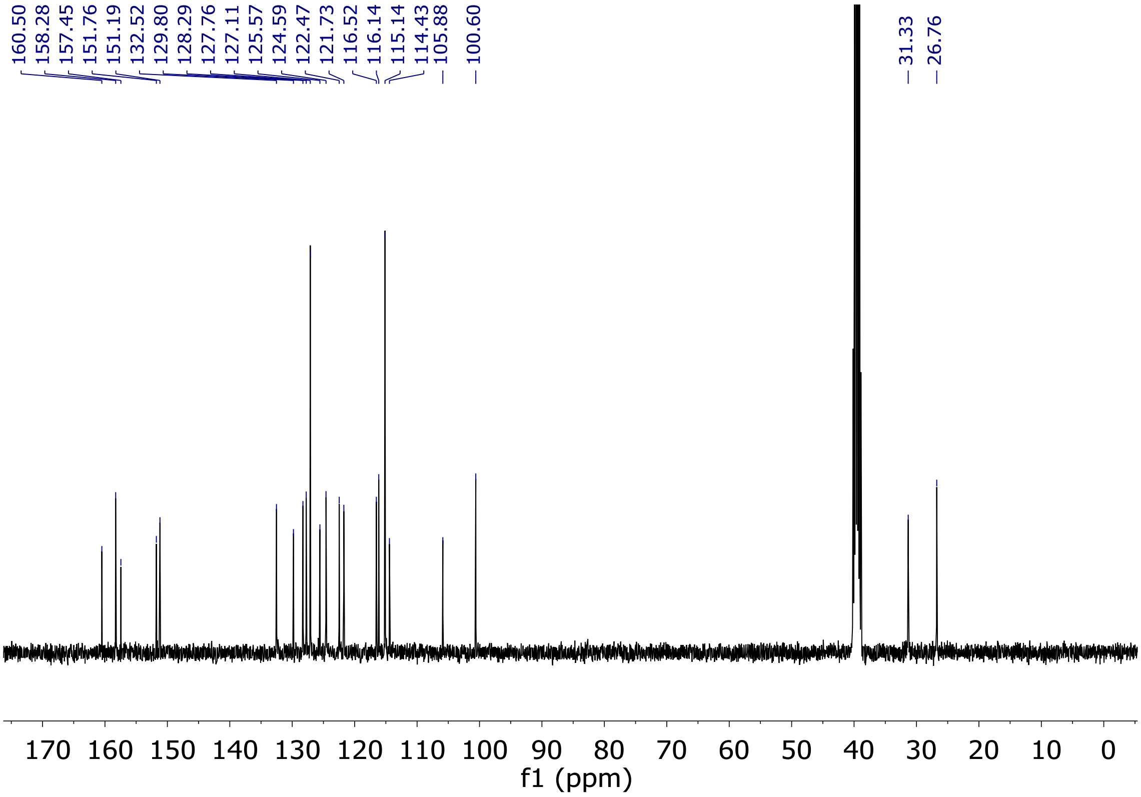

Figure S26.^{13}C-NMR spectrum of compound **51** in DMSO-d_6

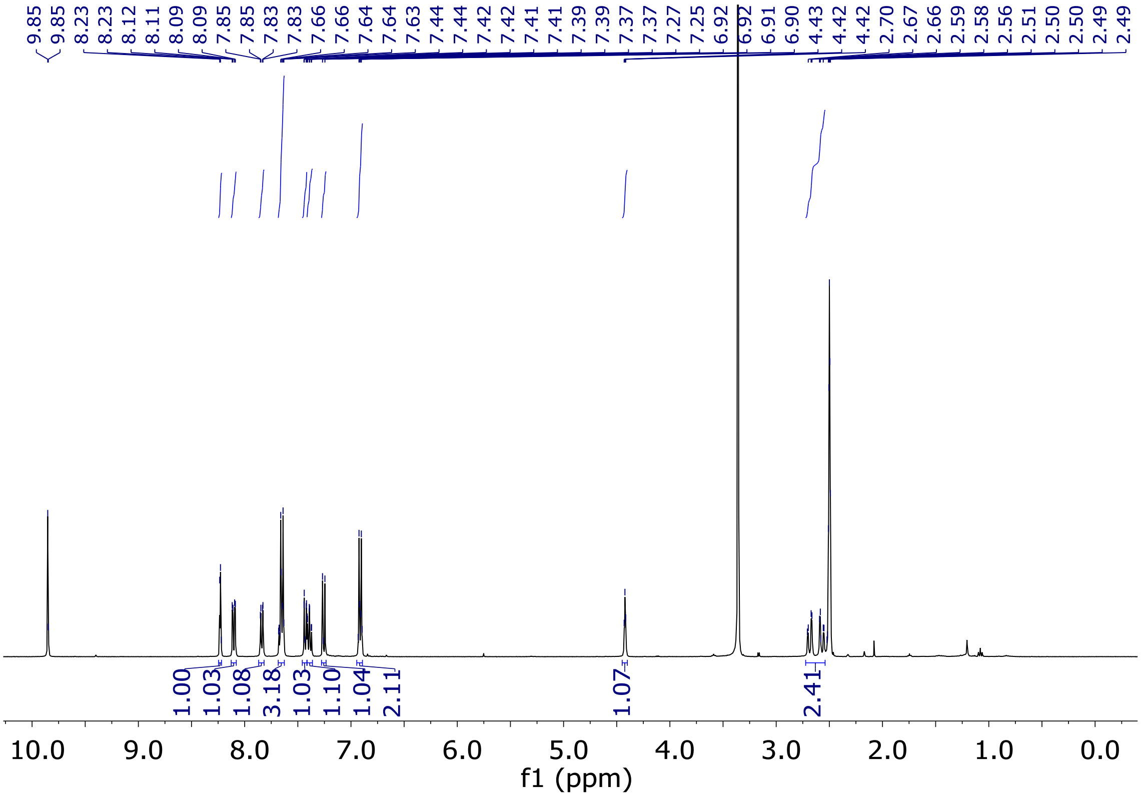

Figure S27.^{1}H-NMR spectrum of compound **52** in DMSO-d_6

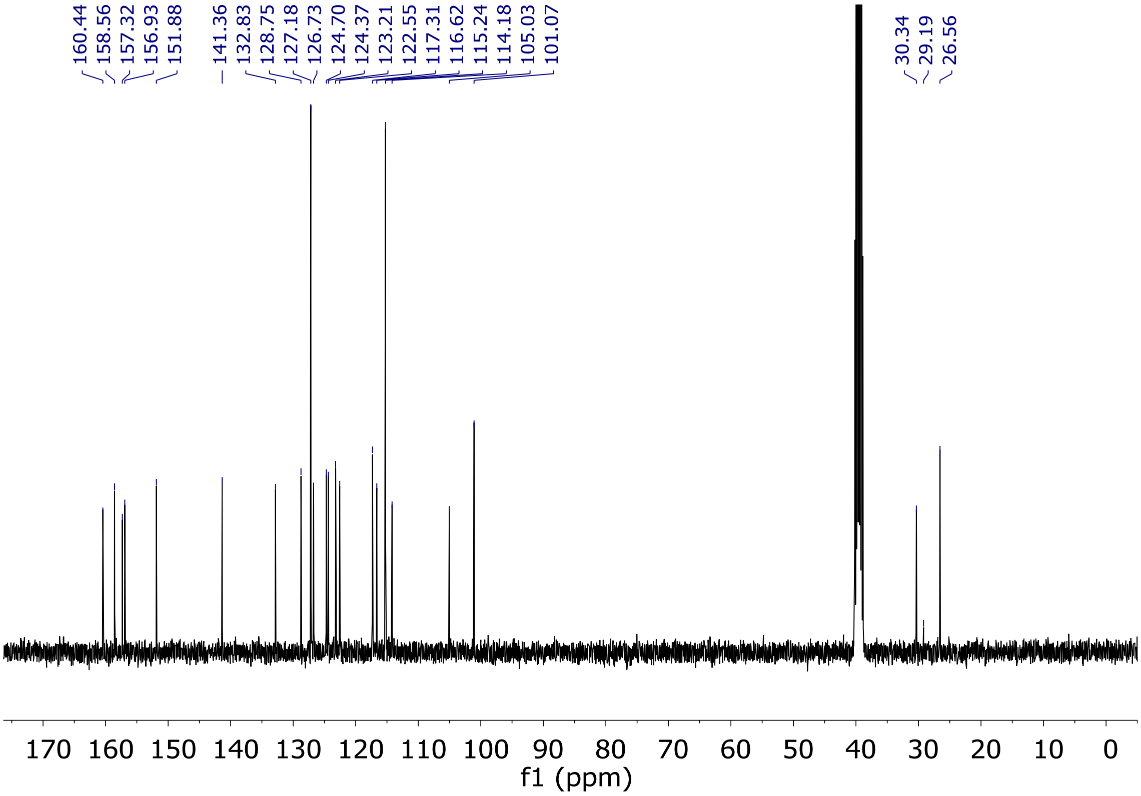

Figure S28.^{13}C-NMR spectrum of compound **52** in DMSO-d_6

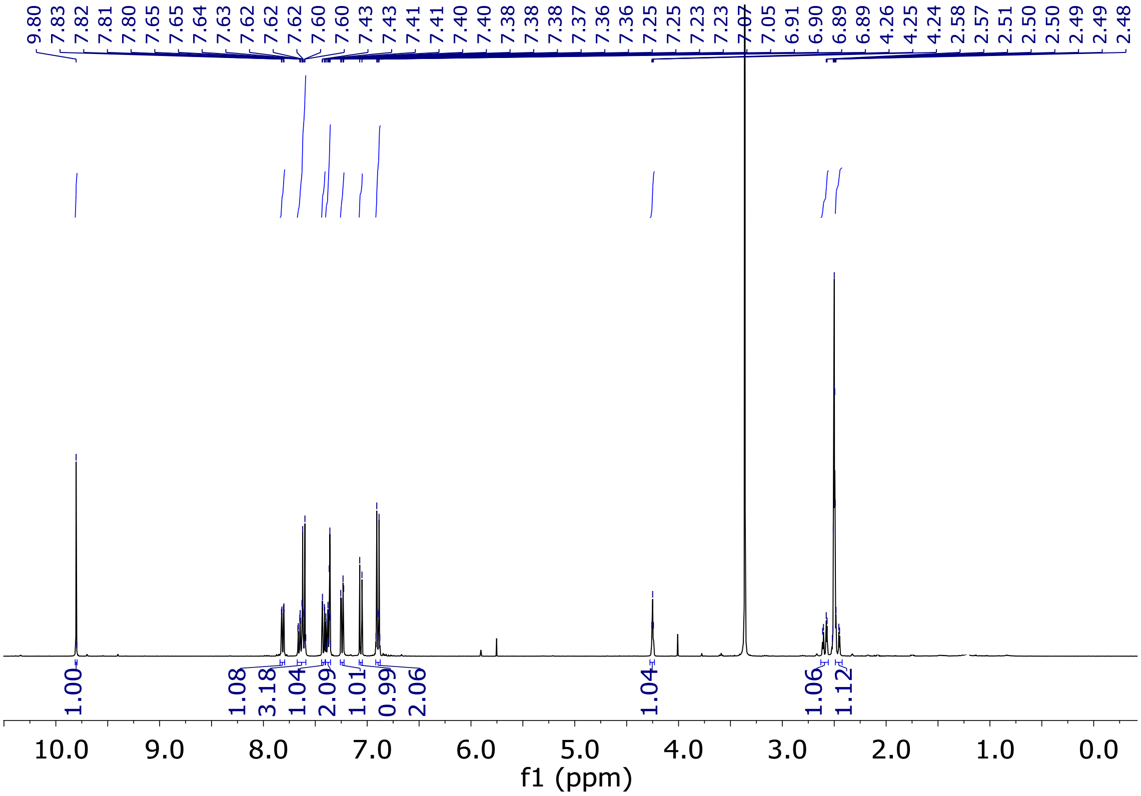

Figure S29.¹H-NMR spectrum of compound **53** in DMSO-d_6

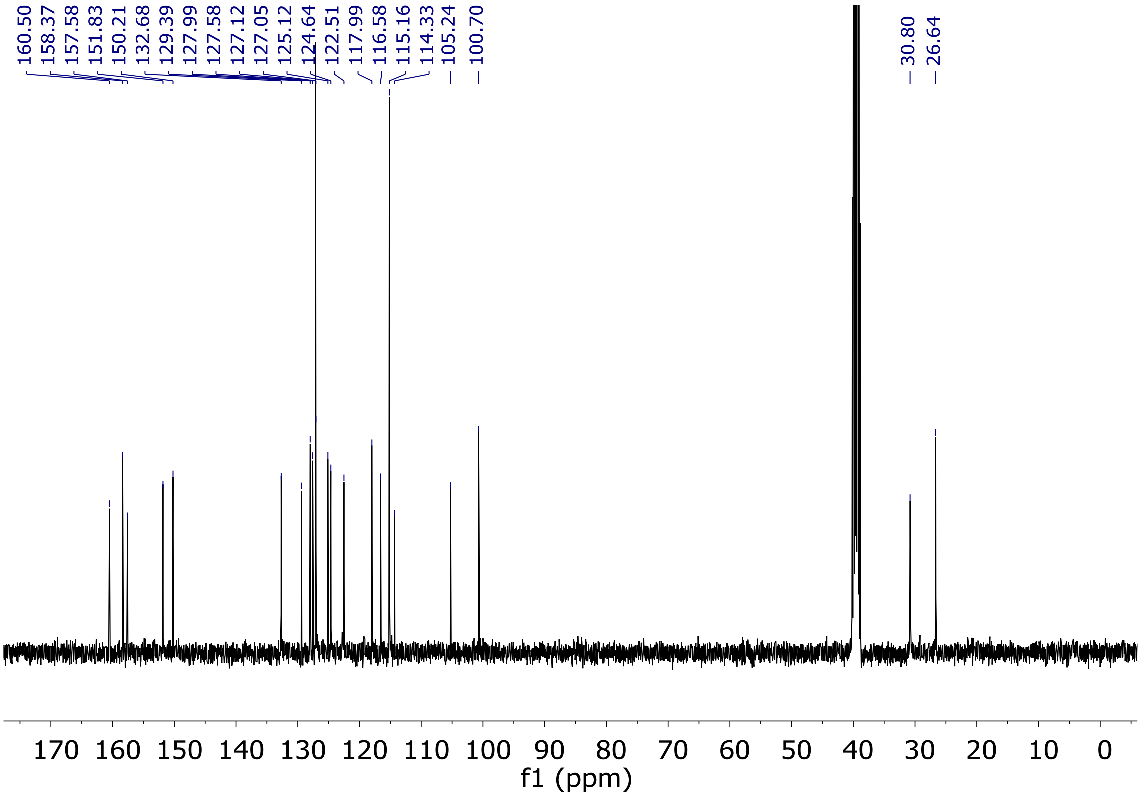

Figure S30.¹³C-NMR spectrum of compound **53** in DMSO-d_6

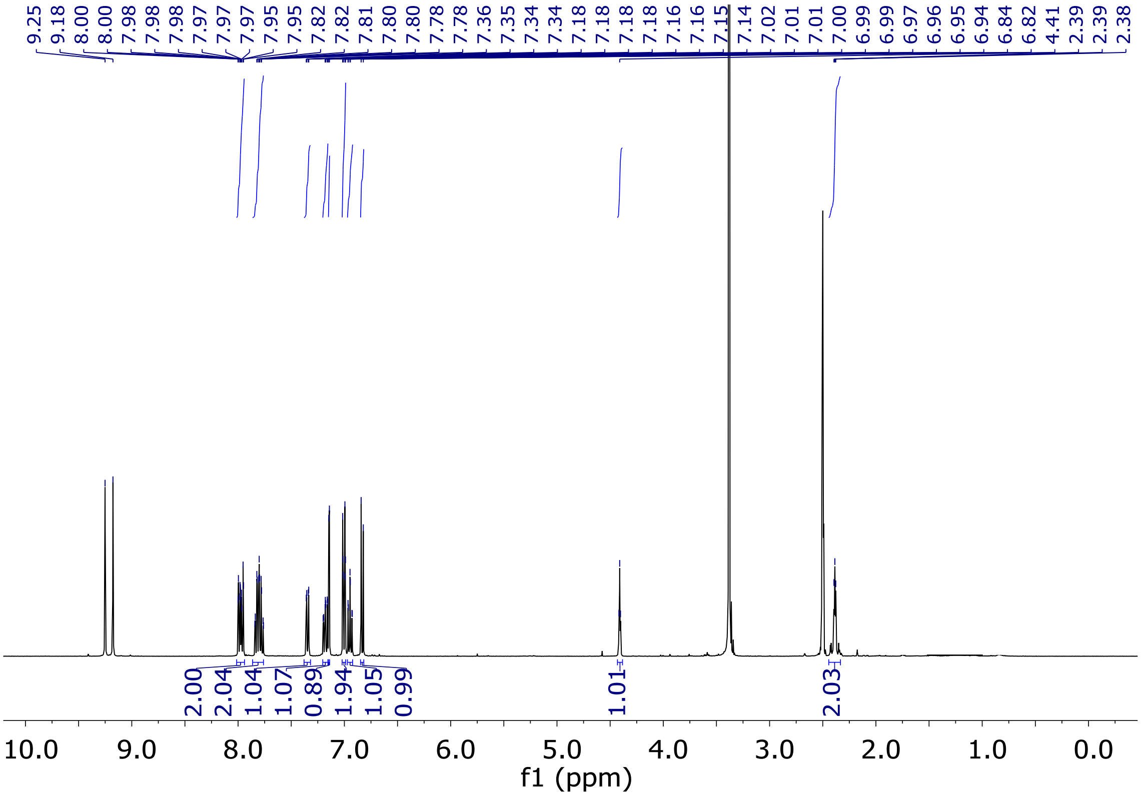

Figure S31.^{1}H-NMR spectrum of compound **54** in DMSO-d_6

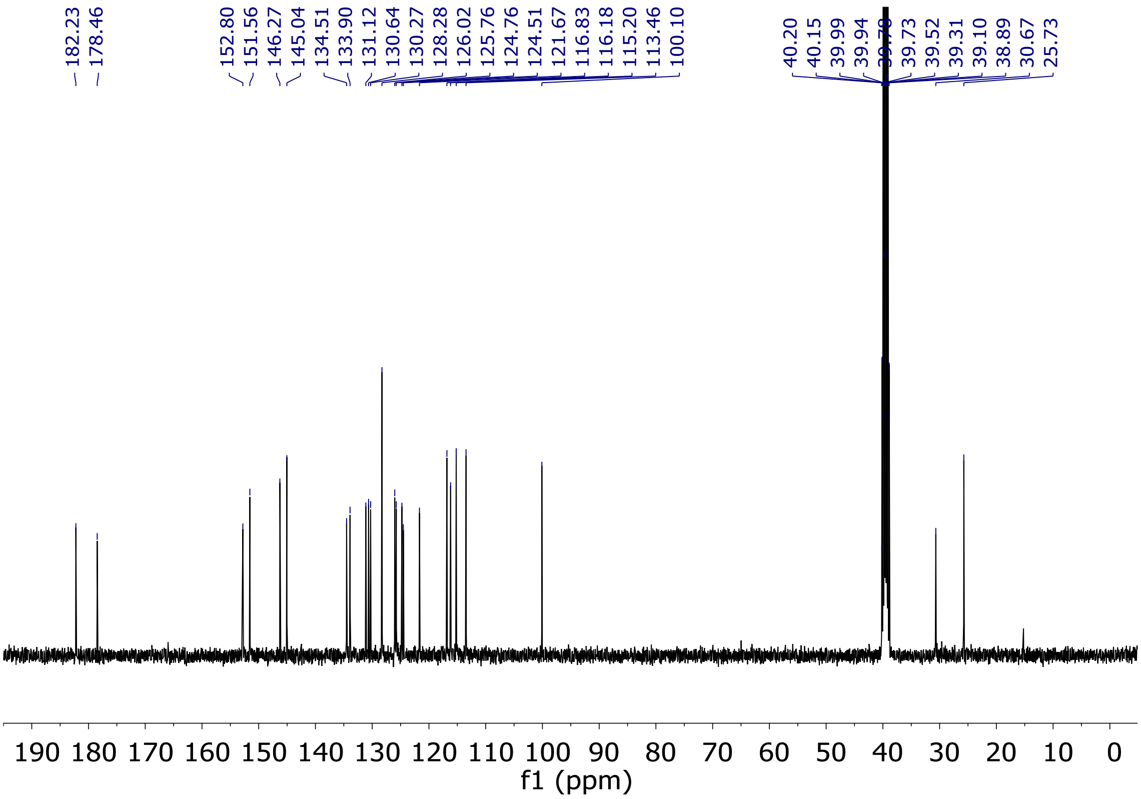

Figure S32.^{13}C-NMR spectrum of compound **54** in DMSO-d_6

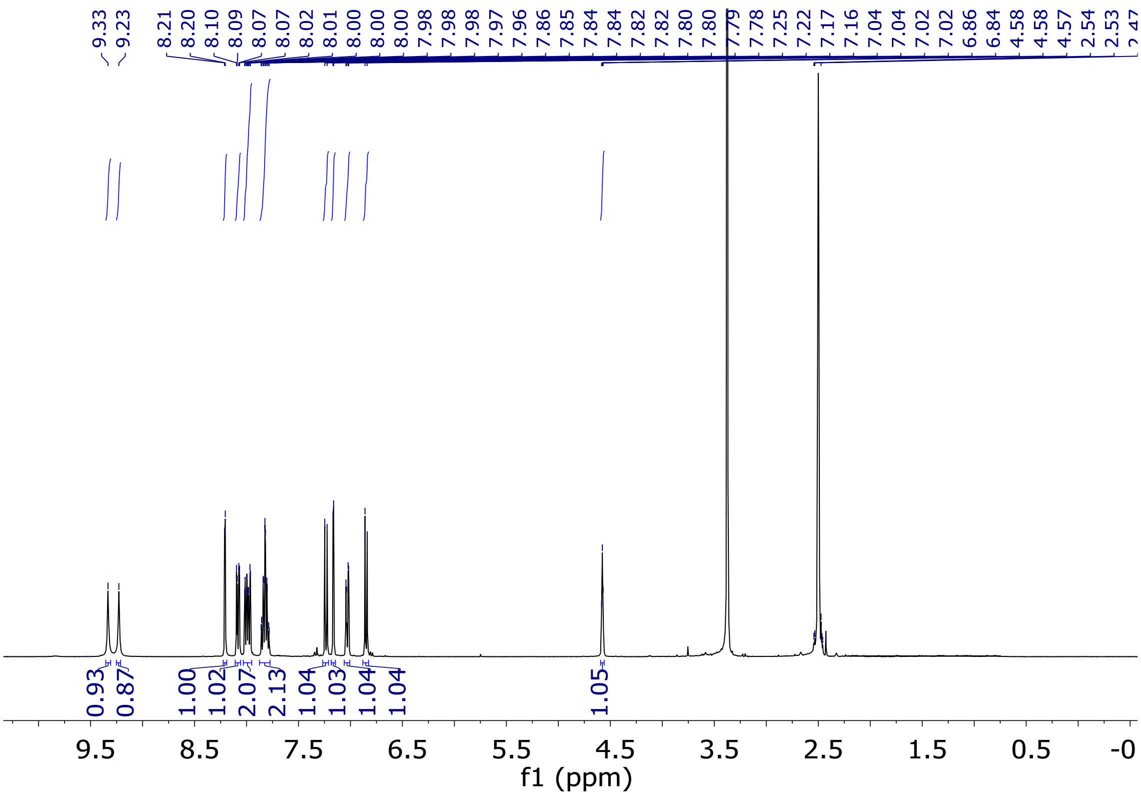

Figure S33.^{1}H-NMR spectrum of compound **55** in DMSO-d_6

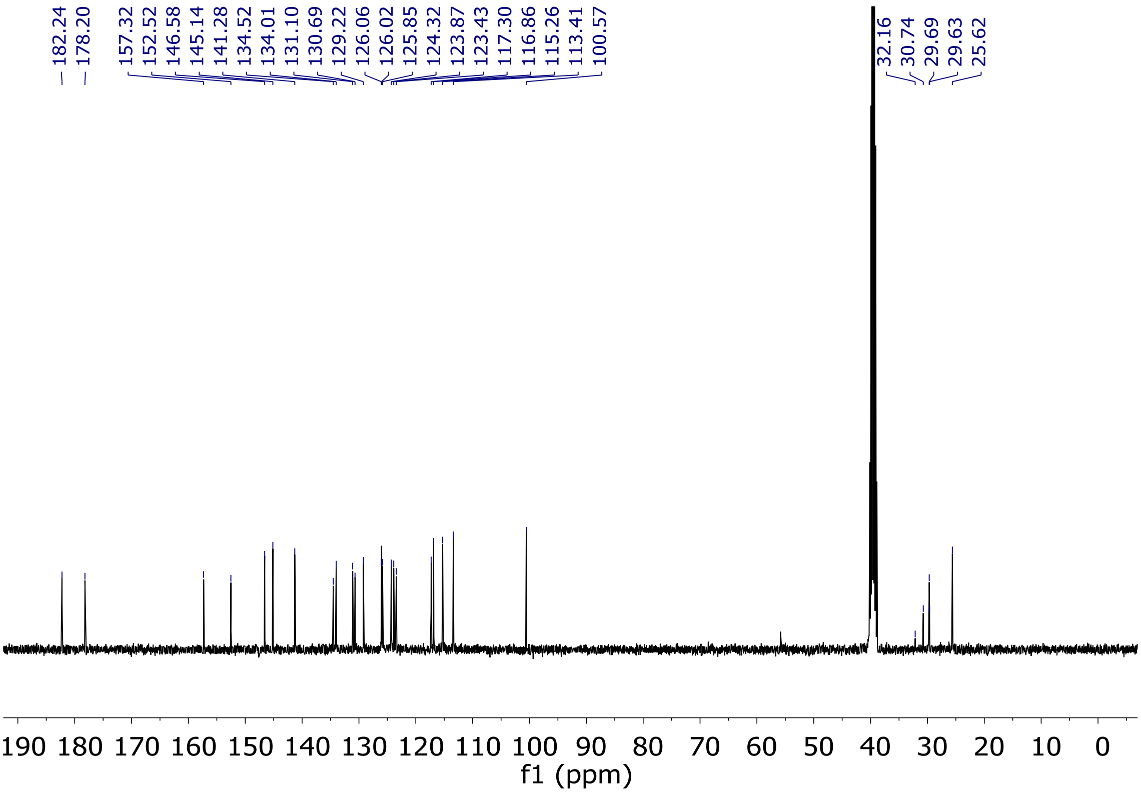

Figure S34.^{13}C-NMR spectrum of compound **55** in DMSO-d_6

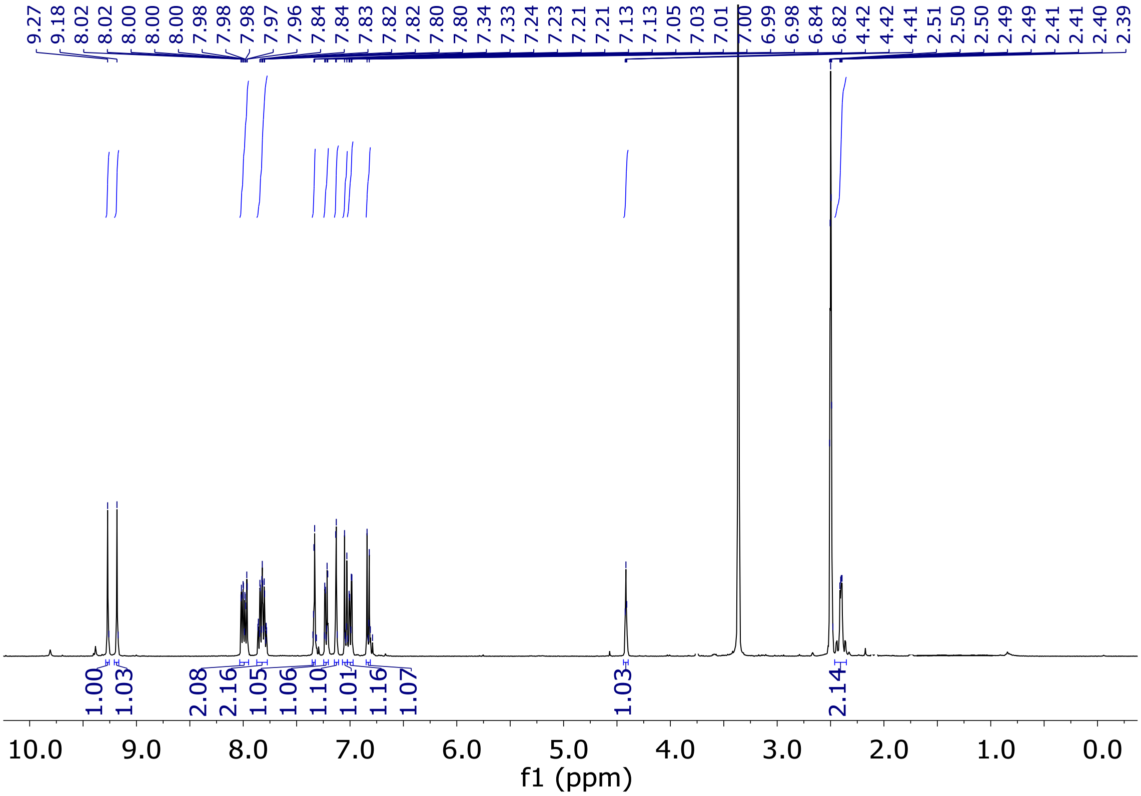

Figure S35.^{1}H-NMR spectrum of compound **56** in DMSO-d_6

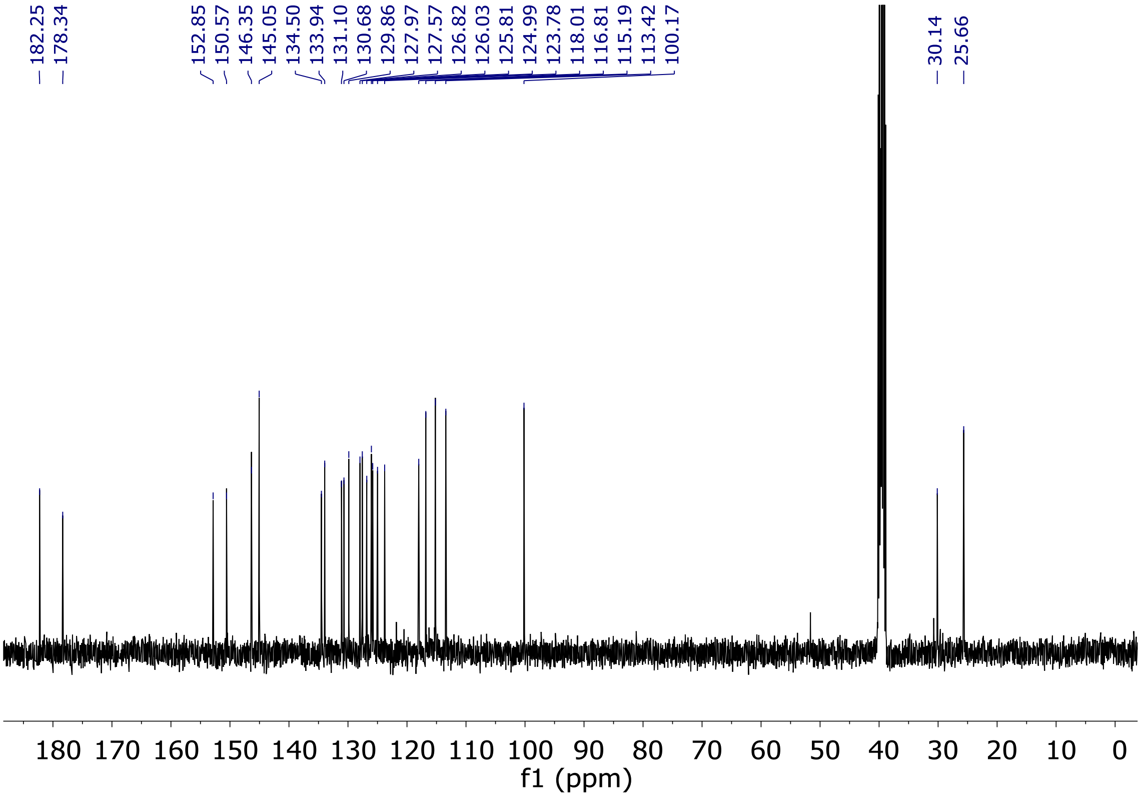

Figure S36.^{13}C-NMR spectrum of compound **56** in DMSO-d_6

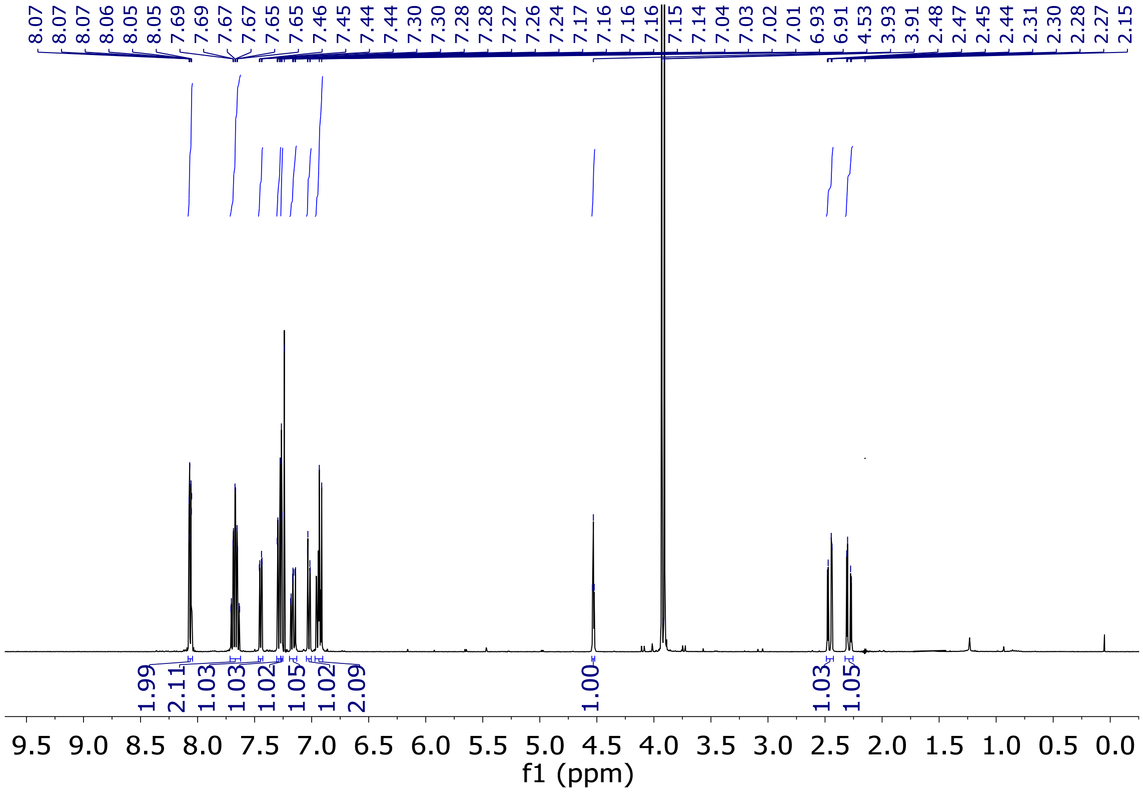

Figure S37.^{1}H-NMR spectrum of compound **57** in $CDCl_3$

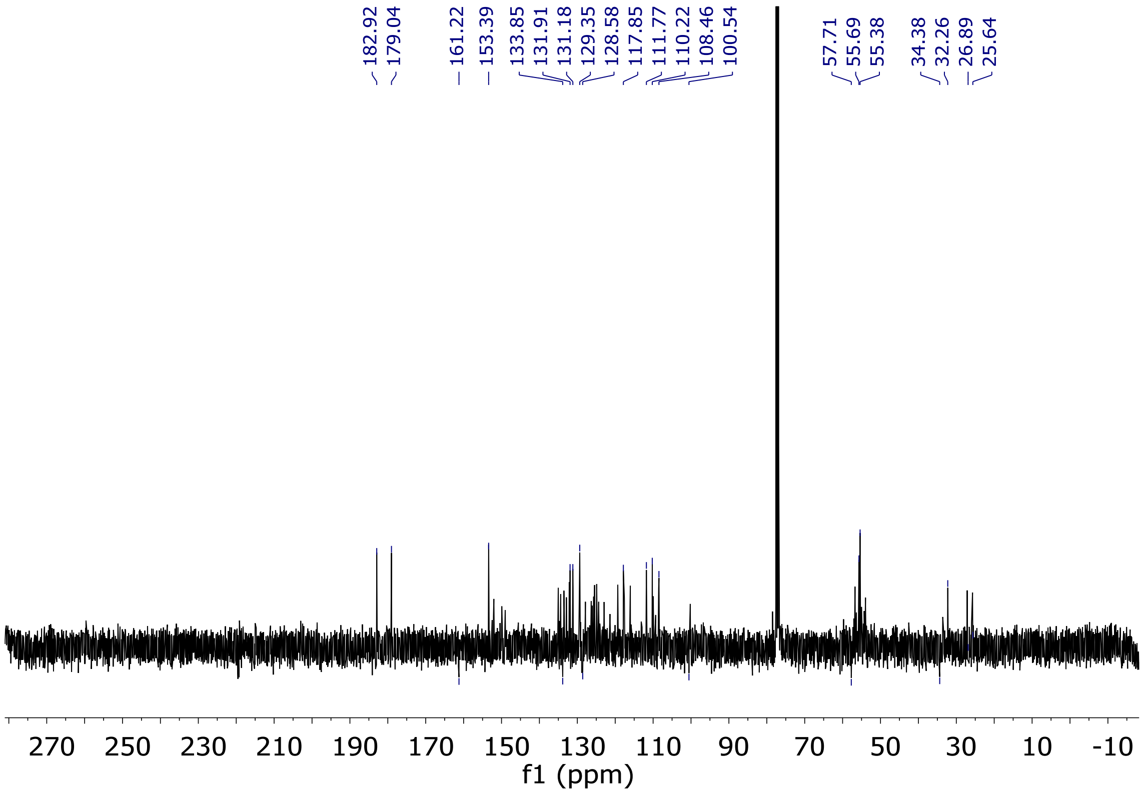

Figure S38.^{13}C-NMR spectrum of compound **57** in $CDCl_3$

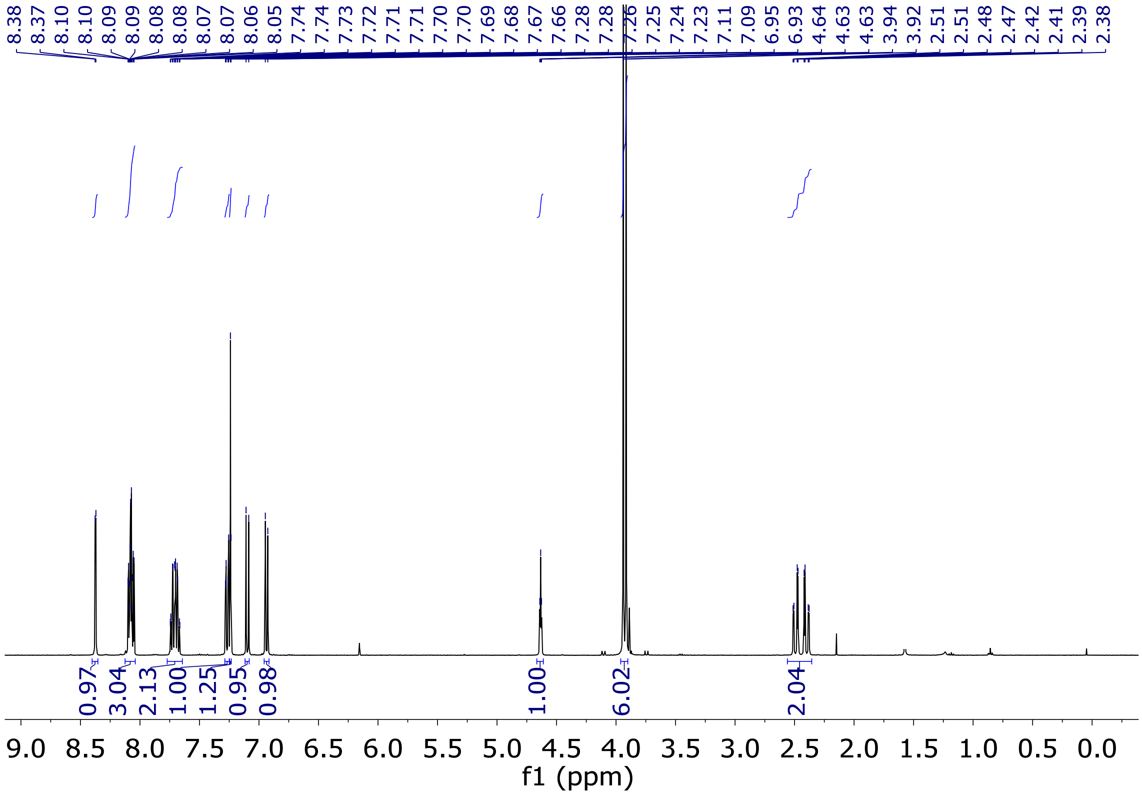

Figure S39.^{1}H-NMR spectrum of compound **58** in $CDCl_3$

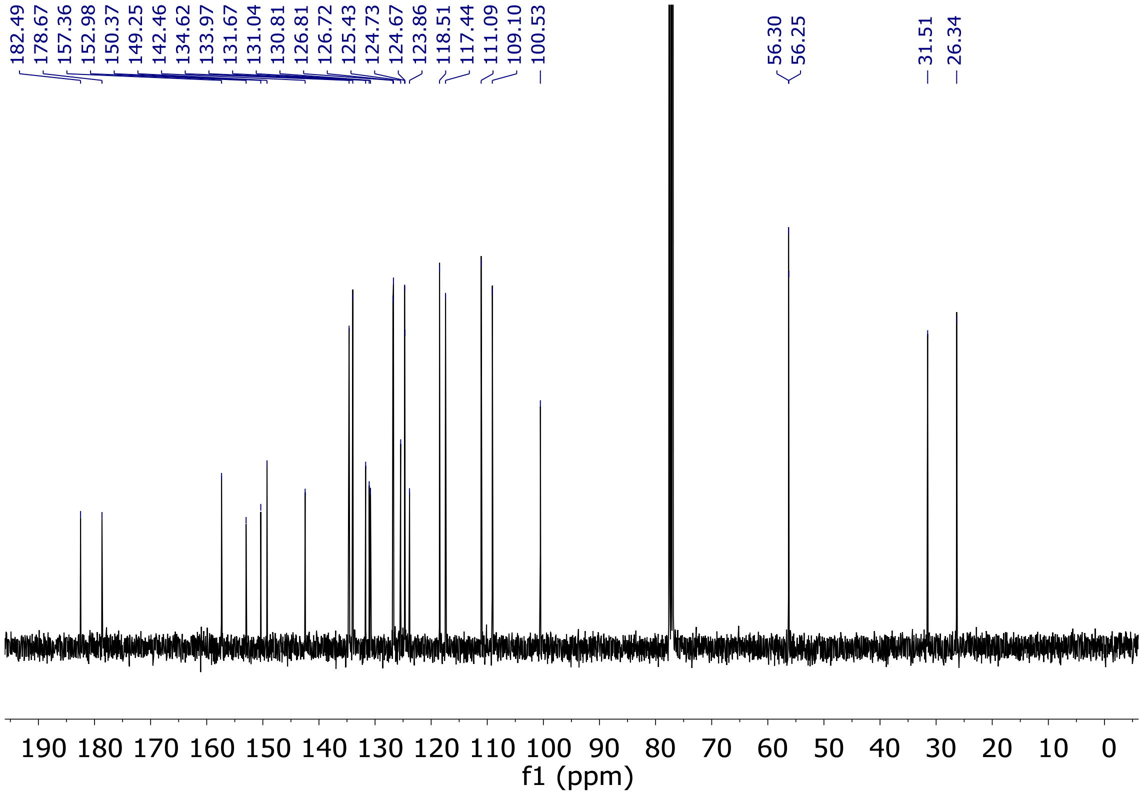

Figure S40.^{13}C-NMR spectrum of compound **58** in $CDCl_3$

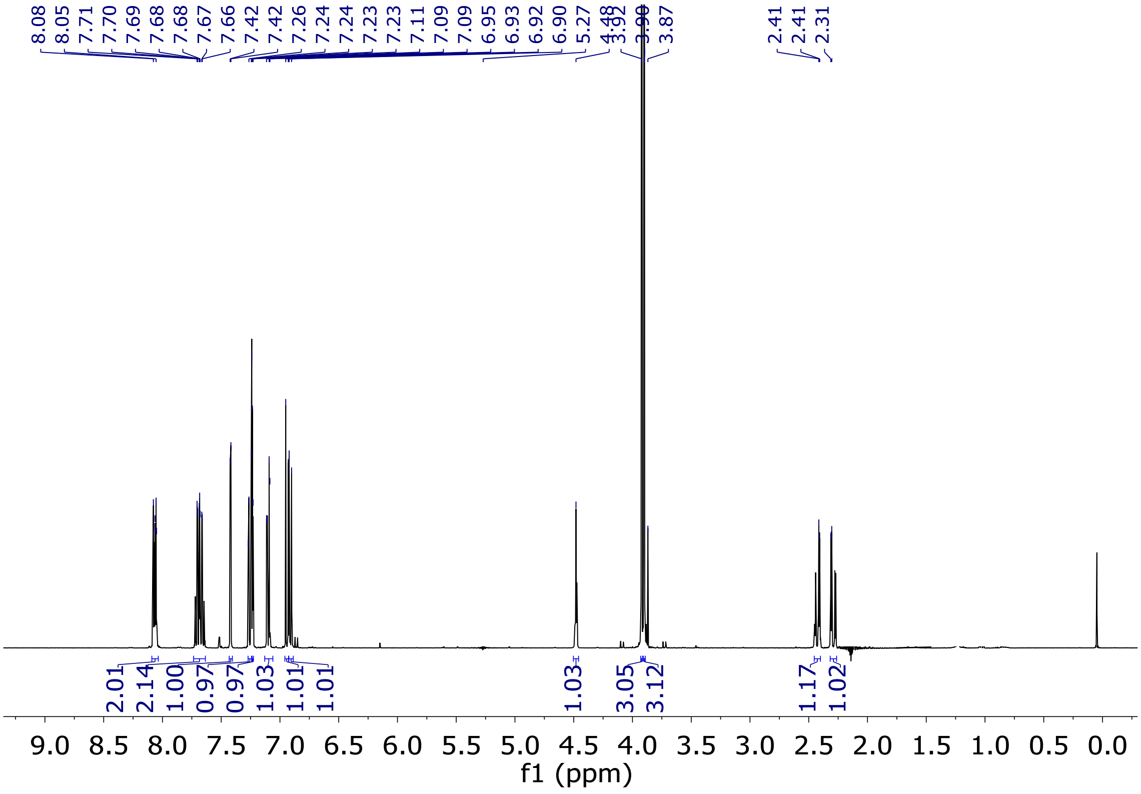

Figure S41.^{1}H-NMR spectrum of compound **59** in $CDCl_3$.

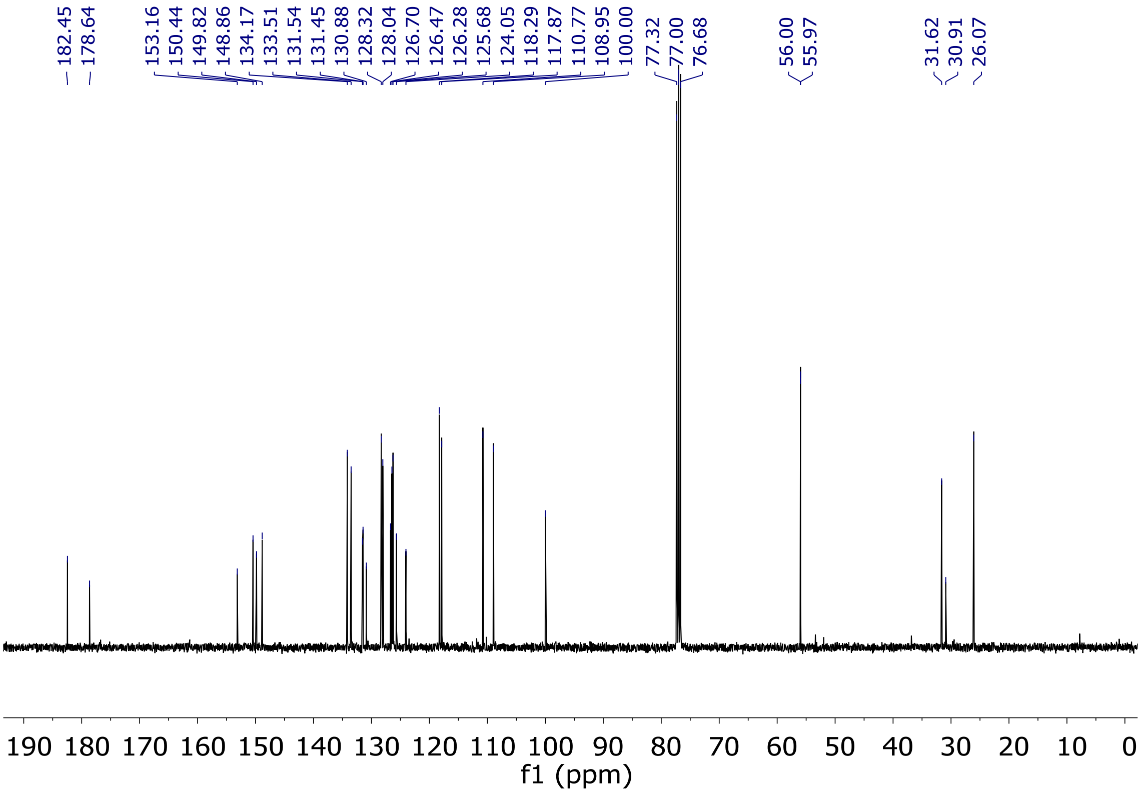

Figure S42.^{13}C-NMR spectrum of compound **59** in $CDCl_3$.

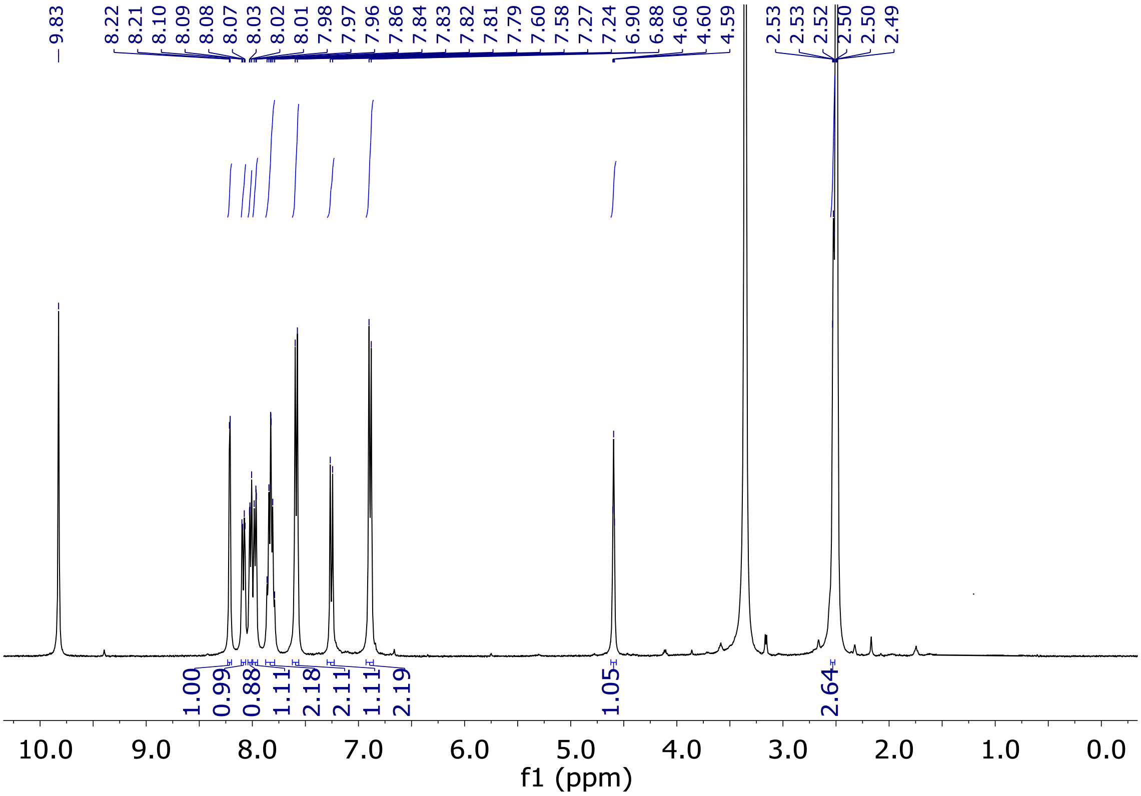

Figure S43.^{1}H-NMR spectrum of compound **60** in DMSO-d_6

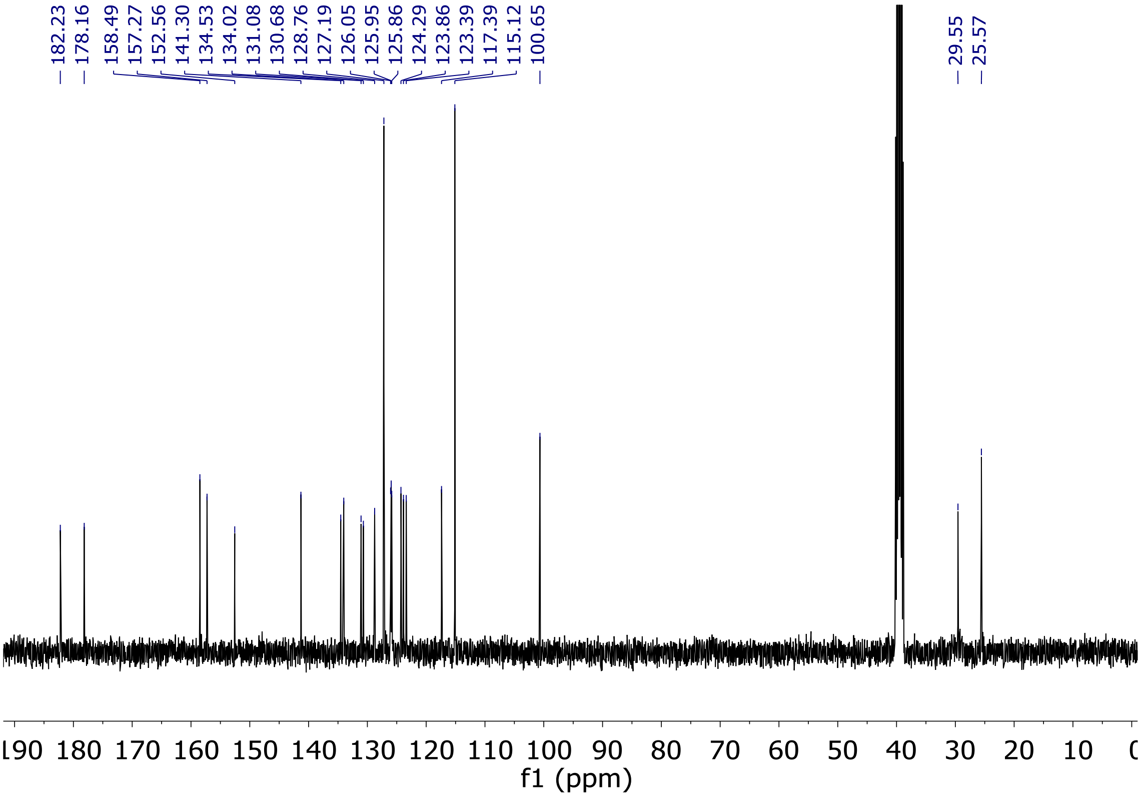

Figure S44.^{13}C-NMR spectrum of compound **60** in DMSO-d_6

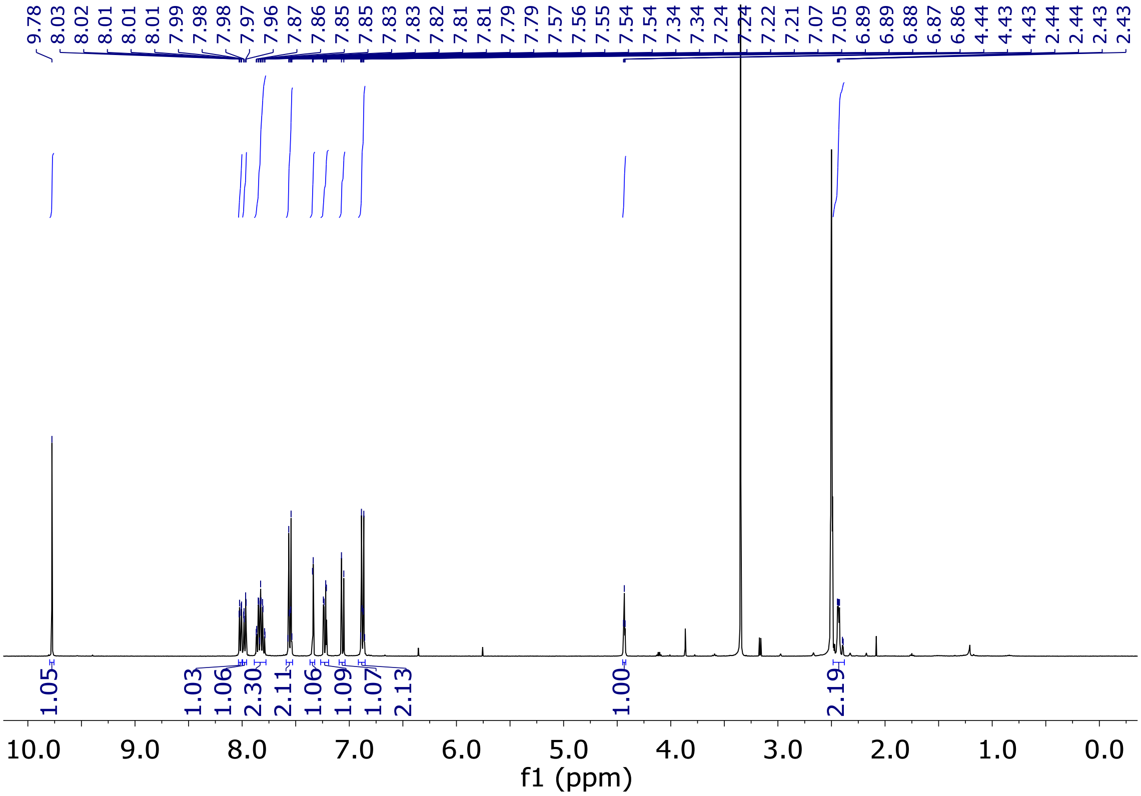

Figure S45.^{1}H-NMR spectrum of compound **61** in DMSO-d_6

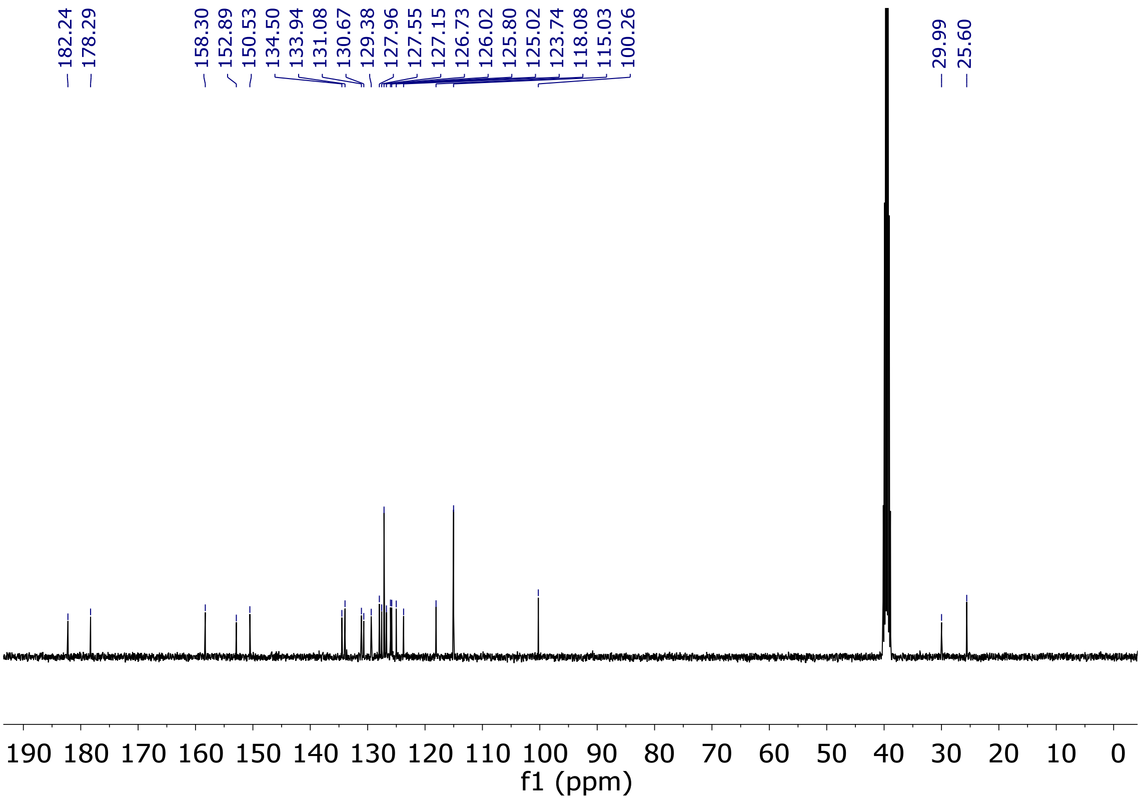

Figure S46.^{13}C-NMR spectrum of compound **61** in DMSO-d_6

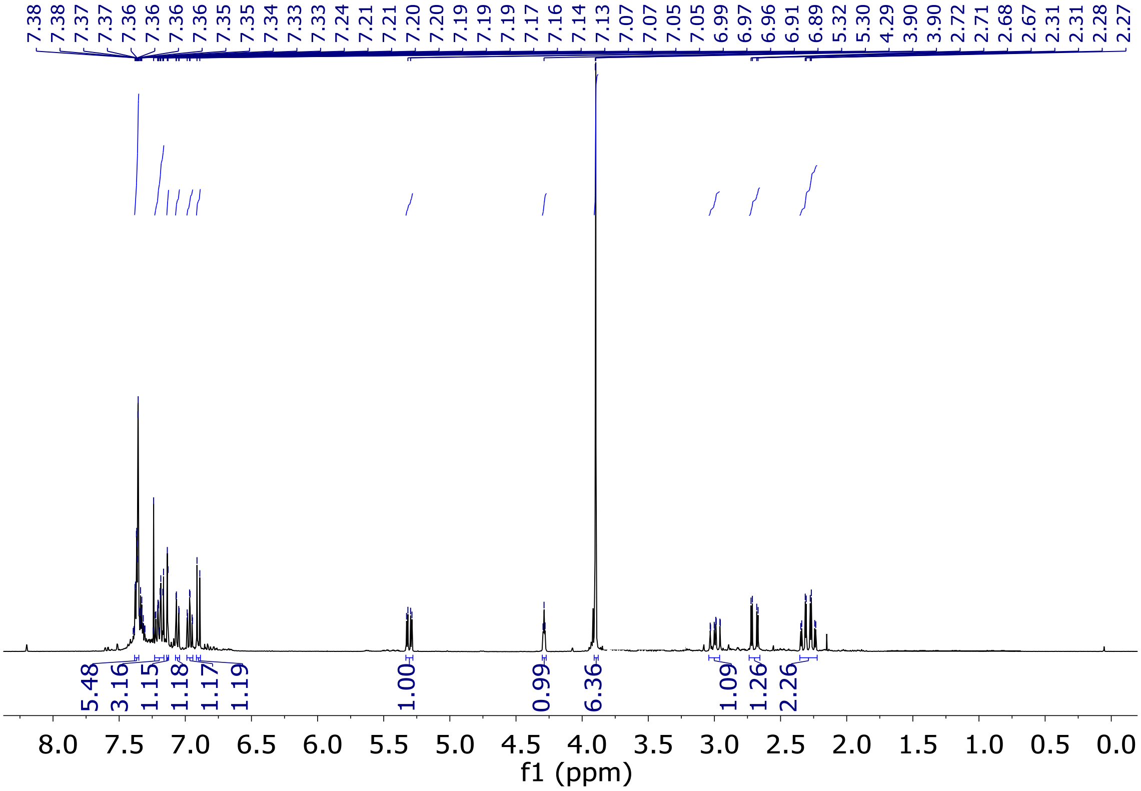

Figure S47.^{1}H-NMR spectrum of compound **62a** in $CDCl_3$

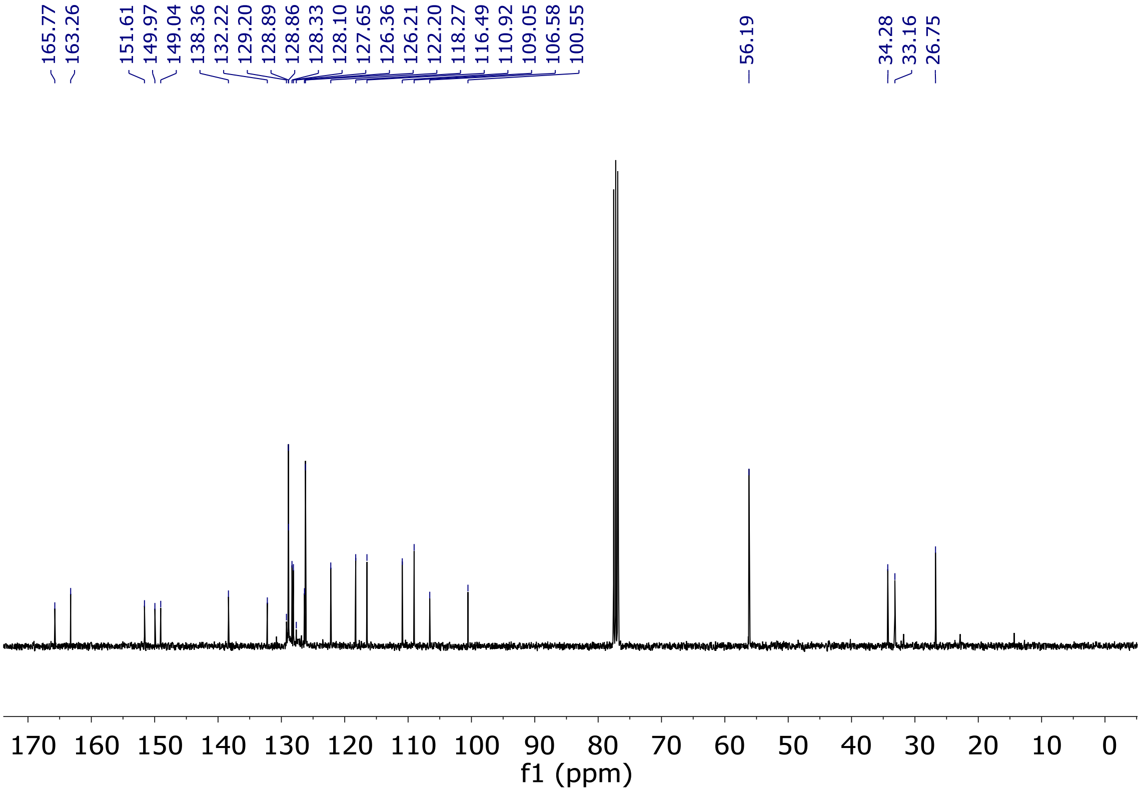

Figure S48.^{13}C-NMR spectrum of compound **62a** in $CDCl_3$

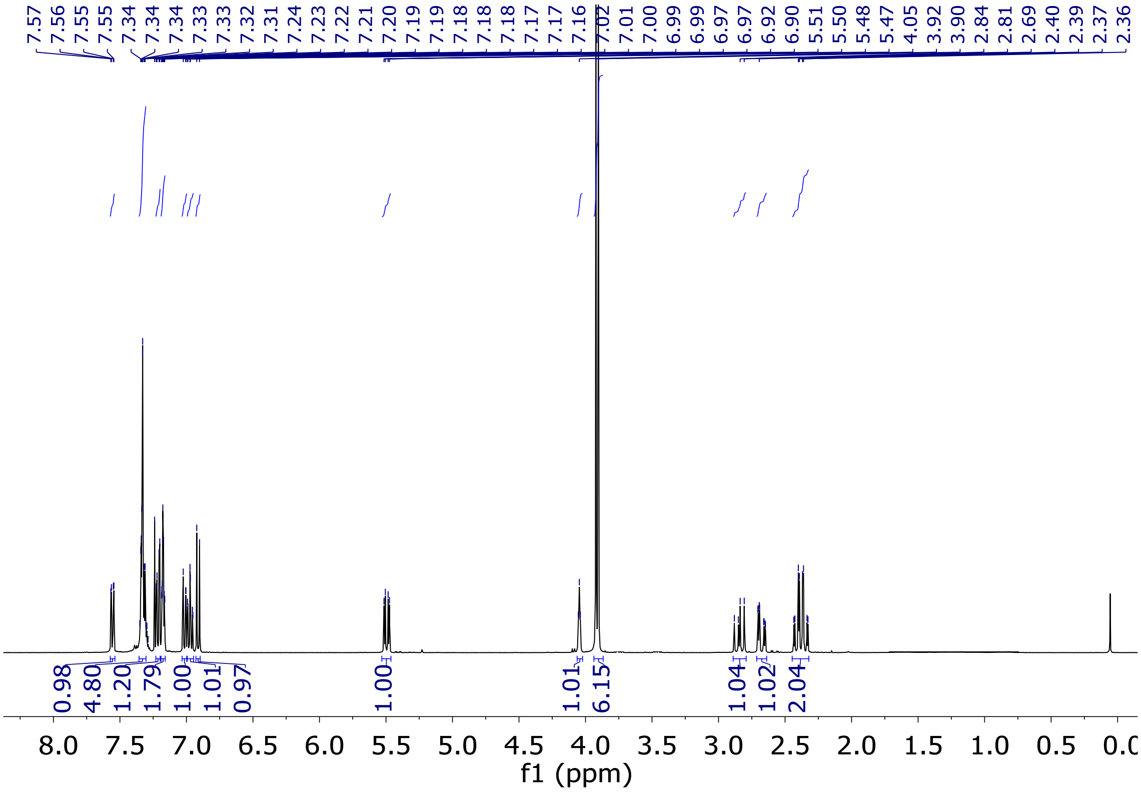

Figure S49.^{1}H-NMR spectrum of compound **62b** in $CDCl_3$

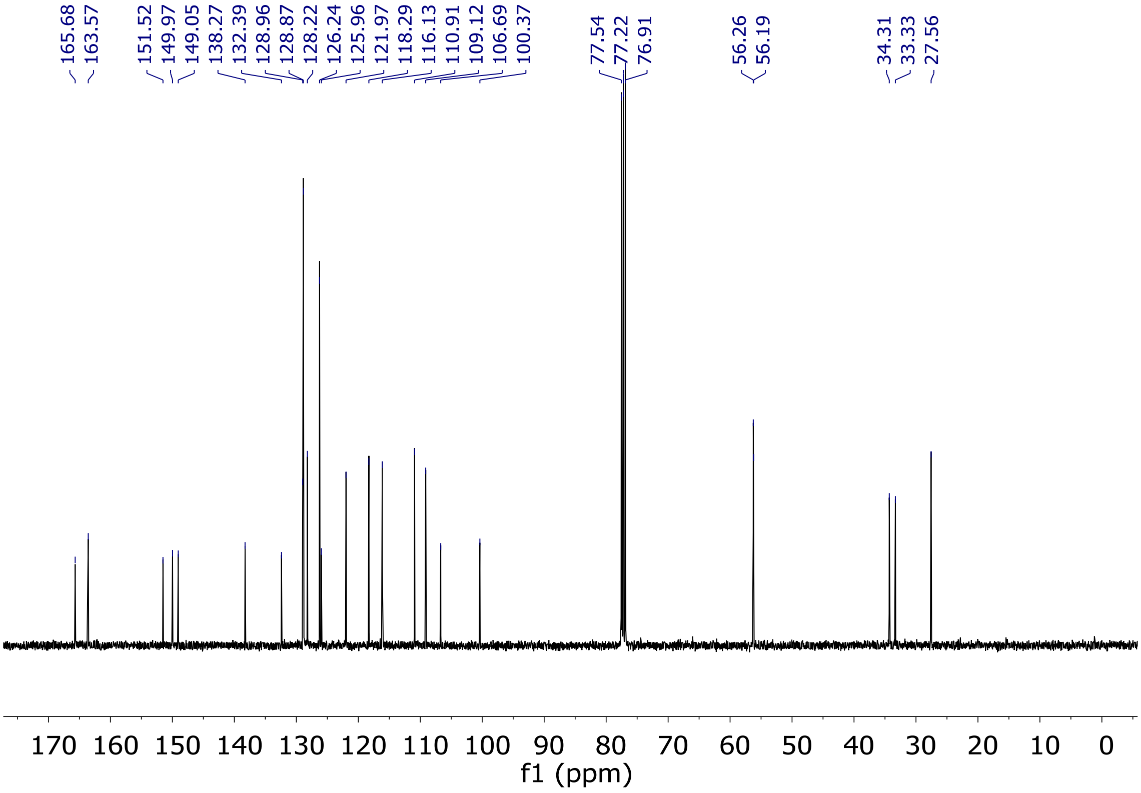

Figure S50.^{13}C-NMR spectrum of compound **62b** in $CDCl_3$

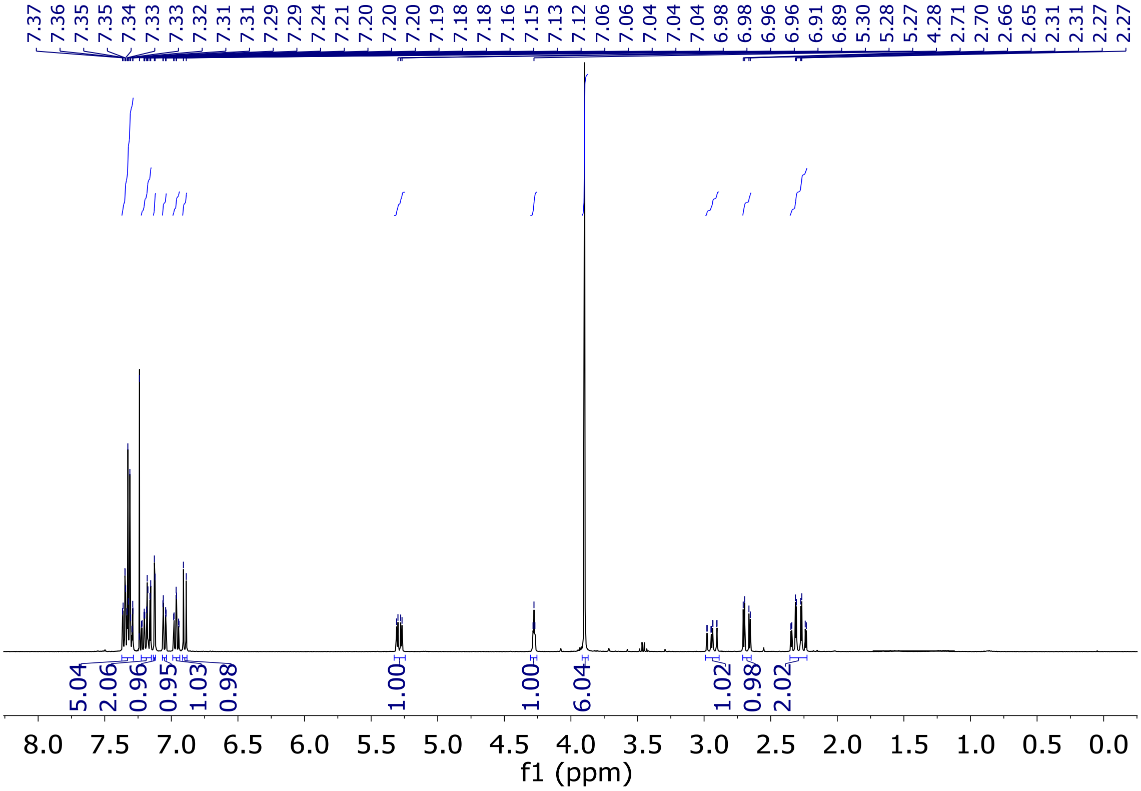

Figure S51.¹H-NMR spectrum of compound **63a** in $CDCl_3$

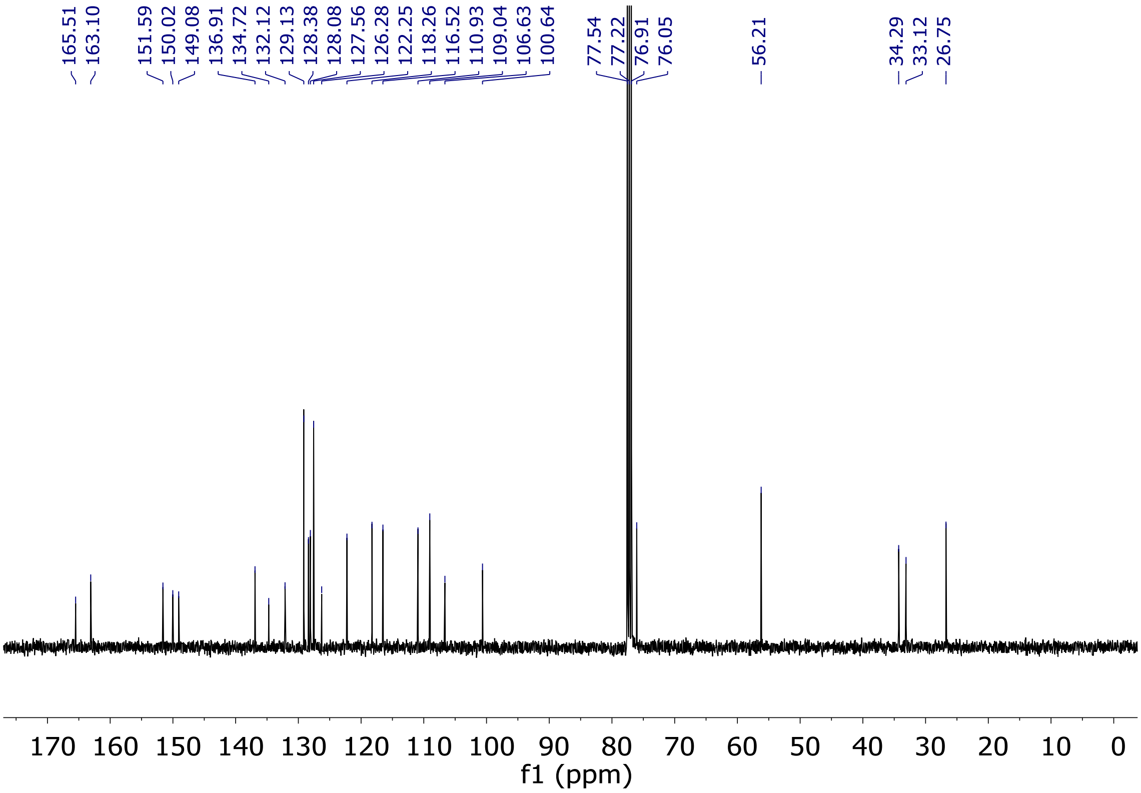

Figure S52.¹³C-NMR spectrum of compound **63a** in $CDCl_3$

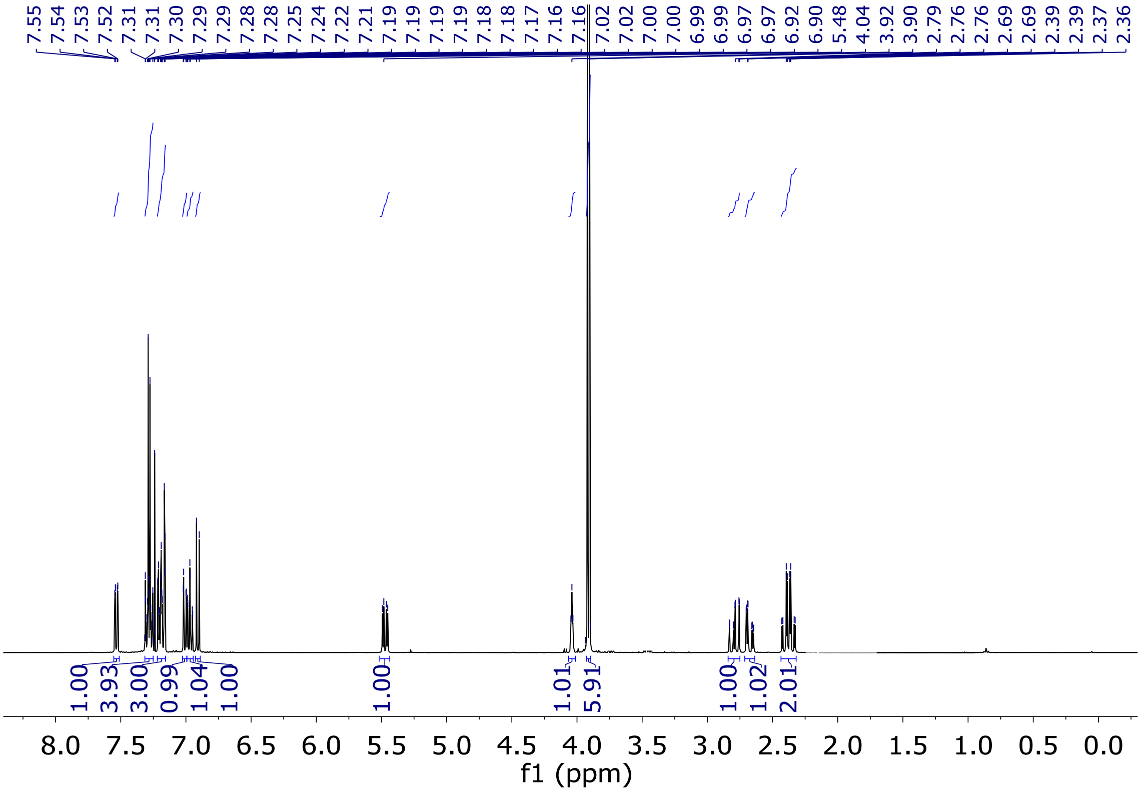

Figure S53.^{1}H-NMR spectrum of compound **63b** in $CDCl_3$

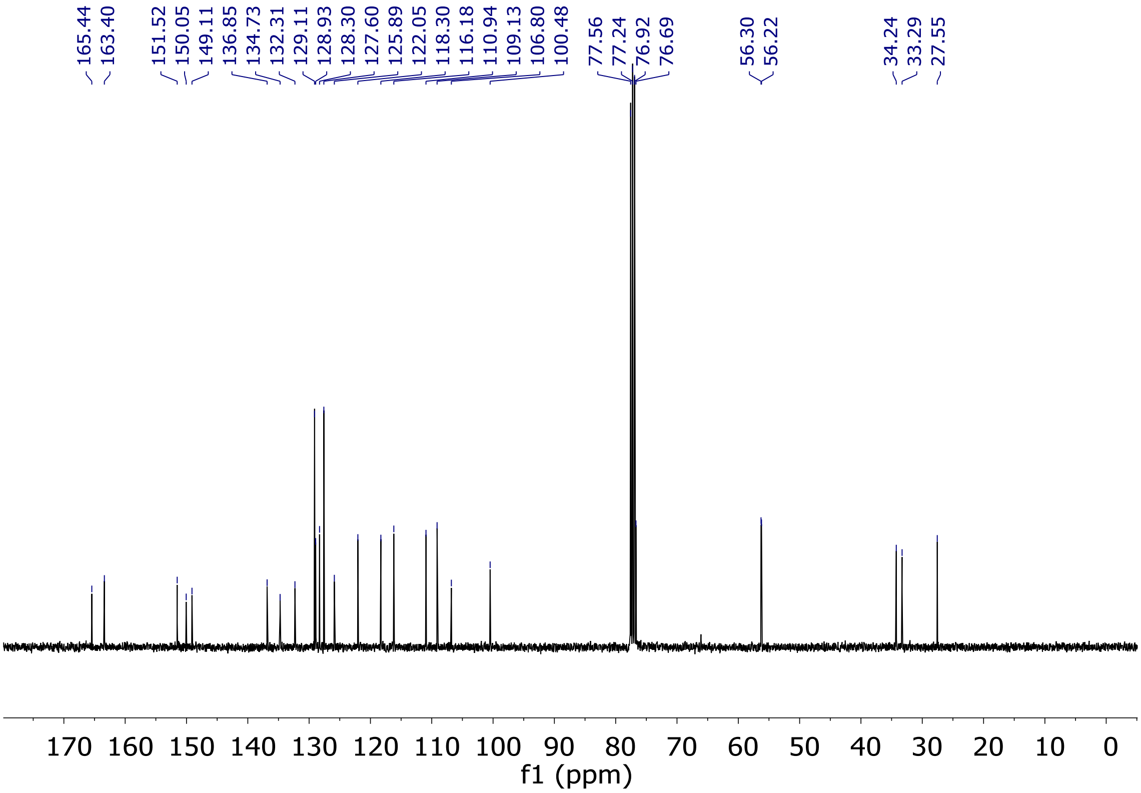

Figure S54.^{13}C-NMR spectrum of compound **63b** in $CDCl_3$

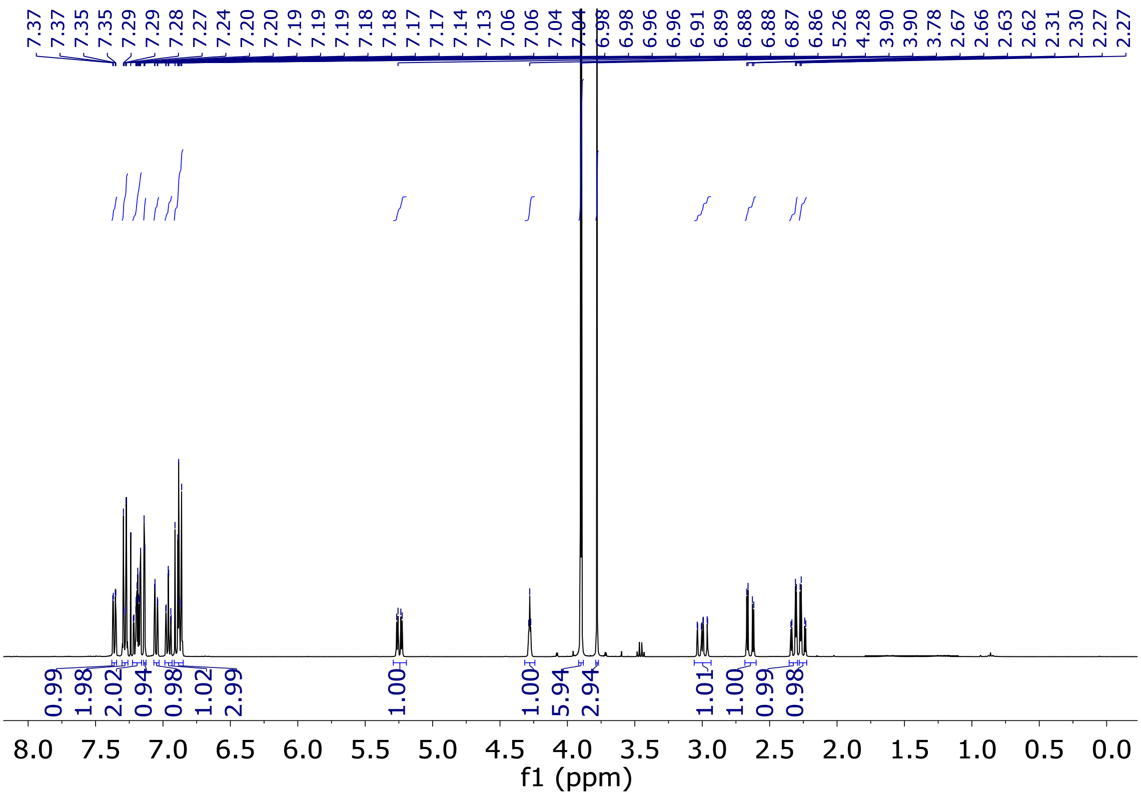

Figure S55. ^{1}H-NMR spectrum of compound **64a** in $CDCl_3$

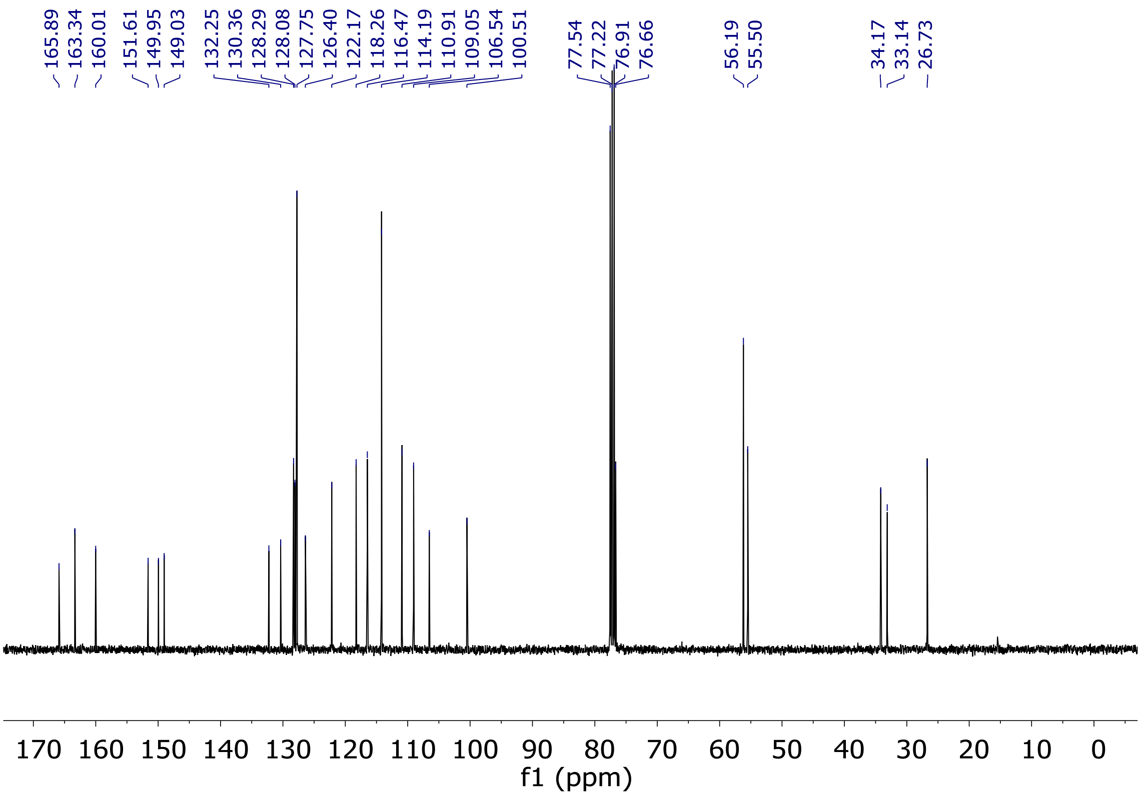

Figure S56. ^{13}C-NMR spectrum of compound **64a** in $CDCl_3$

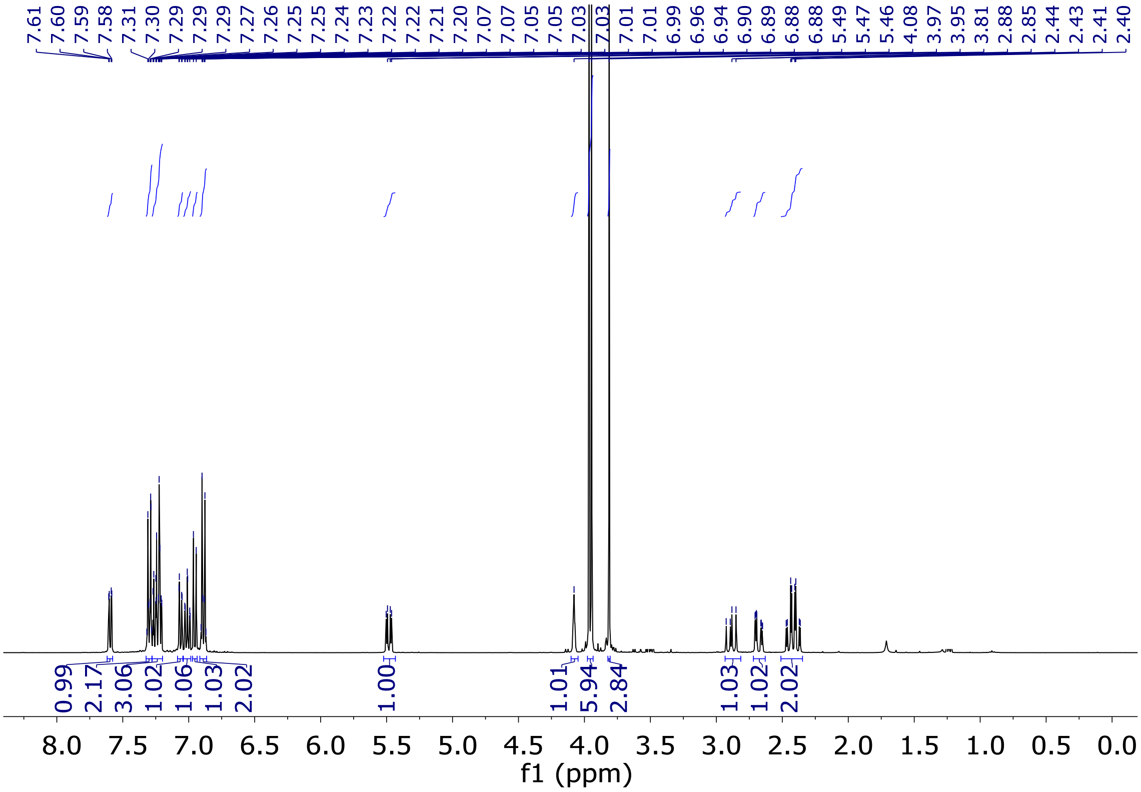

Figure S57.^{1}H-NMR spectrum of compound **64b** in $CDCl_3$

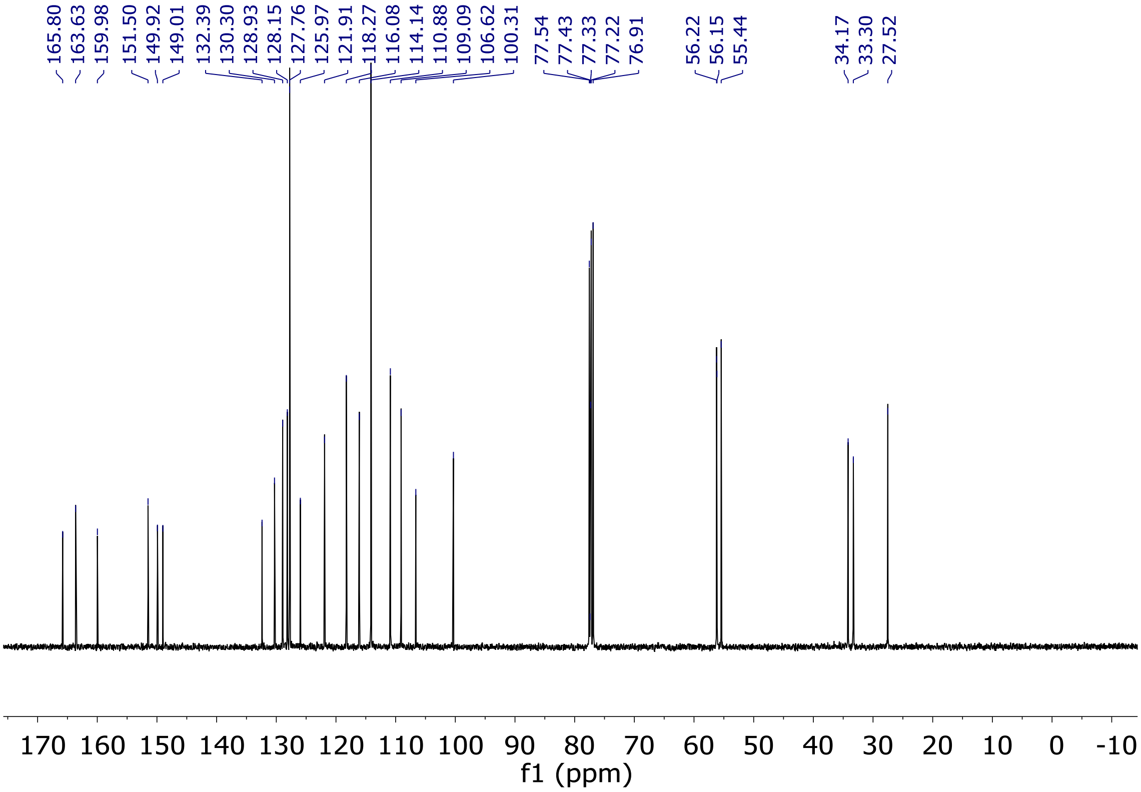

Figure S58.^{13}C-NMR spectrum of compound **64b** in $CDCl_3$

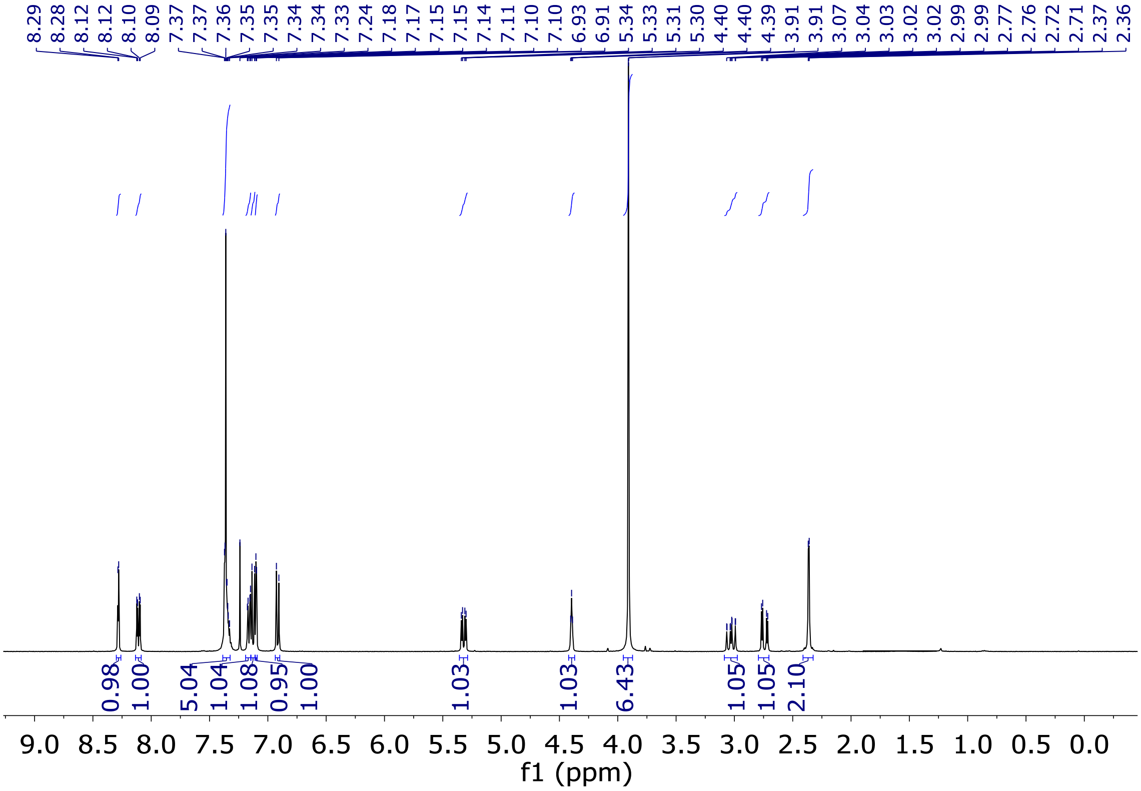

Figure S59.^{1}H-NMR spectrum of compound **65a** in $CDCl_3$

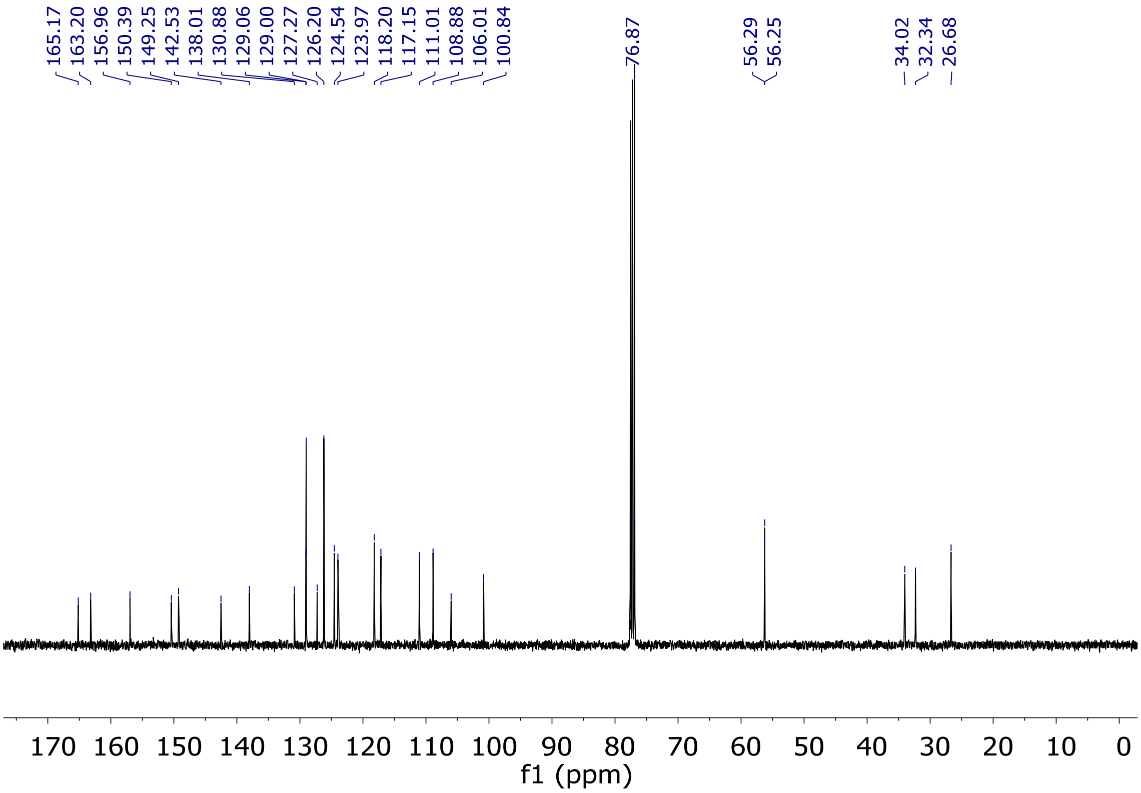

Figure S60.^{13}C-NMR spectrum of compound **65a** in $CDCl_3$

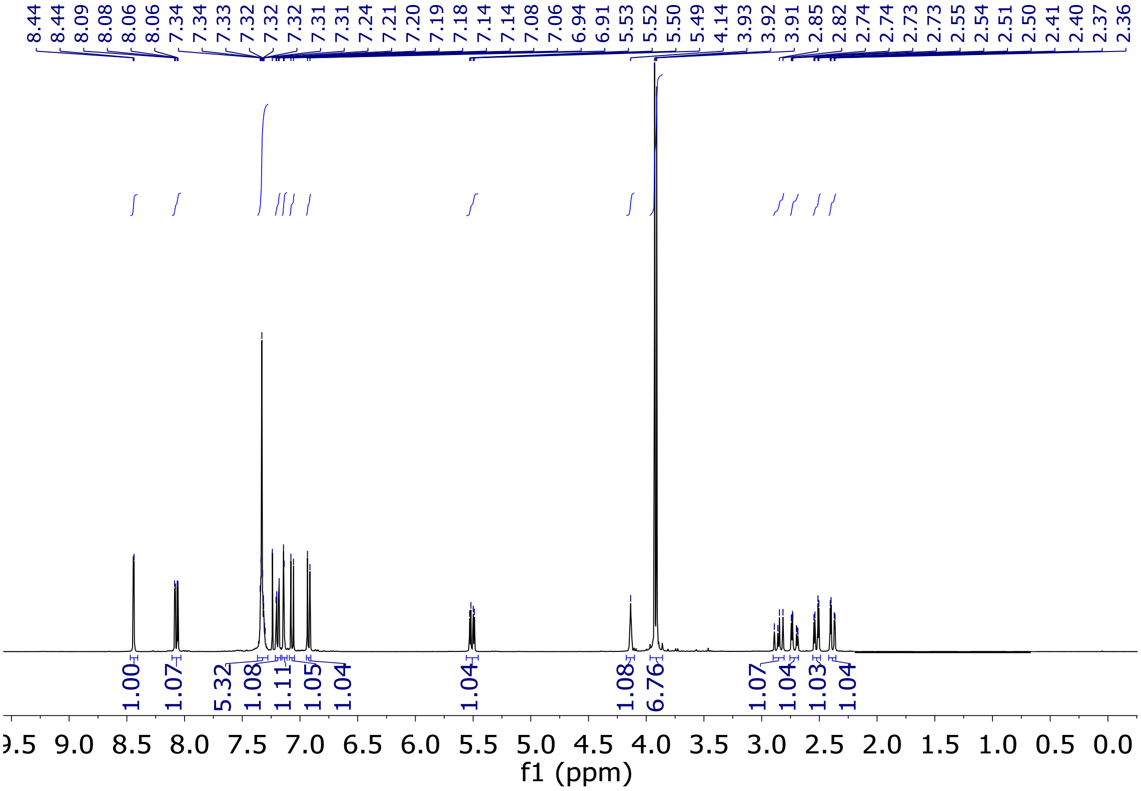

Figure S61.^{1}H-NMR spectrum of compound **65b** in $CDCl_3$

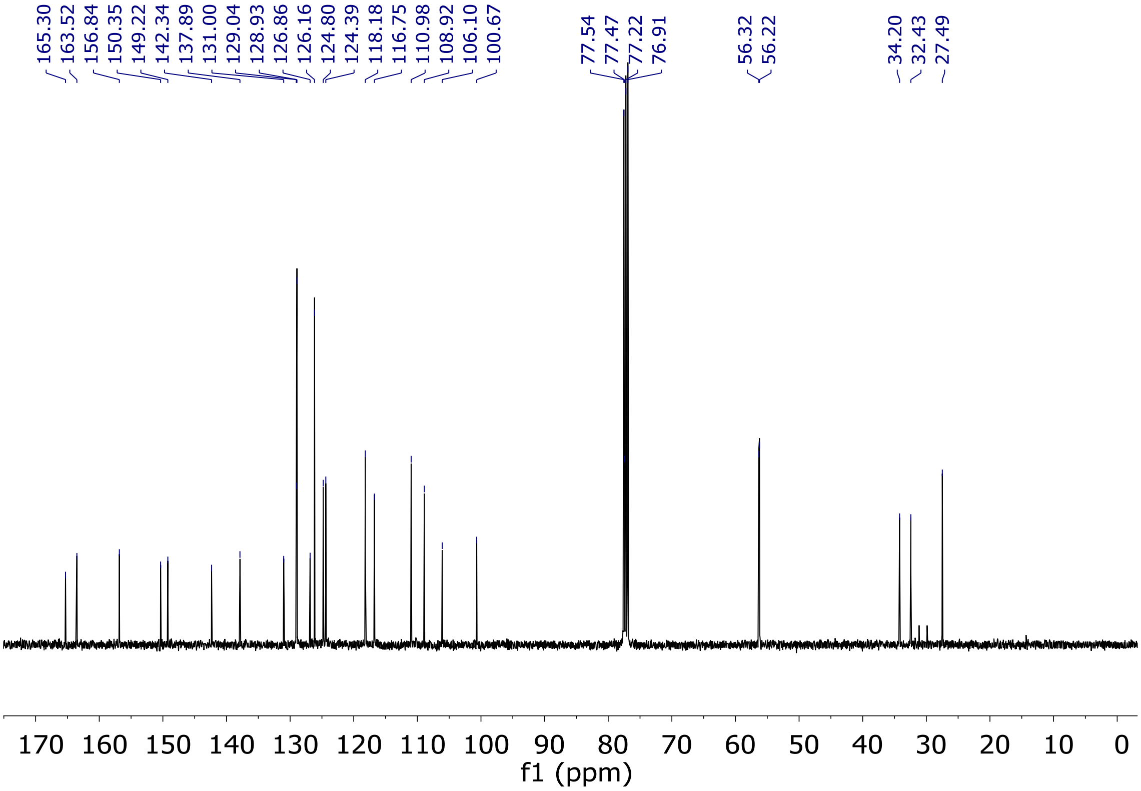

Figure S62.^{13}C-NMR spectrum of compound **65b** in $CDCl_3$

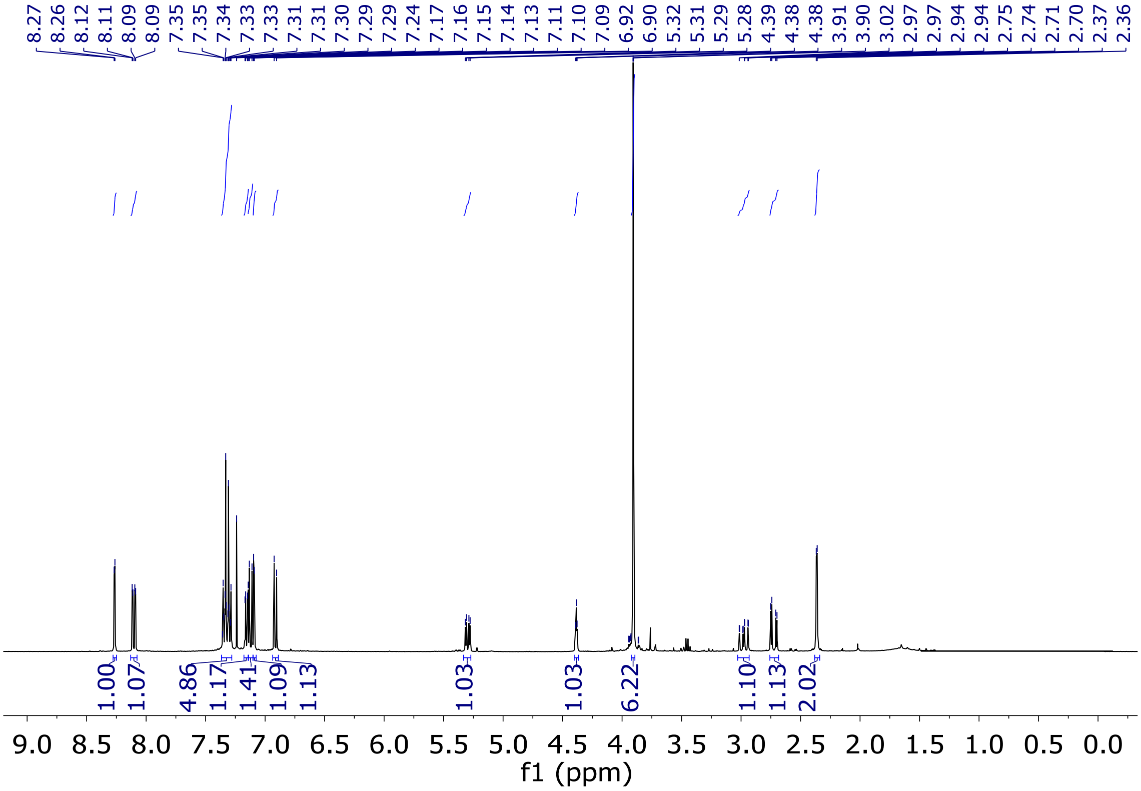

Figure S63. ^{1}H-NMR spectrum of compound **66a** in $CDCl_3$

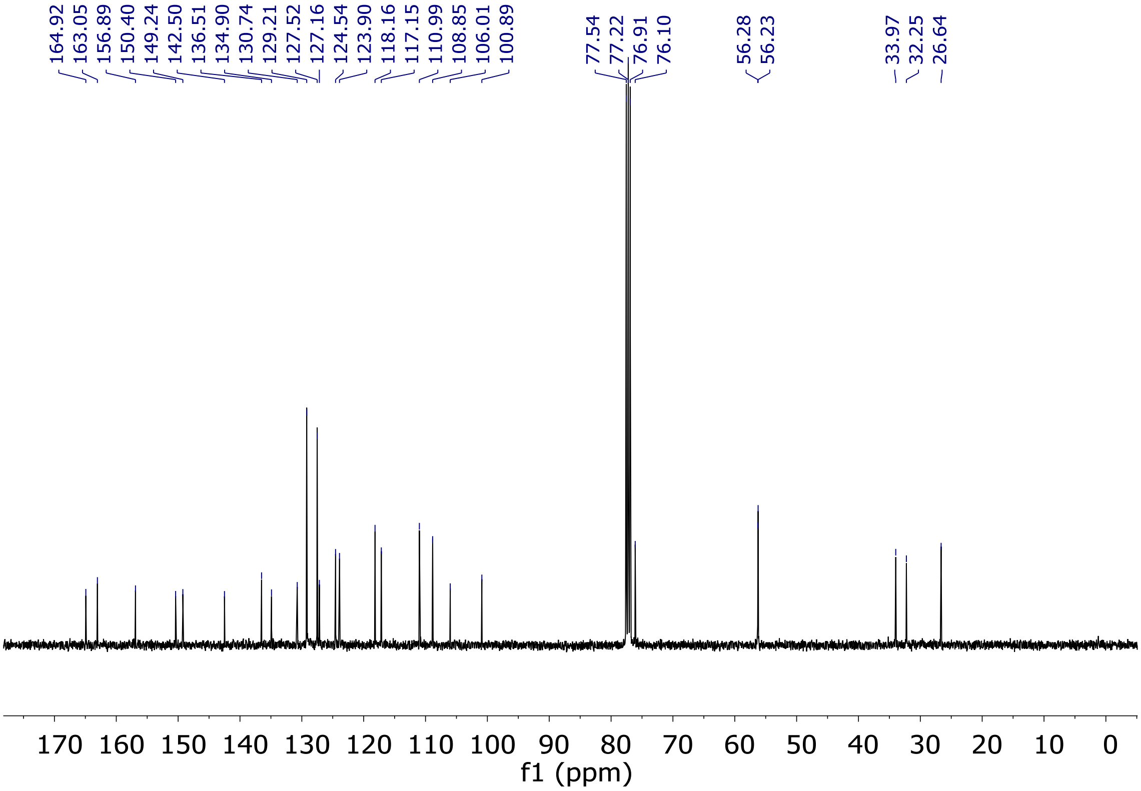

Figure S64. ^{13}C-NMR spectrum of compound **66a** in $CDCl_3$

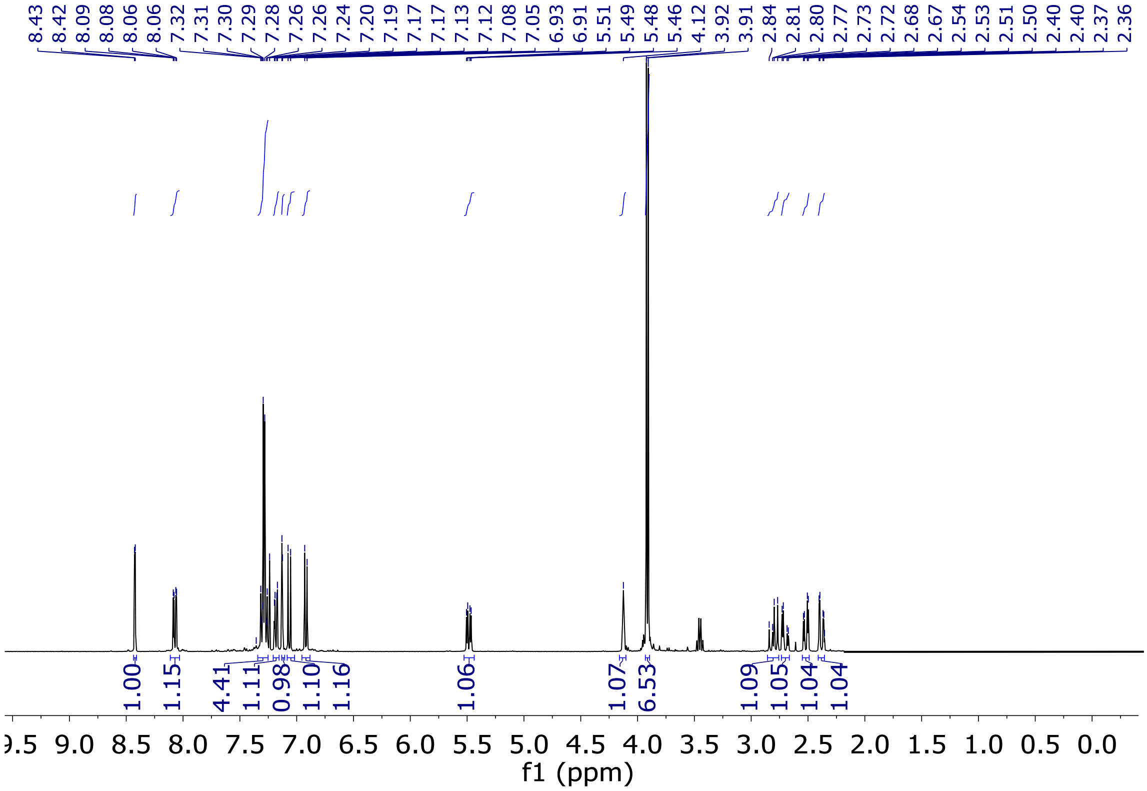

Figure S65.^{1}H-NMR spectrum of compound **66b** in $CDCl_3$

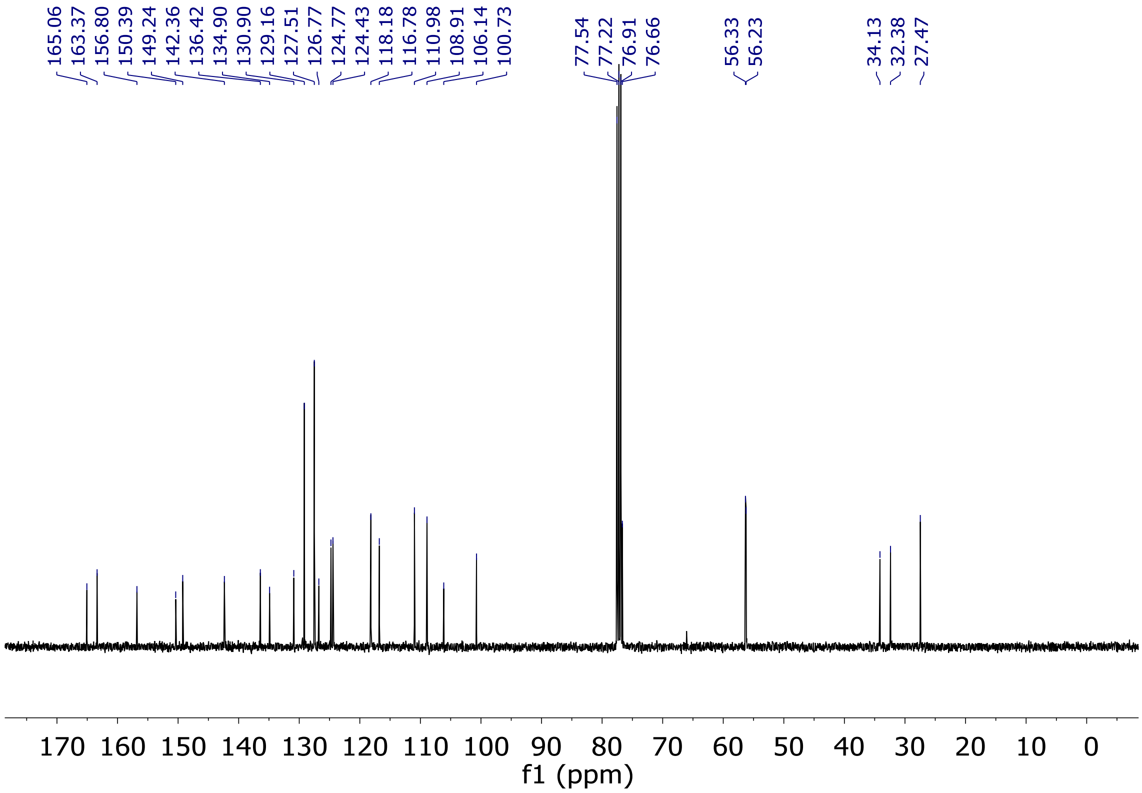

Figure S66.^{13}C-NMR spectrum of compound **66b** in $CDCl_3$

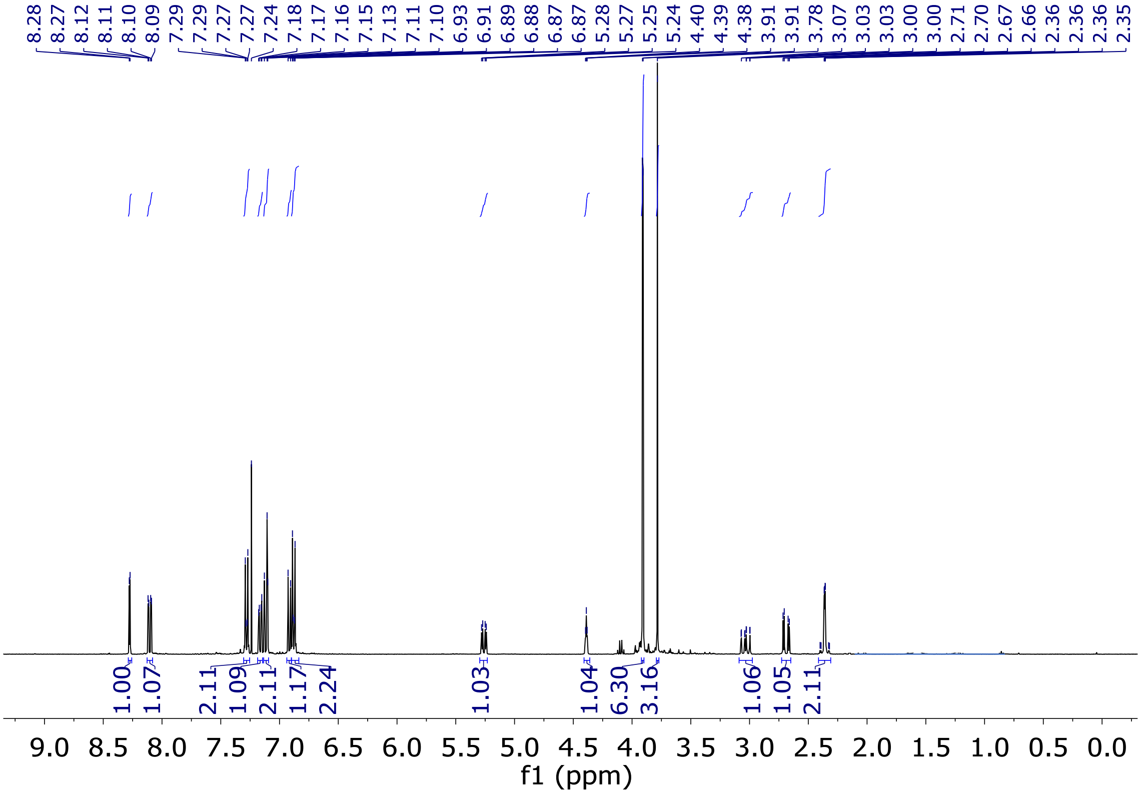

Figure S67.¹H-NMR spectrum of compound **67a** in $CDCl_3$

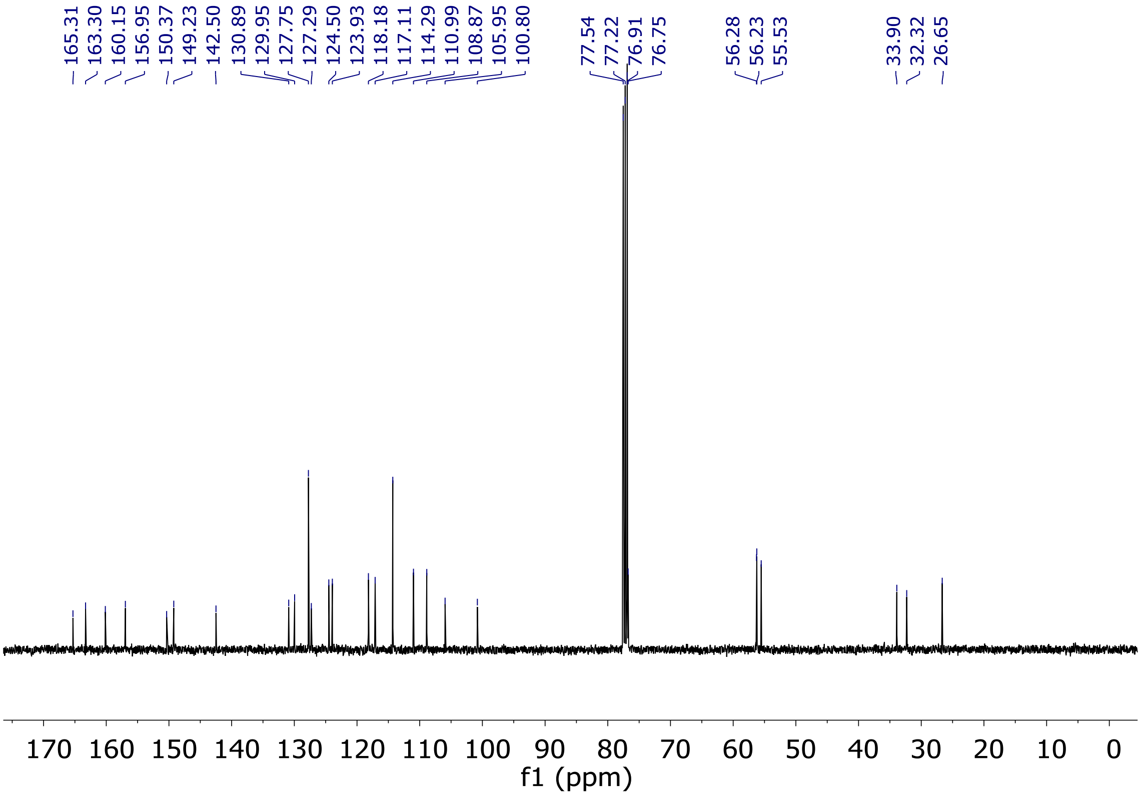

Figure S68.¹³C-NMR spectrum of compound **67a** in $CDCl_3$

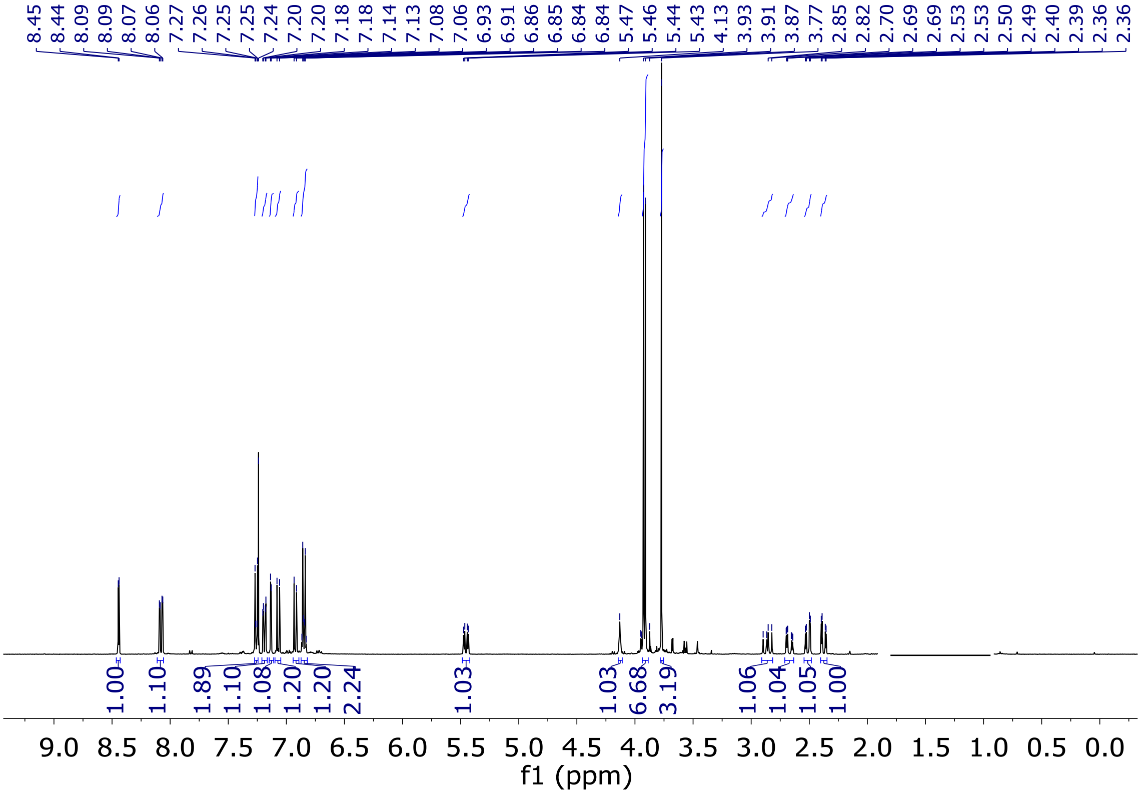

Figure S69.¹H-NMR spectrum of compound **67b** in $CDCl_3$

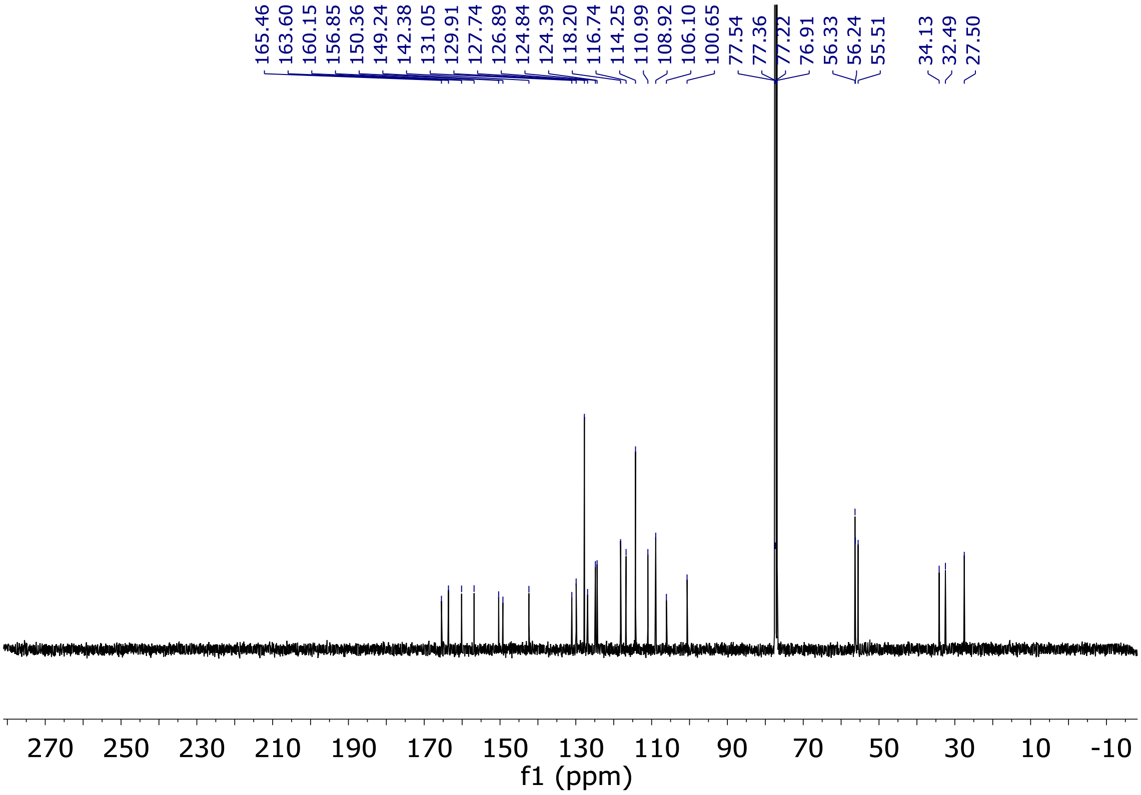

Figure S70.¹³C-NMR spectrum of compound **67b** in $CDCl_3$

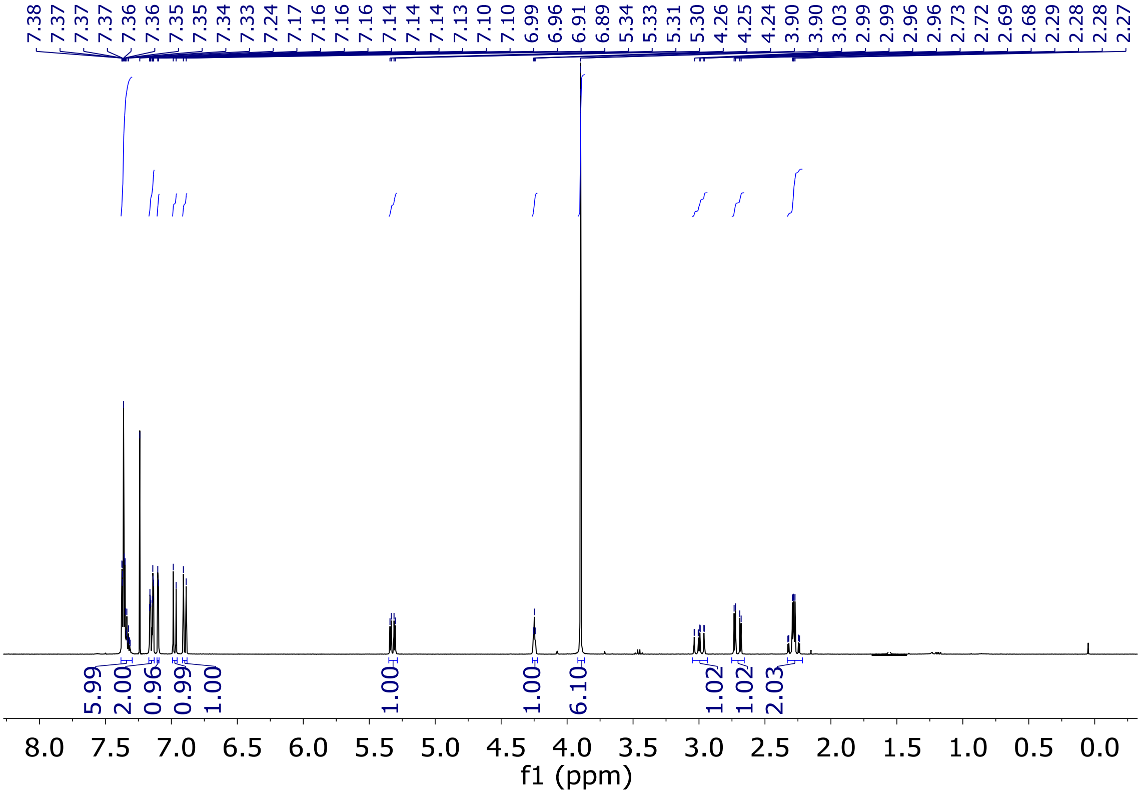

Figure S71.^{1}H-NMR spectrum of compound **68a** in $CDCl_3$

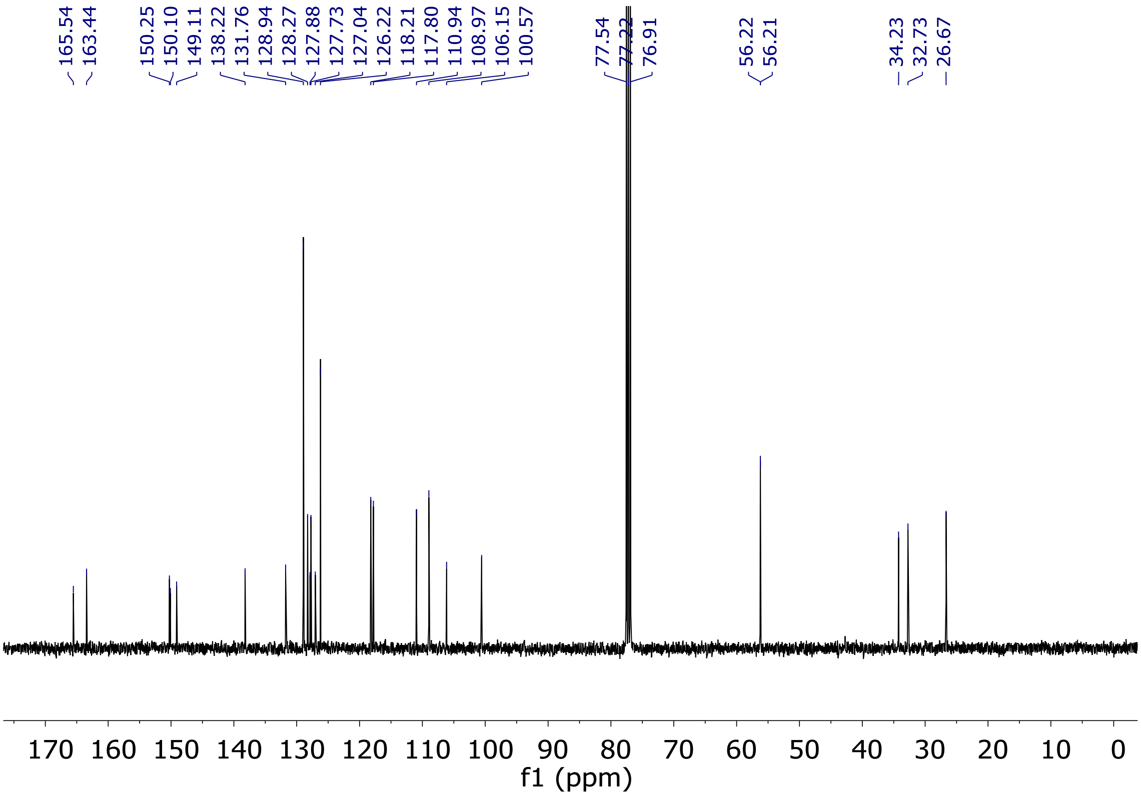

Figure S72.^{13}C-NMR spectrum of compound **68a** in $CDCl_3$

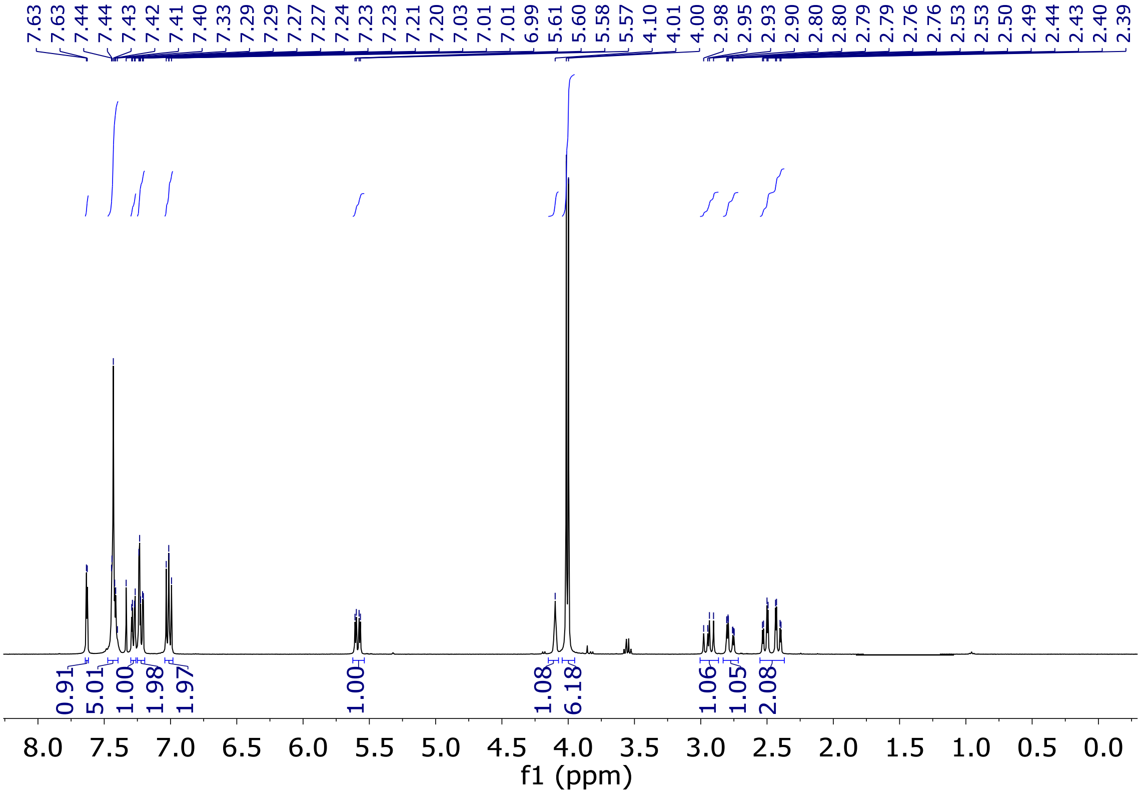

Figure S73.^{1}H-NMR spectrum of compound **68b** in $CDCl_3$

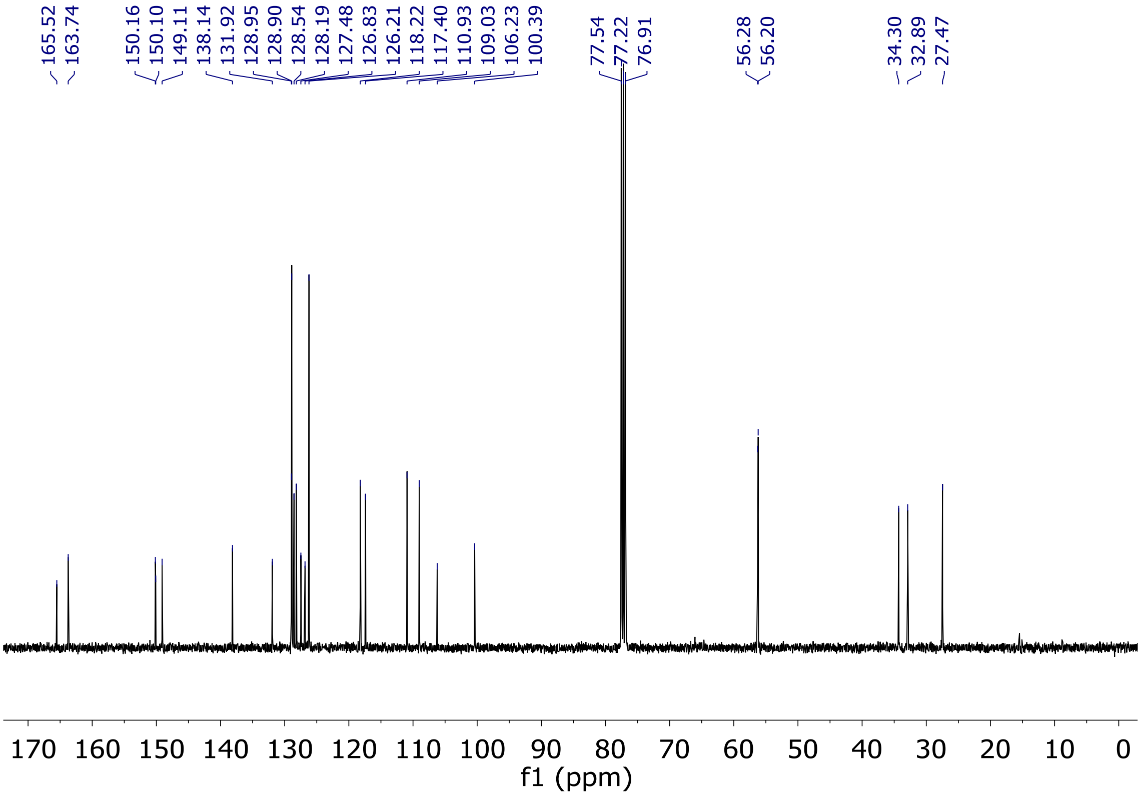

Figure S74.^{13}C-NMR spectrum of compound **68b** in $CDCl_3$

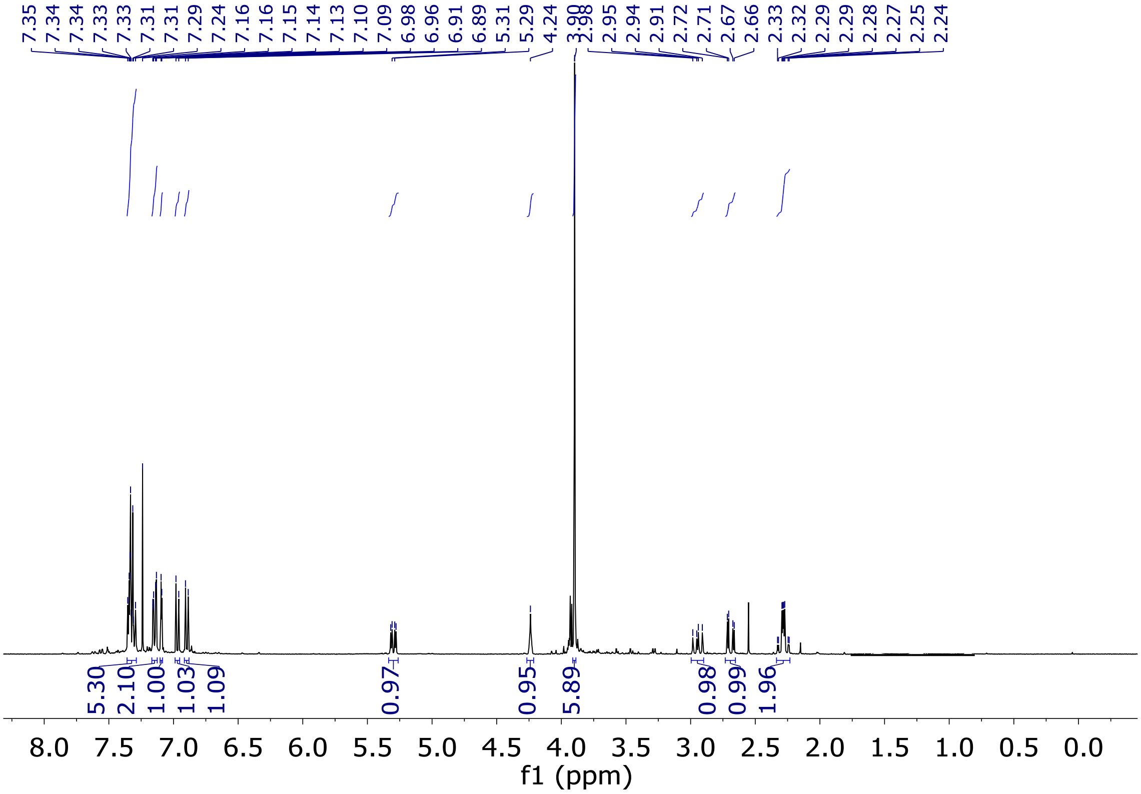

Figure S75.^{1}H-NMR spectrum of compound **69a** in $CDCl_3$

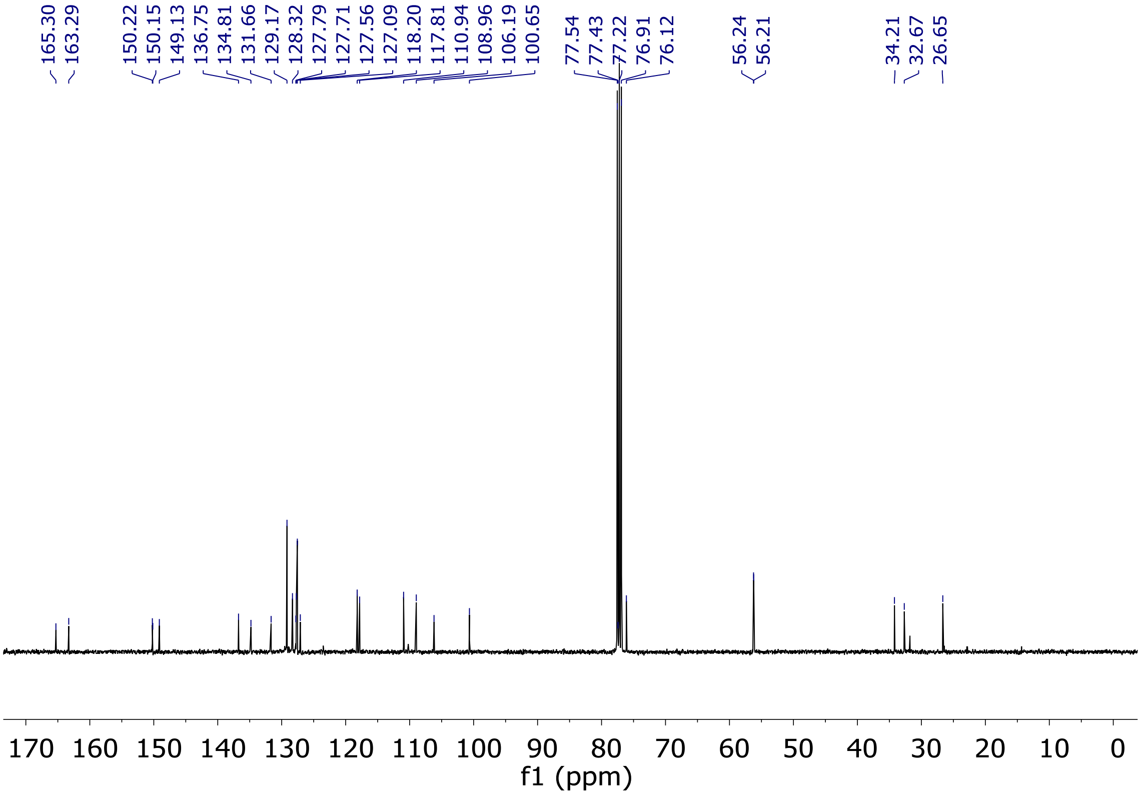

Figure S76.^{13}C-NMR spectrum of compound **69a** in $CDCl_3$

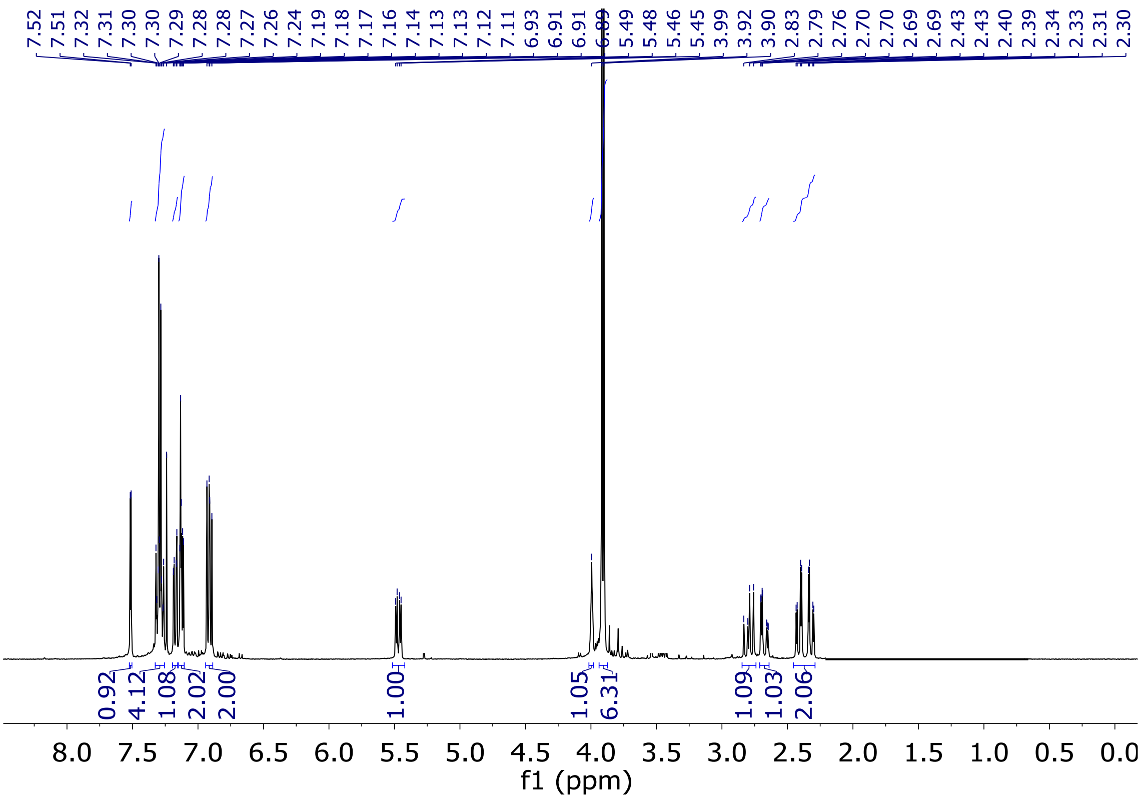

Figure S77.¹H-NMR spectrum of compound **69b** in $CDCl_3$

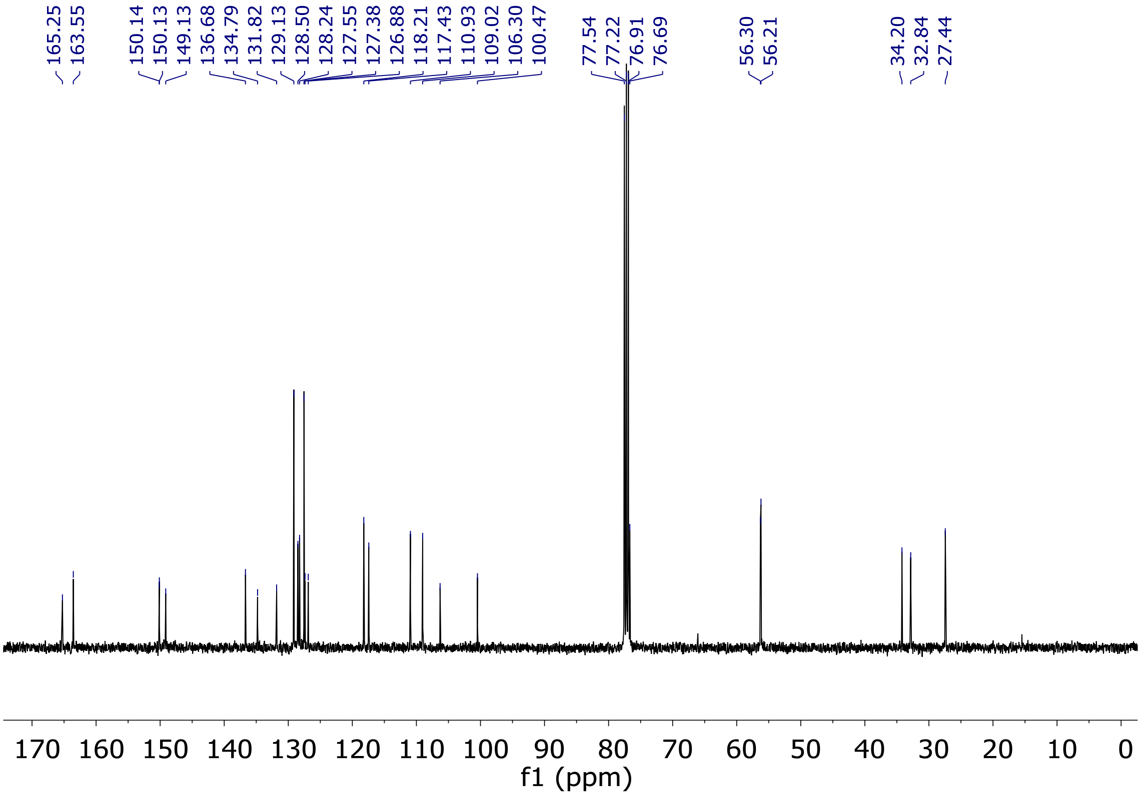

Figure S78.¹³C-NMR spectrum of compound **69b** in $CDCl_3$

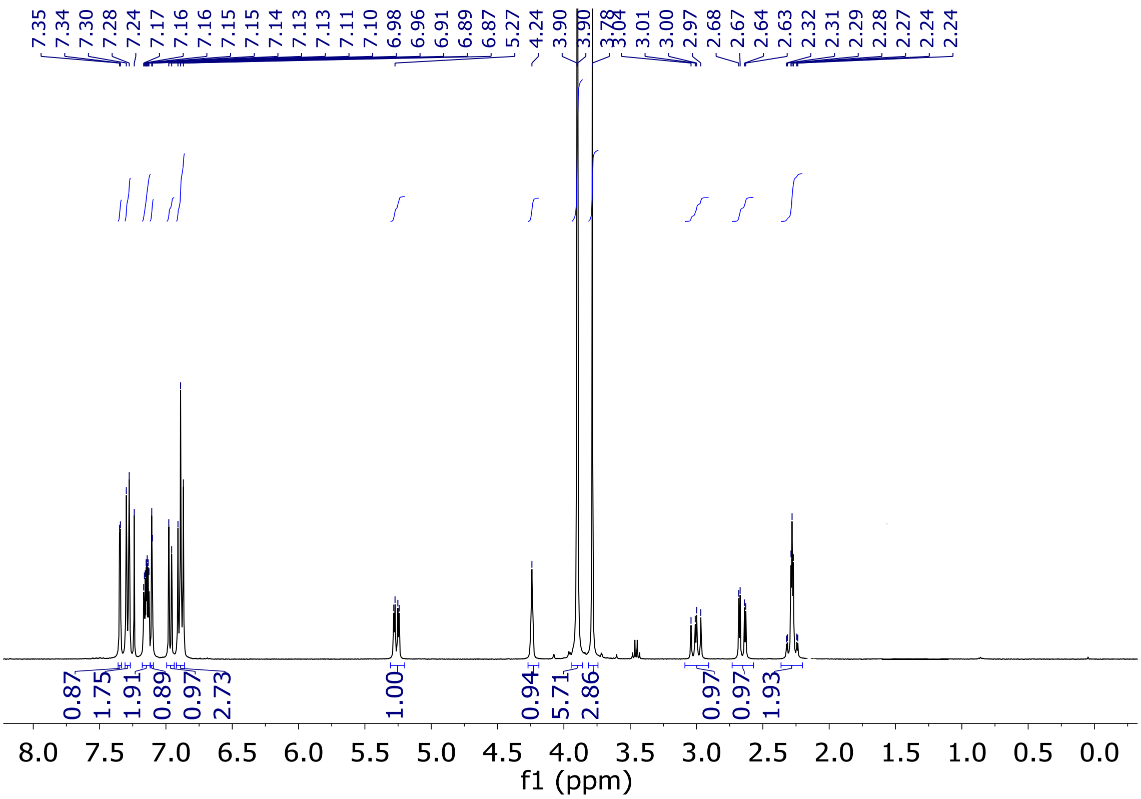

Figure S79.^{1}H-NMR spectrum of compound **70a** in $CDCl_3$

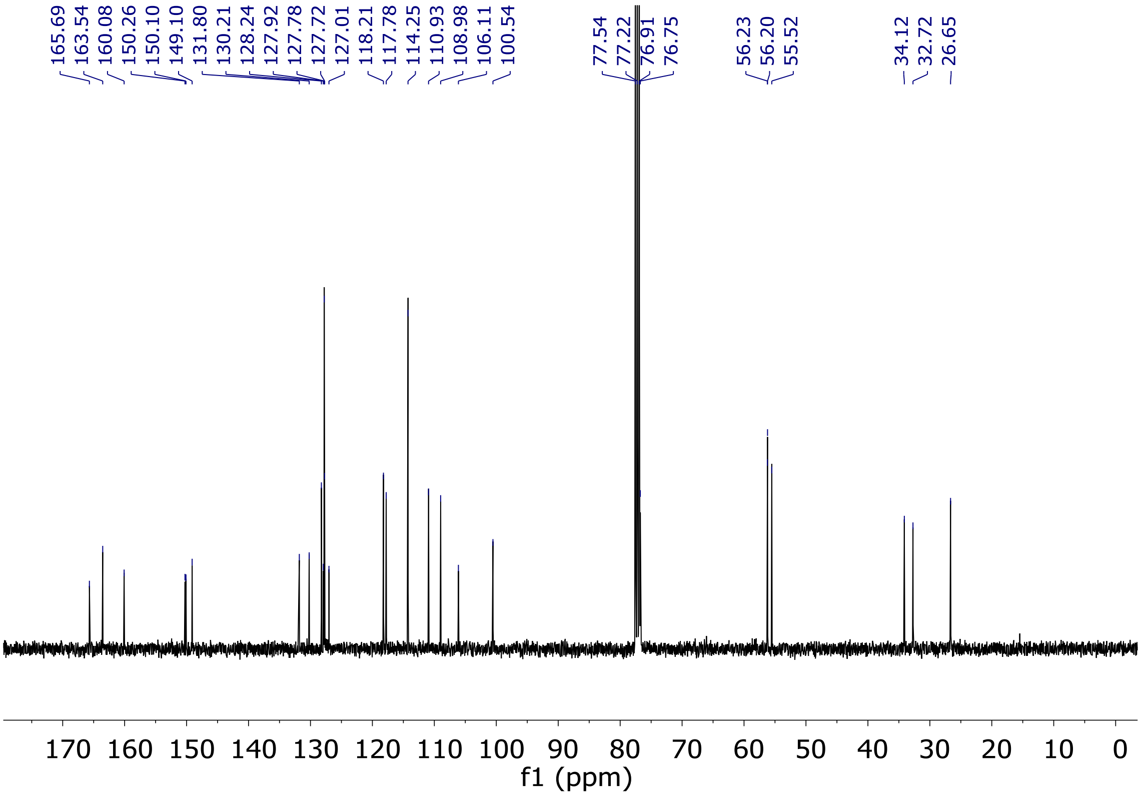

Figure S80.^{13}C-NMR spectrum of compound **70a** in $CDCl_3$

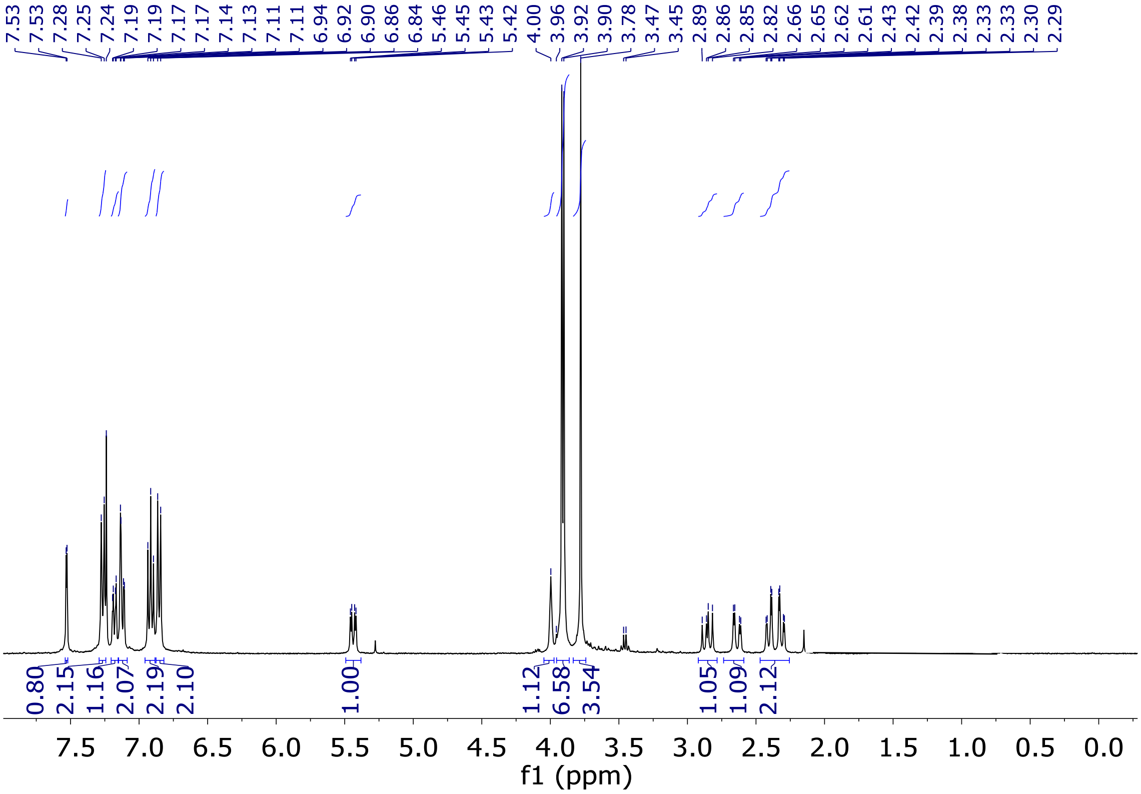

Figure S81.^{1}H-NMR spectrum of compound **70b** in $CDCl_3$

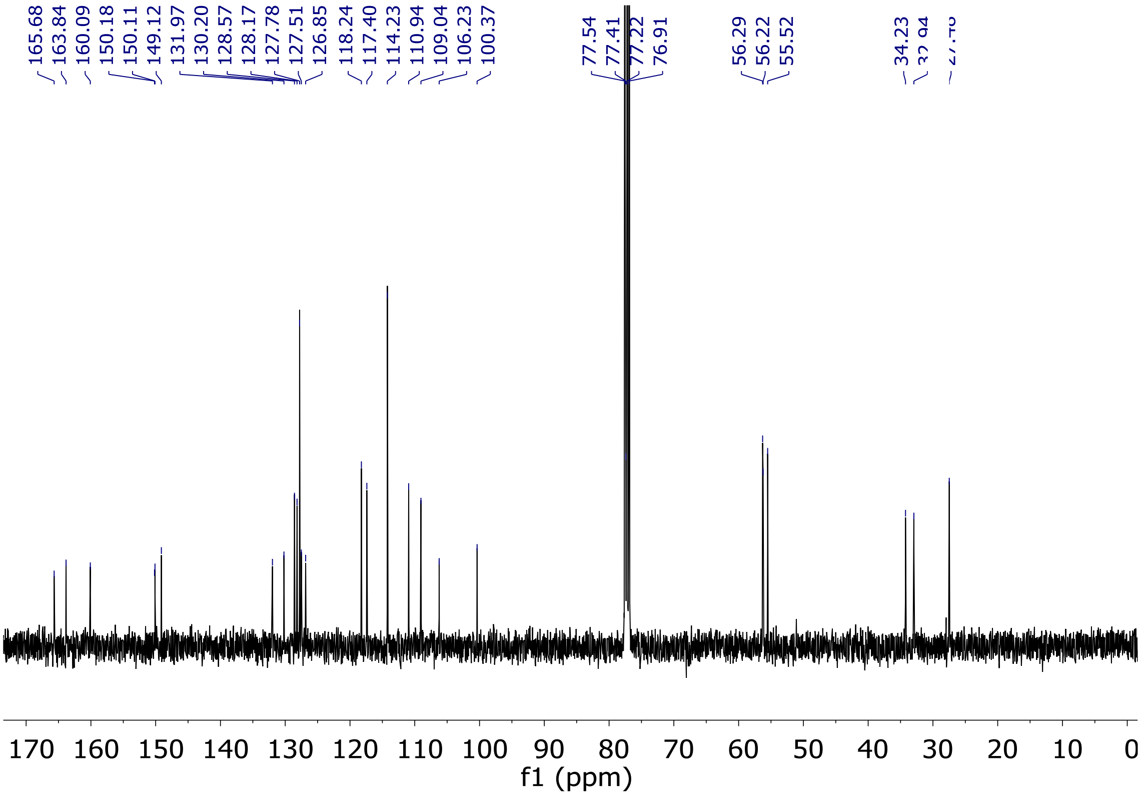

Figure S82.^{13}C-NMR spectrum of compound **70b** in $CDCl_3$

2. HPLC chromatograms

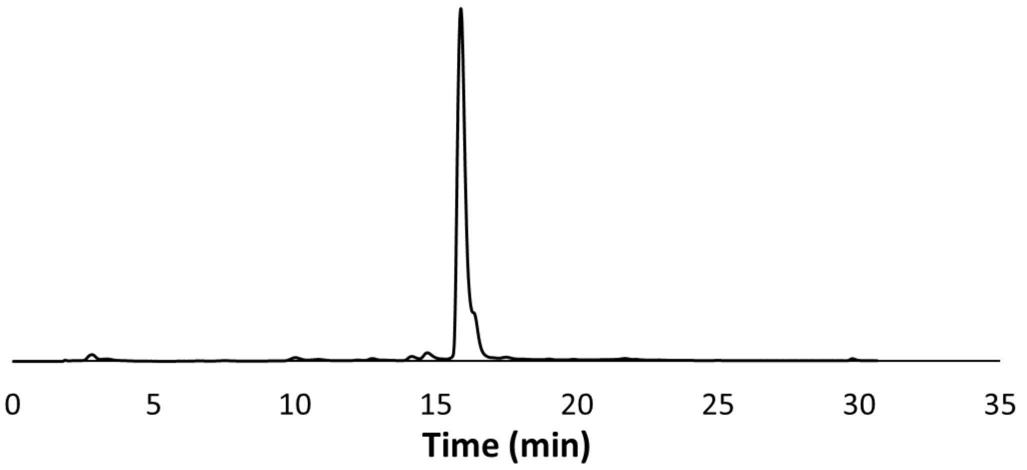

Figure S83. HPLC Chromatogram at 280 nm of compound **1** (r.t. = 15.9 min). Purity 98%

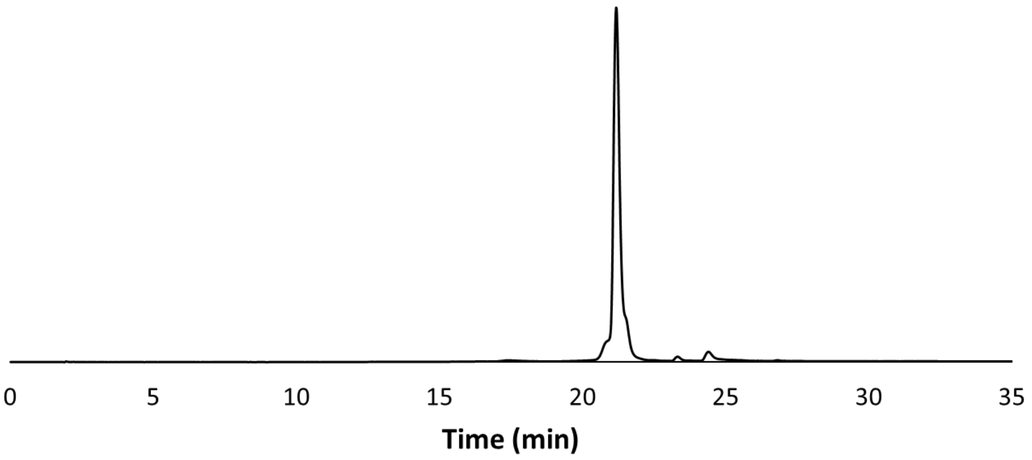

Figure S84. HPLC Chromatogram at 280 nm of compound **2** (r.t. = 21.2 min). Purity 97%

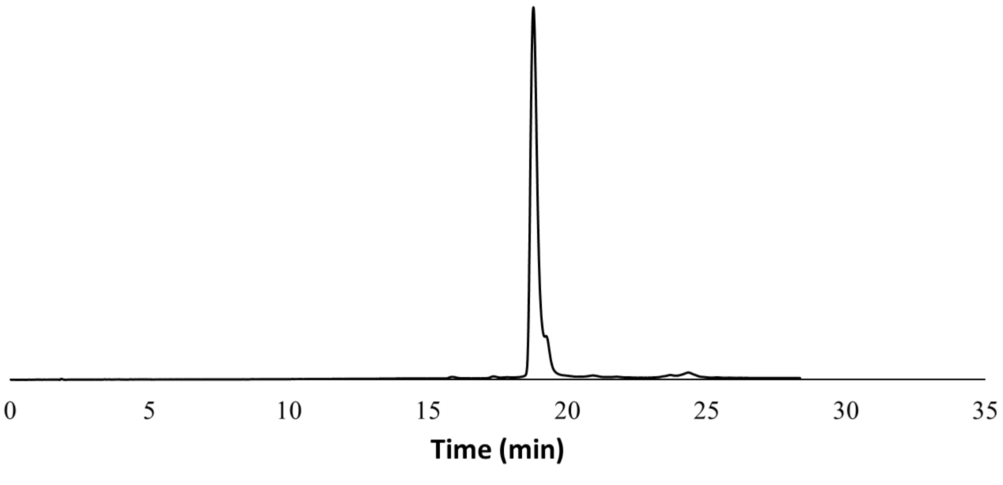

Figure S85. HPLC Chromatogram at 280 nm of compound **3** (r.t. = 18.8 min). Purity 98%

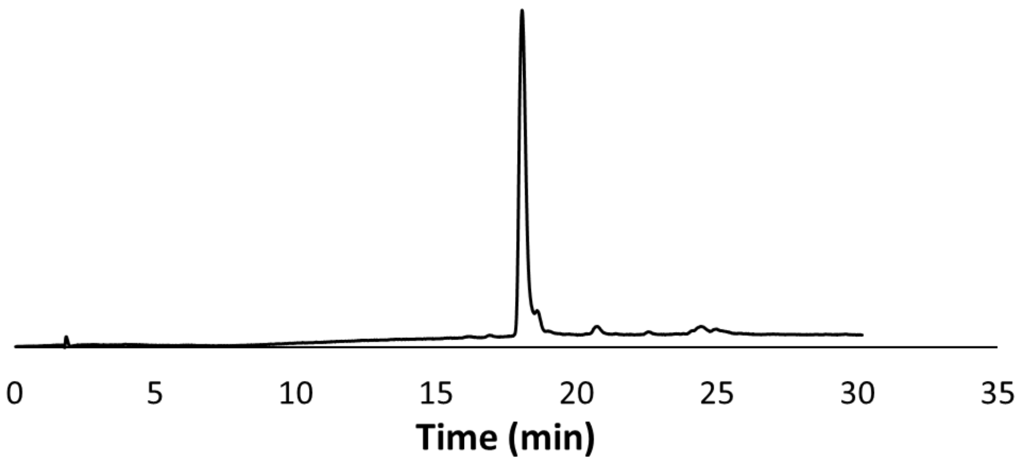

Figure S86. HPLC Chromatogram at 280 nm of compound **4** (r.t. = 18.0 min). Purity 97%

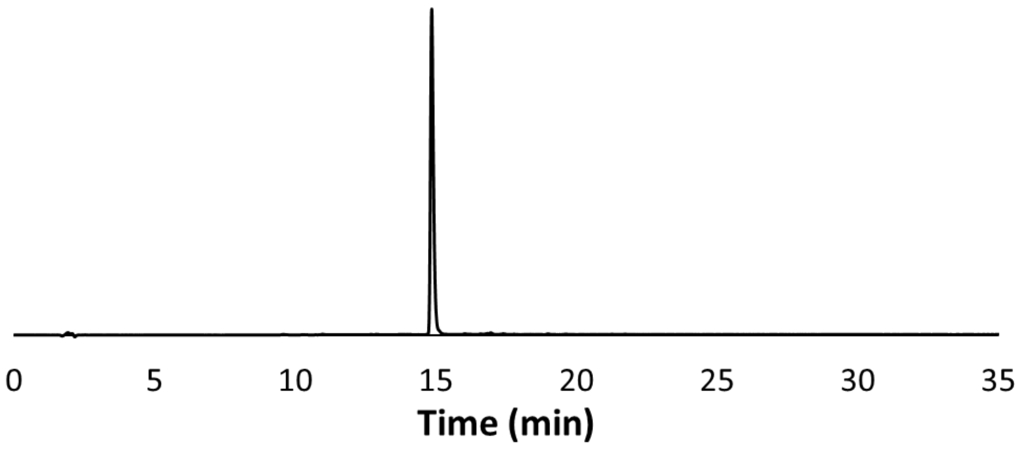

Figure S87. HPLC Chromatogram at 280 nm of compound **5** (r.t. = 14.9 min). Purity >99%

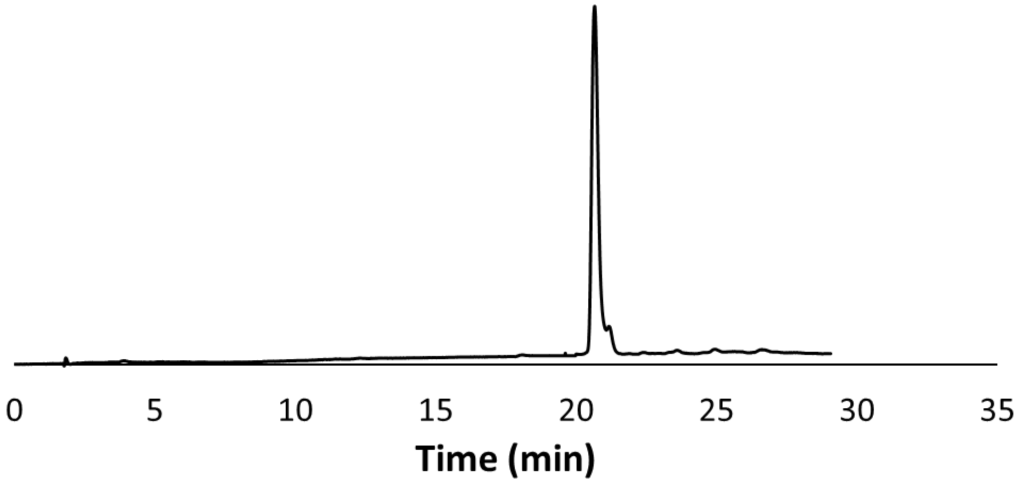

Figure S88. HPLC Chromatogram at 280 nm of compound **6** (r.t. = 20.7 min). Purity 98%

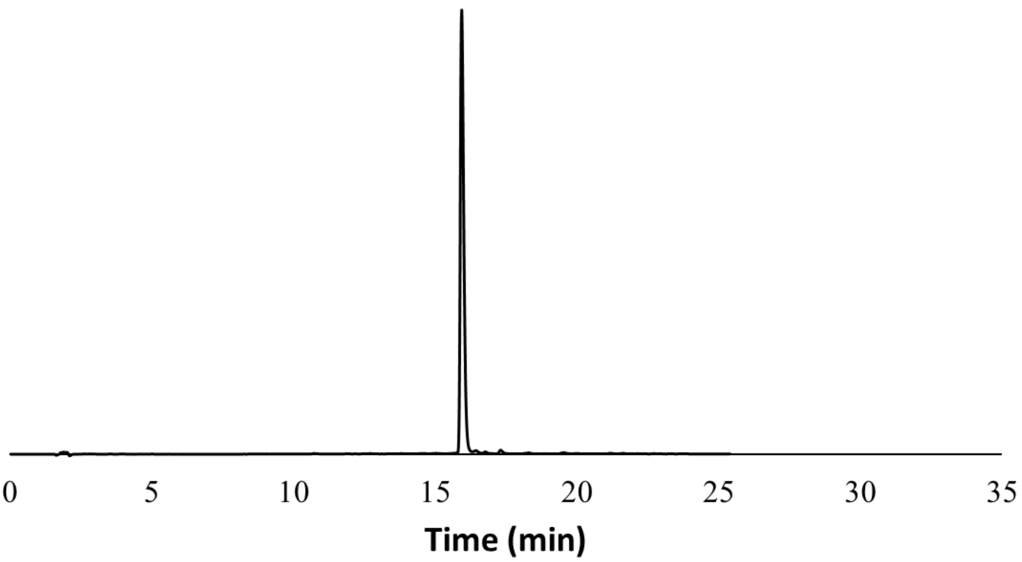

Figure S89. HPLC Chromatogram at 280 nm of compound **42** (r.t. = 15.9 min). Purity >99%

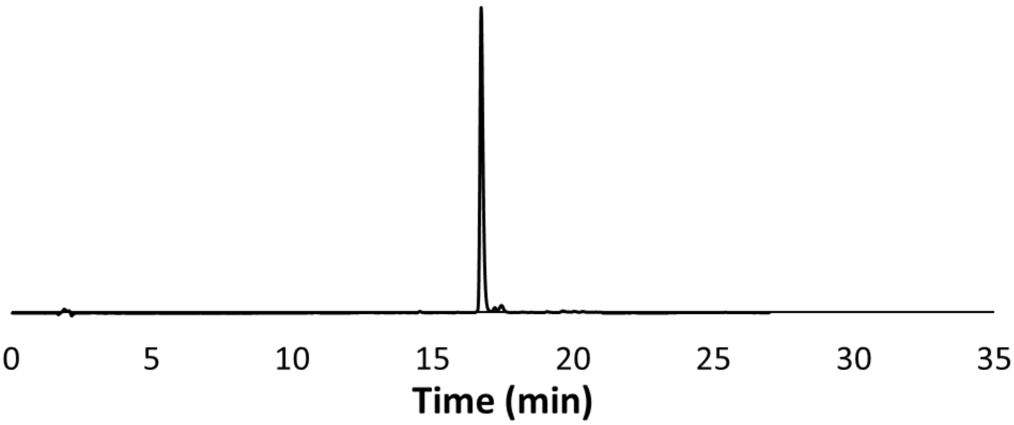

Figure S90. HPLC Chromatogram at 280 nm of compound **43** (r.t. = 16.7 min). Purity 98%

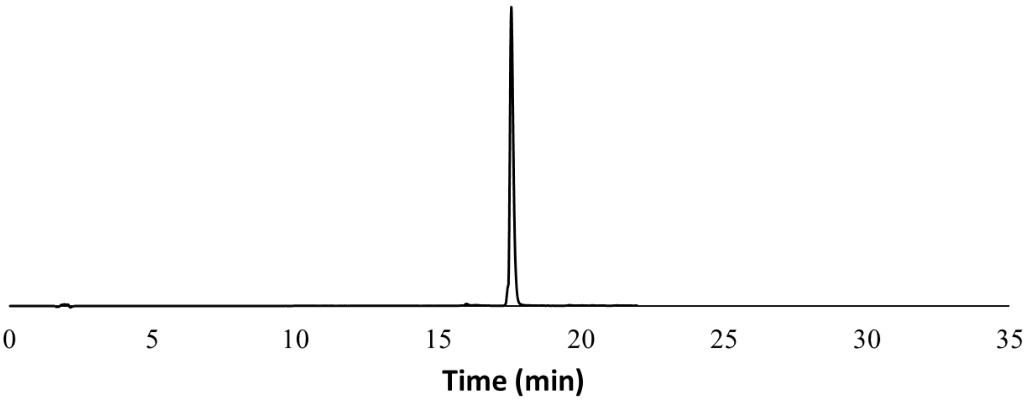

Figure S91. HPLC Chromatogram at 280 nm of compound **44** (r.t. = 17.6 min). Purity >99%

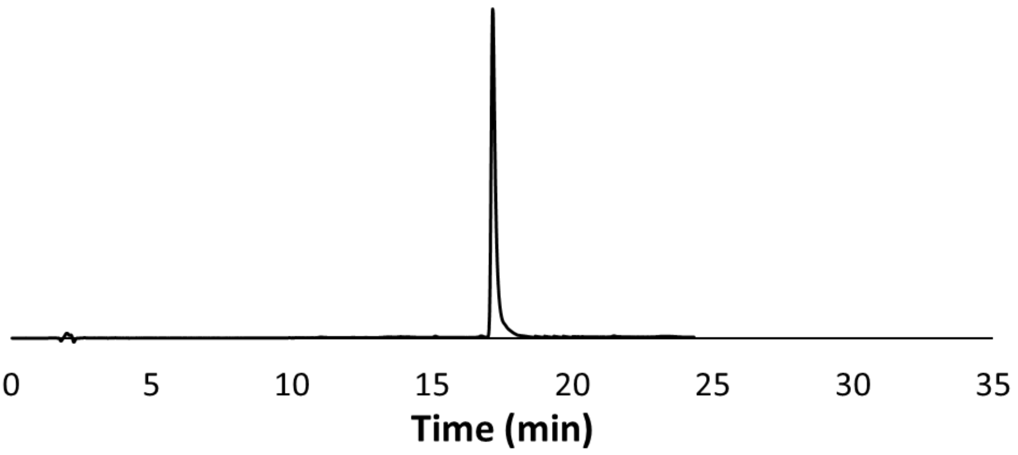

Figure S92. HPLC Chromatogram at 280 nm of compound **45** (r.t. = 17.2 min). Purity >99%

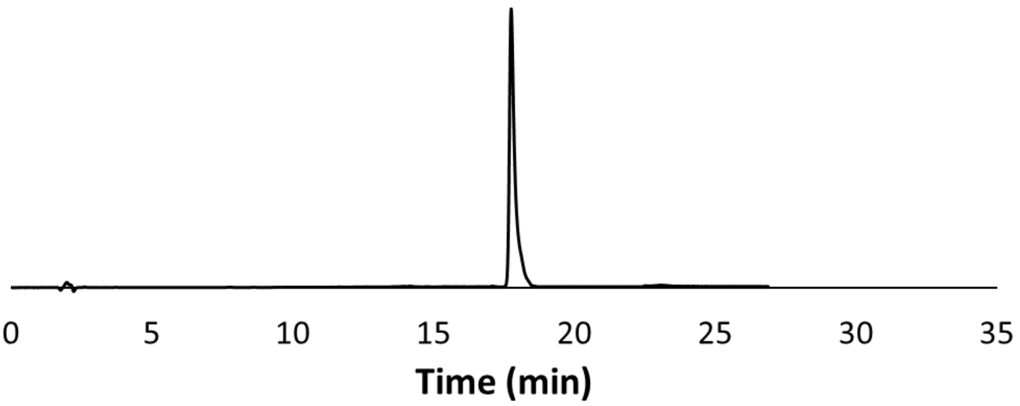

Figure S93. HPLC Chromatogram at 280 nm of compound **46** (r.t. = 17.8 min). Purity 99%

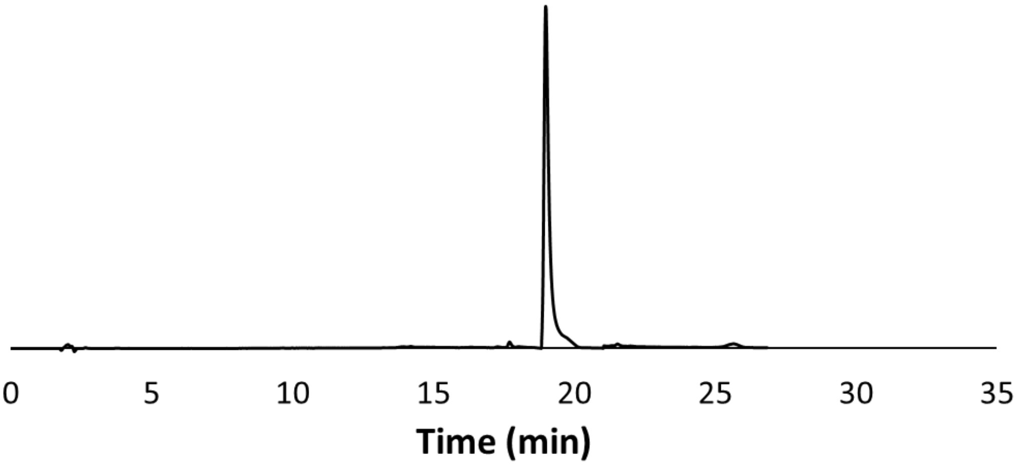

Figure S94. HPLC Chromatogram at 280 nm of compound **47** (r.t. = 19.0 min). Purity 98%

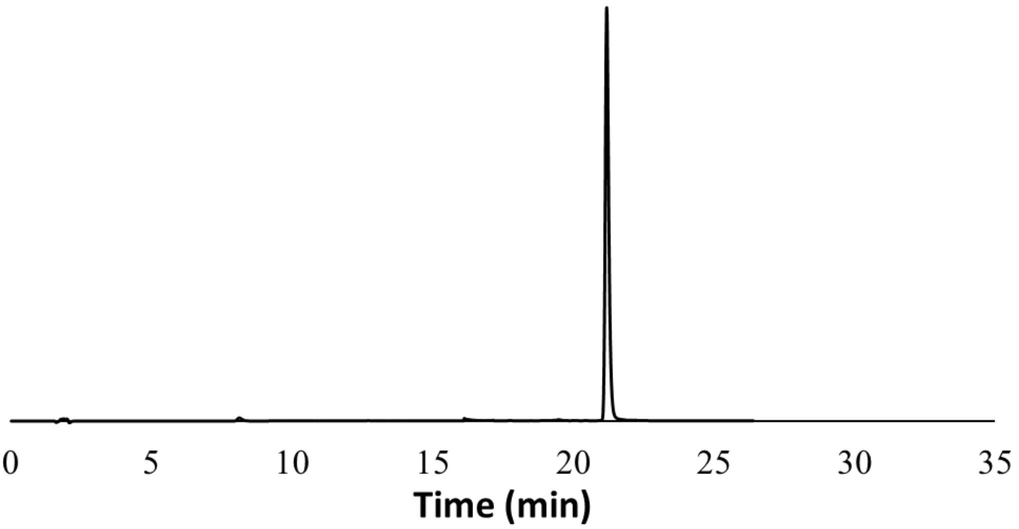

Figure S95. HPLC Chromatogram at 280 nm of compound **48** (r.t. = 21.2 min). Purity 98%

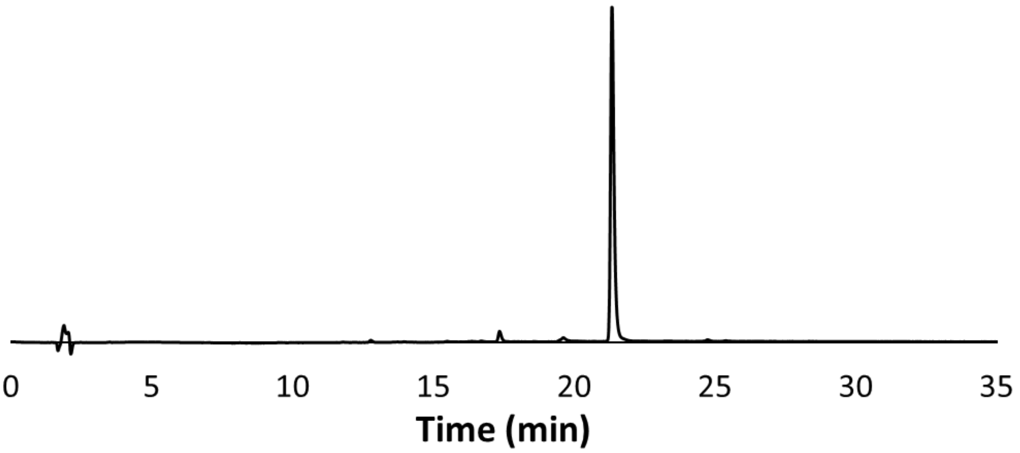

Figure S96. HPLC Chromatogram at 280 nm of compound **49** (r.t. = 21.3 min). Purity 97%

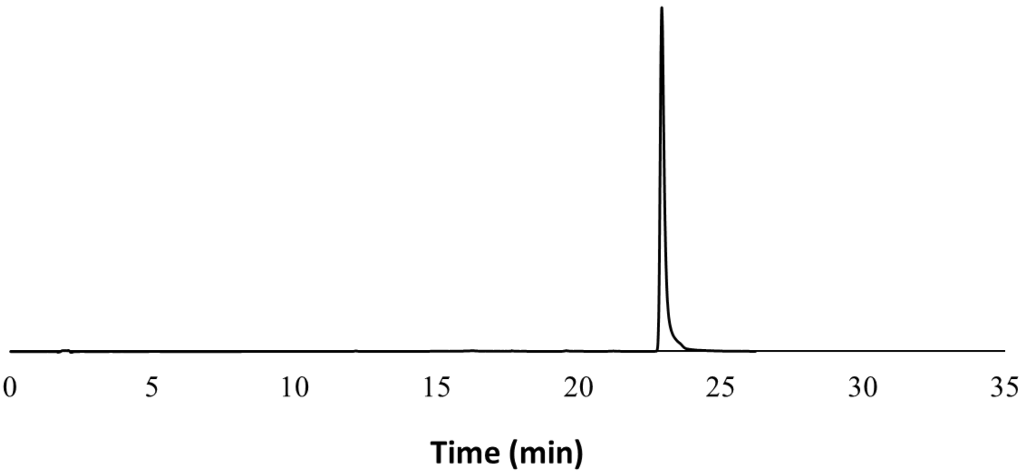

Figure S97. HPLC Chromatogram at 280 nm of compound **50** (r.t. = 22.9 min). Purity >99%

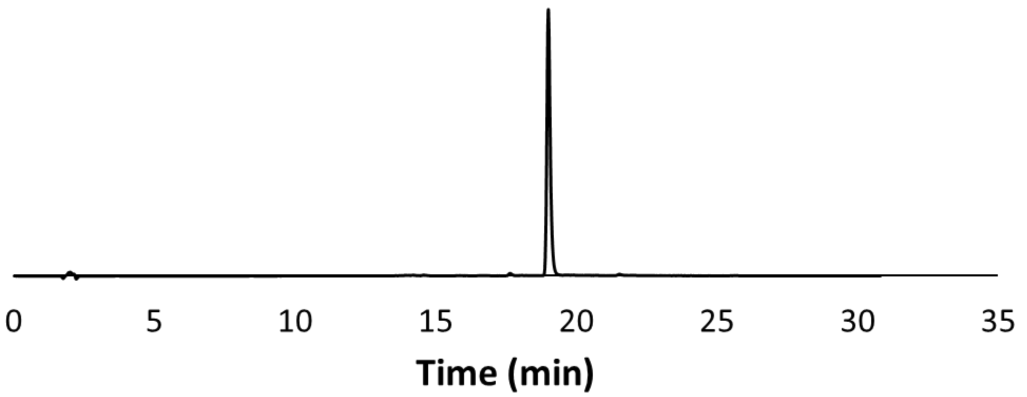

Figure S98. HPLC Chromatogram at 280 nm of compound **51** (r.t. = 19.0 min). Purity >99%

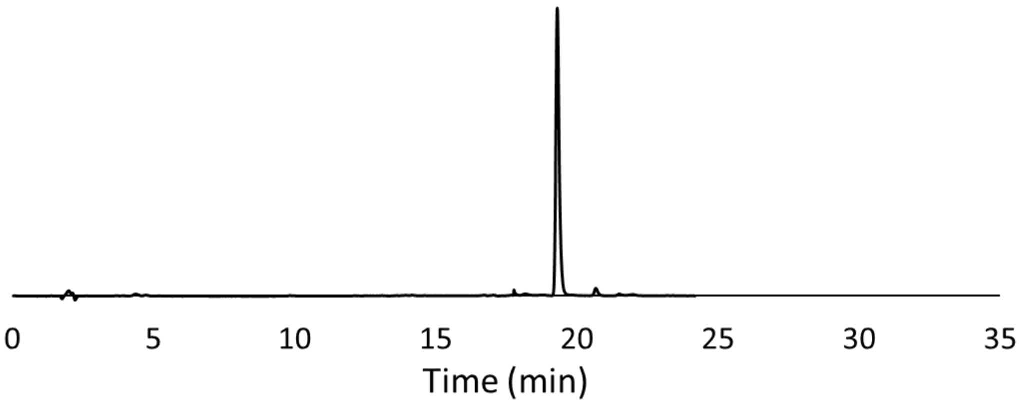

Figure S99. HPLC Chromatogram at 280 nm of compound **52** (r.t. = 19.3 min). Purity 98%

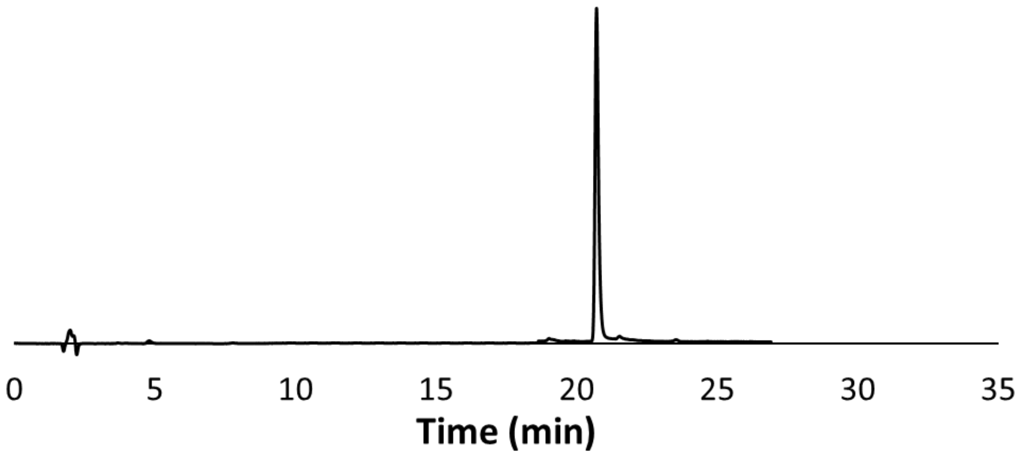

Figure S100. HPLC Chromatogram at 280 nm of compound **53** (r.t. = 20.7 min). Purity 98%

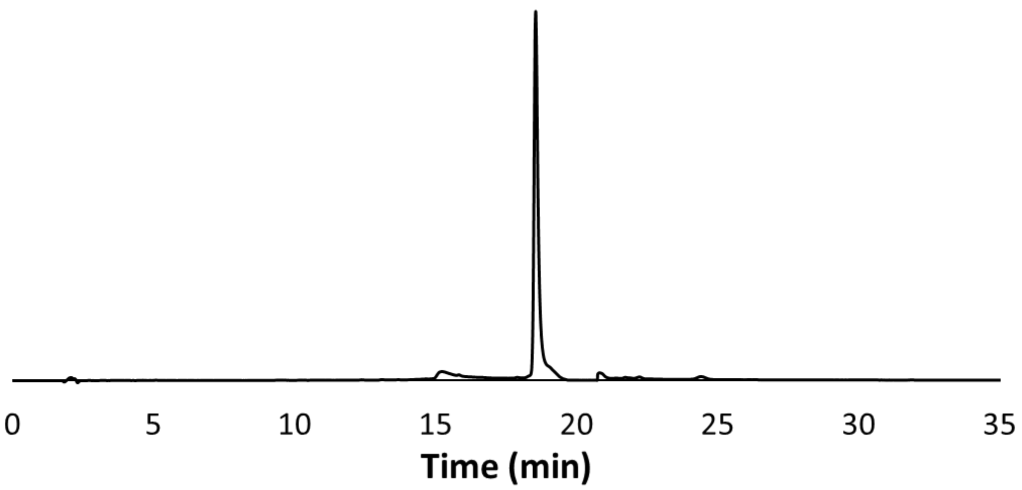

Figure S101. HPLC Chromatogram at 280 nm of compound **54** (r.t. = 18.6 min). Purity 97%

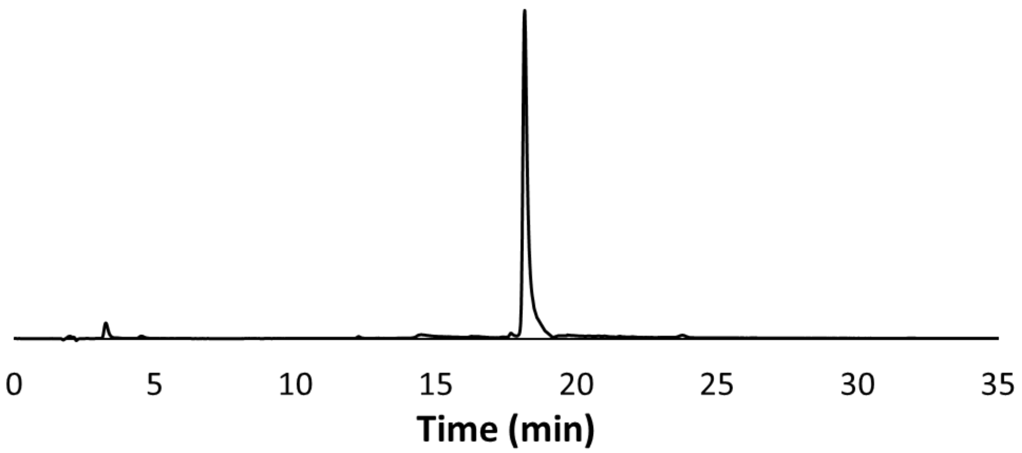

Figure S102. HPLC Chromatogram at 280 nm of compound **55** (r.t. = 18.2 min). Purity 98%

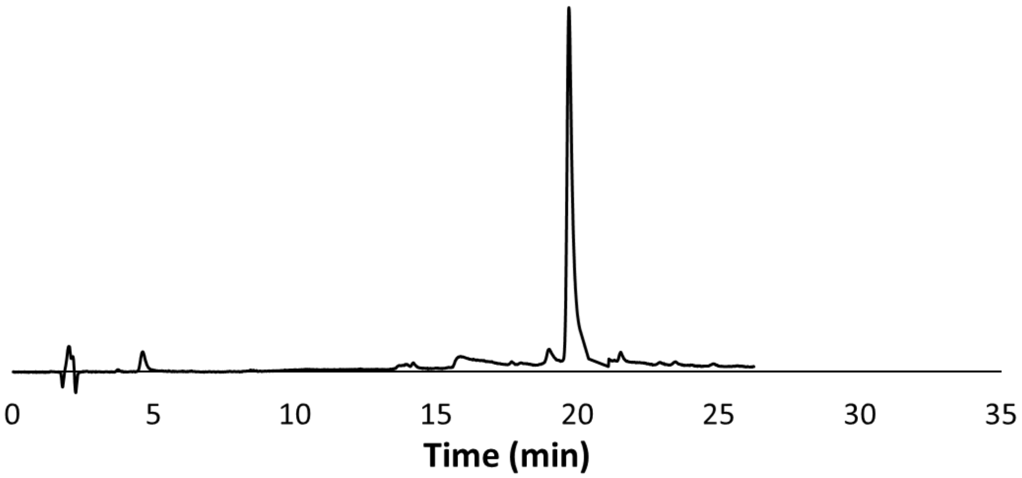

Figure S103. HPLC Chromatogram at 280 nm of compound **56** (r.t. = 19.7 min). Purity 95%

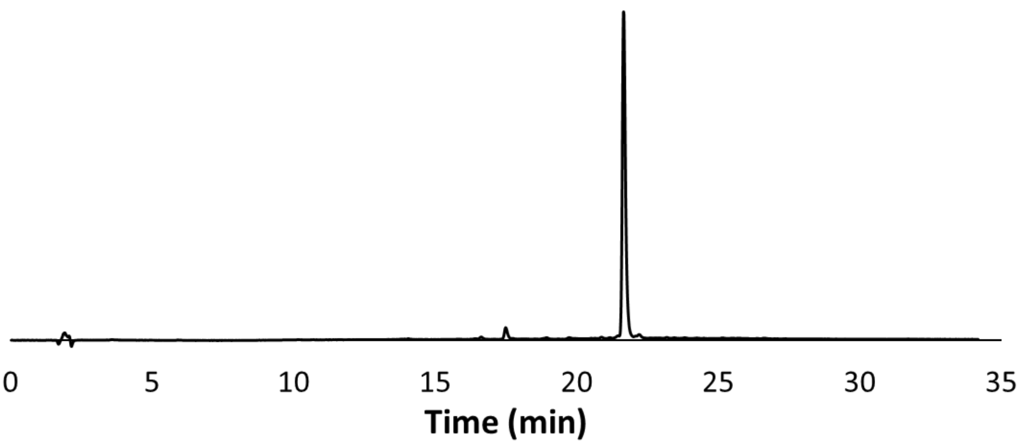

Figure S104. HPLC Chromatogram at 280 nm of compound **57** (r.t. = 21.6 min). Purity 97%

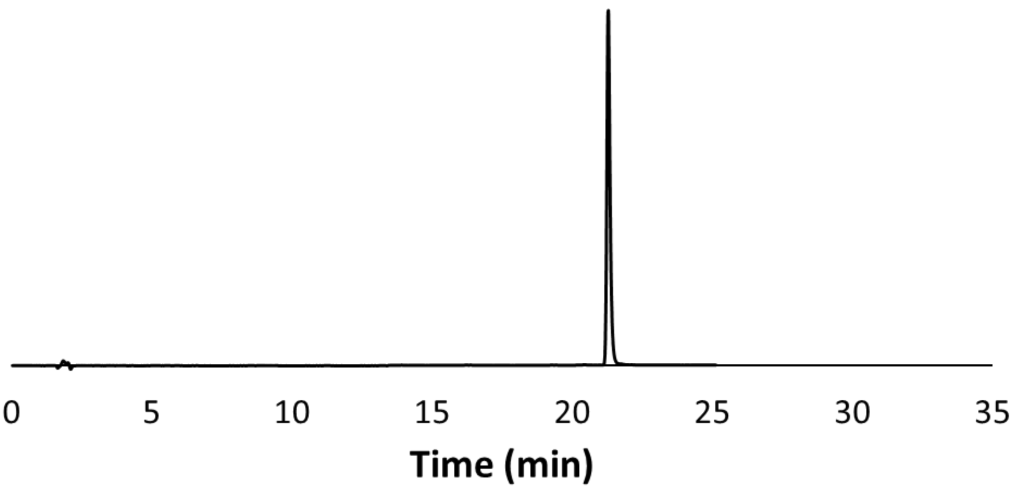

Figure S105. HPLC Chromatogram at 280 nm of compound **58** (r.t. = 21.3 min). Purity >99%

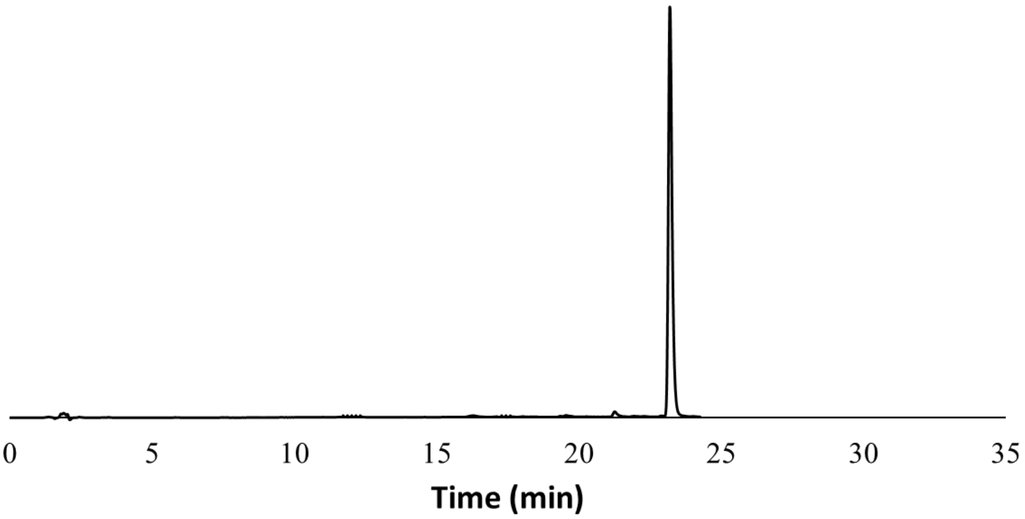

Figure S106. HPLC Chromatogram at 280 nm of compound **59** (r.t. = 23.2 min). Purity 98%

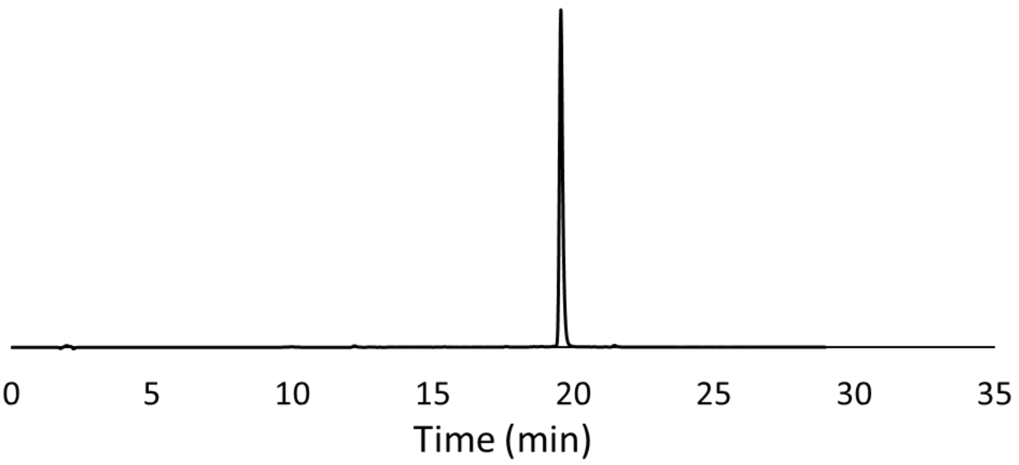

Figure S107. HPLC Chromatogram at 280 nm of compound **60** (r.t. = 19.6 min). Purity >99%

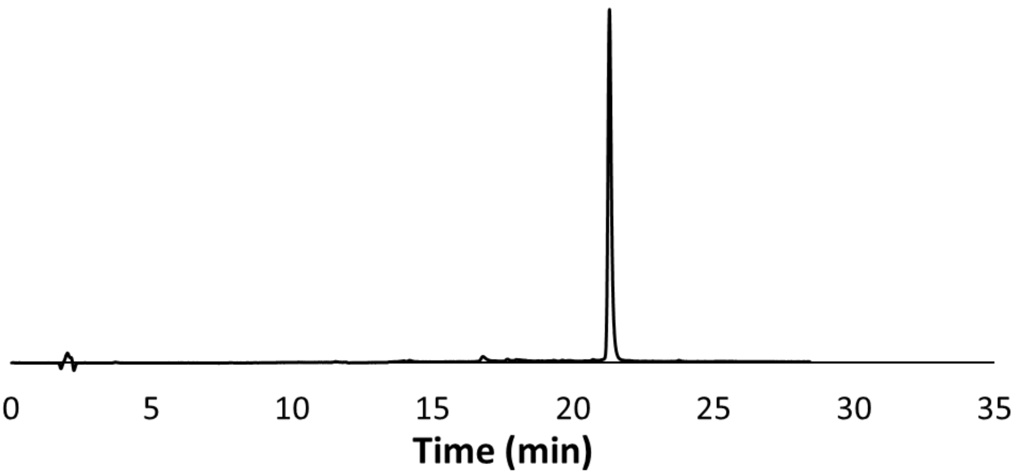

Figure S108. HPLC Chromatogram at 280 nm of compound **61** (r.t. = 21.3 min). Purity 99%

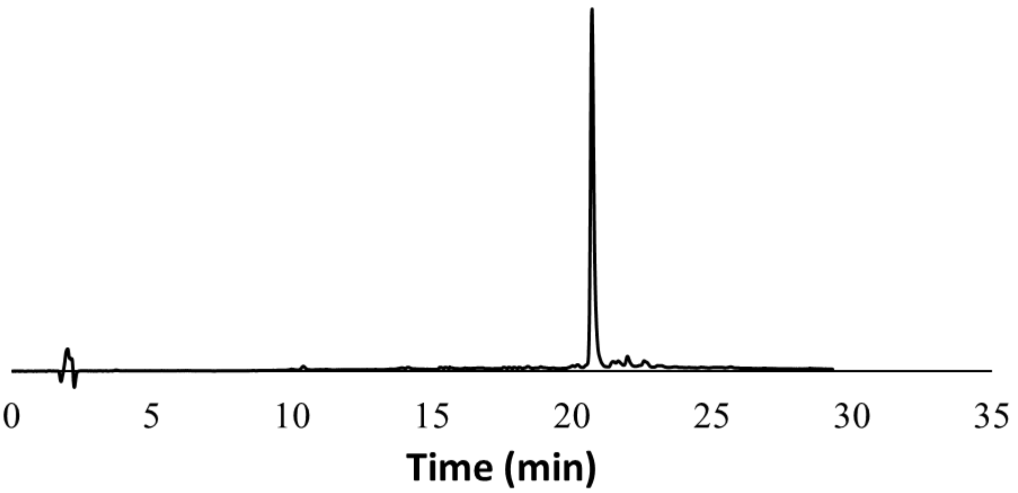

Figure S109. HPLC Chromatogram at 280 nm of compound **62a** (r.t. = 20.7 min). Purity 96%

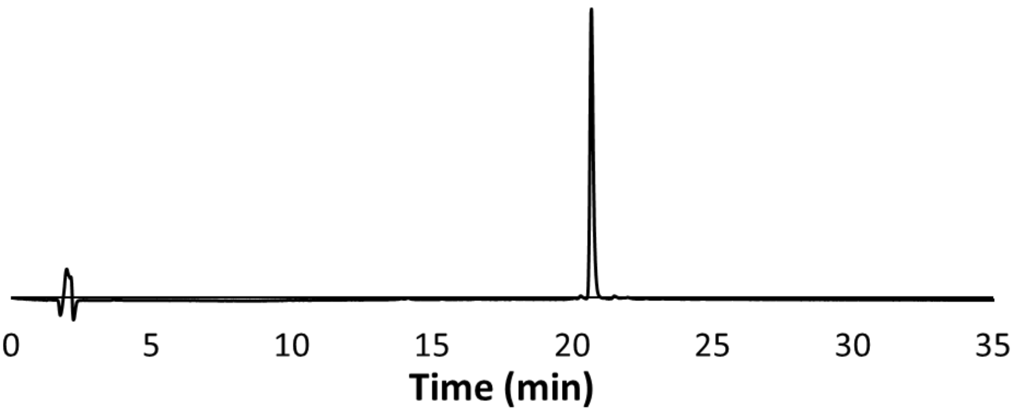

Figure S110. HPLC Chromatogram at 280 nm of compound **62b** (r.t. = 20.7 min). Purity >99%

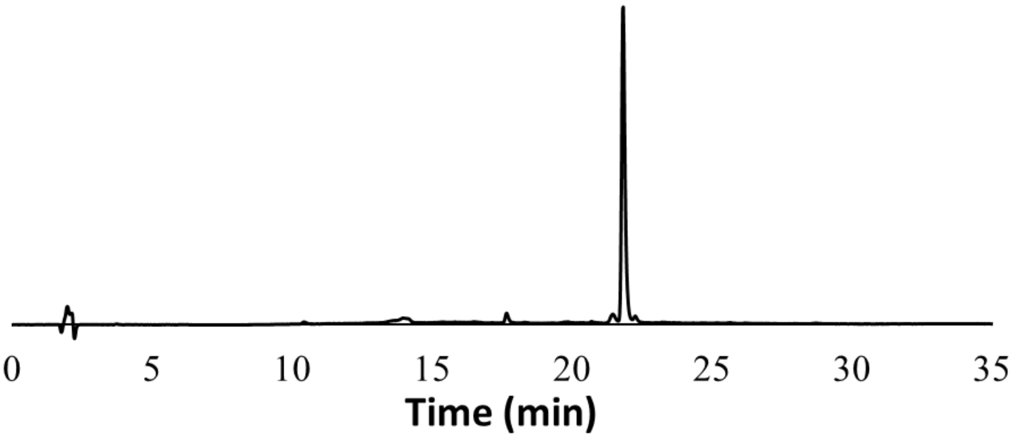

Figure S111. HPLC Chromatogram at 280 nm of compound **63a** (r.t. = 21.8 min). Purity 97%

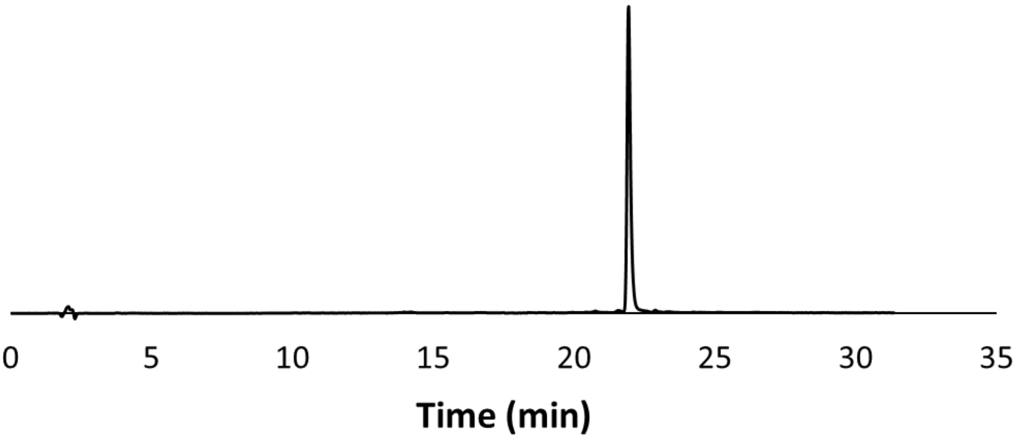

Figure S112. HPLC Chromatogram at 280 nm of compound **63b** (r.t. = 21.9 min). Purity >99%

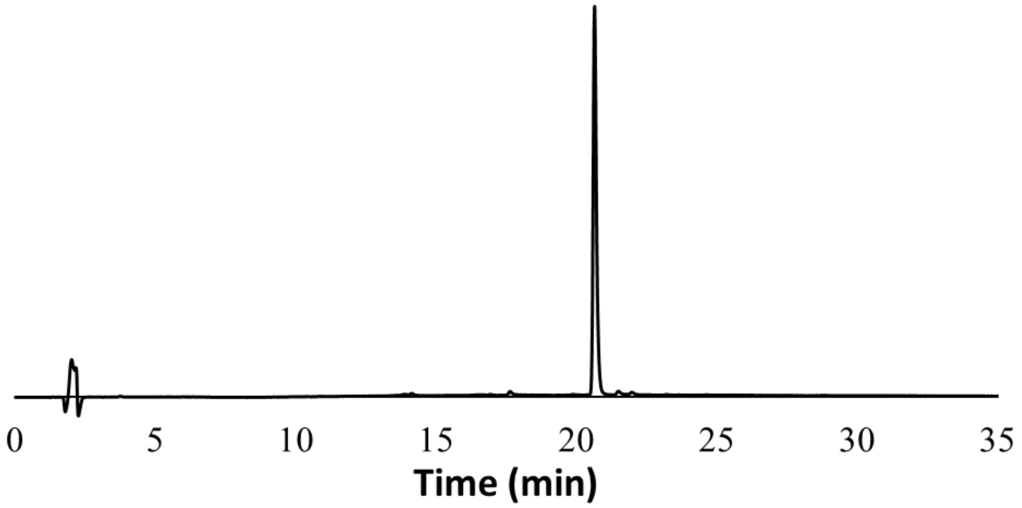

Figure S113. HPLC Chromatogram at 280 nm of compound **64a** (r.t. = 20.7 min). Purity 98%

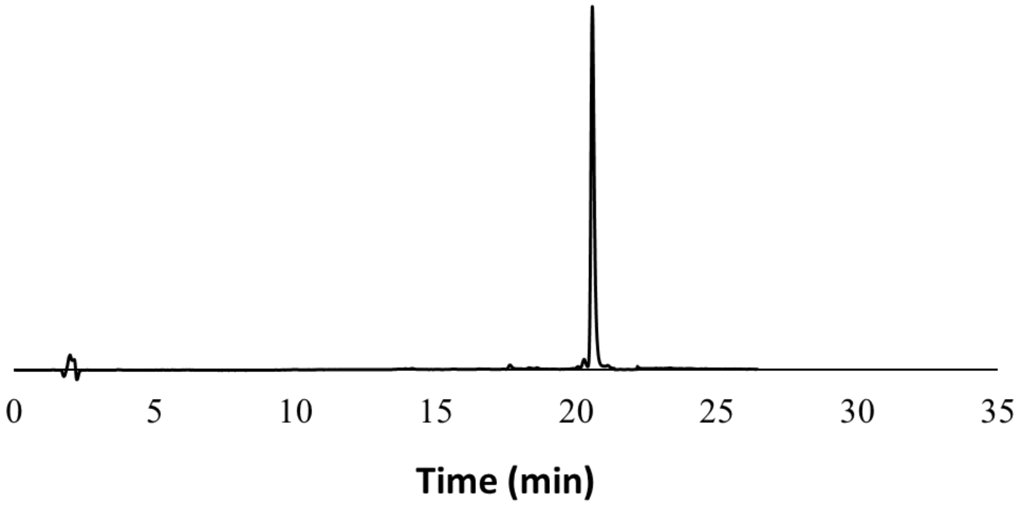

Figure S114. HPLC Chromatogram at 280 nm of compound **64b** (r.t. = 20.6 min). Purity 98%

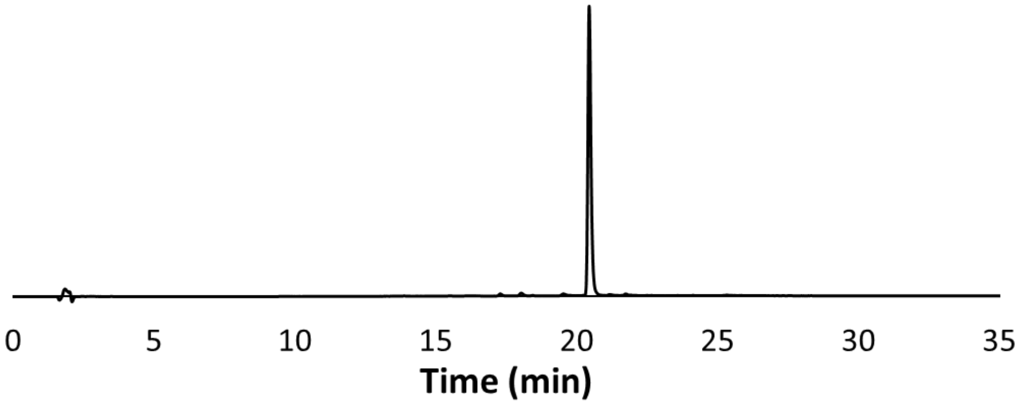

Figure S115. HPLC Chromatogram at 280 nm of compound **65a** (r.t. = 20.4 min). Purity 99%

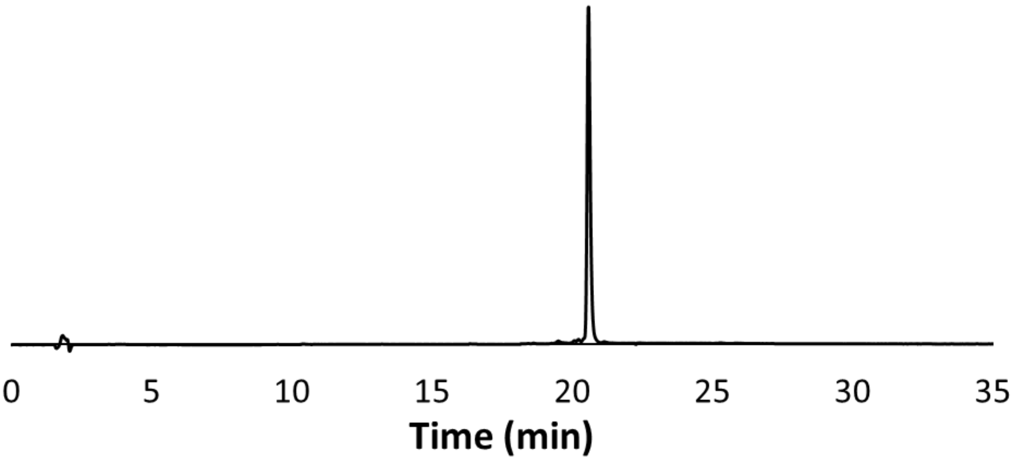

Figure S116. HPLC Chromatogram at 280 nm of compound **65b** (r.t. = 20.6 min). Purity >99%

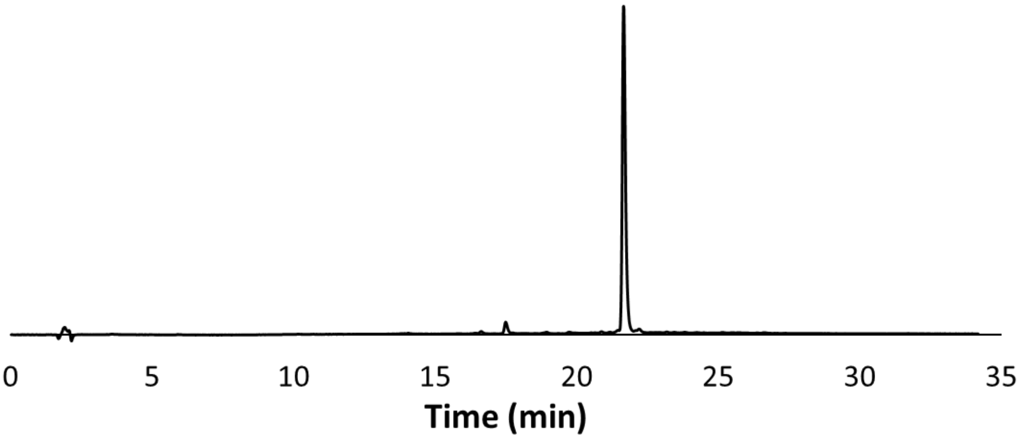

Figure S117. HPLC Chromatogram at 280 nm of compound **66a** (r.t. = 21.7 min). Purity 97%

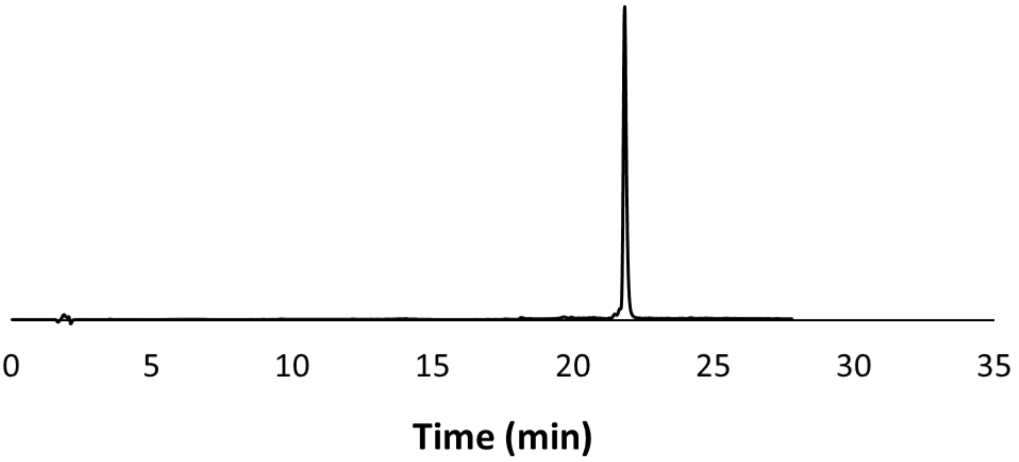

Figure S118. HPLC Chromatogram at 280 nm of compound **66b** (r.t. = 21.8 min). Purity >99%

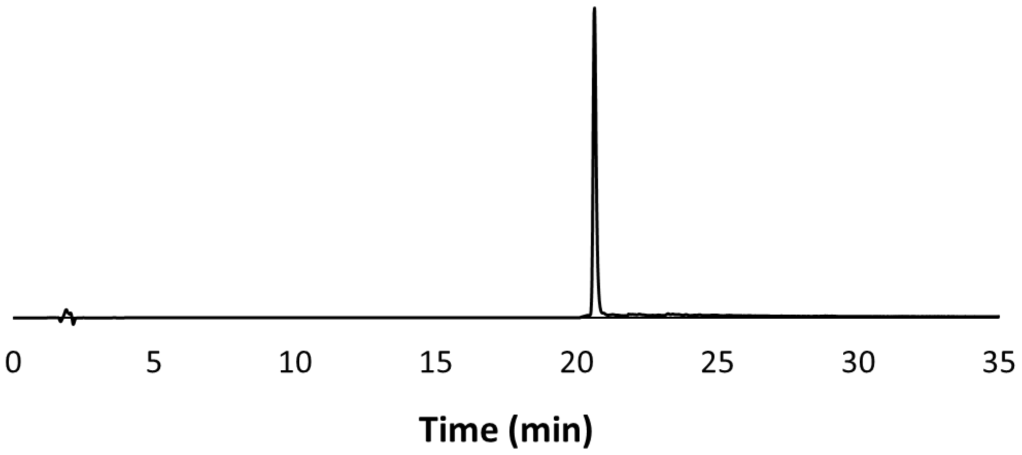

Figure S119. HPLC Chromatogram at 280 nm of compound **67a** (r.t. = 20.6 min). Purity >99%

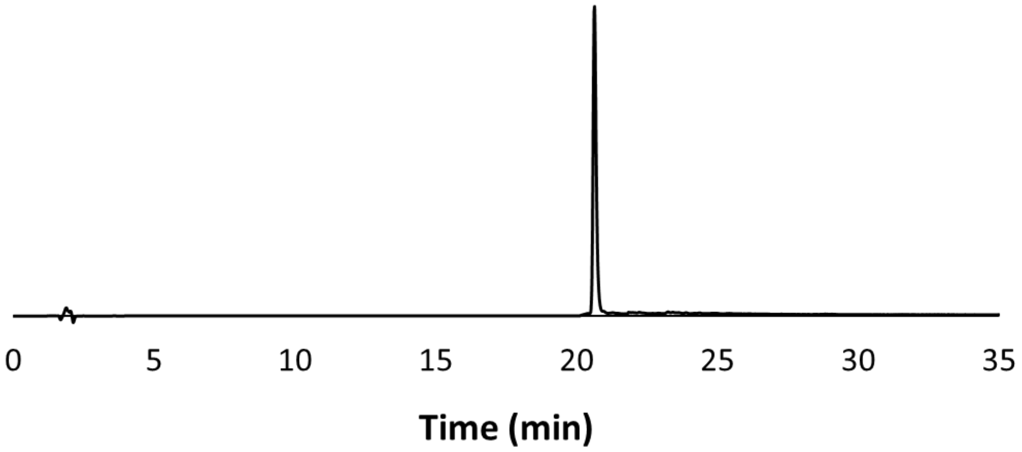

Figure S120. HPLC Chromatogram at 280 nm of compound **67b** (r.t. = 20.6 min). Purity >99%

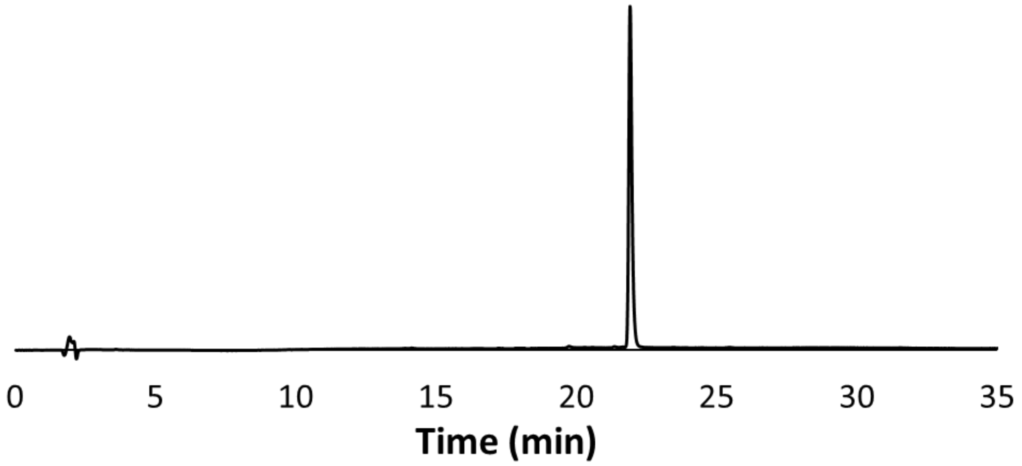

Figure S121. HPLC Chromatogram at 280 nm of compound **68a** (r.t. = 21.9 min). Purity >99%

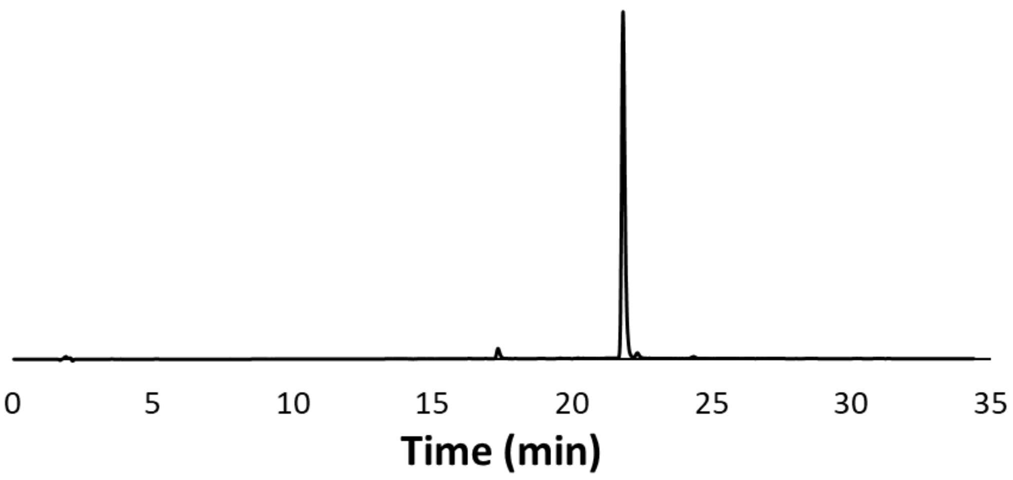

Figure S122. HPLC Chromatogram at 280 nm of compound **68b** (r.t. = 21.8 min). Purity 97%

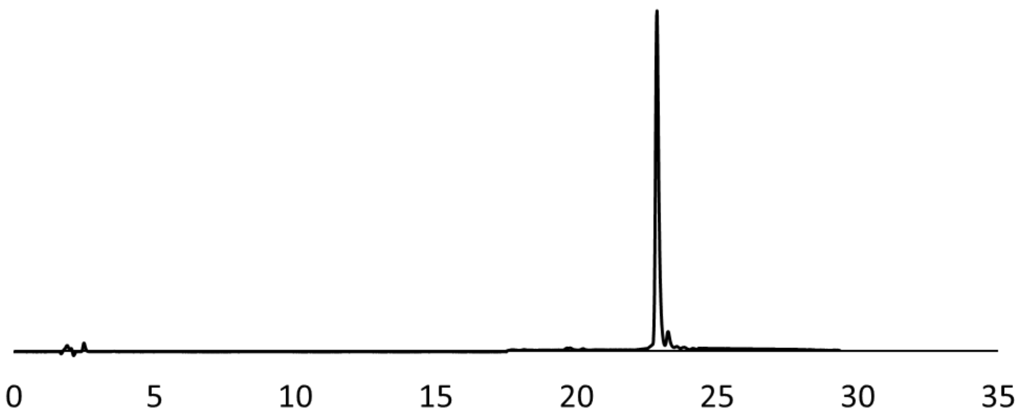

Figure S123. HPLC Chromatogram at 280 nm of compound **69a** (r.t. = 22.9 min). Purity 97%

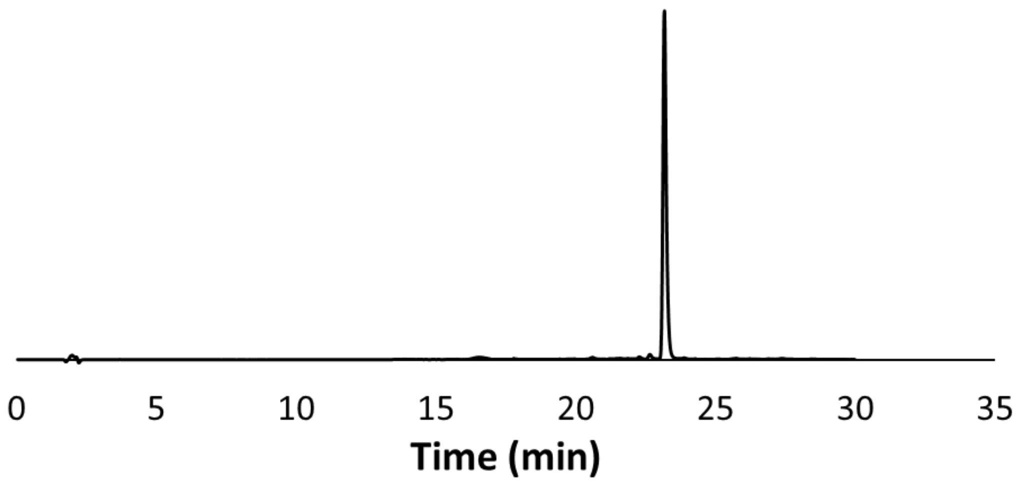

Figure S124. HPLC Chromatogram at 280 nm of compound **69b** (r.t. = 23.2 min). Purity 98%

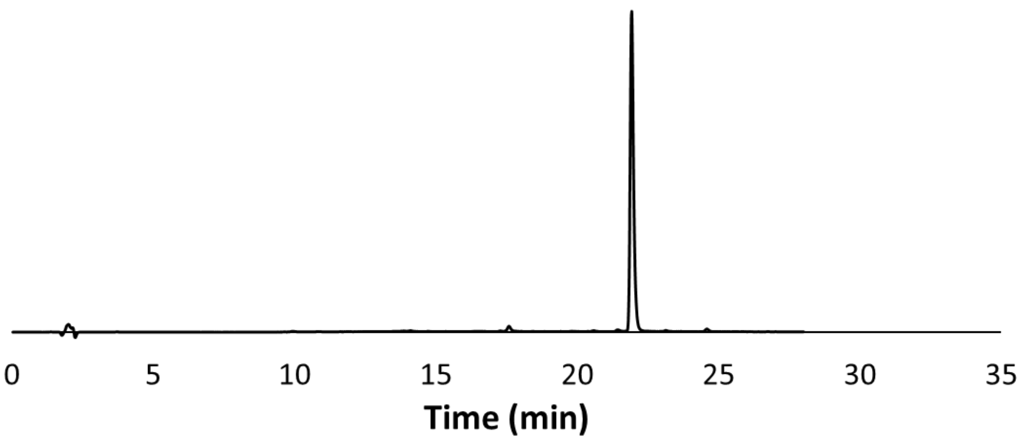

Figure S125. HPLC Chromatogram at 280 nm of compound **70a** (r.t. = 21.9 min). Purity 98%

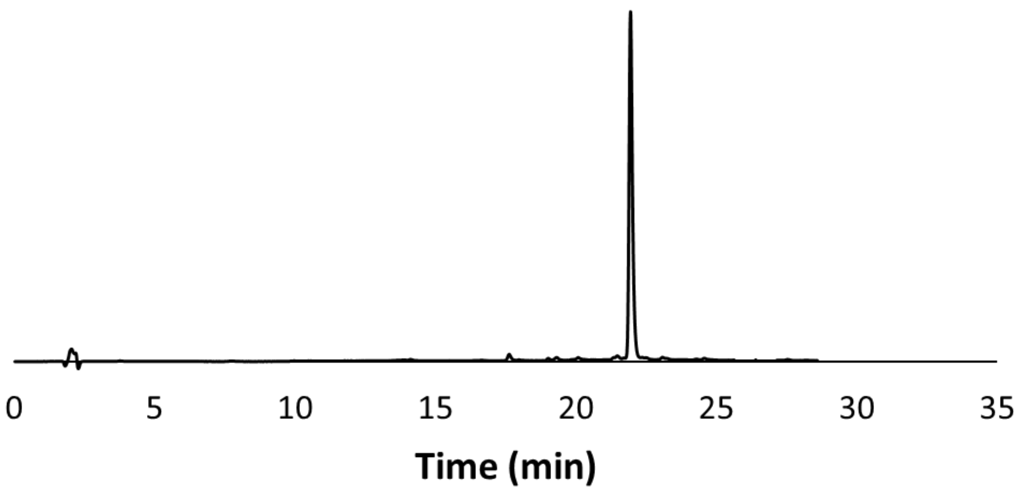

Figure S126. HPLC Chromatogram at 280 nm of compound **70b** (r.t. = 21.9 min). Purity 98%

3. Quiral HPLC chromatograms

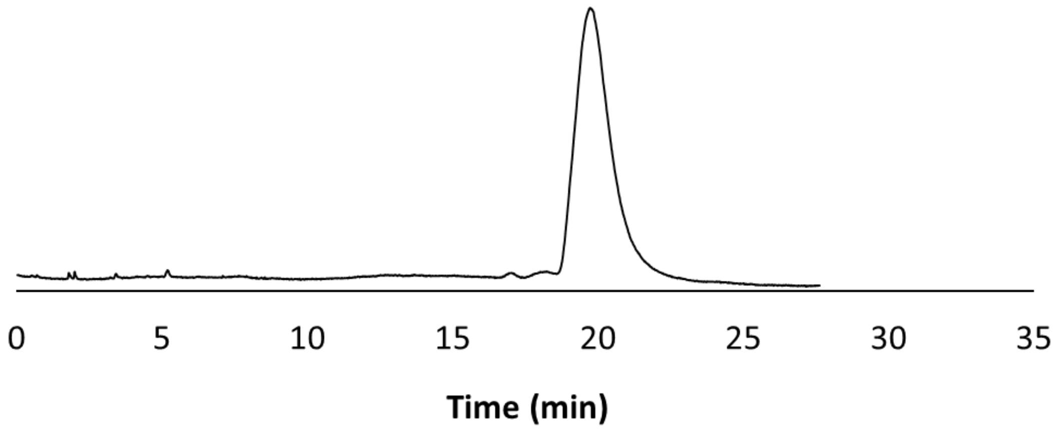

Figure S127 Quiral HPLC chromatogram at 280 nm of compound (+)-**66a** (r.t. = 19.7 min). Purity > 99%

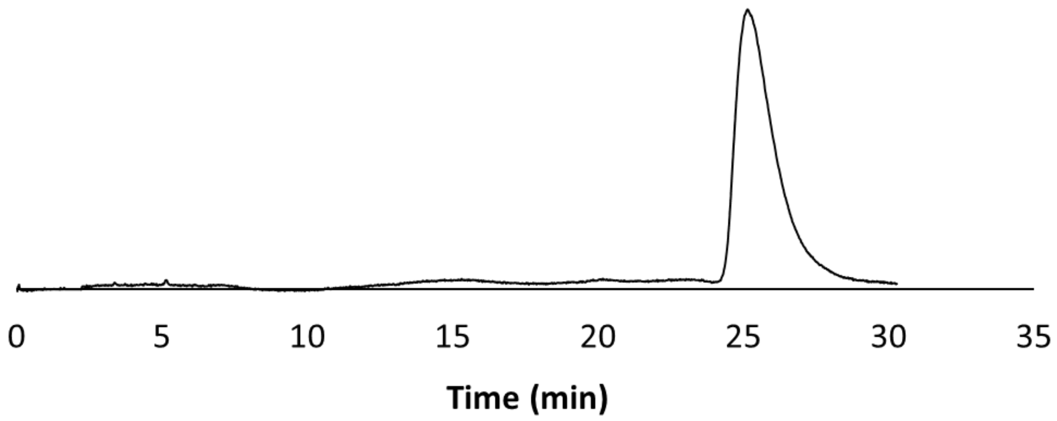

Figure S128 Quiral HPLC chromatogram at 280 nm of compound (−)-**66a** (r.t. = 25.5 min). Purity > 99%

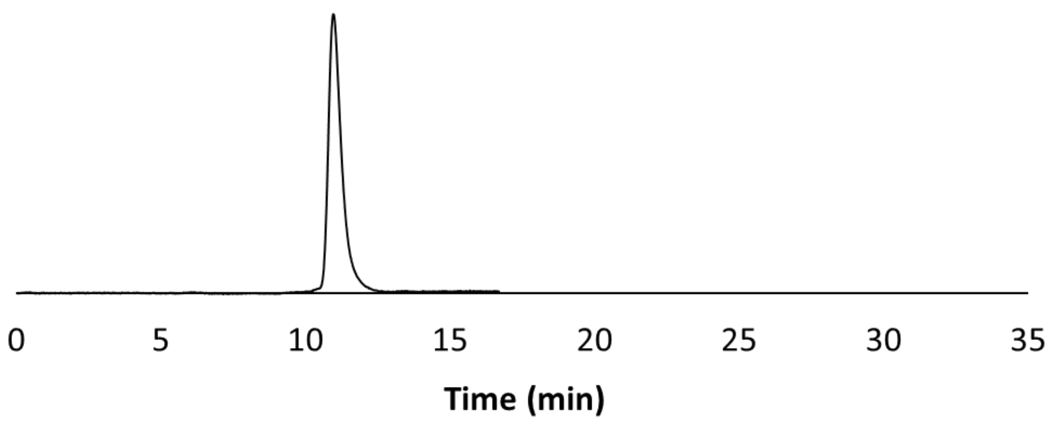

Figure S129 Quiral HPLC chromatogram at 280 nm of compound (+)-**68a** (r.t. = 10.9 min). Purity > 99%

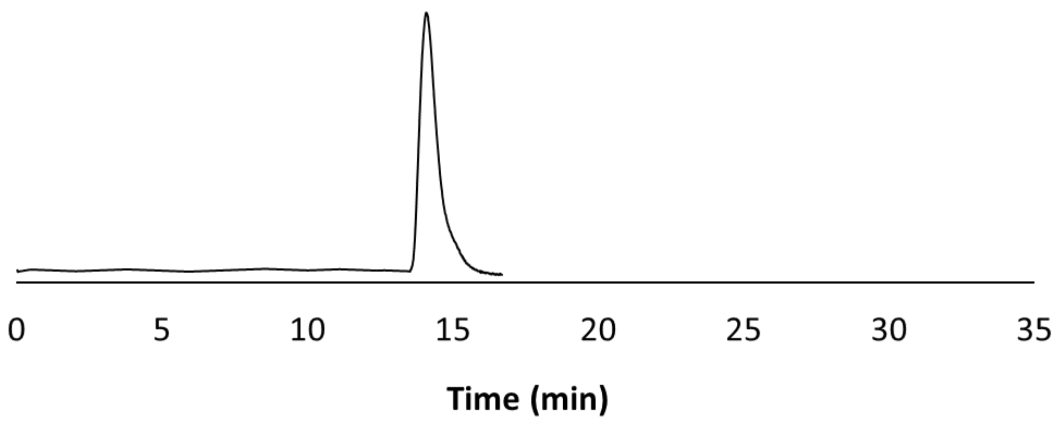

Figure S130 Quiral HPLC chromatogram at 280 nm of compound (−)-**68a** (r.t. = 14.1 min). Purity > 99%

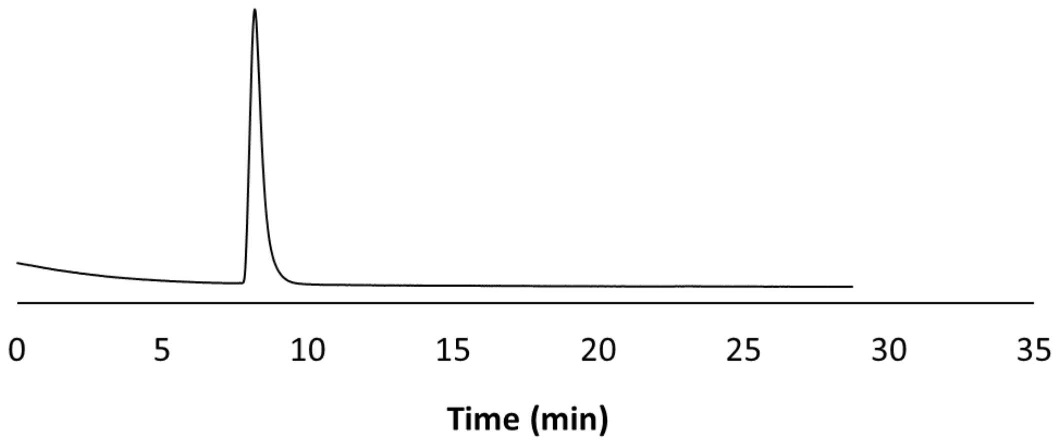

Figure S131 Quiral HPLC chromatogram at 280 nm of compound (+)-**68b** (r.t. = 8.2 min). Purity > 99%

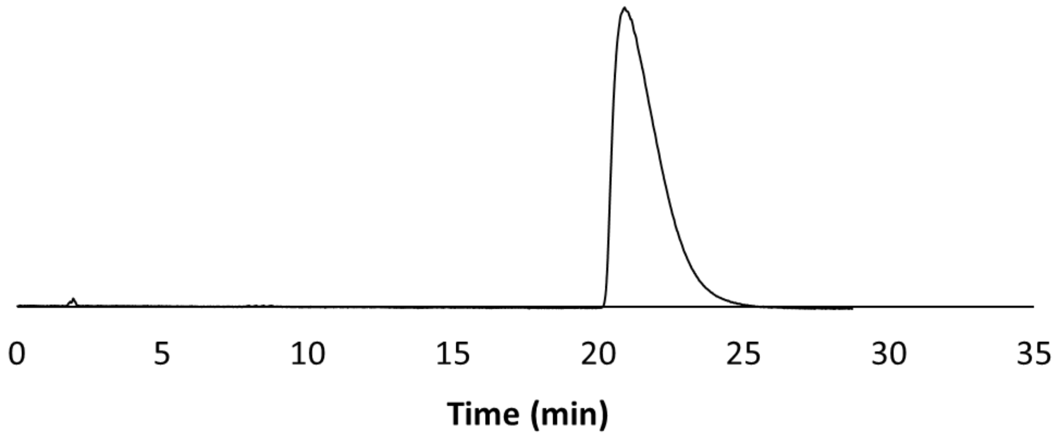

Figure S132 Quiral HPLC chromatogram at 280 nm of compound (−)-**68b** (r.t. = 20.9 min). Purity > 99%

4. Dose response curves against *h*LDHA and *h*LDHB

*h*LDHA

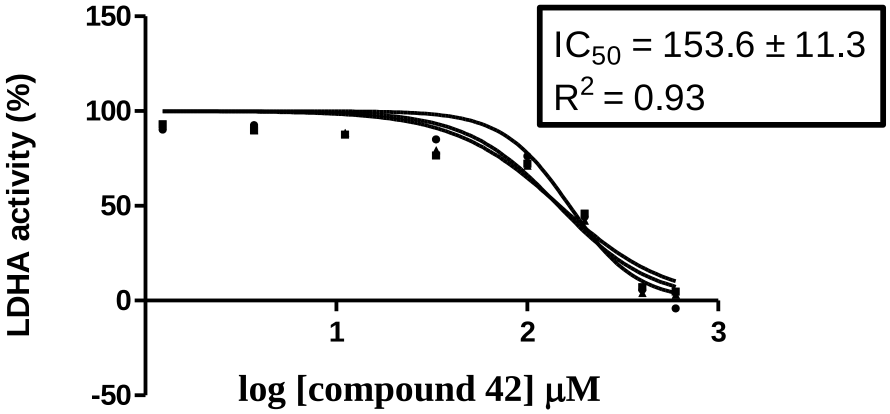

Figure S133. *h*LDHA inhibition curve of compound **42** (mean ± SD of n = 3 replicates).

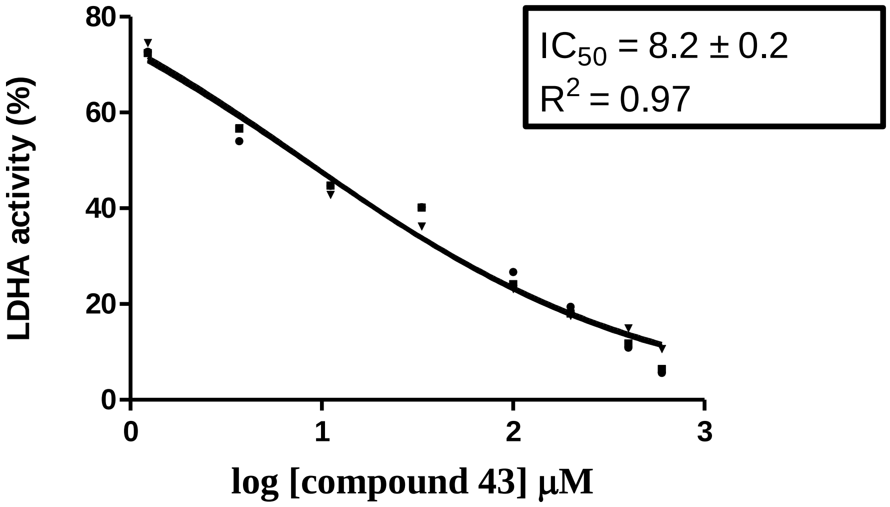

Figure S134. *h*LDHA inhibition curve of compound **43** (mean ± SD of n = 3 replicates).

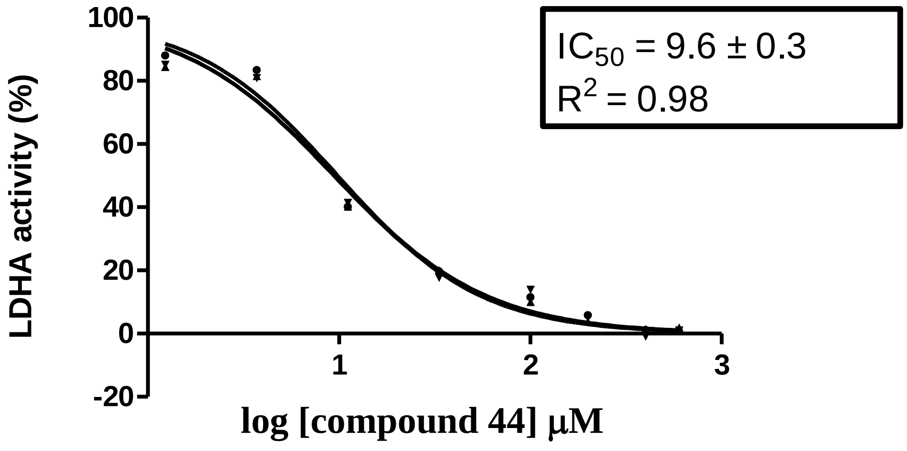

Figure S135. *h*LDHA inhibition curve of compound **44** (mean ± SD of n = 3 replicates).

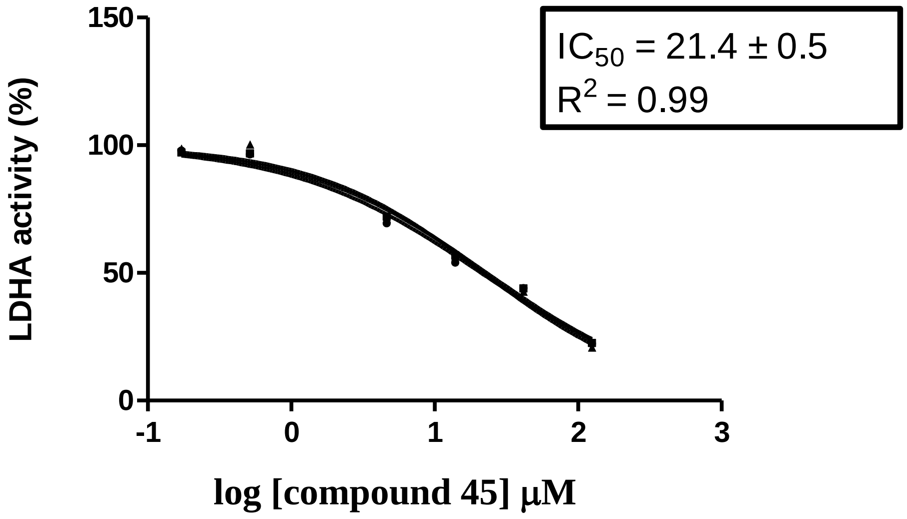

Figure S136. *h*LDHA inhibition curve of compound **45** (mean ± SD of n = 3 replicates).

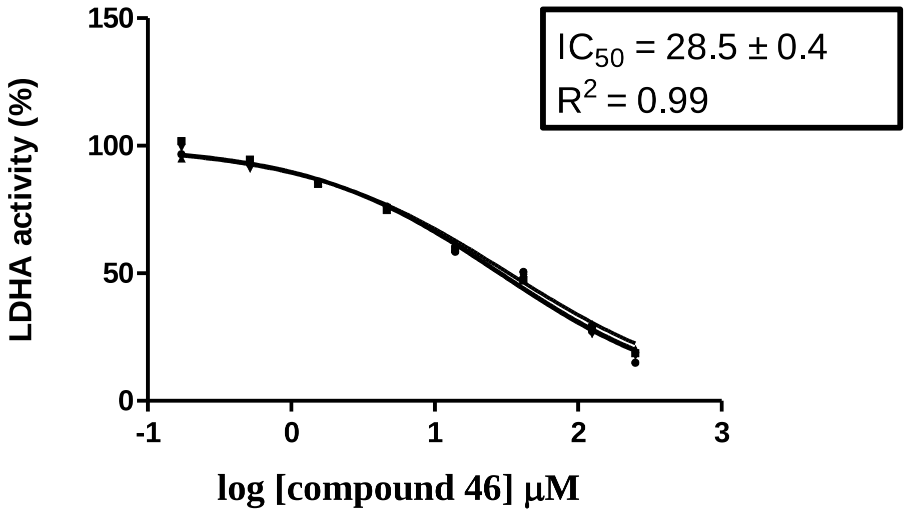

Figure S137. *h*LDHA inhibition curve of compound **46** (mean ± SD of n = 3 replicates).

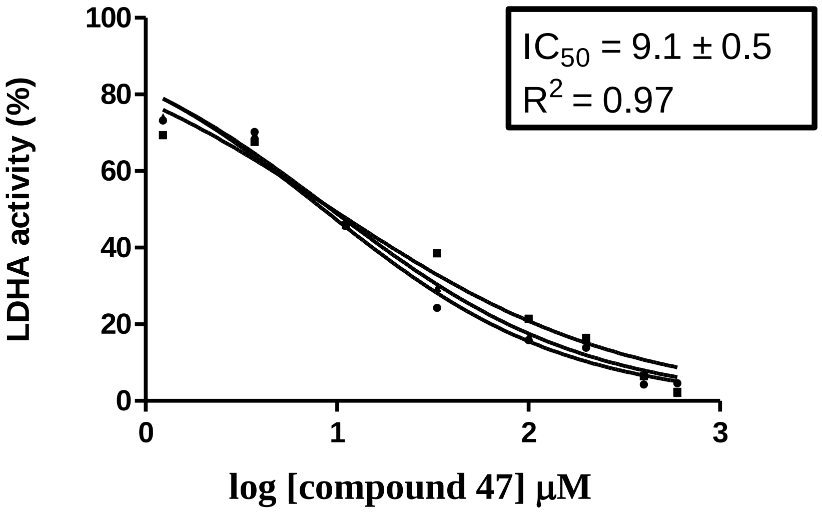

Figure S138. *h*LDHA inhibition curve of compound **47** (mean ± SD of n = 3 replicates).

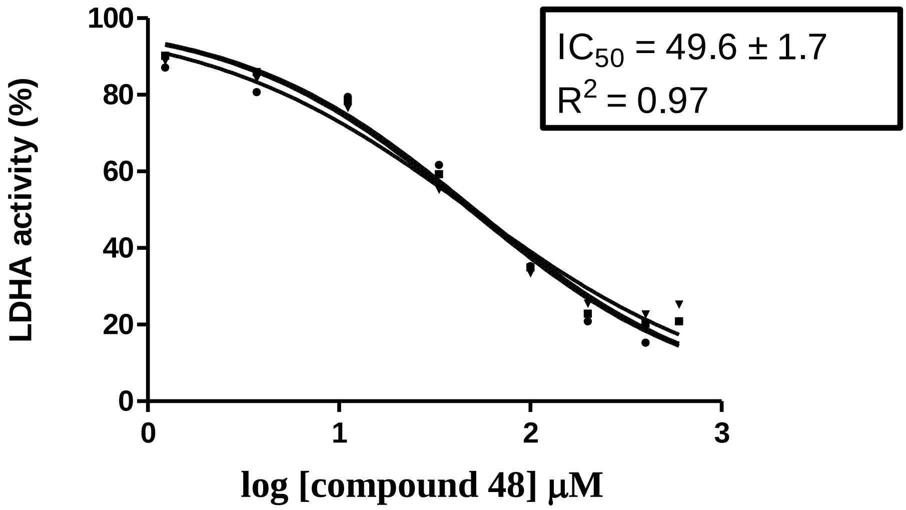

Figure S139. *h*LDHA inhibition curve of compound **48** (mean ± SD of n = 3 replicates).

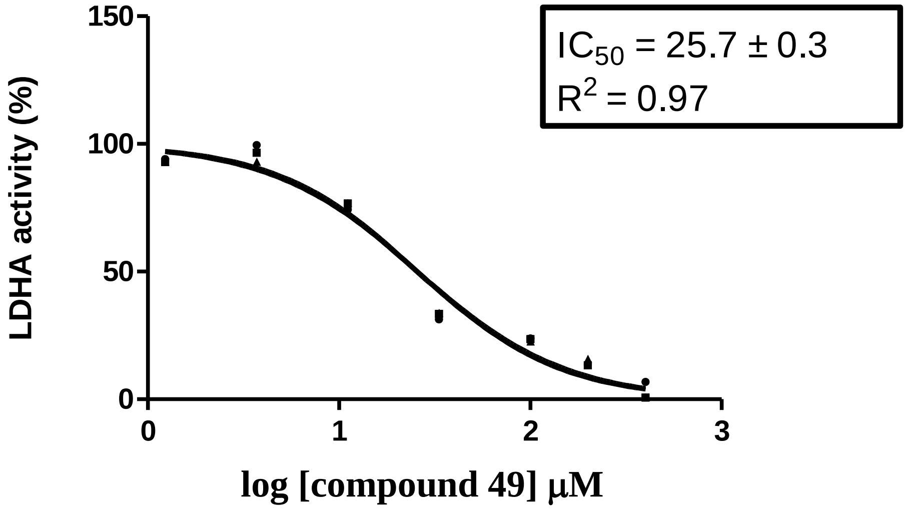

Figure S140. *h*LDHA inhibition curve of compound **49** (mean ± SD of n = 3 replicates).

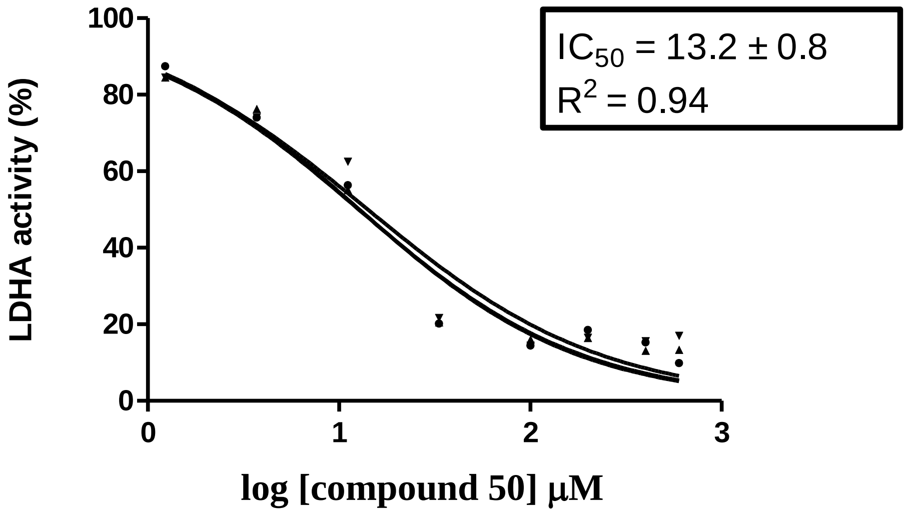

Figure S141. *h*LDHA inhibition curve of compound **50** (mean ± SD of n = 3 replicates).

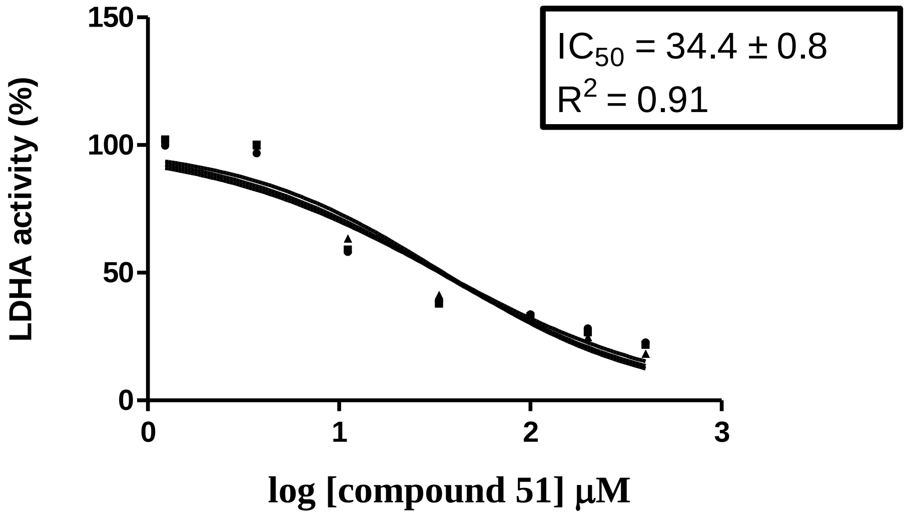

Figure S142. *h*LDHA inhibition curve of compound **51** (mean ± SD of n = 3 replicates).

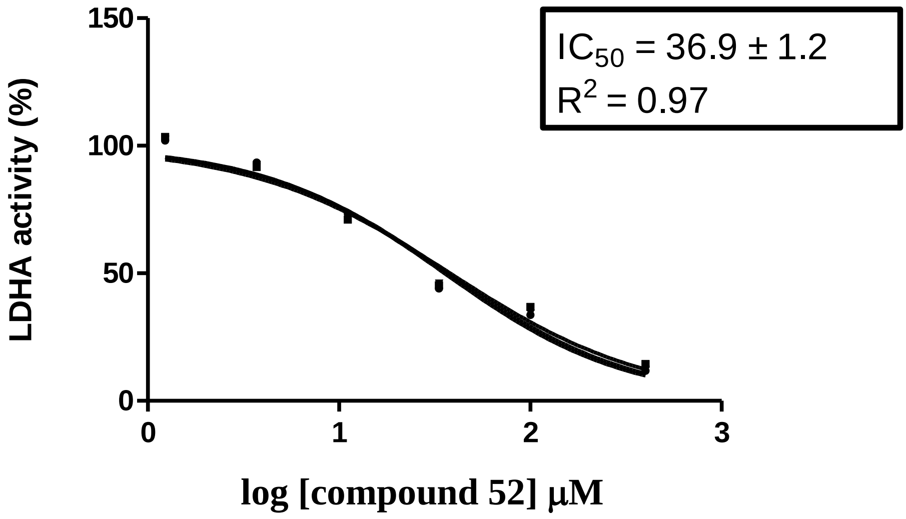

Figure S143. *h*LDHA inhibition curve of compound **52** (mean ± SD of n = 3 replicates).

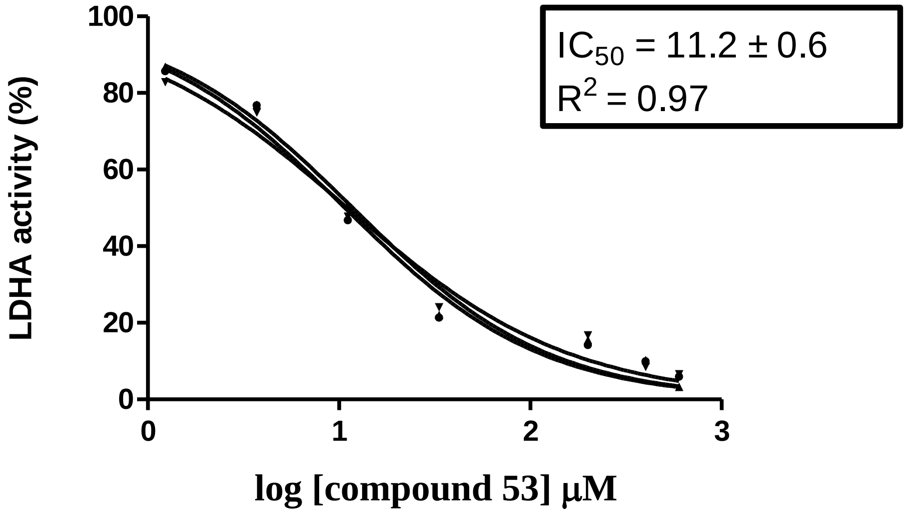

Figure S144. *h*LDHA inhibition curve of compound **53** (mean ± SD of n = 3 replicates).

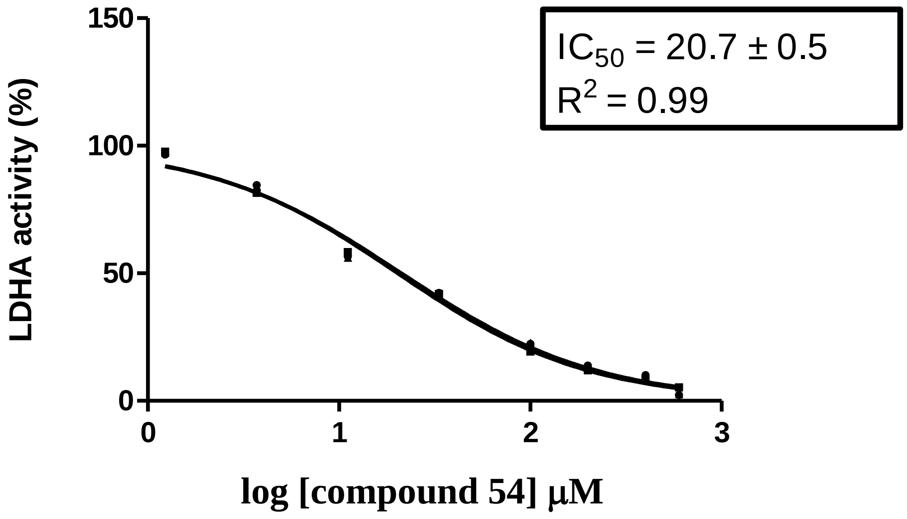

Figure S145. *h*LDHA inhibition curve of compound **54** (mean ± SD of n = 3 replicates).

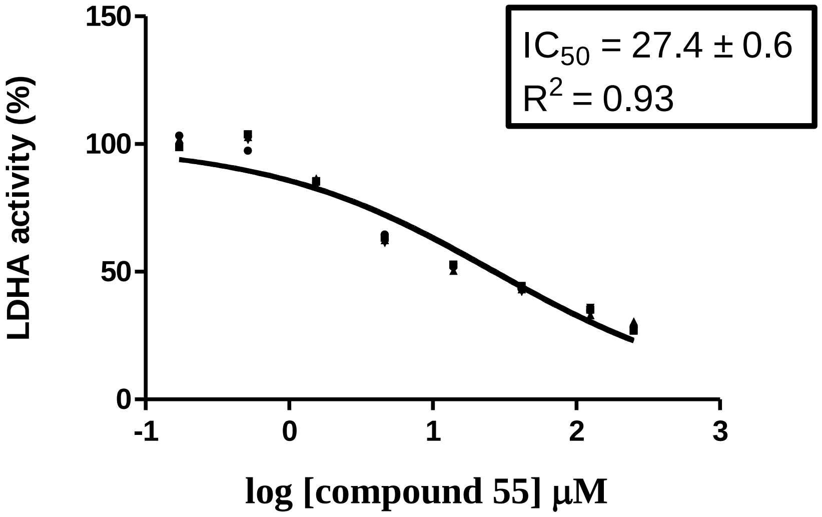

Figure S146. *h*LDHA inhibition curve of compound **55** (mean ± SD of n = 3 replicates).

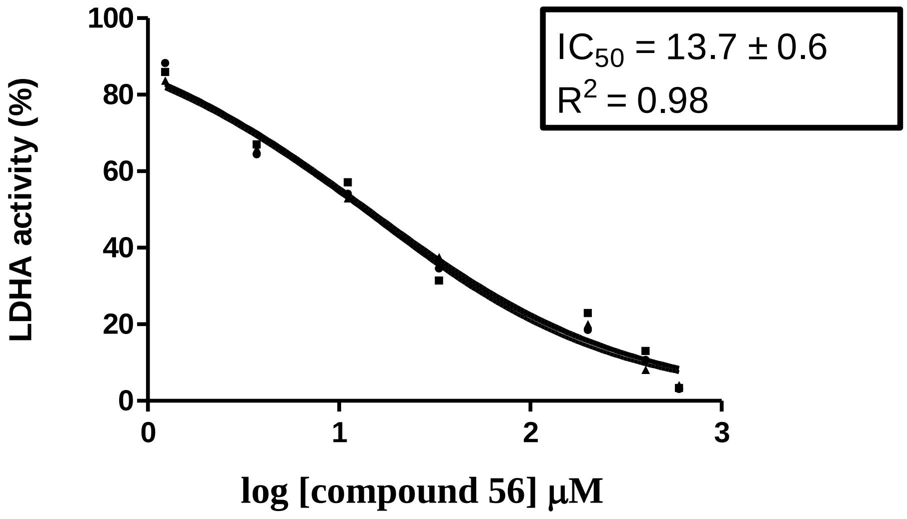

Figure S147. *h*LDHA inhibition curve of compound **56** (mean ± SD of n = 3 replicates).

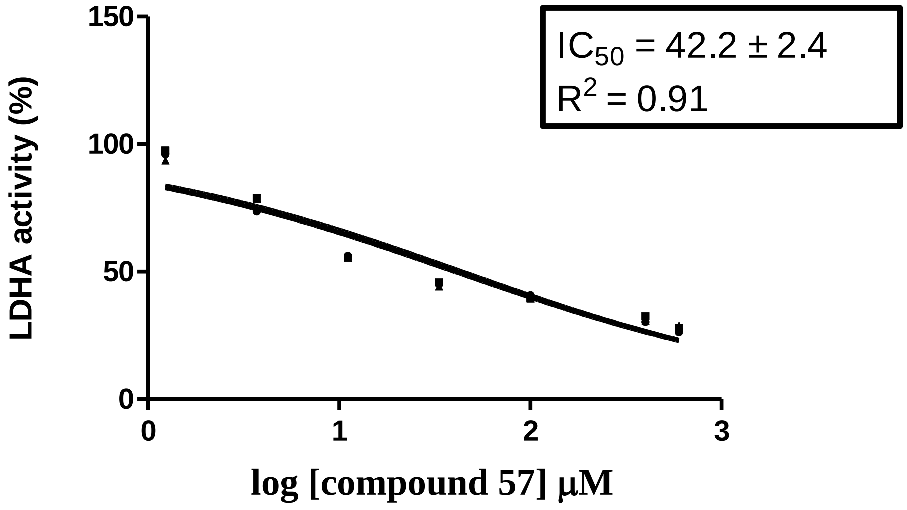

Figure S148. *h*LDHA inhibition curve of compound **57** (mean ± SD of n = 3 replicates).

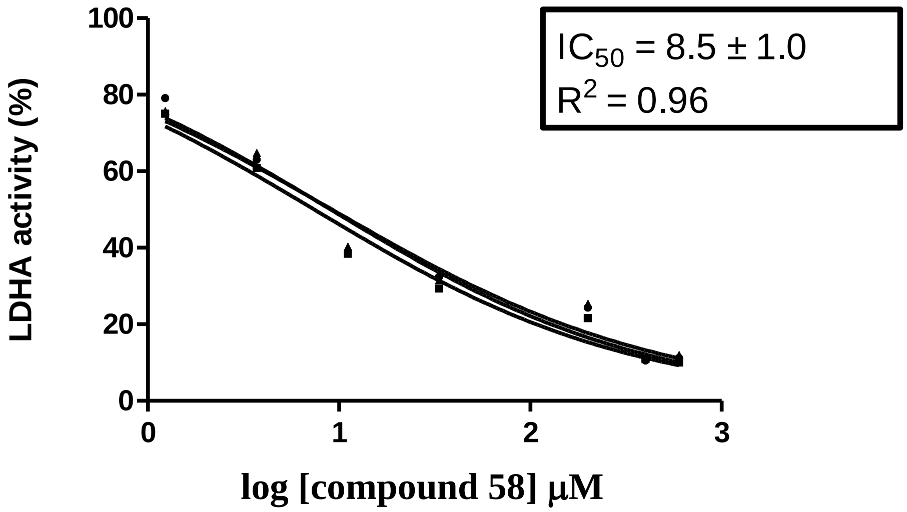

Figure S149. *h*LDHA inhibition curve of compound **58** (mean ± SD of n = 3 replicates).

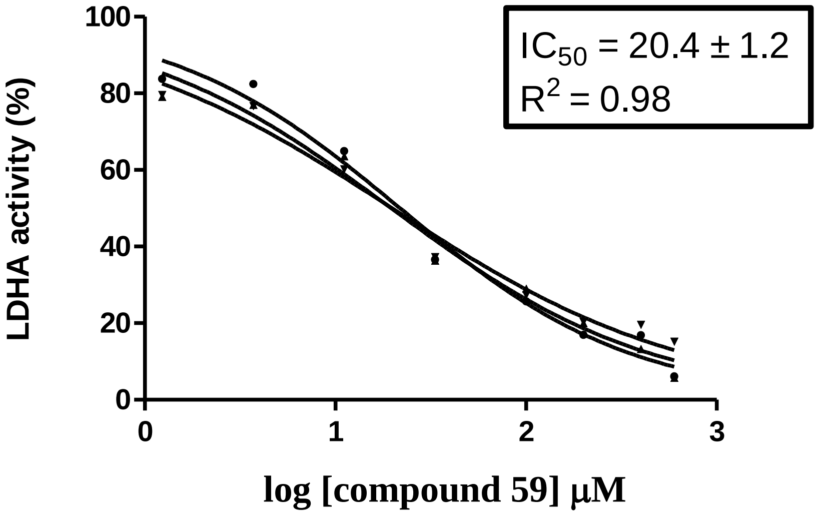

Figure S150. *h*LDHA inhibition curve of compound **59** (mean ± SD of n = 3 replicates).

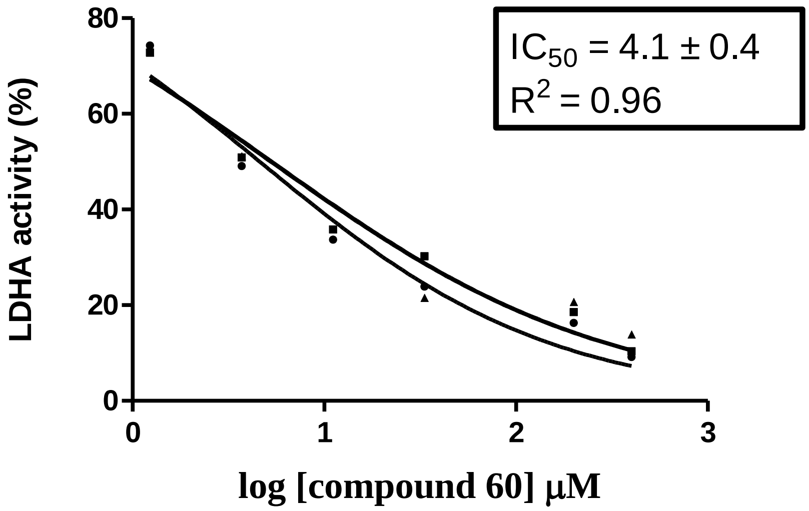

Figure S151. *h*LDHA inhibition curve of compound **60** (mean ± SD of n = 3 replicates).

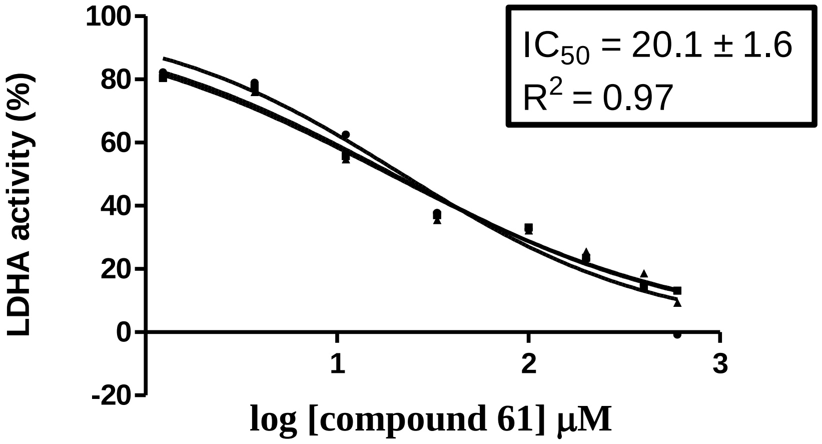

Figure S152. *h*LDHA inhibition curve of compound **61** (mean ± SD of n = 3 replicates).

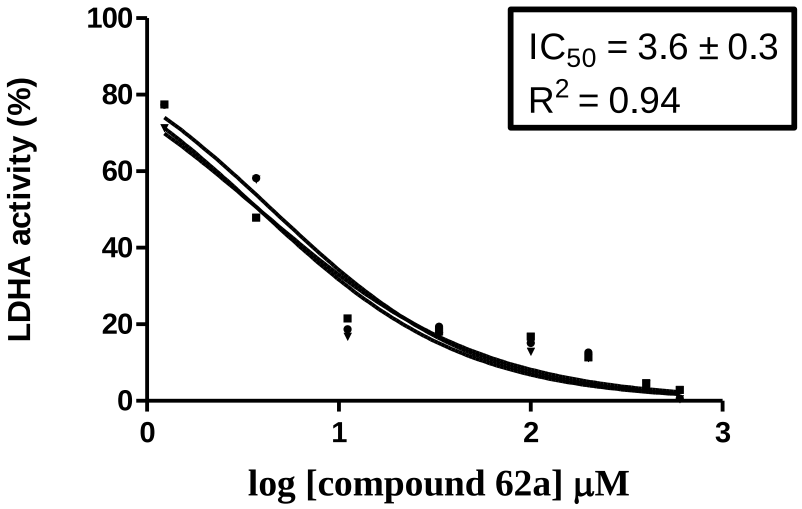

Figure S153. *h*LDHA inhibition curve of compound **62a** (mean ± SD of n = 3 replicates).

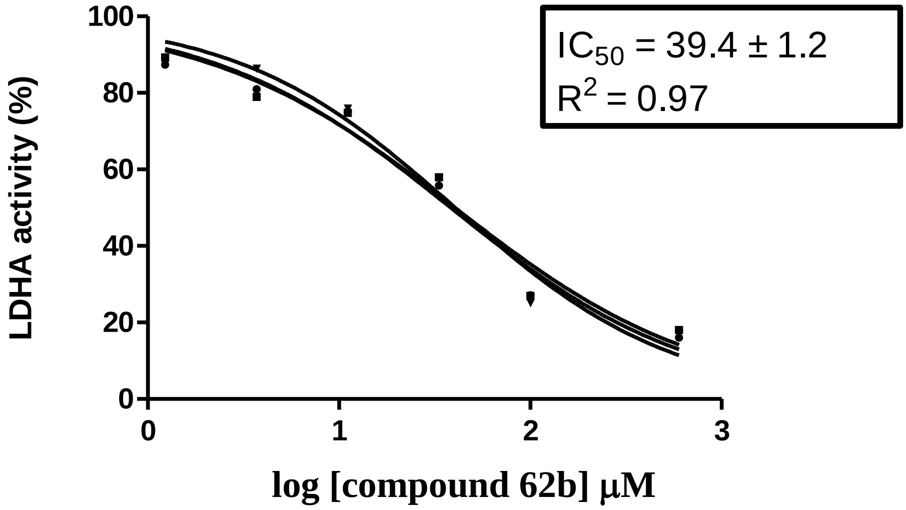

Figure S154. *h*LDHA inhibition curve of compound **62b** (mean ± SD of n = 3 replicates).

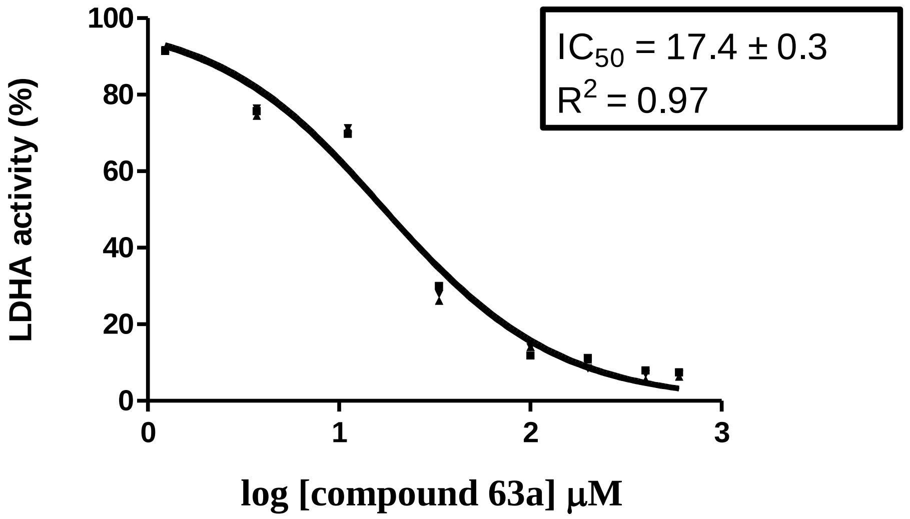

Figure S155. *h*LDHA inhibition curve of compound **63a** (mean ± SD of n = 3 replicates).

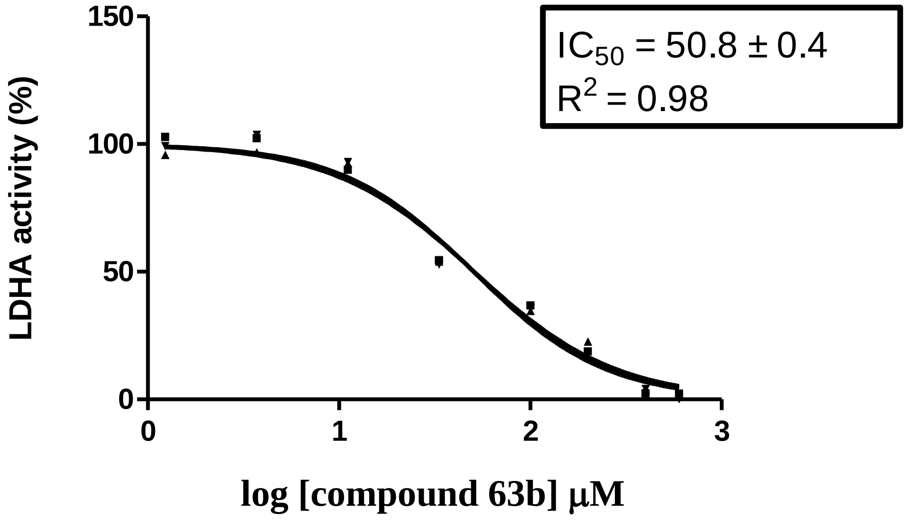

Figure S156. *h*LDHA inhibition curve of compound **63b** (mean ± SD of n = 3 replicates).

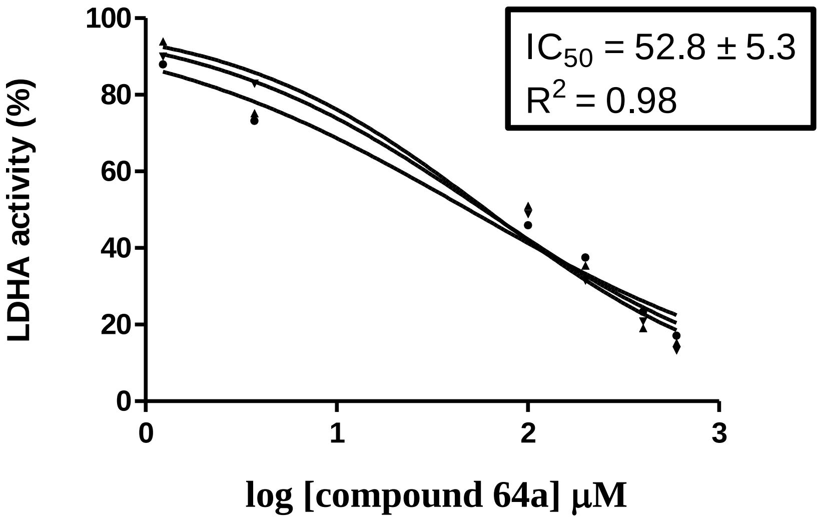

Figure S157. *h*LDHA inhibition curve of compound **64a** (mean ± SD of n = 3 replicates).

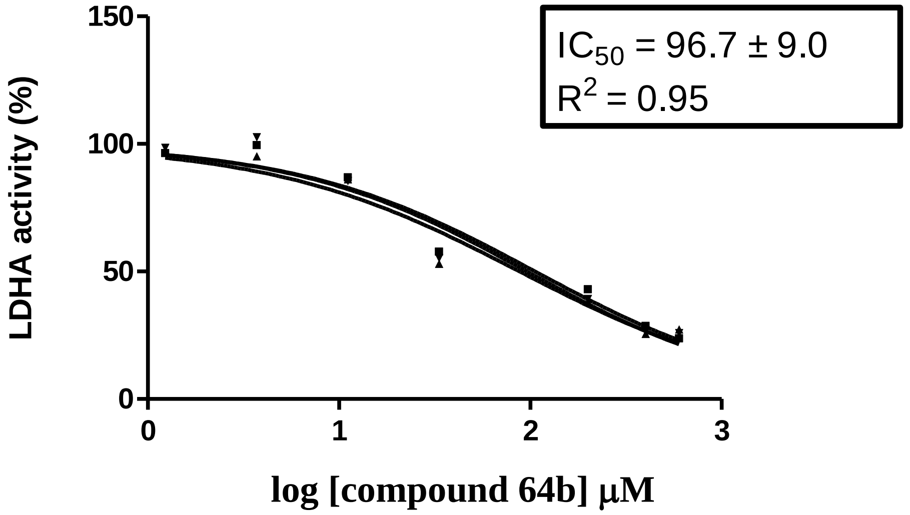

Figure S158. *h*LDHA inhibition curve of compound **64b** (mean ± SD of n = 3 replicates).

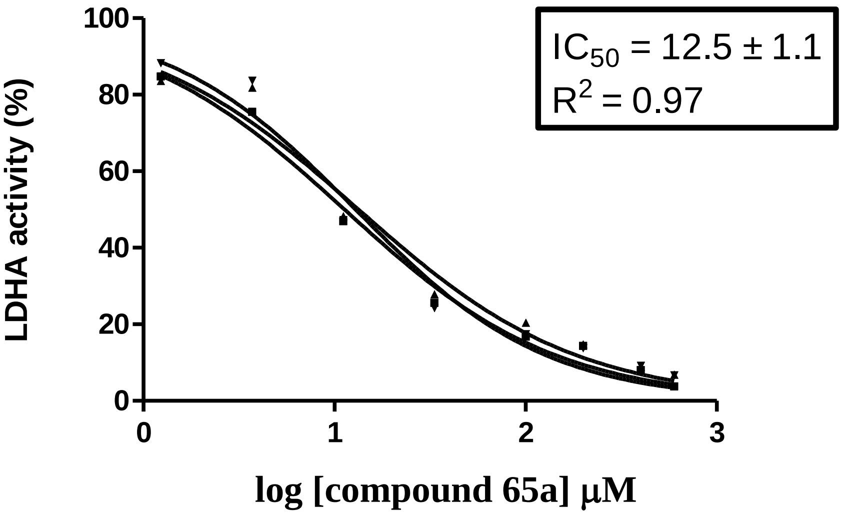

Figure S159. *h*LDHA inhibition curve of compound **65a** (mean ± SD of n = 3 replicates).

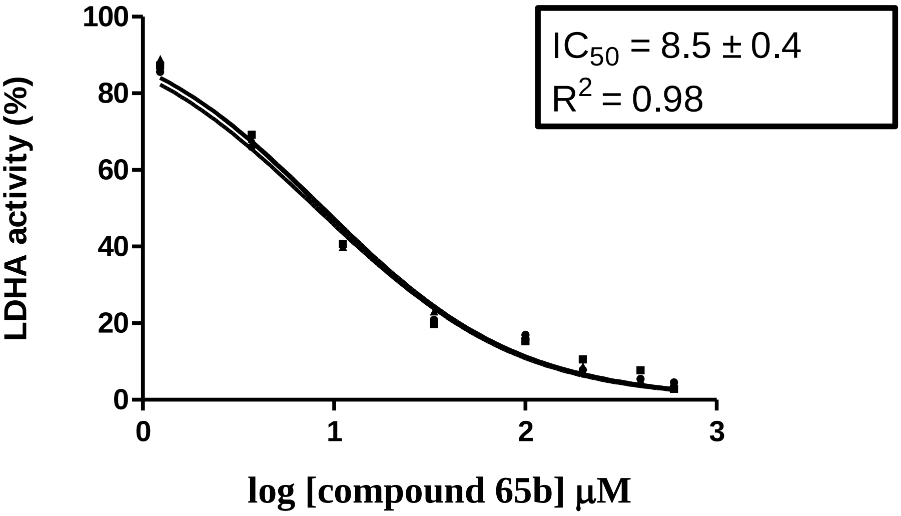

Figure S160. *h*LDHA inhibition curve of compound **65b** (mean ± SD of n = 3 replicates).

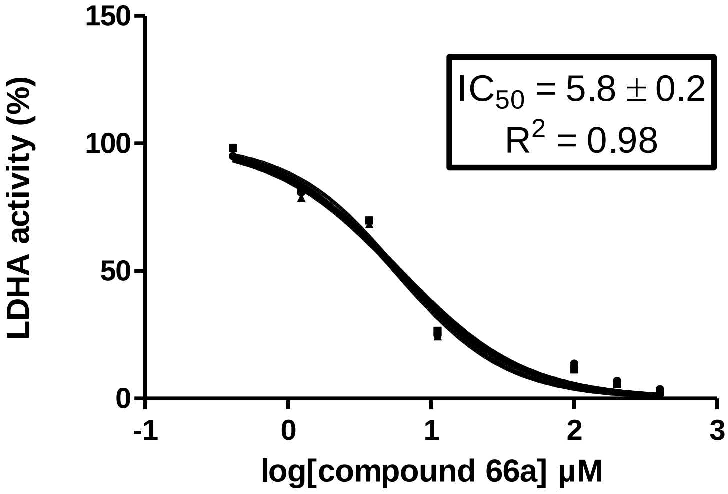

Figure S161. *h*LDHA inhibition curve of compound **66a** (mean ± SD of n = 3 replicates).

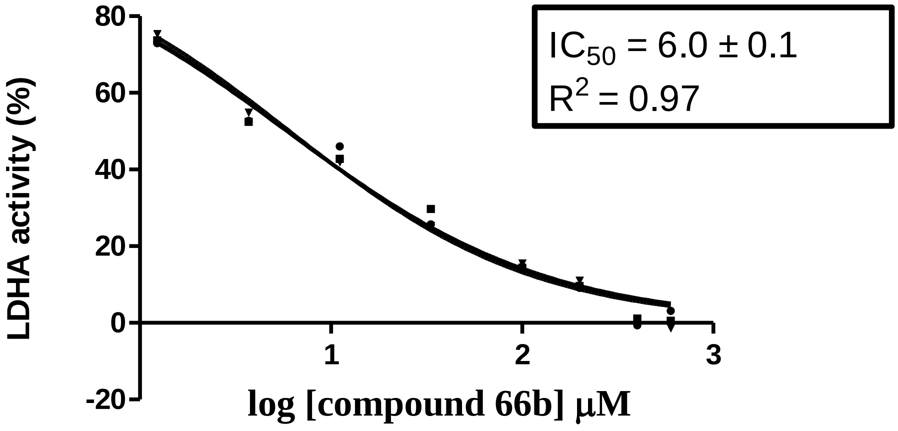

Figure S162. *h*LDHA inhibition curve of compound **66b** (mean ± SD of n = 3 replicates).

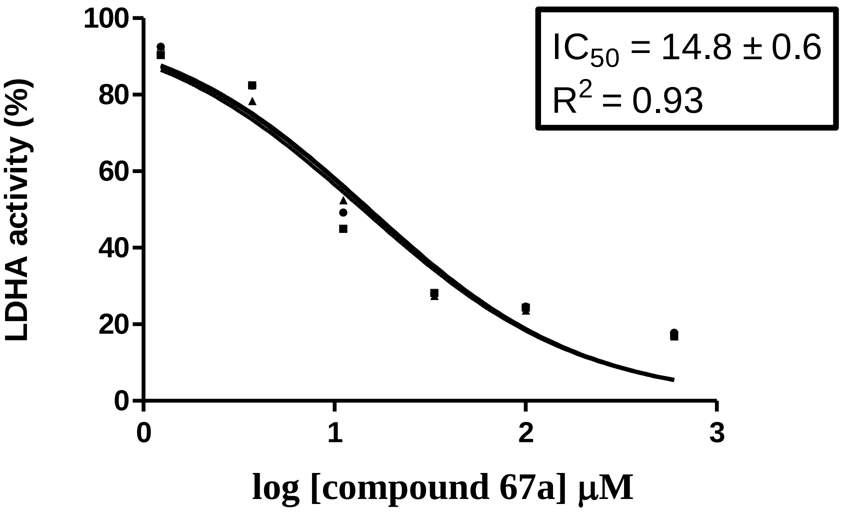

Figure S163. *h*LDHA inhibition curve of compound **67a** (mean ± SD of n = 3 replicates).

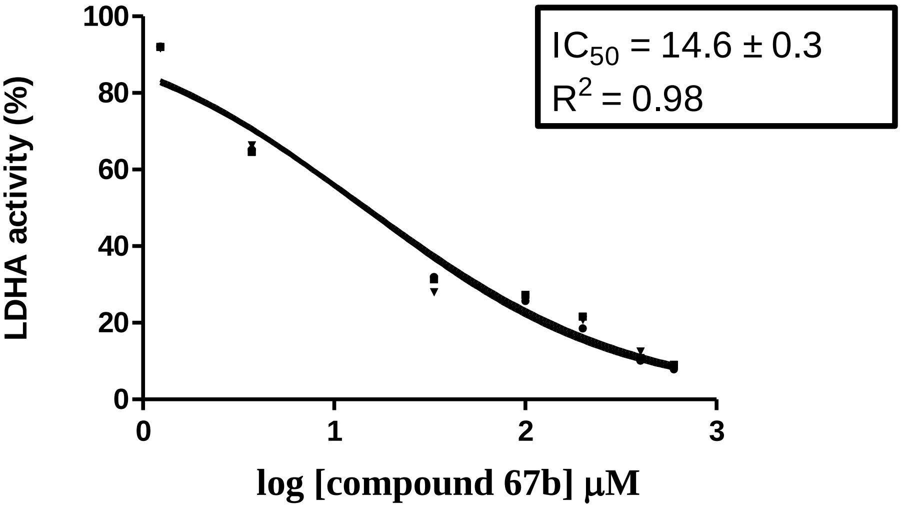

Figure S164. *h*LDHA inhibition curve of compound **67b** (mean ± SD of n = 3 replicates).

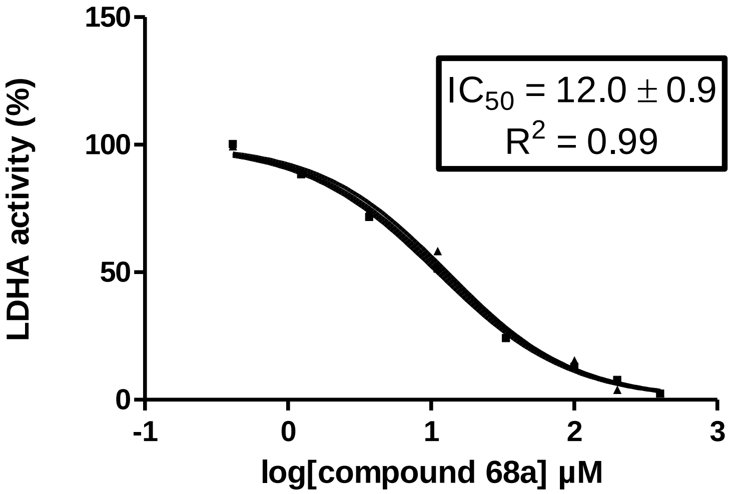

Figure S165. *h*LDHA inhibition curve of compound **68a** (mean ± SD of n = 3 replicates).

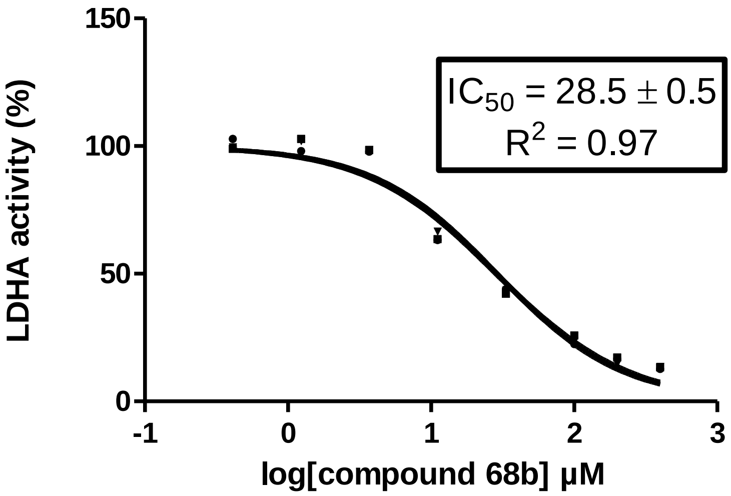

Figure S166. *h*LDHA inhibition curve of compound **68b** (mean ± SD of n = 3 replicates).

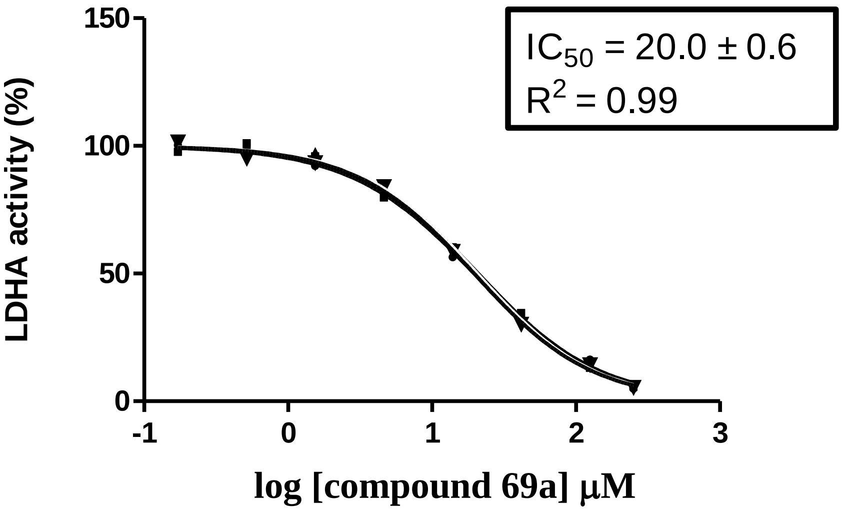

Figure S167. *h*LDHA inhibition curve of compound **69a** (mean ± SD of n = 3 replicates).

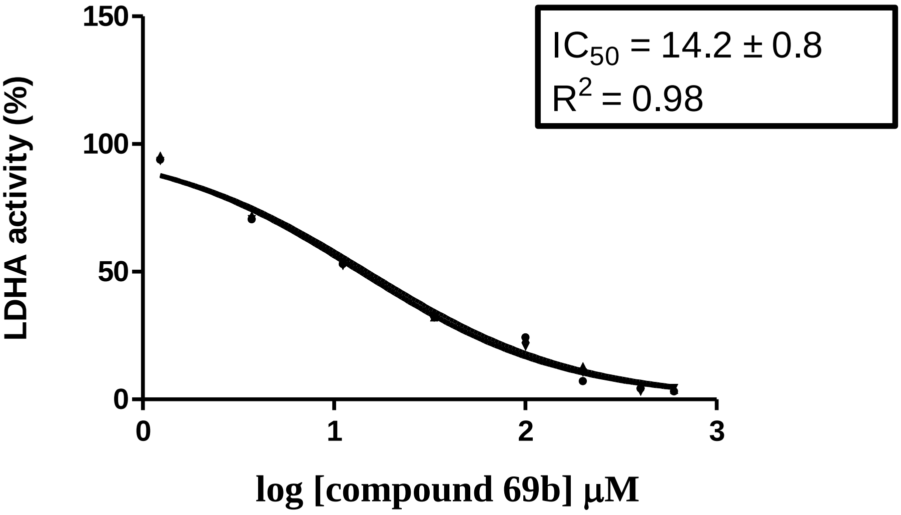

Figure S168. *h*LDHA inhibition curve of compound **69b** (mean ± SD of n = 3 replicates).

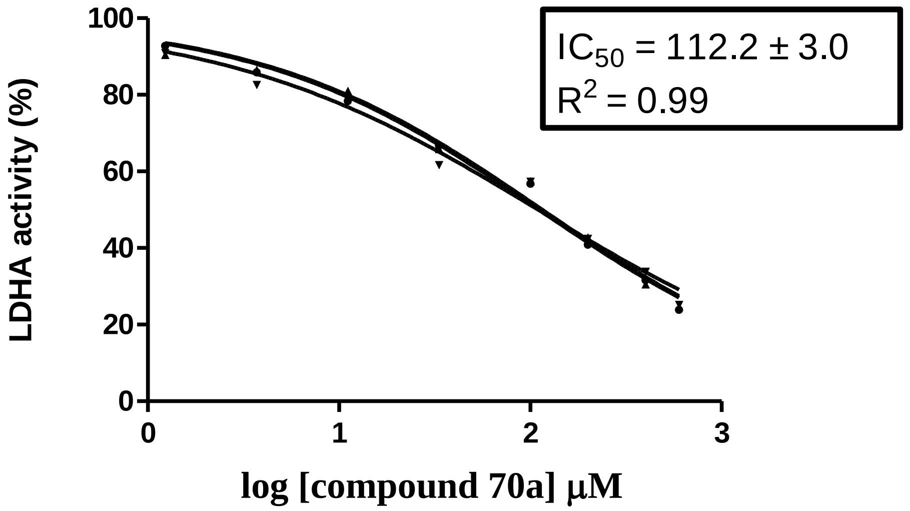

Figure S169. *h*LDHA inhibition curve of compound **70a** (mean ± SD of n = 3 replicates).

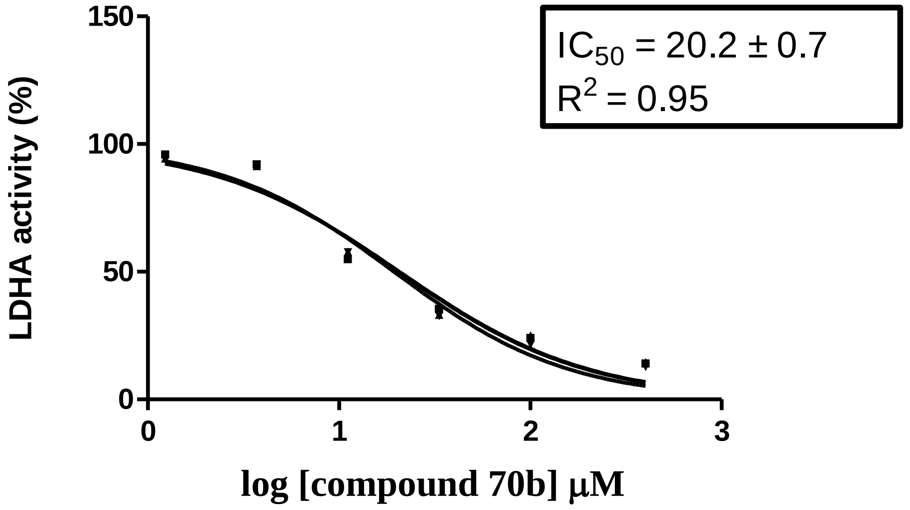

Figure S170. *h*LDHA inhibition curve of compound **70b** (mean ± SD of n = 3 replicates).

*h*LDHB

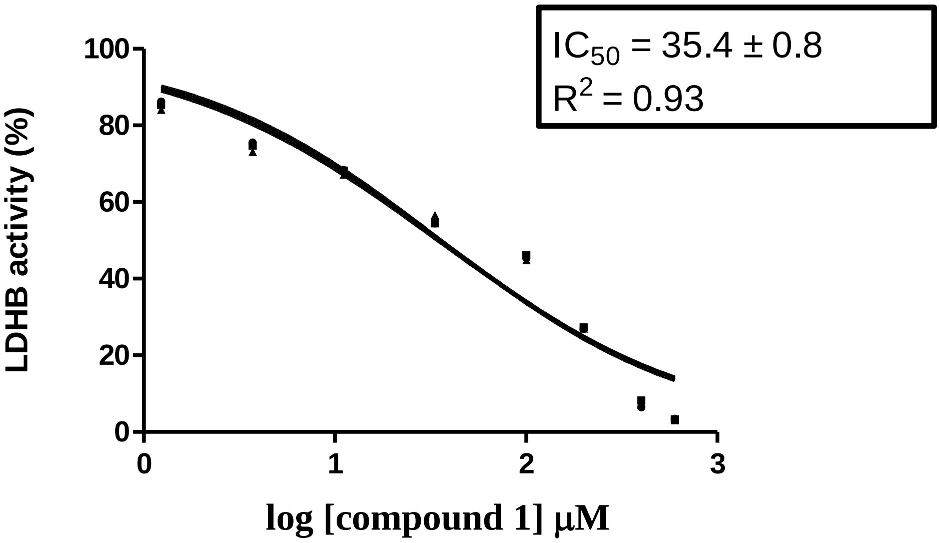

Figure S171. *h*LDHB inhibition curve of compound **1** (mean ± SD of n = 3 replicates).

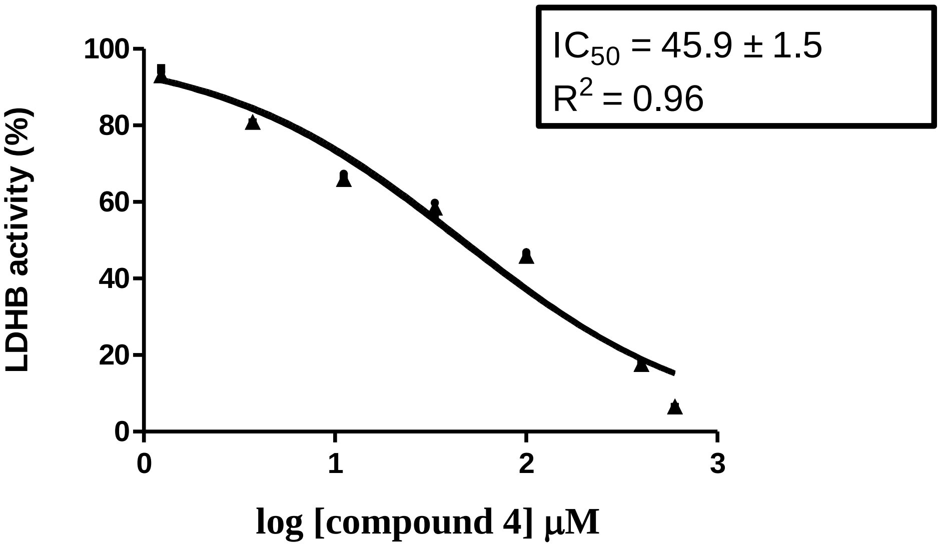

Figure S172. *h*LDHB inhibition curve of compound **4** (mean ± SD of n = 3 replicates).

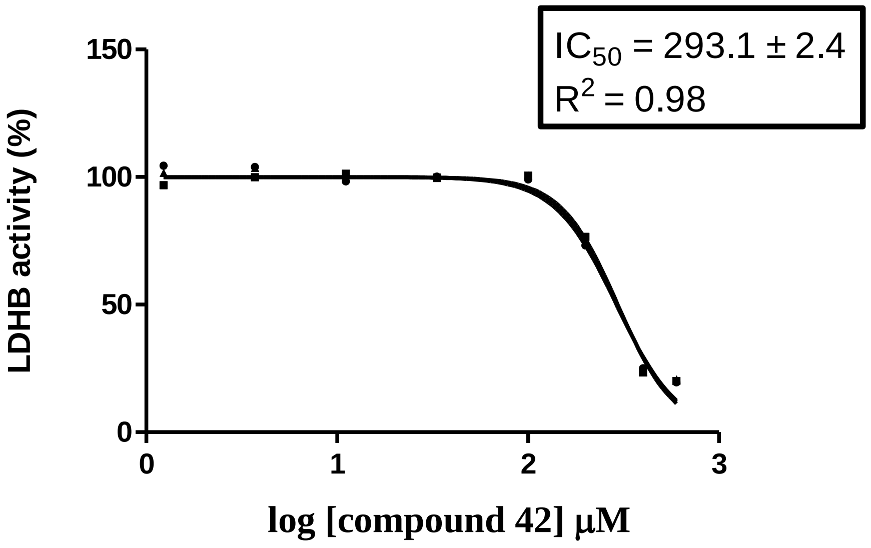

Figure S173. *h*LDHB inhibition curve of compound **42** (mean ± SD of n = 3 replicates).

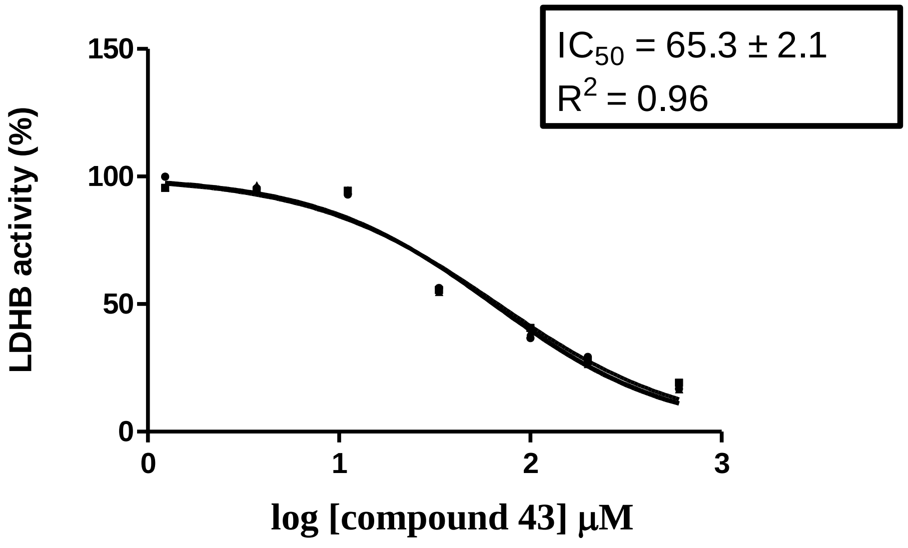

Figure S174. *h*LDHB inhibition curve of compound **43** (mean ± SD of n = 3 replicates).

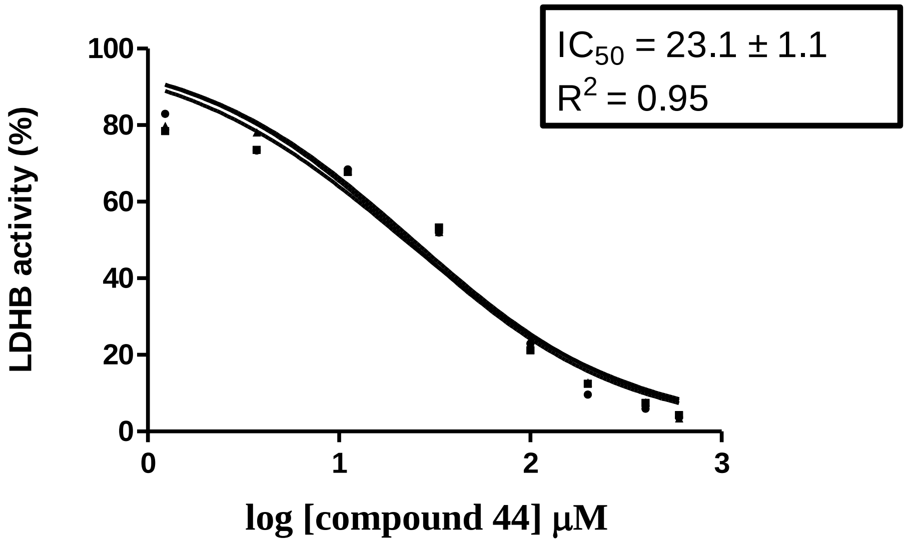

Figure S175. *h*LDHB inhibition curve of compound **44** (mean ± SD of n = 3 replicates).

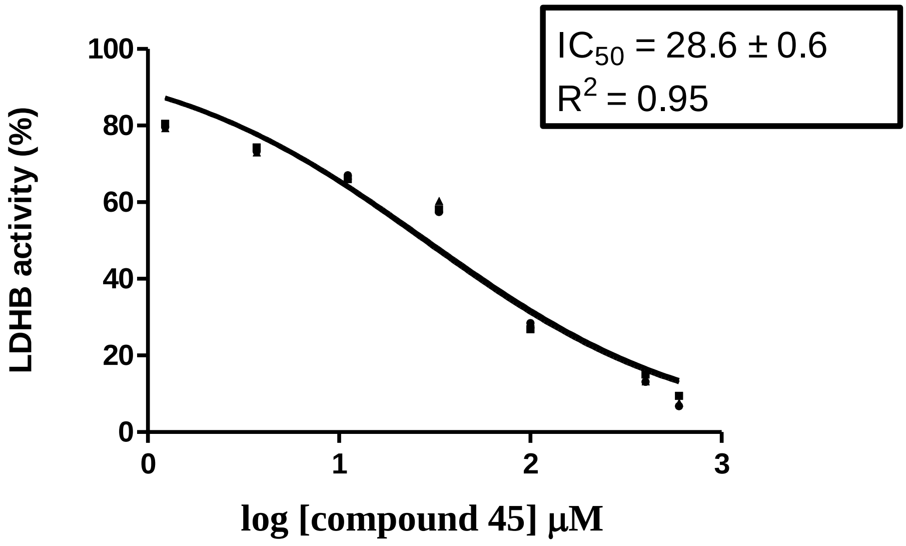

Figure S176. *h*LDHB inhibition curve of compound **45** (mean ± SD of n = 3 replicates).

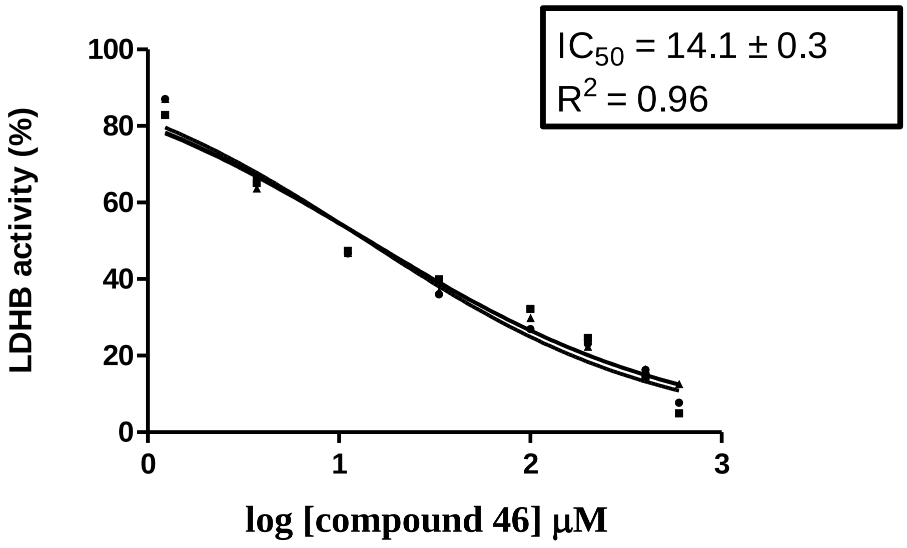

Figure S177. *h*LDHB inhibition curve of compound **46** (mean ± SD of n = 3 replicates).

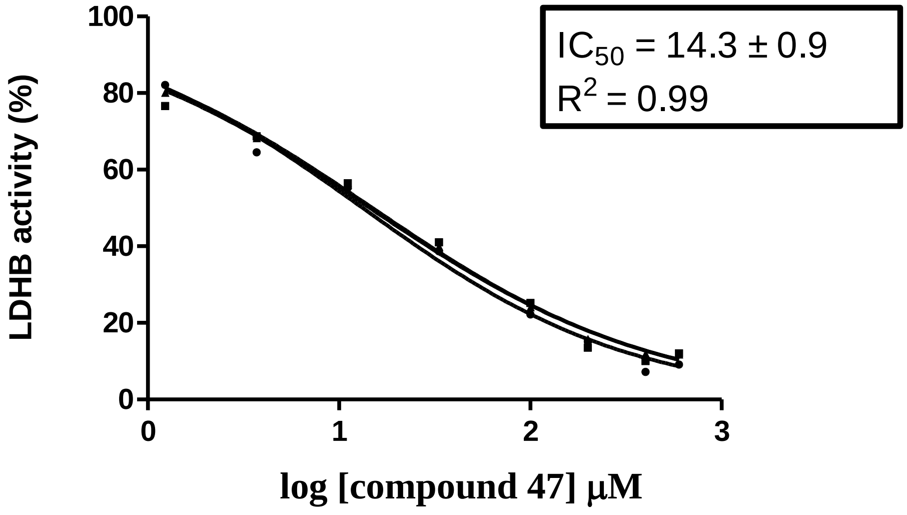

Figure S178. *h*LDHB inhibition curve of compound **47** (mean ± SD of n = 3 replicates).

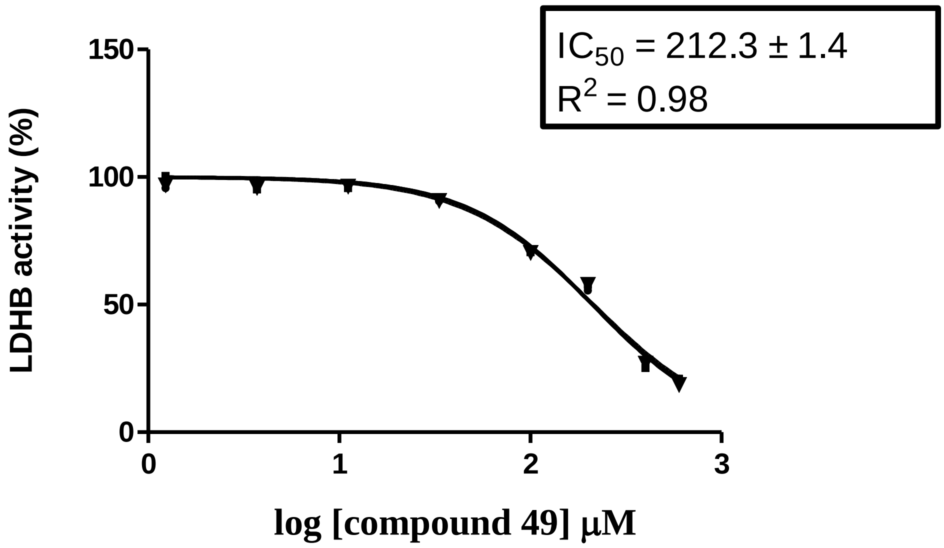

Figure S179. *h*LDHB inhibition curve of compound **49** (mean ± SD of n = 3 replicates).

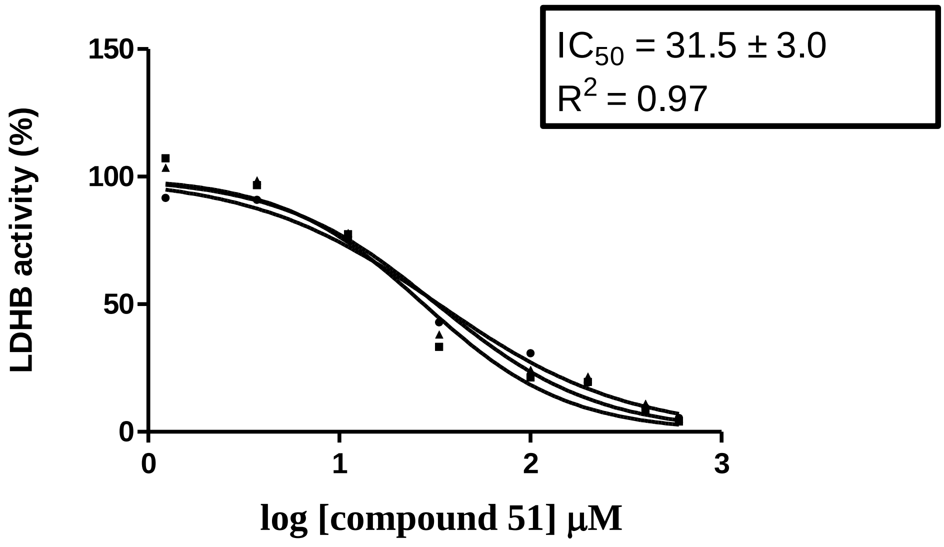

Figure S180. *h*LDHB inhibition curve of compound **51** (mean ± SD of n = 3 replicates).

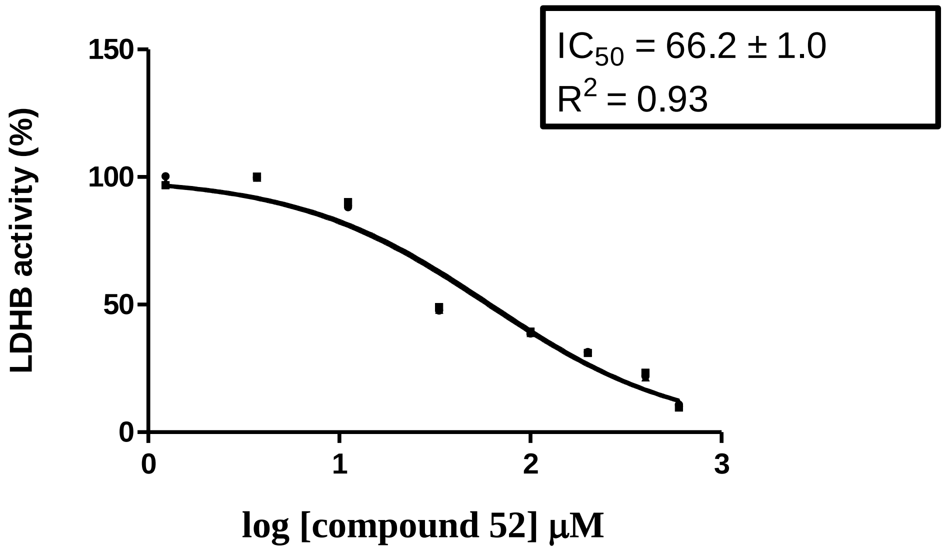

Figure S181. *h*LDHB inhibition curve of compound **52** (mean ± SD of n = 3 replicates).

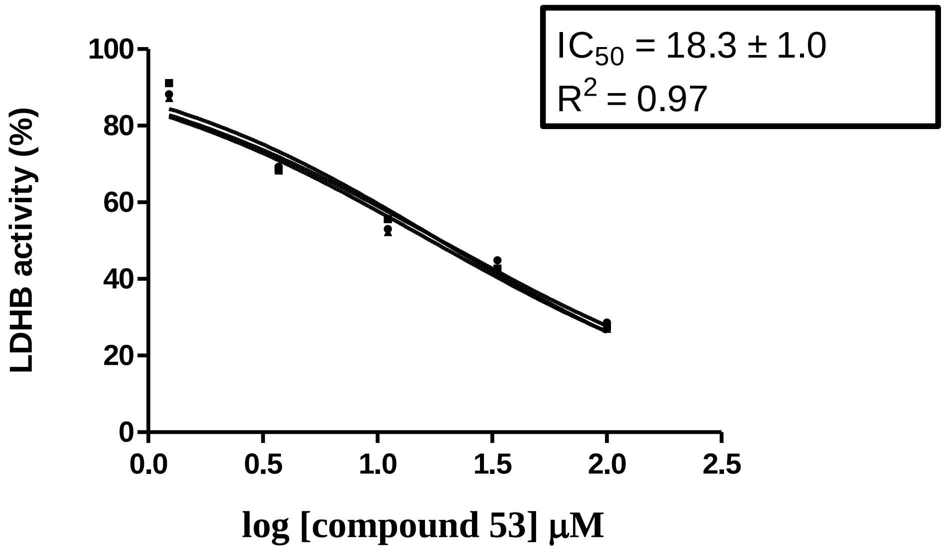

Figure S182. *h*LDHB inhibition curve of compound **53** (mean ± SD of n = 3 replicates).

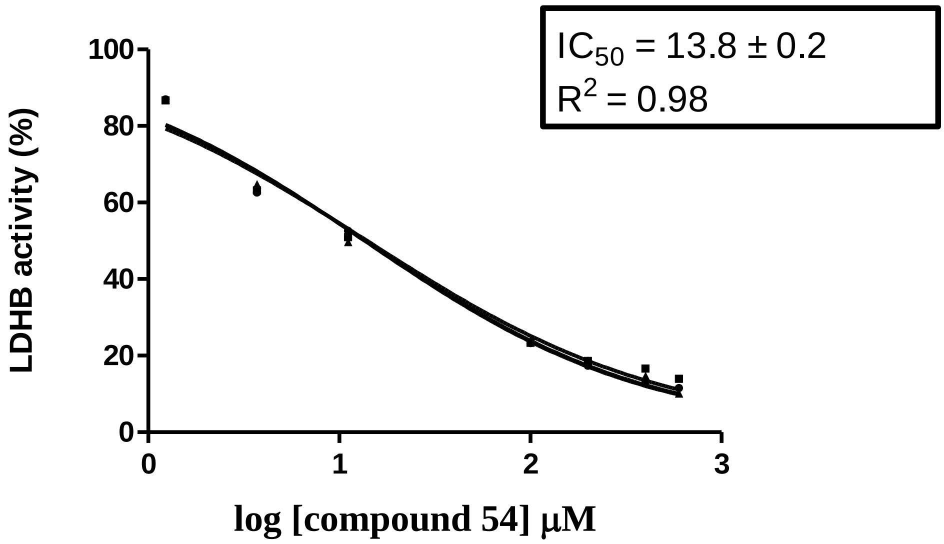

Figure S183. *h*LDHB inhibition curve of compound **54** (mean ± SD of n = 3 replicates).

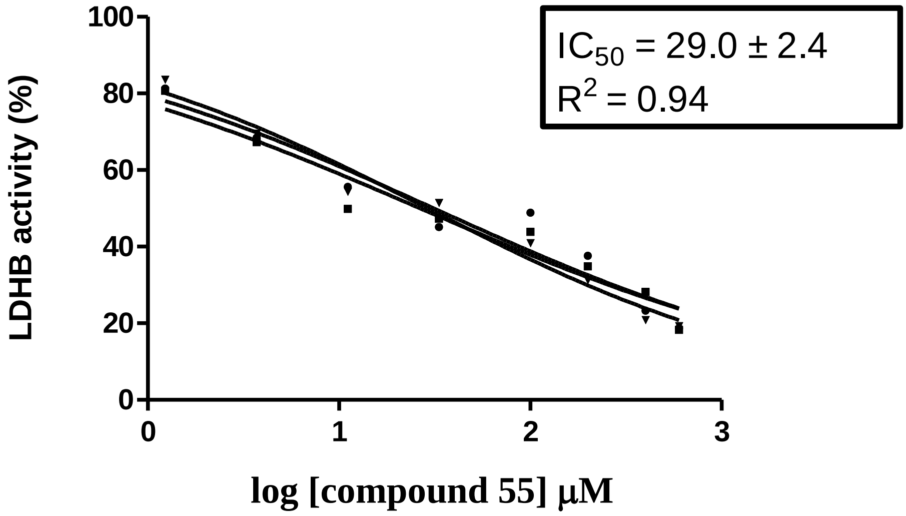

Figure S184. *h*LDHB inhibition curve of compound **55** (mean ± SD of n = 3 replicates).

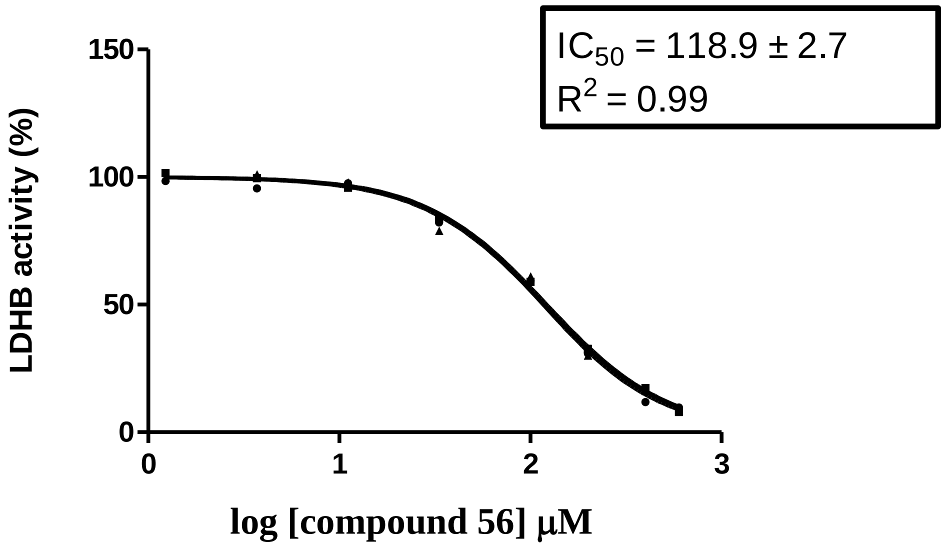

Figure S185. *h*LDHB inhibition curve of compound **56** (mean ± SD of n = 3 replicates).

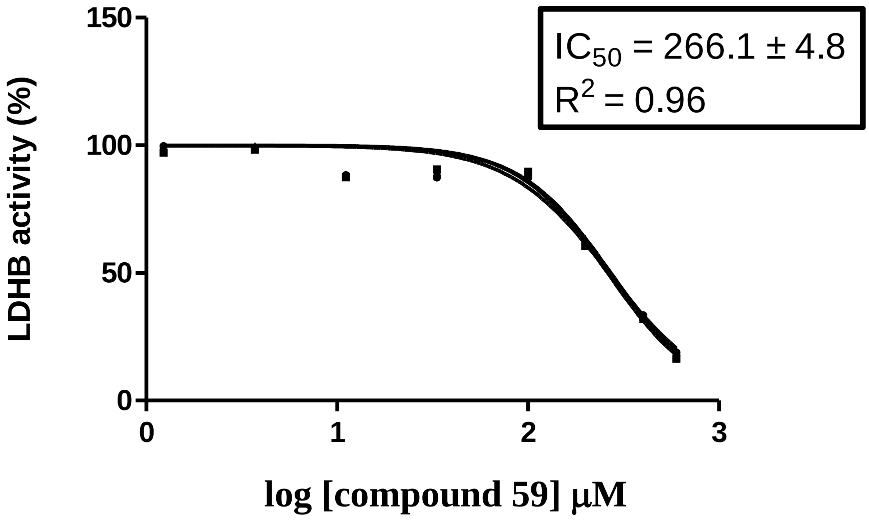

Figure S186. *h*LDHB inhibition curve of compound **59** (mean ± SD of n = 3 replicates).

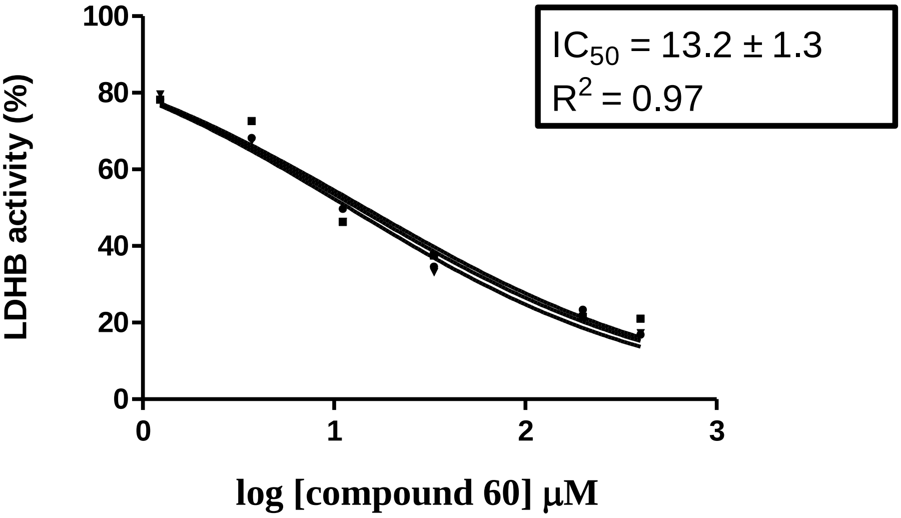

Figure S187. *h*LDHB inhibition curve of compound **60** (mean ± SD of n = 3 replicates).

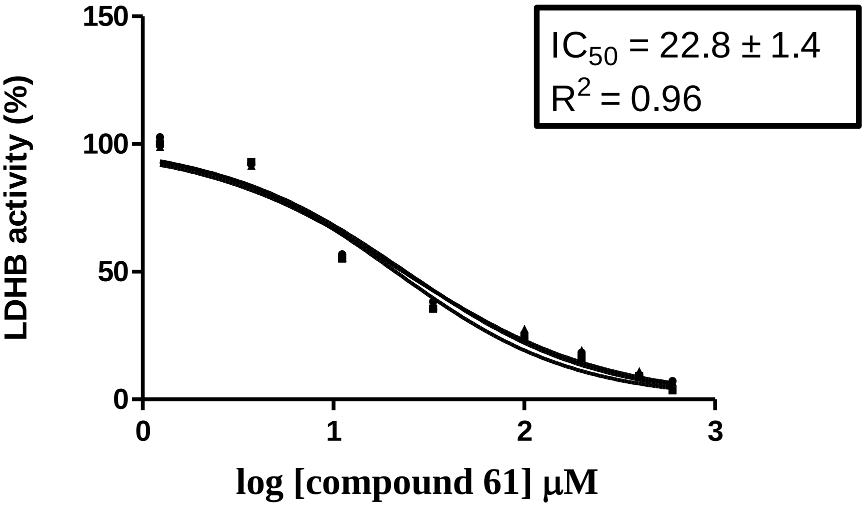

Figure S188. *h*LDHB inhibition curve of compound **61** (mean ± SD of n = 3 replicates).

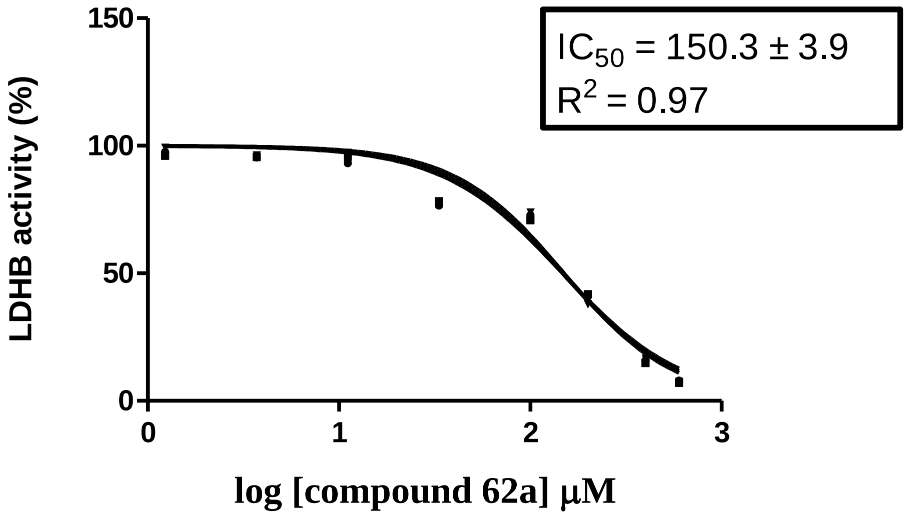

Figure S189. *h*LDHB inhibition curve of compound **62a** (mean ± SD of n = 3 replicates).

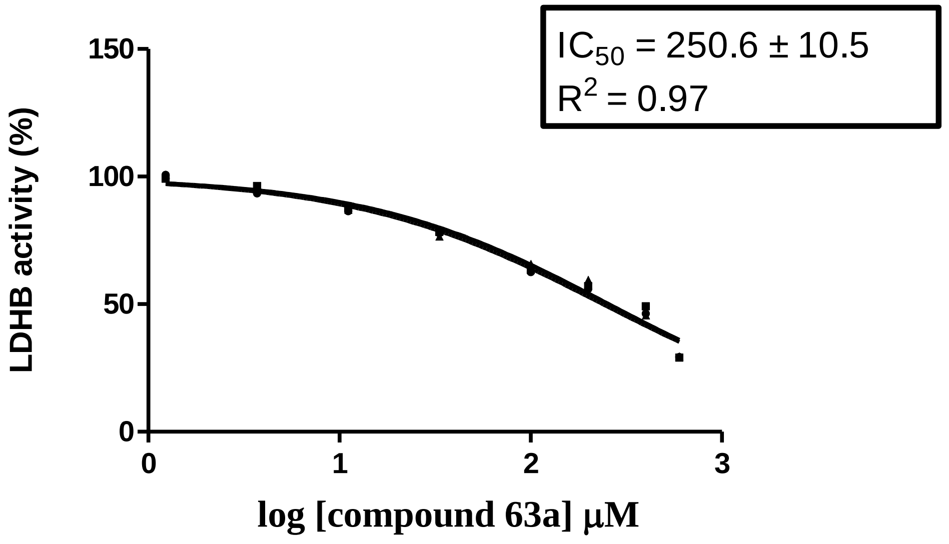

Figure S190. *h*LDHB inhibition curve of compound **63a** (mean ± SD of n = 3 replicates).

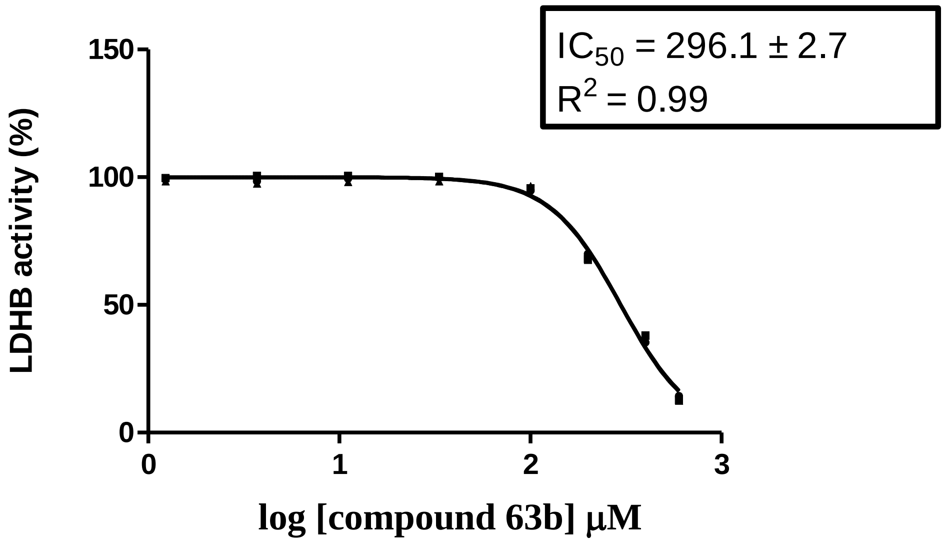

Figure S191. *h*LDHB inhibition curve of compound **63b** (mean ± SD of n = 3 replicates).

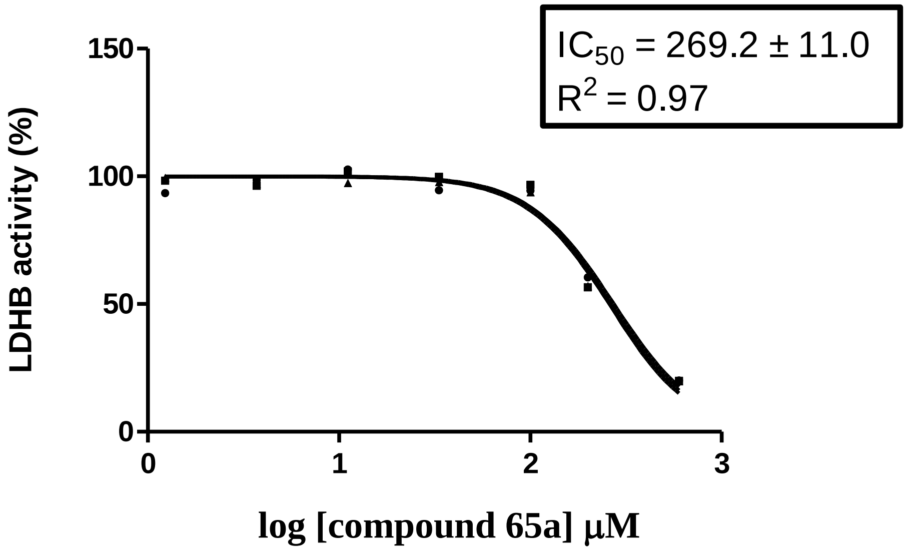

Figure S192. *h*LDHB inhibition curve of compound **65a** (mean ± SD of n = 3 replicates).

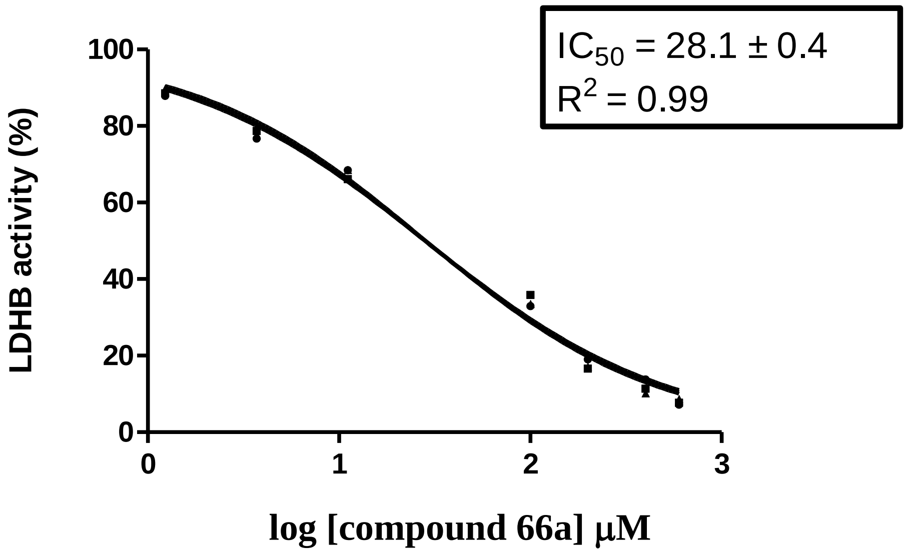

Figure S193. *h*LDHB inhibition curve of compound **66a** (mean ± SD of n = 3 replicates).

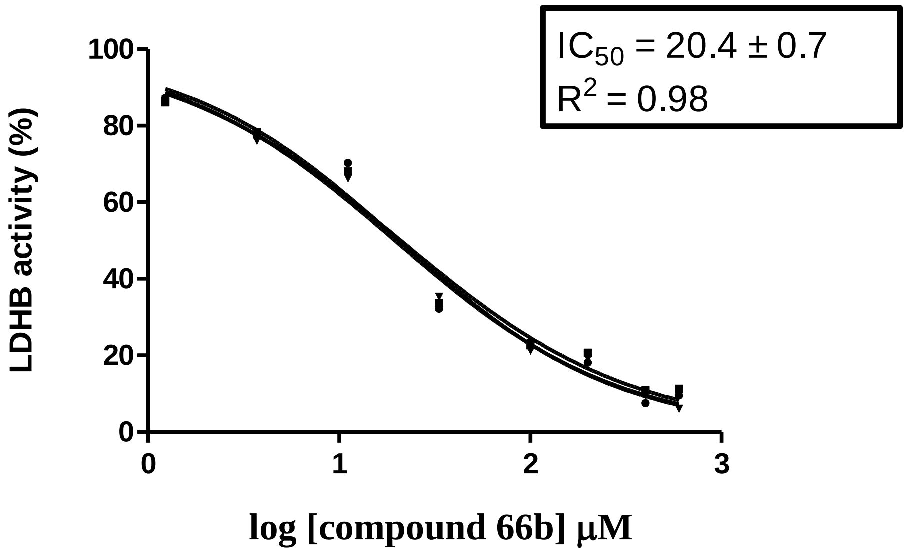

Figure S194. *h*LDHB inhibition curve of compound **66b** (mean ± SD of n = 3 replicates).

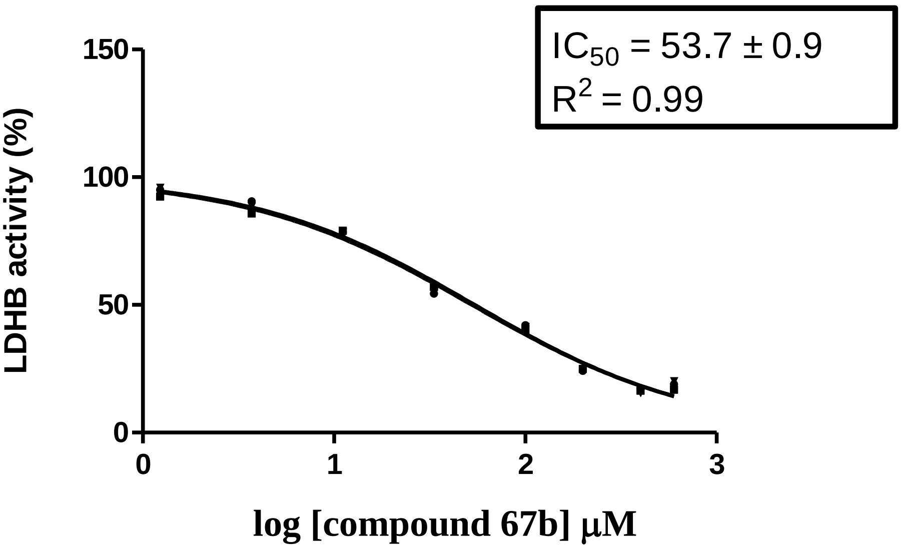

Figure S195. *h*LDHB inhibition curve of compound **67b** (mean ± SD of n = 3 replicates).

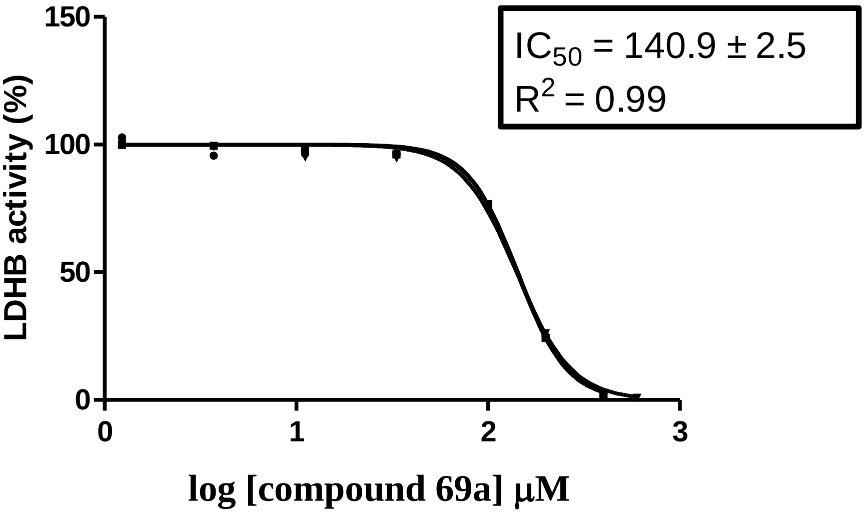

Figure S196. *h*LDHB inhibition curve of compound **69a** (mean ± SD of n = 3 replicates).

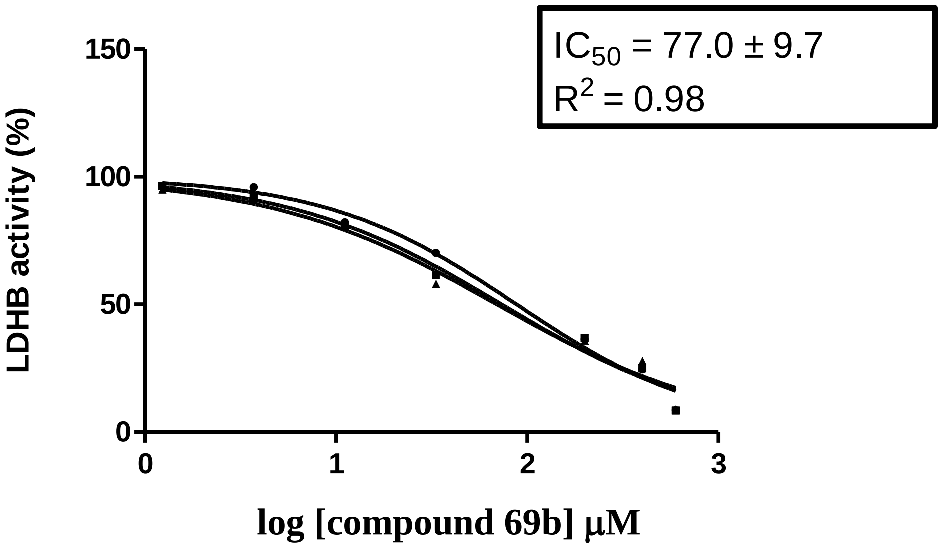

Figure S197. *h*LDHB inhibition curve of compound **69b** (mean ± SD of n = 3 replicates).

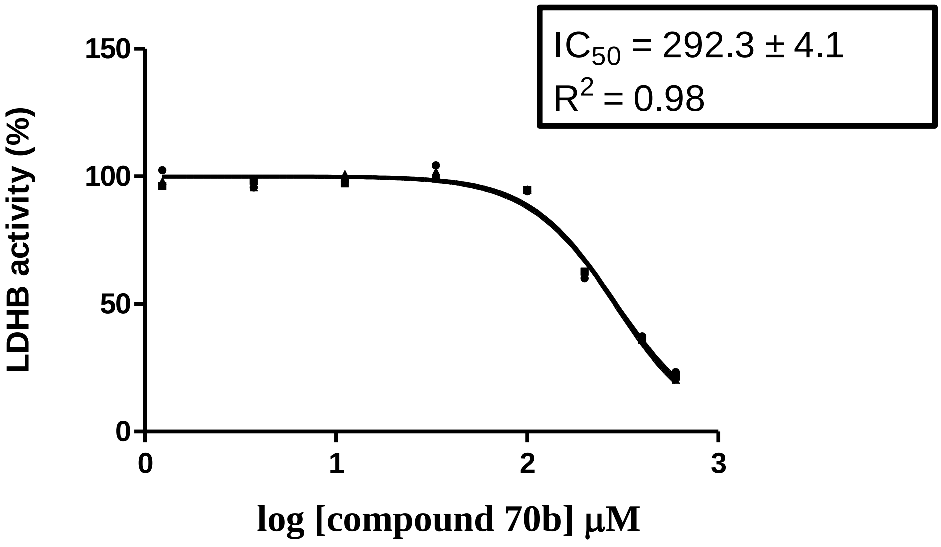

Figure S198. *h*LDHB inhibition curve of compound **70b** (mean ± SD of n = 3 replicates).

5. Dose response curves against *h*LDHA and *h*LDHB of pure enantiomers

*h*LDHA

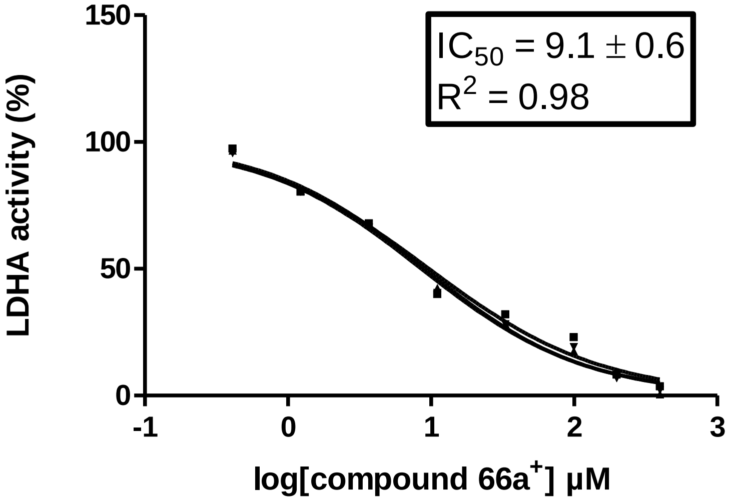

Figure S199. *h*LDHA inhibition curve of compound (+)-**66a** (mean ± SD of n = 3 replicates).

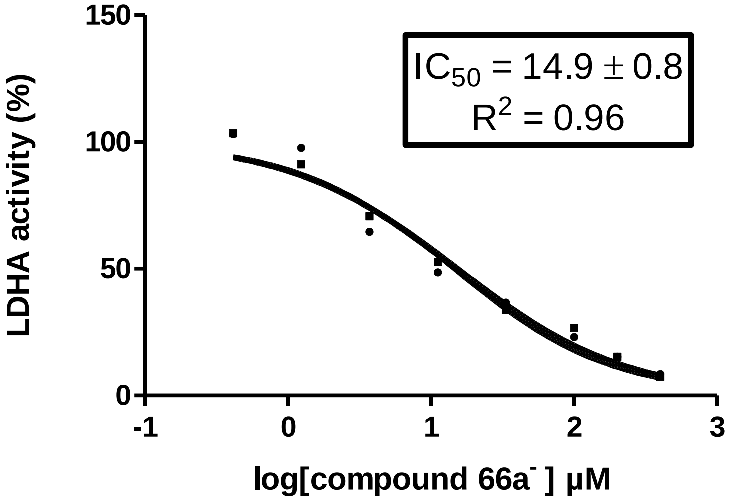

Figure S200. *h*LDHA inhibition curve of compound (−)-**66a** (mean ± SD of n = 3 replicates).

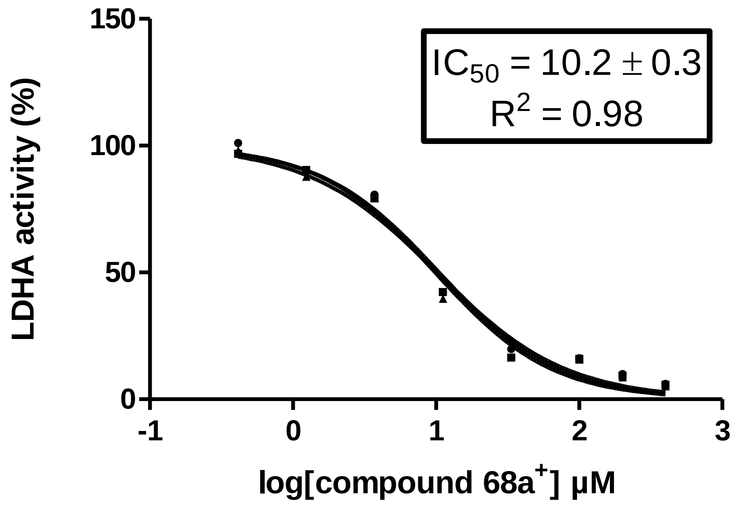

Figure S201. *h*LDHA inhibition curve of compound (+)-**68a** (mean ± SD of n = 3 replicates).

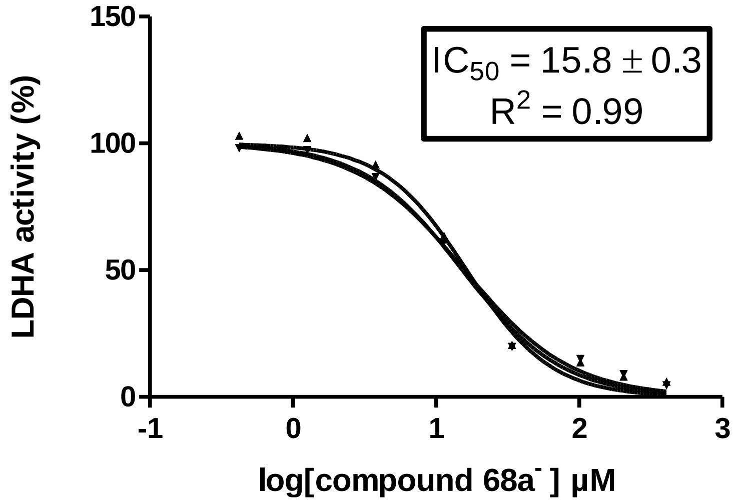

Figure S202. *h*LDHA inhibition curve of compound (−)-**68a** (mean ± SD of n = 3 replicates).

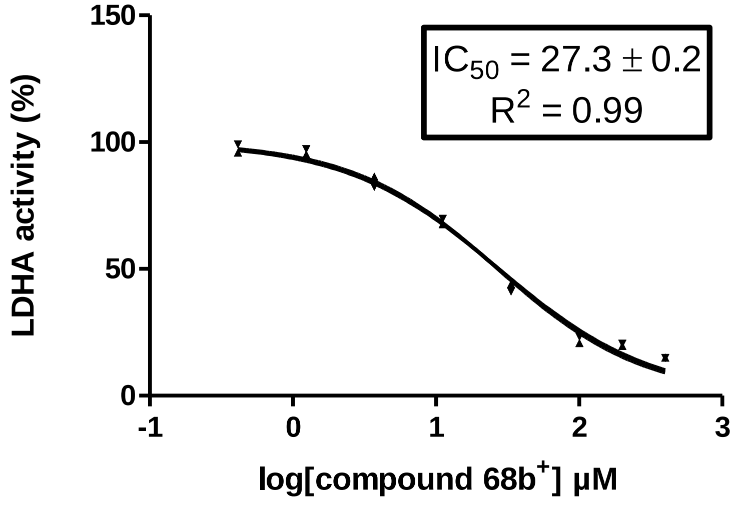

Figure S203. *h*LDHA inhibition curve of compound (+)-**68b** (mean ± SD of n = 3 replicates

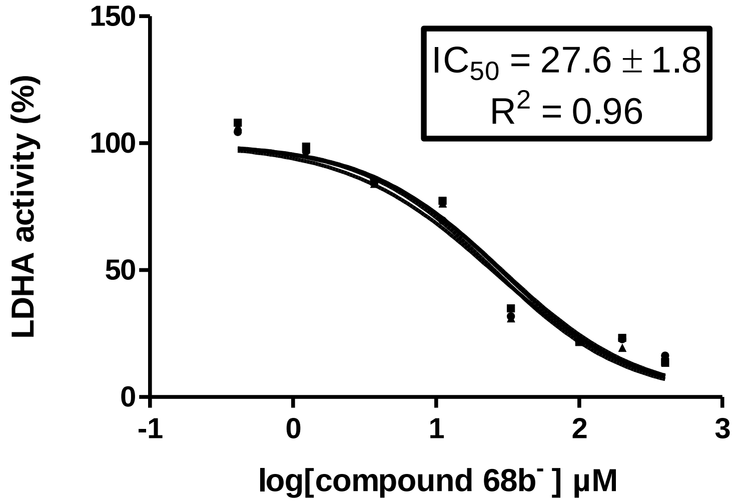

Figure S204. *h*LDHA inhibition curve of compound (−)-**68b** (mean ± SD of n = 3 replicates

***h*LDHB**

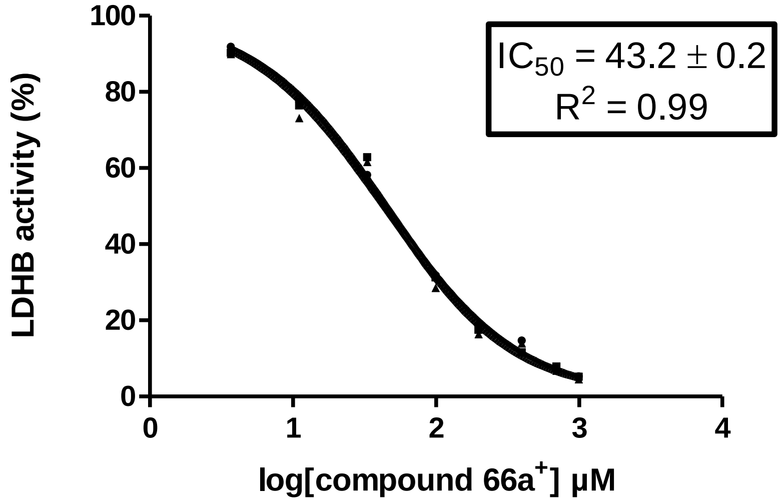

Figure S205. *h*LDHB inhibition curve of compound (+)-**66a** (mean ± SD of n = 3 replicates).

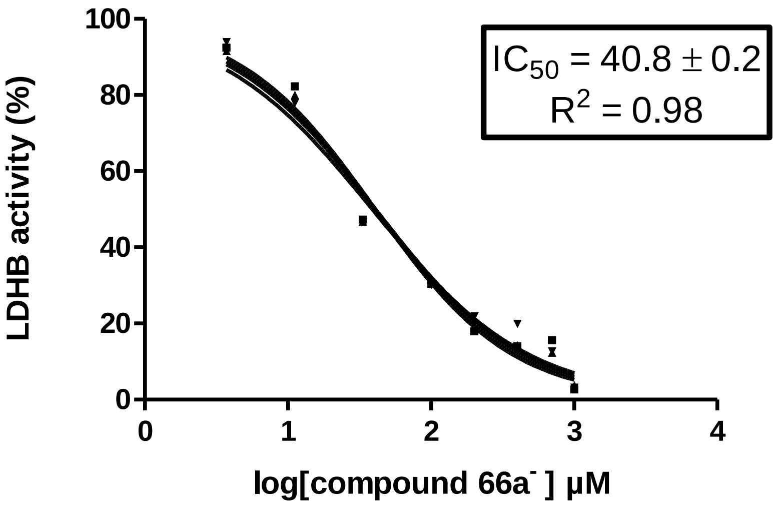

Figure S206. *h*LDHB inhibition curve of compound (−)-**66a** (mean ± SD of n = 3 replicates).

6. Lineweaver-Burk double reciprocal graphs for pure enantiomers on *h*LDHA

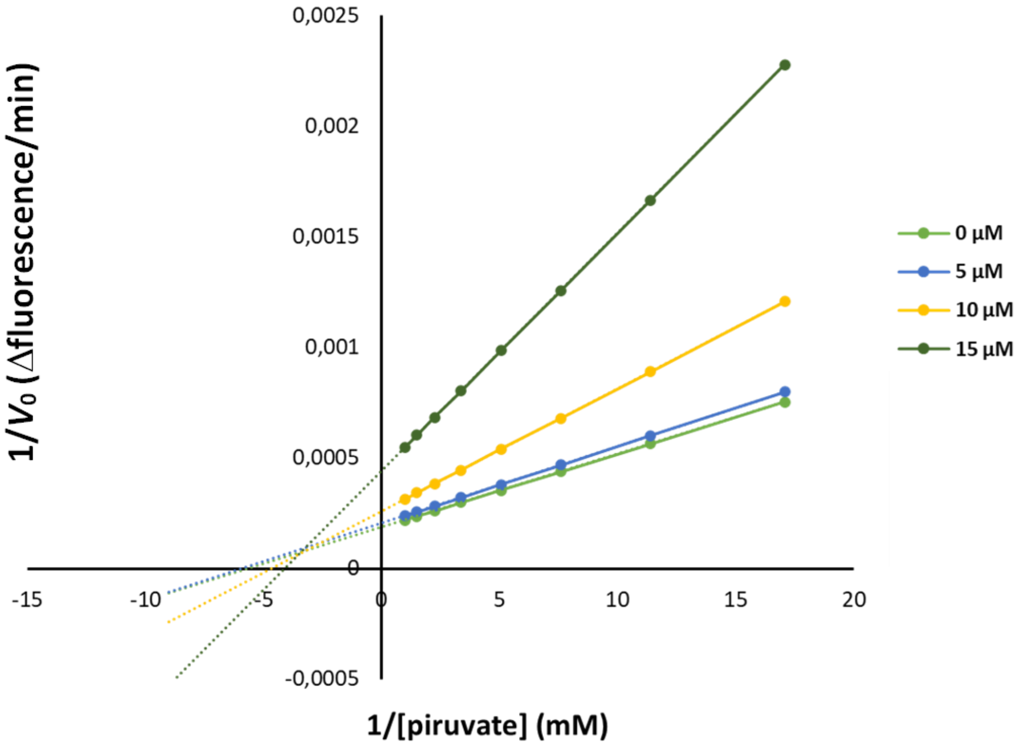

Figure S207. Lineweaver-Burk double-reciprocal graphs for (+)-**68a** (mean ± SD of n = 3 replicates) on *h*LDHA

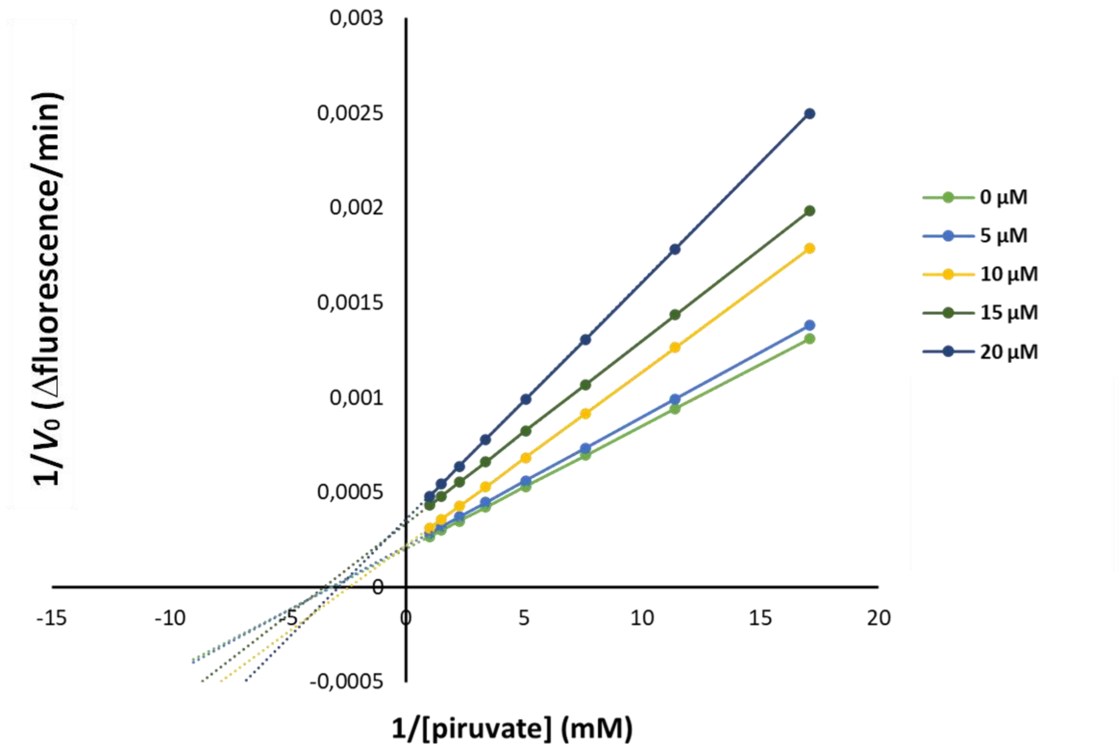

Figure S208. Lineweaver-Burk double-reciprocal graphs for (−)-**68a** (mean ± SD of n = 3 replicates) on *h*LDHA

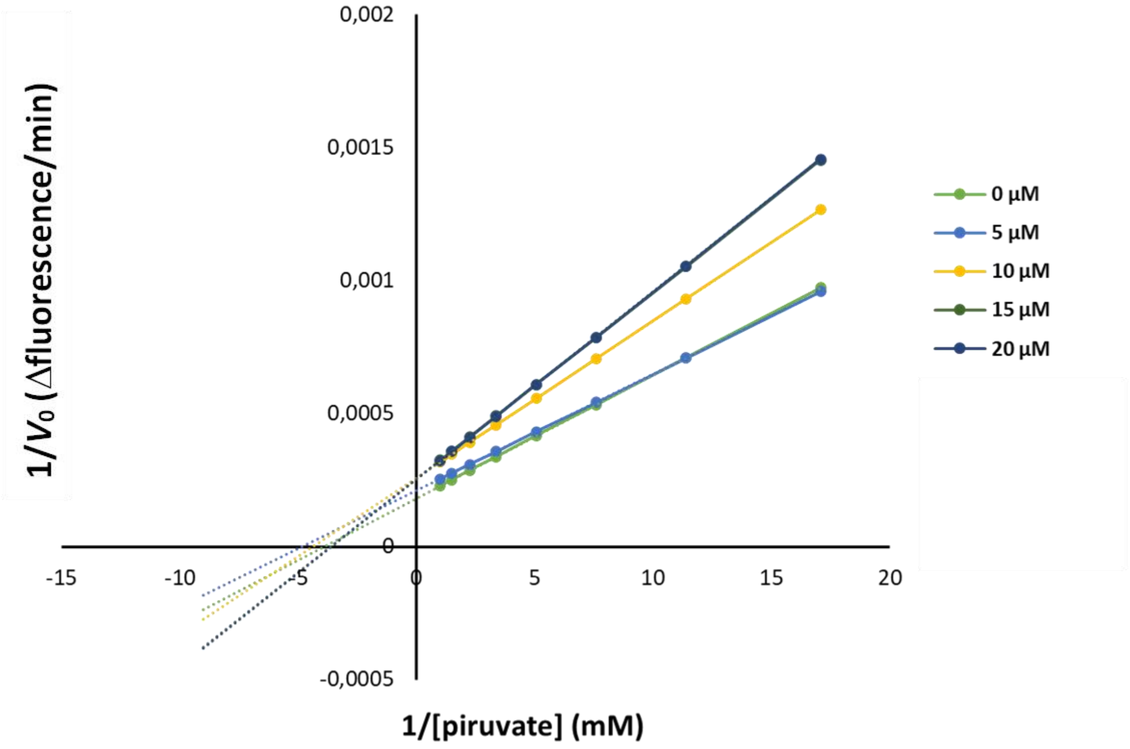

Figure S209. Lineweaver-Burk double-reciprocal graphs for (+)-**68b** (mean ± SD of n = 3 replicates) on *h*LDHA

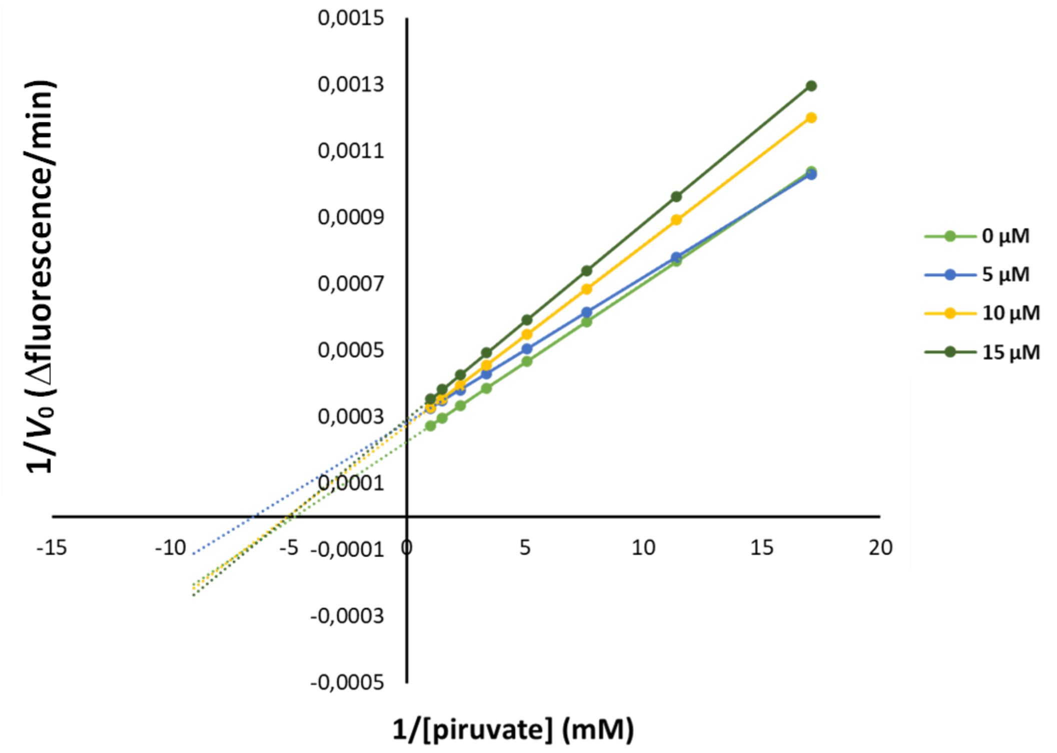

Figure S210. Lineweaver-Burk double-reciprocal graphs for (−)-**68b** (mean ± SD of n = 3 replicates) on *h*LDHA

7. Values of V_{max} and K_M for pure enantiomers on *h*LDHA

Table S1. Values of V_{max} and K_M calculated for *h*LDHA obtained at each tested concentration of (+)-**68a**

	(+)-68a			
Inhibitor concentration (µM)	**0**	**5**	**10**	**15**
V_{max} *(Δfluorescence/min)*	5374	4894	3877	2269
K_M *(µM)*	178	170	215	244

Table S2. Values of V_{max} and K_M calculated for *h*LDHA obtained at each tested concentration of (−)-**68a**

	(−)-68a				
Inhibitor concentration (µM)	**0**	**5**	**10**	**15**	**20**
V_{max} *(Δfluorescence/min)*	4916	4597	4544	2982	2809
K_M *(µM)*	318	313	416	288	352

Table S3. Values of V_{max} and K_M calculated for *h*LDHA obtained at each tested concentration of (+)-**68b**

	(+)-68b				
Inhibitor concentration (µM)	**0**	**5**	**10**	**15**	**20**
V_{max} *(Δfluorescence/min)*	5498	4753	3860	3932	3980
K_M *(µM)*	255	208	227	276	281

Table S4. Values of V_{max} and K_M calculated for *h*LDHA obtained at each tested concentration of (−)-**68b**

	(−)-**68b**			
Inhibitor concentration (μM)	**0**	**5**	**10**	**15**
V_{max} *(Δfluorescence/min)*	4421	3528	3653	3392
K_M *(μM)*	210	154	198	199
